# Molecular Signatures of Mitochondrial Complexes Involved in Alzheimer's Disease via Oxidative Phosphorylation and Retrograde Endocannabinoid Signaling Pathways

**DOI:** 10.1155/2022/9565545

**Published:** 2022-04-05

**Authors:** Fenqin Chen, Jun Bai, Shanshan Zhong, Rongwei Zhang, Xiaoqian Zhang, Ying Xu, Mei Zhao, Chuansheng Zhao, Zhike Zhou

**Affiliations:** ^1^Department of Geriatrics, The First Affiliated Hospital, China Medical University, Shenyang, 110001 Liaoning, China; ^2^Cancer Systems Biology Center, The China-Japan Union Hospital, Jilin University, Changchun, 130033 Jilin, China; ^3^Department of Neurology, The First Affiliated Hospital, China Medical University, Shenyang, 110001 Liaoning, China; ^4^Computational Systems Biology Lab, Department of Biochemistry and Molecular Biology and Institute of Bioinformatics, The University of Georgia, USA; ^5^Department of Cardiology, The Shengjing Affiliated Hospital, China Medical University, Shenyang, 110004 Liaoning, China

## Abstract

**Objective:**

The inability to intervene in Alzheimer's disease (AD) forces the search for promising gene-targeted therapies. This study was aimed at exploring molecular signatures and mechanistic pathways to improve the diagnosis and treatment of AD.

**Methods:**

Microarray datasets were collected to filter differentially expressed genes (DEGs) between AD and nondementia controls. Weight gene correlation network analysis (WGCNA) was employed to analyze the correlation of coexpression modules with AD phenotype. A global regulatory network was established and then visualized using Cytoscape software to determine hub genes and their mechanistic pathways. Receiver operating characteristic (ROC) analysis was conducted to estimate the diagnostic performance of hub genes in AD prediction.

**Results:**

A total of 2,163 DEGs from 13,049 background genes were screened in AD relative to nondementia controls. Among the six coexpression modules constructed by WGCNA, DEGs of the key modules with the strongest correlation with AD were extracted to build a global regulatory network. According to the Maximal Clique Centrality (MCC) method, five hub genes associated with mitochondrial complexes were chosen. Further pathway enrichment analysis of hub genes, such as oxidative phosphorylation and retrograde endocannabinoid signaling, was identified. According to the area under the curve (AUC) of about 70%, each hub gene exhibited a good diagnostic performance in predicting AD.

**Conclusions:**

Our findings highlight the perturbation of mitochondrial complexes underlying AD onset, which is mediated by molecular signatures involved in oxidative phosphorylation (COX5A, NDUFAB1, SDHB, UQCRC2, and UQCRFS1) and retrograde endocannabinoid signaling (NDUFAB1) pathways.

## 1. Introduction

Alzheimer's disease (AD), accounting for 60~80% of all dementias, is rapidly becoming a devastating, epidemic, and costly neurodegenerative disease [1, 2]. It is also known as the third “health killer” worldwide, a serious threat to the life security of the elderly, with the number of cases predicted to rise to 152 million by 2050 [3]. Pathologically, the core hallmarks of AD are the accumulation of beta-amyloid (A*β*) peptide and hyperphosphorylated tau, which constitute extracellular senile plaques and intracellular neurofibrillary tangles, respectively [4]. With the increase of deposits, this clinical entity presents with progressive cognitive decline, accompanied by late behavioral and psychiatric abnormalities [1]. Such a well-defined pathology and slow-progressing course of AD offer no opportunity for intervention; that is, there are currently no Food and Drug Administration- (FDA-) approved pharmacotherapies (including the common clinical use of rivastigmine, galantamine, donepezil, and memantine) that can stop or even delay the occurrence and development of disease [5–7]. Accordingly, much effort has been directed towards genomic identification of novel targets, which may be the optimal strategy for early diagnosis and therapy of AD.

With advances in high-throughput sequencing and microarray technology, bioinformatics analysis is cumulatively employed to investigate genetic factors that contribute to the risk of AD [8]. Several AD-related genes (e.g., PP2A, RGS2, TOMM40, and APOE4) have been recognized as potential candidates in an attempt to predict individual susceptibility, provide targeted prevention, and create personalized treatment [9–11]. Nevertheless, integrated genomic analysis has not been systematically applied to AD research [12]. Indeed, previous outcomes were mainly derived from a single dataset with insufficient sample size and monocentric study design in Gene Expression Omnibus (GEO) database, an invaluable resource of a publicly available genomic repository affording gene expression data and clinical phenotypes [13]. The obtained molecular signatures may simply represent the genetic predisposition of a small population with AD, a potential interpretation for the inapplicability of partial outcomes in clinical practice. In view of this, we attempted to excavate novel molecular targets and decipher their mechanistic pathways via integrated genomic analysis on multiple datasets, aiming to provide more authentic and trustworthy results to gain insight into the pathogenesis of AD.

Throughout the GEO database, five gene expression profiles of GSE132903, GSE118553, GSE5281, GSE37264, and GSE36980 that met the inclusion criteria were collected for this analysis. First, we compared the gene expression data from AD tissues and non-AD tissues, which have fundamentally different biological properties to generate differentially expressed genes (DEGs). Subsequently, all DEGs were grouped into six modules with high statistical significance by weighted gene coexpression network analysis (WGCNA), a commonly used and proven approach for bioinformatics analysis of coexpressed genes, indicating that genes in each module are transcriptionally correlated and work together in a coordinated fashion. Furthermore, pathway enrichment analysis over genes in each module was conducted to determine in what cellular processes the correlated genes wrought in, hence providing a cellular level understanding of the coexpressed genes. We noted that brown and turquoise modules exhibited the strongest positive/negative correlation with AD, implying the close relationship of their enrichment pathways with AD. Having all these statistical results, we have then built a global regulatory network to identify hub genes and their pathogenic processes, for how these molecular targets are causally linked to AD. The findings of this study contribute to our understanding of the underlying genomic mechanisms of AD, which may provide key clues for diagnostic and therapeutic strategies based on these molecular signatures.

## 2. Materials and Methods

### 2.1. Data Resources

Microarray datasets including GSE132903, GSE118553, GSE5281, GSE37264, and GSE36980 were downloaded from the GEO database. The RNA sequencing (RNA-seq) data were derived from temporal lobe tissues of postmortem human brains. GSE132903 collected the middle temporal gyrus from AD cases (*n* = 97) and nondementia controls (*n* = 98); GSE118553 contained the temporal cortex from AD (*n* = 45) and control subjects (*n* = 24); GSE5281 gathered the middle temporal gyrus from AD patients (*n* = 16) and controls (*n* = 11); GSE37264 included the temporal cortex from AD cases (*n* = 8) and controls (*n* = 8); GSE36980 obtained the temporal cortex from AD (*n* = 10) and nondementia controls (*n* = 19). Both the middle temporal gyrus and temporal cortex belong to the temporal lobe, a preferential region that is susceptible to AD neurodegeneration [14, 15] and has commonly been analyzed in genomic studies [16–18]. All individuals in the case group can be considered as sporadic AD patients based on the original literature (or recruitment studies) for each dataset [19–23]. In total, 336 participants including 176 AD (male/female: 89/87) and 160 gender-matched controls (male/female: 89/87; *p* = 0.64) were enrolled. The average age for AD patients was 83.60 ± 8.25 (range: 40-105) years relative to 81.38 ± 10.78 (range: 43-102) years for controls. Demographic data of each subject were detailed in Supplementary Table 1. Participants with pathological and/or clinical diagnosis of AD should meet one of following standardized criteria: (1) the Diagnostic and Statistical Manual of Mental Disorders- (DSM-) III, DSM-IV, or DSM-V criteria [24–27]; (2) the International Classification of Diseases- (ICD-) 10 criteria; (3) the National Institute of Neurological and Communicative Disorders and Stroke-Alzheimer's Disease and Related Disorders Association (NINCDS-ADRDA) [28]; and (4) the Consortium to Establish a Registry for Alzheimer's Disease (CERAD) guidelines [29] and the Braak stage [14].

### 2.2. Data Preprocessing and DEG Screening

Before processing of gene data, GSE113439 and GSE53408 were annotated according to the GPL10558 platform of Illumina Human HT-12 v4 arrays expression beadchip; GSE5281 was obtained based on the GPL570 platform of Affymetrix Human Genome U133 Plus 2.0 Array; GSE37264 was available from the GPL5188 platform of Affymetrix Human Exon 1.0 ST Array; GSE36980 was profiled on the GPL6244 platform of Affymetrix Human Gene 1.0 ST Array. Gene expression data of all datasets were uniformly normalized employing the *normalizeBetweenArrays* function of limma package in *R* software [30]. Referring to the annotation profile of each dataset, we converted the probes into gene symbols or deleted unlabeled probes. If multiple probes were annotated to a single gene, the one with the highest expression value was retained. RNA-seq data from five datasets were merged into a new dataset, and their batch effects were eliminated using the *removeBatchEffect* function. Gene expression between AD and control tissues was compared to screen DEGs by *lmFit* and *eBayes* functions. The limma package was adopted for two-dimensional hierarchical clustering analysis of DEGs, with the results visualized by volcano plots. A false discovery rate- (FDR-) adjusted *p* < 0.05 and fold change (FC) ≥ 1.3 were considered statistically significant [30–32].

### 2.3. Weighted Gene Coexpression Network Analysis

Using WGCNA package in R software [33], a gene coexpression network was constructed to investigate the expression and interaction of DEGs in AD. First, gene expression values were standardized into fragments per kilobase of transcript per million mapped reads (FPKM), and sample clustering was carried out to remove outliers using the *hclust* function. Next, Pearson correlation coefficients of all gene pairs were calculated, by which an eigengene network matrix reflecting the similarity between genes was generated. The soft thresholding power (*β* value) of 7 was selected to ensure the scale-free network, hence transforming the similarity matrix into an adjacency matrix. A topological overlap matrix (TOM) was then established to measure the average network connectivity of each gene. Based on the relevant parameters (mergeCutHeight = 0.25 and minModuleSize = 30), genes with similar expression profiles were classified into different modules through the dynamic tree cutting method. Finally, a cluster dendrogram was plotted by hierarchical clustering to assess the correlation of module eigengenes (MEs) with clinical phenotypes (e.g., AD, age, and gender). Among coexpression modules, the one with the highest negative or positive correlation with AD was defined as the key module. Detailed information of DEGs in module-trait relationships is listed in Supplementary Table 2.

### 2.4. Functional Enrichment Analysis of Coexpression Modules

Employing the packages of org.Hs.eg.db and clusterProfiler, functional annotations of DEGs in coexpression modules were accomplished by Kyoto Encyclopedia of Genes and Genomes (KEGG) pathway enrichment analysis [34]. The biological significance of each module was exhibited in the form of a bar chart. In this analysis, *p*valueCutoff = 0.05 was set as the enrichment of KEGG pathway with statistical significance.

### 2.5. Global Regulatory Network Construction, Hub Gene Selection, and Pathway Enrichment Analysis

Through the online STRING (Search Tool for the Retrieval of Interacting Genes, https://www.string-db.org/) database [35], DEGs of key modules were uploaded to build a global regulatory network. The results of protein-protein information (PPI) were further analyzed and visualized by using Cytoscape (version 3.8.2) software [36]. In the global regulatory network, the cytoHubba plugin was adopted to identify hub genes through the Maximal Clique Centrality (MCC) method [37]. Moreover, the mechanistic pathways of hub genes were enriched applying ClueGO plugin [38].

### 2.6. Diagnostic Performance Assessment

Utilizing the *pROC* function in R software, the receiver operating characteristic (ROC) analysis was performed to evaluate the diagnostic performance of hub genes in distinguishing AD from nondementia controls [39]. The area under the curve (AUC) value was calculated to quantify the sensitivity and specificity of ROC analytic results. In general, an almost perfect prediction is represented by an AUC value of close to 100%, while a random selection of AUC is closed to 50%. For all analyses, statistical significance is assigned at *p* values less than 0.05 (*p* < 0.05).

## 3. Results

### 3.1. Differentially Expressed Genes

**Figure 1:**
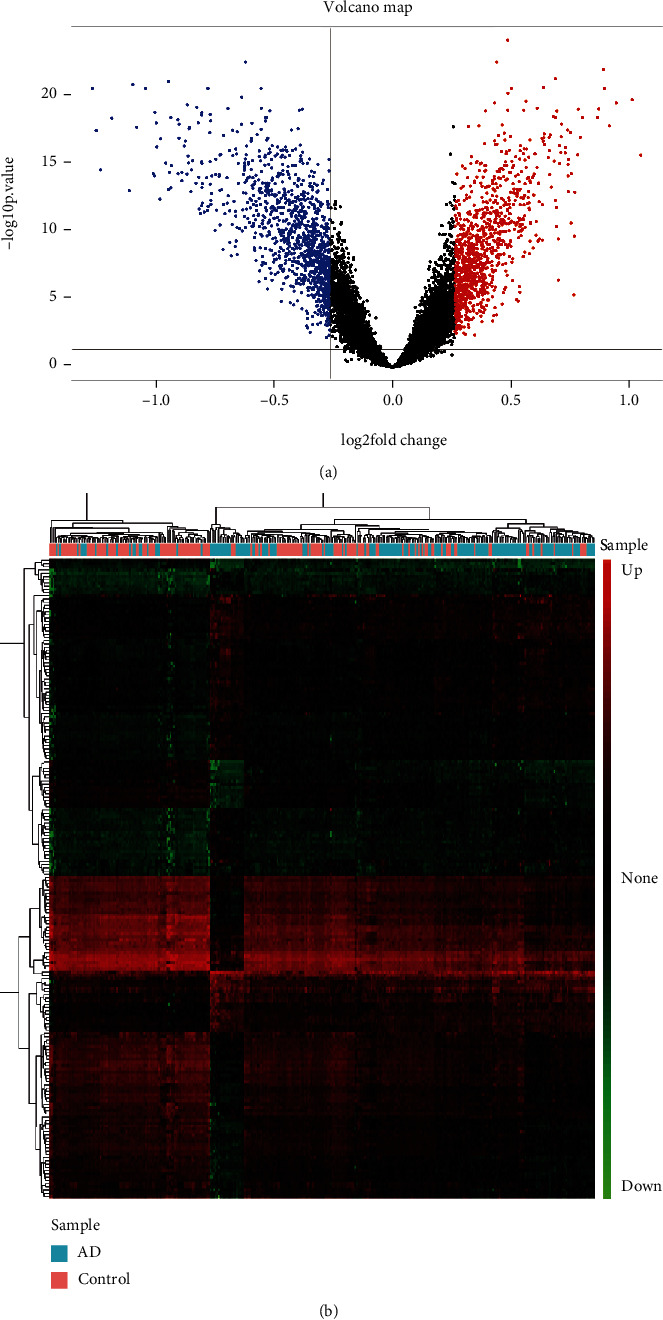
Visualization of differential expression analysis. (a) Volcano plot of the genes between AD and nondementia controls: red and blue indicate upregulation and downregulation, respectively. (b) Heatmap of differentially expressed genes: color alterations from green to red represent gene expression from downregulation to upregulation. AD: Alzheimer's disease.

After preprocessing the gene expression profiles through annotation, merging, and normalization, we used the limma package to filtrate DEGs between AD cases and nondementia controls. Gene expression analysis showed that 2,163 out of 13,049 background genes were differentially expressed in AD relative to nondementia controls. Volcano plot exhibited the number of DEGs identified from the merged gene expression data (Figure 1(a)). Heatmap displayed the expression of DEGs between AD cases and controls (Figure 1(b)).

### 3.2. Coexpression Modules and Functional Enrichment Analysis

**Figure 2:**
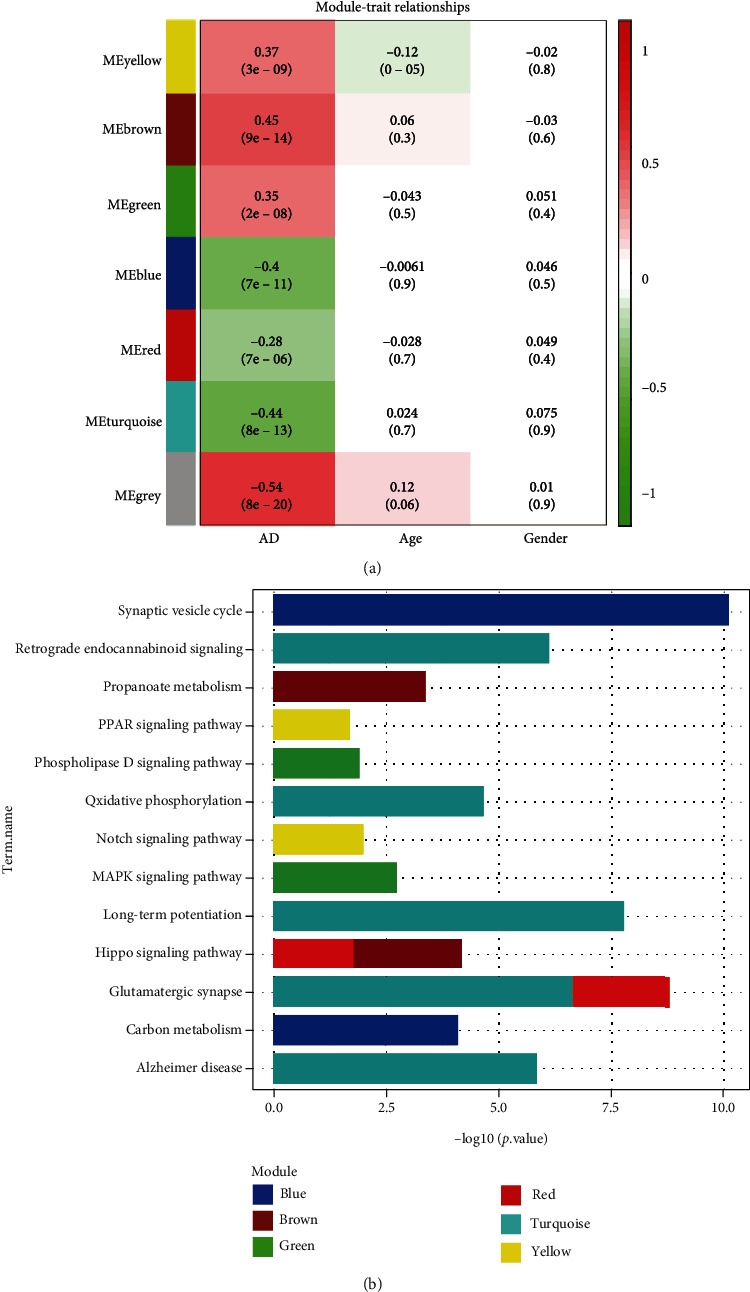
Weighted correlation network analysis and module-pathway enrichment. (a) Module-trait relationships: color changes from green to red indicate the correlation between MEs and clinical phenotypes from negative to positive. (b) KEGG pathway enriched by coexpressed genes in each module. AD: Alzheimer's disease; KEGG: Kyoto Encyclopedia of Genes and Genomes; MEs: module eigengenes.

In the process of hierarchical sample clustering through average linkage, 245 eligible subjects were incorporated according to cut-off height of 40. WGCNA were used to cluster DEGs into seven uniquely colored modules, wherein non-co-expressed genes were grouped into the grey module, implying that they participated in abiotic processes. Heatmap of module-trait relationships (Figure 2(a)) showed the diminishing negative correlation of turquoise (correlation coefficient = −0.44, *p* = 8*e* − 13), blue (correlation coefficient = −0.4, *p* = 7*e* − 11), and red (correlation coefficient = −0.28, *p* = 7*e* − 06) modules with AD phenotype, while brown (correlation coefficient = 0.45, *p* = 9*e* − 14), yellow (correlation coefficient = 0.37, *p* = 3*e* − 09), and green (correlation coefficient = 0.35, *p* = 2*e* − 08) modules were degressively positively correlated with AD. Annotation results of the KEGG pathway (Figure 2(b)) revealed that DEGs in the blue module were enriched in synaptic vesicle cycle and carbon metabolism; DGEs in the brown module were related to propanoate metabolism and hippo signaling pathway; DEGs in the green module were involved in mitogen-activated protein kinase (MAPK) and phospholipase D signaling pathways; DEGs in the red module had implications in glutamatergic synapse and hippo signaling pathway; DGEs of the turquoise module participated in long-term potentiation, glutamatergic synapse, retrograde endocannabinoid signaling, and oxidative phosphorylation; and DEGs in the yellow module were associated with Notch and peroxisome proliferator-activated receptor (PPAR) signaling pathways.

### 3.3. Global Regulatory Network and ROC Analysis

**Figure 3:**
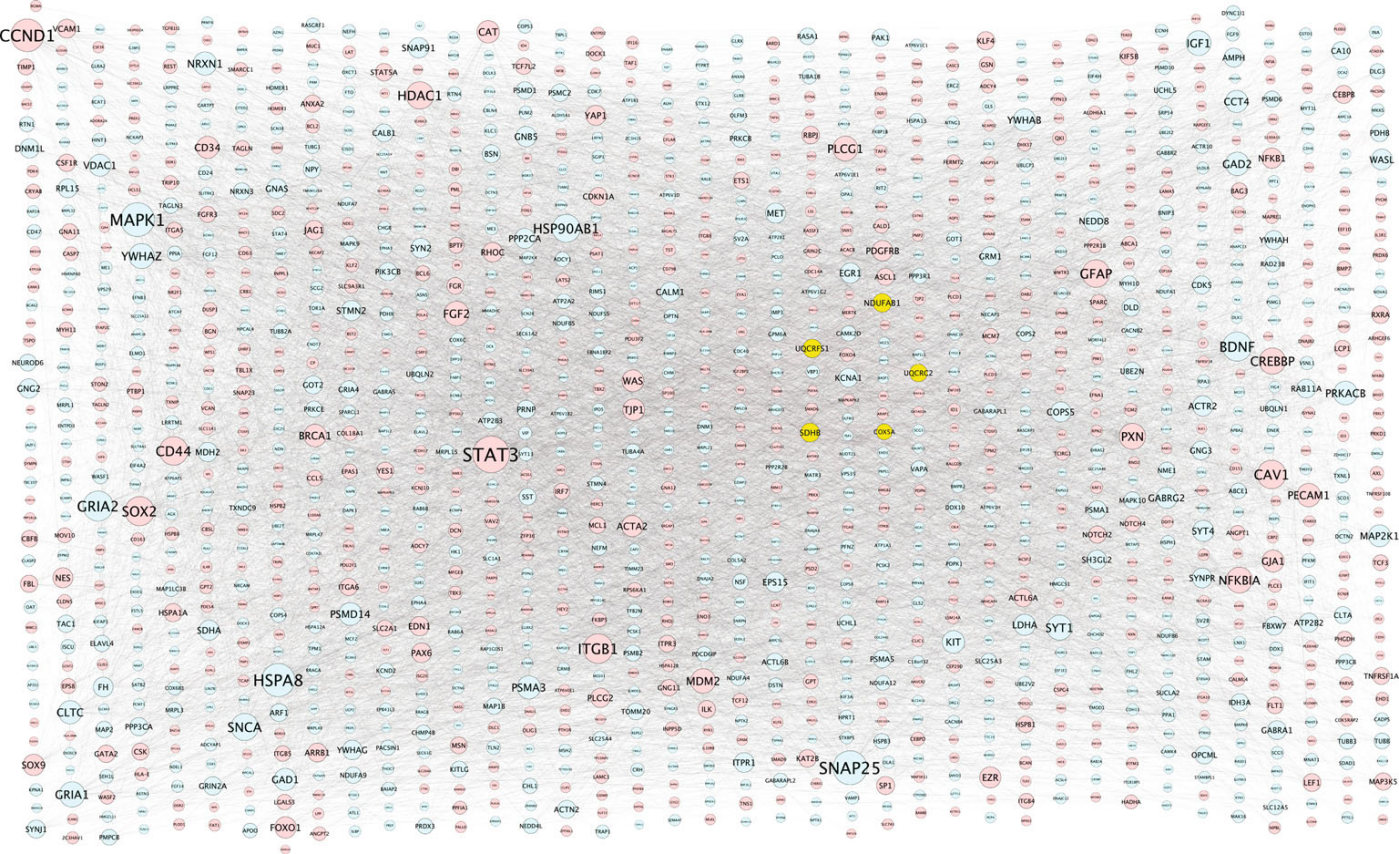
Global regulatory network. Global regulatory network based on brown and turquoise modules: yellow indicates the hub genes; larger size of node implies higher degree of gene connectivity; blue and red represent low and high expression, respectively.

**Figure 4:**
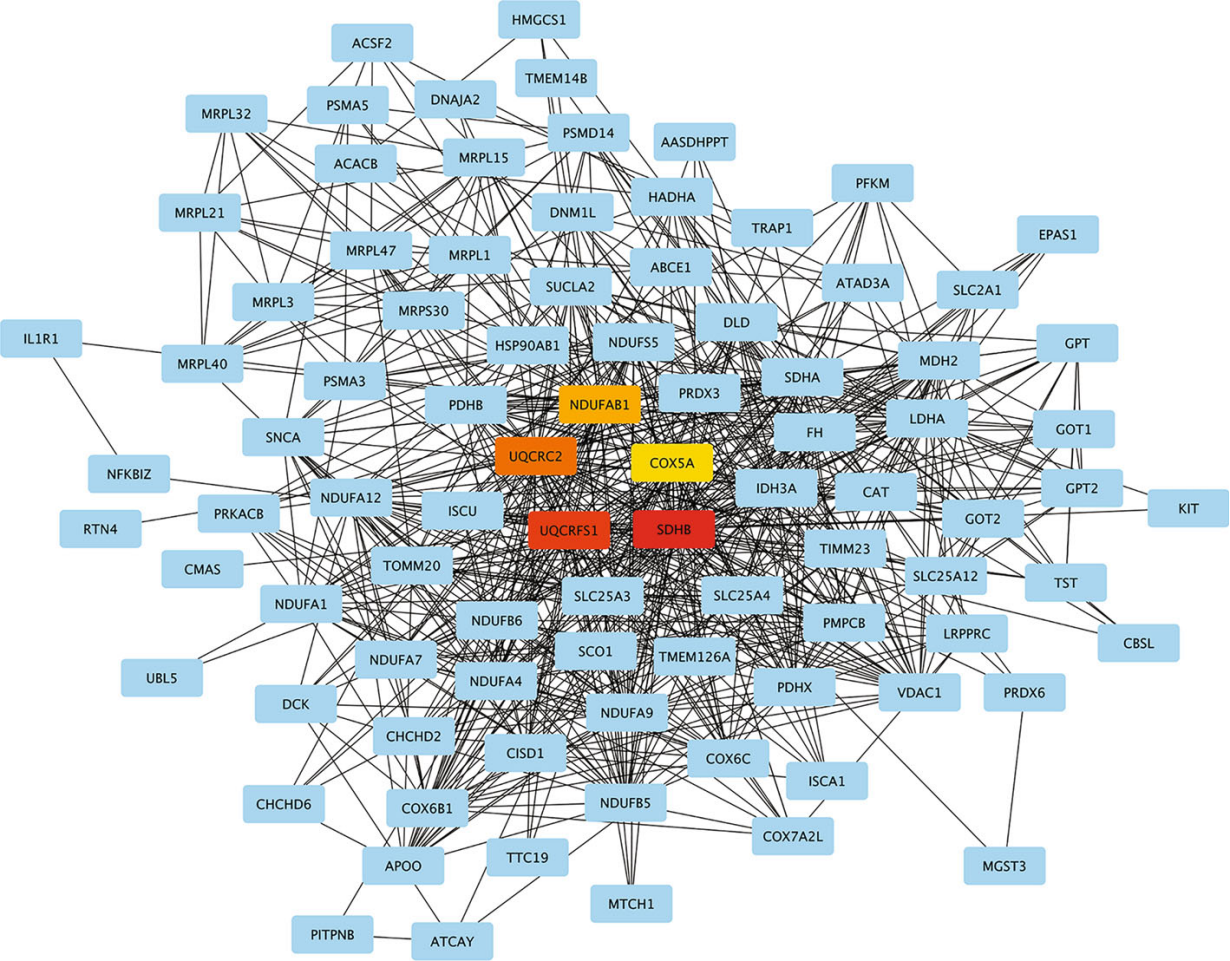
Hub gene identification. Five hub genes were determined by cytoHubba: the darker color indicates the higher rank.

**Figure 5:**
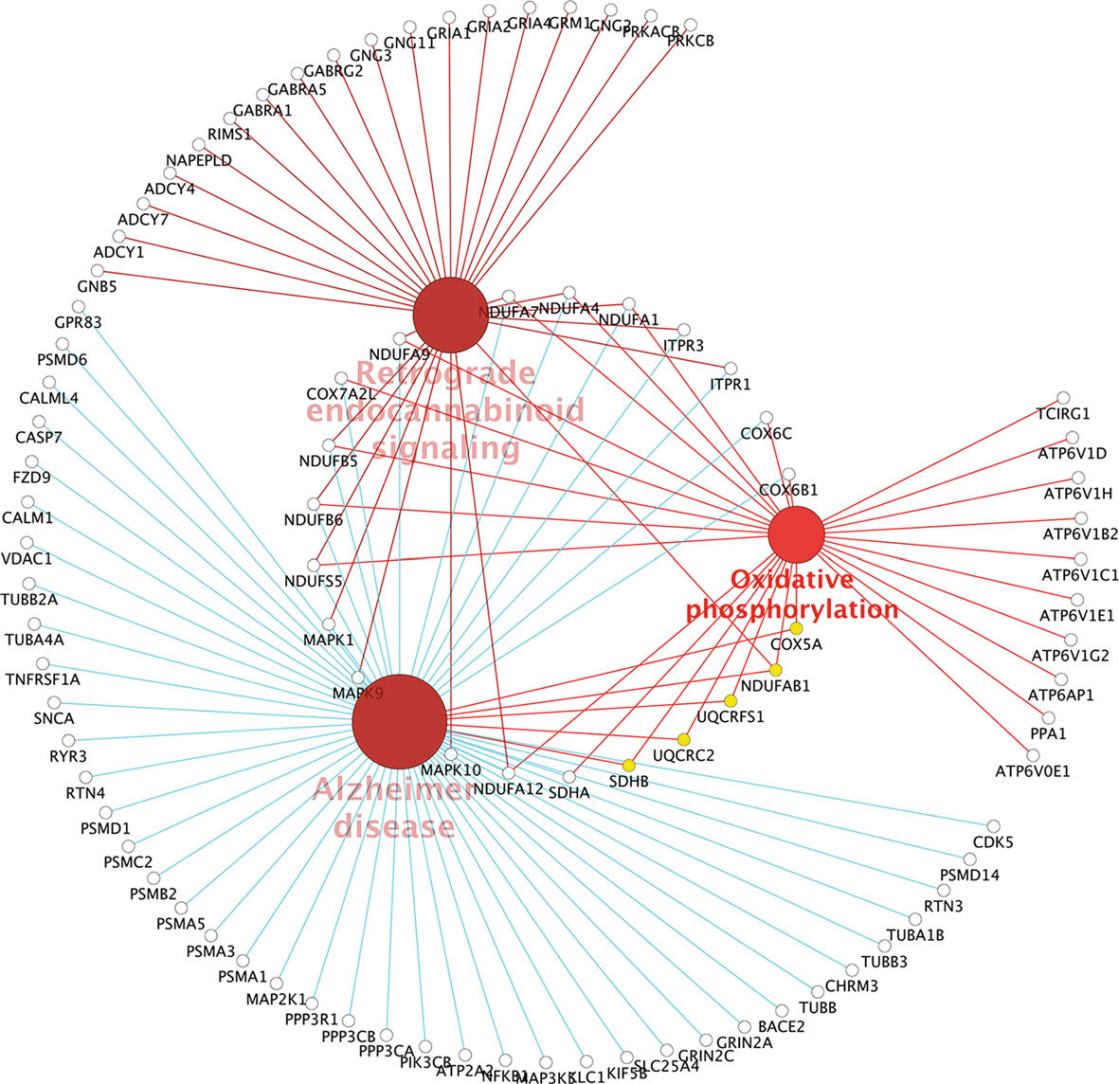
Pathway enrichment analysis of hub genes. Yellow nodes represent hub genes.

**Figure 6:**
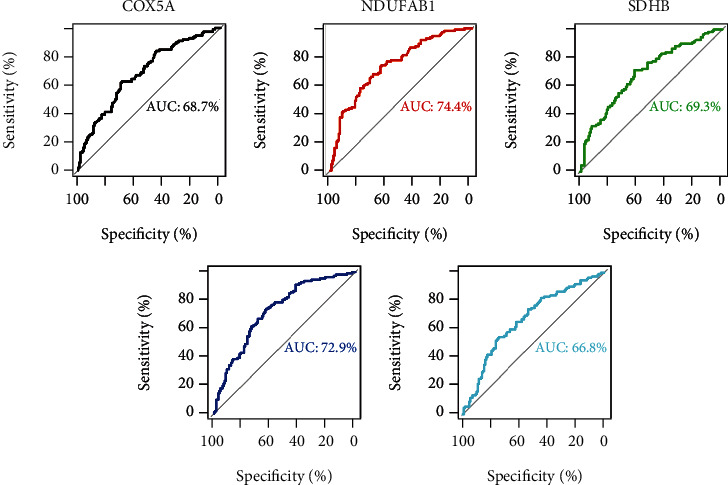
ROC curve analysis of hub genes. AUC: area under the curve; ROC: receiver operating characteristic.

On the basis of DEGs in two key modules (brown and turquoise), a global regulation network was constructed to provide PPI (Figure 3). As shown in Figure 4, five hub genes (COX5A, NDUFAB1, SDHB, UQCRC2, and UQCRFS1) strongly interacting with other DEGs were screened out through the cytoHubba function. Pathway enrichment analysis of hub genes (Figure 5) showed that COX5A, SDHB, UQCRC2, and UQCRFS1 were involved in oxidative phosphorylation; NDUFAB1 was implicated in oxidative phosphorylation and retrograde endocannabinoid signaling pathways. Analytic results of the ROC curve (Figure 6) presented a high discrimination ability of each hub gene in the onset of AD (COX5A: AUC = 68.7%; NDUFAB1: AUC = 74.4%; SDHB: AUC = 69.3%; UQCRC2: AUC = 72.9%; and UQCRFS1: AUC = 66.8%).

## 4. Discussion

Although AD has been investigated for decades, the inability to prevent and cure it remains a thorny issue at present. However, previous failed attempts at pharmaceutical development have not been in vain, as they highlight the critical need for gene-targeted therapies, which have made considerable breakthroughs in oncology and are increasingly being extended to central nervous system diseases [40–43]. With this background, genomic identification of novel biomarkers, therapeutic targets, and potential molecular underpinnings appears to be particularly important. Herein, we performed an integrative genomic analysis of five datasets involving 13,049 background genes, of which 2,163 DEGs were generated and then statistically analyzed to establish coexpression modules related to AD phenotype. Based on coexpressed genes of key modules, a global regulatory network was constructed to identify hub genes (i.e., COX5A, NDUFAB1, SDHB, UQCRC2, and UQCRFS1) as well as their mechanistic pathways underlying AD onset.

According to the results of WGCNA, the brown and turquoise modules were overwhelmingly correlated with AD, which were enriched in propanoate metabolism, glutamatergic synapse, long-term potentiation, oxidative phosphorylation, and hippo and retrograde endocannabinoid signaling pathways. Converging lines of evidence have linked AD to mitochondrial bioenergetic dysfunction, a process that predates the apparent appearance of plaques and persists throughout the pathological cascades of AD [44–47]. In terms of oxidative phosphorylation, it is the main energy source of mitochondria modulated by four respiratory multisubunit enzyme complexes, namely, complexes I-IV [48, 49]. During neurodegeneration of AD, A*β* and tau synergistically restrain the synthesis and function of mitochondrial respiratory complexes, giving rise to an impaired oxidative phosphorylation system [50]. The resultant decrease in oxidative phosphorylation promotes free radical production and adenosine triphosphate (ATP) depletion, leading to altered axonal transport, dysregulated organelle dynamics, neuronal loss, and apoptosis, as documented by *in vitro* experiments of AD at transcriptional and proteomic levels [51]. Additional evidence in support of this viewpoint comes from observations in Ts65Dn mice that oxidative phosphorylation disruption occurs in the early stages of cognitive decline in AD, an indicator specifically associated with basal forebrain cholinergic neurodegeneration that may propagate pathology within cortical memory and executive function circuits [52]. For retrograde endocannabinoid signaling, endocannabinoids play a profound neuroprotective role in the AD model by modulating the temporal dynamics of excitatory and inhibitory synaptic neurotransmitter release [53, 54]. For instance, endocannabinoids bind to and activate the cannabinoid 1 receptor (CB1R) to enhance synaptic plasticity and neurotransmitter delivery, which are essential for cognition, memory emotion, and motor functions [55, 56]. Administration of 2-arachidonoylglycerol (2-AG), a complete agonist of cannabinoid receptors, has been found to evoke the generation of anti-inflammatory mediators that protect neurons against A*β* insults in a concentration-dependent manner [57, 58]. This is confirmed by multiple experiments that inhibition of monoacylglycerol lipase (MAGL), a 2-AG hydrolase, mitigates A*β*-induced neurodegeneration and apoptosis via CB1R-dependent suppression of NF-*κ*B phosphorylation and ERK1/2 and cyclooxygenase-2 expression [59, 60]. Collectively, these data are supportive of our findings that oxidative phosphorylation and retrograde endocannabinoid signaling participate in the pathogenesis of AD.

More importantly, COX5A, NDUFAB1, SDHB, UQCRC2, and UQCRFS1 were selected as hub genes, and the downregulation of these genes is supposed to be vitally causative of AD. COX5A is the nuclear genome-encoded subunit Va of cytochrome c oxidase (COX or complex IV), which is indispensable in the regulation and assembly of mitochondrial respiratory chain holoenzyme [61, 62]. Previous studies have shown that mitochondrial COX activity in AD brains is dramatically decreased, an early alteration of bioenergetic dysfunction closely related to cognitive decline [63, 64]. Knockdown of COX5A suppresses the COX activity by reducing the affinity of residual enzymes to oxygen, thereby interfering with the ultracomplex pattern of the mitochondrial respiratory chain necessary for bioenergy metabolism [65]. Conversely, upregulation of COX5A can restore COX activity and intracellular ATP depletion to increase the synaptic excitability of entire dendrites through activation of brain-derived neurotrophic factor (BDNF), resulting in improvements in AD hippocampus-dependent spatial leaning, recognition, and memory deterioration [66]. In addition, there is convincing evidence that COX5A is also an important regulator of oxidative phosphorylation in brain senescence and degeneration [67], consistent with our pathway enrichment analysis for the involvement of COX5A in AD via oxidative phosphorylation.

NDUFAB1, a subunit of the NADH: ubiquinone oxidoreductase (NQR), encodes the initial enzyme of the mitochondrial respiratory chain (i.e., complex I) that catalyzes the transport of electrons from NADH to ubiquinone for ATP synthesis [68]. In Parkinson's disease [69] and AD [70], deficiency of complex I inhibits the activity of the electron transport chain, rendering it unable to cope with increased oxidative stress, leading to a pattern of programmed cell death termed as apoptosis. This is validated by administration of 1-methyl-4-phenylpyridinium ion (MPP1), an inhibitor of complex I, eliciting internucleosomal DNA degradation and inappropriate apoptotic activation in cultured PC12 and cerebellar granule cells [71]. SDHB, encoding the iron-sulfur subunit B of succinate dehydrogenase (SDH or complex II), carries electrons from flavin adenine dinucleotide (FADH) to coenzyme Q during succinate oxidation [72]. As demonstrated in iPSC-derived neural stem cells of presenilin 1 familial AD, downregulation of SDH contributes to an imbalance between mitochondrial fission, fusion, and morphology in AD hippocampus, a chronic fragmentary state of neurogenesis defects not only impelling self-renewal capacity of stem cells but also depleting stem cell pool [38, 73].

UQCRC2 and UQCRFS1 encode ubiquinol-cytochrome c reductase proteins, which are components of mitochondrial respiratory complex III. Approximately 30~70% reduction in complex III activity was observed in AD, possibly associated with excitotoxic cell death ascribed to insufficient control over glutamate release [74, 75]. More specifically, ATP depletion caused by complex III deficiency facilitates plasma membrane depolarization and Ca^2+^ overload, followed by the release of Ca^2+^-independent glutamate and overactivation of postsynaptic glutamate receptors, which are excitotoxic neurotransmitters contributing to cell death [76]. Moreover, APOE4 (1-272) fragment preferentially binds UQCRC2 to inhibit the enzymatic activity of complex III and promote the formation of neurofibrillary tangle-like structures, hence directly linking UQCRC2 to AD pathology [77]. Likewise, our findings pointed to a generalized downregulation of mitochondrial complexes I-IV in AD brains, which participated in oxidative phosphorylation and retrograde endocannabinoid signaling pathways. Analytic results of the ROC curve showed that five candidate genes had high diagnostic performance in AD prediction, supporting them as potential biomarkers or intervention targets for AD. These data highlight the involvement of the mitochondrial respiratory chain in AD onset, in agreement with previous biochemical studies using assays for electron transport chain activity [78].

Of note, several limitations of the present study should be taken into consideration. The batch effects during bioinformatics analysis cannot be completely removed by using the *removeBatchEffect* function, which may obscure the real biological signals if not adequately handled [79–81]. Moreover, we cannot rule out AD-related gene mutations in a small number of patients due to lack of such relevant information from the original study. Another limitation is the lack of detailed clinical and pathological data (e.g., Braak stage, plaque and tangle density, and Mini-Mental State Examination Score (MMSE)) to classify the severity of disease stages for each individual. To estimate the impact of the candidate markers proposed in this study on the progression of AD (not only limited to AD onset) and their clinical application value, prospective trials with large sample sizes and long-term follow-up, as well as detailed recording data of clinical and pathological disease severity, are encouraged to be carried out in the future. Additionally, basic experiments *in vivo* and *in vitro* are also recommended to verify the mechanistic pathways of COX5A, NDUFAB1, SDHB, UQCRC2, and UQCRFS1 in the pathogenesis of AD, which likely provide a substantial foundation for clinically targeted therapy.

## 5. Conclusion

In aggregate, integrative genomic analysis identified five molecular signatures associated with mitochondrial complexes, potentially participating in the pathogenesis of AD through oxidative phosphorylation (COX5A, NDUFAB1, SDHB, UQCRC2, and UQCRFS1) and retrograde endocannabinoid signaling (NDUFAB1) pathways. Our findings lend strong support for mitochondrial dysfunction underlying AD onset, which may elicit enthusiasm for the interaction of bioenergy restoration with AD therapy by targeting these molecular signatures.

## Data Availability

The datasets (GSE132903, GSE118553, GSE5281, GSE37264, and GSE36980) analyzed in this study are openly available in GEO (https://www.ncbi.nlm.nih.gov/geo/), and further inquiries may be directed to the corresponding author.

## Supplementary Materials

Supplementary 1 Supplementary Table 1: demographic data of participants.

Supplementary 2 Supplementary Table 2: differentially expressed genes in module-trait relationships. AD: Alzheimer's disease; GS: gene significance; MM: module membership.
