# Supplementary material for: Molecular Signatures of Mitochondrial Complexes Involved in Alzheimer's Disease via Oxidative Phosphorylation and Retrograde Endocannabinoid Signaling Pathways
Source: Oxid Med Cell Longev. 2022 Apr 5;2022:9565545. doi: 10.1155/2022/9565545 (PMC9006080; doi:10.1155/2022/9565545)
Supplement: Supplementary 1 — Supplementary Table 1: demographic data of participants. [file 9565545.f1.pdf]

Sample	Group	Age	Gender
GSM119641	Control	85	Male
GSM119642	Control	80	Male
GSM119643	Control	102	Female
GSM119644	Control	63	Male
GSM119645	Control	79	Male
GSM119646	Control	83	Male
GSM119647	Control	79	Male
GSM119648	Control	88	Female
GSM119650	Control	73	Female
GSM119651	Control	69	Male
GSM119652	Control	78	Male
GSM238809	AD	73	Female
GSM238810	AD	81	Male
GSM238811	AD	72	Male
GSM238812	AD	75	Male
GSM238813	AD	78	Male
GSM238815	AD	75	Male
GSM238816	AD	87	Male
GSM238817	AD	90	Female
GSM238818	AD	68	Male
GSM238819	AD	81	Female
GSM238820	AD	85	Female
GSM238821	AD	79	Male
GSM238822	AD	82	Female
GSM238823	AD	88	Male
GSM238824	AD	72	Male
GSM238825	AD	80	Female
GSM3332689	AD	84	Female
GSM3332693	Control	82	Female
GSM3332697	Control	51	Female
GSM3332703	Control	92	Female
GSM3332721	AD	66	Male
GSM3332729	AD	96	Female
GSM3332735	AD	72	Female
GSM3332737	AD	93	Female
GSM3332742	Control	52	Female
GSM3332753	Control	87	Female
GSM3332755	Control	81	Male
GSM3332762	Control	77	Male

GSM3332773	AD	88	Male
GSM3332789	AD	90	Female
GSM3332790	AD	97	Male
GSM3332794	AD	89	Male
GSM3332800	Control	91	Female
GSM3332805	AD	88	Male
GSM3332810	AD	85	Female
GSM3332811	Control	95	Male
GSM3332814	AD	86	Male
GSM3332819	AD	85	Female
GSM3332821	Control	78	Male
GSM3332824	AD	83	Female
GSM3332829	Control	73	Male
GSM3332835	Control	86	Male
GSM3332841	AD	80	Male
GSM3332843	Control	40	Male
GSM3332854	AD	71	Male
GSM3332870	AD	82	Female
GSM3332871	AD	69	Male
GSM3332876	AD	75	Male
GSM3332883	AD	96	Female
GSM3332885	Control	80	Male
GSM3332891	AD	72	Male
GSM3332893	AD	81	Female
GSM3332899	AD	105	Male
GSM3332902	AD	67	Male
GSM3332906	AD	92	Female
GSM3332909	AD	79	Female
GSM3332911	AD	87	Female
GSM3332925	AD	84	Female
GSM3332927	AD	81	Male
GSM3332932	AD	71	Female
GSM3332941	AD	80	Female
GSM3332944	AD	79	Female
GSM3332946	AD	76	Male
GSM3332949	Control	78	Male
GSM3332954	AD	66	Male
GSM3332960	Control	41	Male
GSM3332977	AD	79	Male
GSM3332984	AD	90	Female

GSM3332990	Control	51	Male
GSM3332993	AD	95	Female
GSM3332996	Control	67	Male
GSM3332999	AD	83	Female
GSM3333004	AD	68	Male
GSM3333016	Control	43	Female
GSM3333022	AD	89	Male
GSM3333032	AD	90	Male
GSM3333038	Control	66	Male
GSM3333039	AD	88	Female
GSM3333044	AD	63	Female
GSM3333050	AD	83	Female
GSM3333053	Control	88	Female
GSM3333070	AD	98	Female
GSM3333079	Control	80	Female
GSM3333082	Control	65	Male
GSM3333088	Control	73	Female
GSM3895951	Control	90	Female
GSM3895952	Control	82	Male
GSM3895953	Control	88	Female
GSM3895954	Control	92	Female
GSM3895955	Control	91	Male
GSM3895956	Control	87	Female
GSM3895957	Control	86	Male
GSM3895958	Control	78	Male
GSM3895959	Control	87	Male
GSM3895960	Control	79	Male
GSM3895961	Control	77	Female
GSM3895962	Control	77	Male
GSM3895963	Control	88	Female
GSM3895964	Control	85	Male
GSM3895965	Control	95	Female
GSM3895966	Control	102	Female
GSM3895967	Control	89	Male
GSM3895968	Control	70	Male
GSM3895969	Control	82	Female
GSM3895970	Control	73	Female
GSM3895971	Control	90	Male
GSM3895972	Control	94	Male
GSM3895973	Control	96	Female

GSM3895974	Control	85	Female
GSM3895975	Control	84	Male
GSM3895976	Control	83	Male
GSM3895977	Control	90	Female
GSM3895978	Control	87	Female
GSM3895979	Control	85	Male
GSM3895980	Control	83	Female
GSM3895981	Control	84	Male
GSM3895982	Control	88	Male
GSM3895983	Control	98	Female
GSM3895984	Control	85	Male
GSM3895985	Control	86	Female
GSM3895986	Control	87	Female
GSM3895987	Control	89	Male
GSM3895988	Control	92	Male
GSM3895989	Control	78	Female
GSM3895990	Control	77	Female
GSM3895991	Control	91	Male
GSM3895992	Control	100	Male
GSM3895993	Control	82	Male
GSM3895994	Control	87	Female
GSM3895995	Control	73	Male
GSM3895996	Control	75	Male
GSM3895997	Control	82	Male
GSM3895998	Control	90	Female
GSM3895999	Control	96	Female
GSM3896000	Control	84	Female
GSM3896001	Control	80	Male
GSM3896002	Control	86	Male
GSM3896003	Control	91	Female
GSM3896004	Control	91	Female
GSM3896005	Control	94	Female
GSM3896006	Control	87	Male
GSM3896007	Control	75	Female
GSM3896008	Control	74	Male
GSM3896009	Control	76	Male
GSM3896010	Control	71	Male
GSM3896011	Control	87	Male
GSM3896012	Control	90	Male
GSM3896013	Control	80	Male

GSM3896014	Control	84	Male
GSM3896015	Control	80	Male
GSM3896016	Control	89	Male
GSM3896017	Control	86	Female
GSM3896018	Control	80	Female
GSM3896019	Control	92	Male
GSM3896020	Control	83	Female
GSM3896021	Control	86	Female
GSM3896022	Control	91	Female
GSM3896023	Control	95	Female
GSM3896024	Control	95	Female
GSM3896025	Control	82	Female
GSM3896026	Control	85	Female
GSM3896027	Control	87	Female
GSM3896028	Control	95	Male
GSM3896029	Control	85	Female
GSM3896030	Control	91	Female
GSM3896031	Control	89	Female
GSM3896032	AD	80	Male
GSM3896033	AD	87	Female
GSM3896034	AD	92	Female
GSM3896035	AD	77	Female
GSM3896036	AD	84	Female
GSM3896037	AD	91	Female
GSM3896038	AD	87	Female
GSM3896039	AD	97	Female
GSM3896040	AD	87	Female
GSM3896041	AD	78	Male
GSM3896042	AD	76	Male
GSM3896043	AD	81	Male
GSM3896044	AD	80	Male
GSM3896045	AD	86	Female
GSM3896046	AD	81	Female
GSM3896047	AD	79	Male
GSM3896048	AD	91	Female
GSM3896049	AD	91	Female
GSM3896050	AD	89	Female
GSM3896051	AD	82	Female
GSM3896052	AD	92	Female
GSM3896053	AD	86	Male

GSM3896054	AD	82	Female
GSM3896055	AD	86	Female
GSM3896056	AD	80	Male
GSM3896057	AD	87	Female
GSM3896058	AD	92	Male
GSM3896059	AD	90	Female
GSM3896060	AD	88	Female
GSM3896061	AD	90	Male
GSM3896062	AD	90	Male
GSM3896063	AD	72	Male
GSM3896064	AD	87	Male
GSM3896065	AD	75	Male
GSM3896066	AD	86	Female
GSM3896067	AD	95	Female
GSM3896068	AD	95	Male
GSM3896069	AD	88	Female
GSM3896070	AD	87	Male
GSM3896071	AD	81	Female
GSM3896072	AD	83	Male
GSM3896073	AD	85	Female
GSM3896074	AD	95	Female
GSM3896075	AD	81	Male
GSM3896076	AD	83	Male
GSM3896077	AD	85	Male
GSM3896078	AD	85	Female
GSM3896079	AD	94	Male
GSM3896080	AD	97	Male
GSM3896081	AD	82	Female
GSM3896082	AD	91	Male
GSM3896083	AD	92	Male
GSM3896084	AD	70	Male
GSM3896085	AD	84	Male
GSM3896086	AD	86	Male
GSM3896087	AD	95	Female
GSM3896088	AD	88	Male
GSM3896089	AD	79	Male
GSM3896090	AD	87	Male
GSM3896091	AD	73	Female
GSM3896092	AD	90	Female
GSM3896093	AD	83	Male

GSM3896094	AD	85	Female
GSM3896095	AD	74	Male
GSM3896096	AD	71	Male
GSM3896097	AD	78	Female
GSM3896098	AD	82	Male
GSM3896099	AD	85	Male
GSM3896100	AD	96	Female
GSM3896101	AD	70	Female
GSM3896102	AD	78	Female
GSM3896103	AD	77	Female
GSM3896104	AD	87	Female
GSM3896105	AD	84	Male
GSM3896106	AD	98	Male
GSM3896107	AD	75	Male
GSM3896108	AD	76	Male
GSM3896109	AD	94	Female
GSM3896110	AD	84	Male
GSM3896111	AD	75	Female
GSM3896112	Control	75	Male
GSM3896113	Control	92	Female
GSM3896114	Control	81	Female
GSM3896115	Control	77	Female
GSM3896116	AD	88	Male
GSM3896117	AD	87	Female
GSM3896118	AD	77	Female
GSM3896119	AD	93	Male
GSM3896120	AD	97	Female
GSM3896121	AD	89	Male
GSM3896122	AD	88	Male
GSM3896123	AD	73	Male
GSM3896124	AD	91	Female
GSM3896125	AD	91	Female
GSM3896126	Control	78	Female
GSM3896127	AD	89	Male
GSM3896128	AD	78	Female
GSM3896129	AD	90	Female
GSM3896130	AD	85	Male
GSM3896131	AD	85	Female
GSM3896132	AD	82	Male
GSM3896133	AD	82	Male

GSM3896134	Control	72	Male
GSM3896135	Control	82	Female
GSM3896136	Control	81	Female
GSM3896137	Control	81	Male
GSM3896138	Control	79	Male
GSM3896139	Control	91	Male
GSM3896140	Control	81	Male
GSM3896141	Control	70	Female
GSM3896142	Control	76	Male
GSM3896143	Control	90	Male
GSM3896144	Control	83	Male
GSM3896145	Control	83	Male
GSM907825	AD	84	Male
GSM907826	AD	88	Female
GSM907827	AD	95	Female
GSM907828	AD	88	Male
GSM907829	AD	91	Female
GSM907830	AD	95	Female
GSM907831	AD	95	Female
GSM907832	AD	89	Male
GSM907833	AD	83	Male
GSM907834	AD	90	Male
GSM907835	Control	77	Female
GSM907836	Control	87	Female
GSM907837	Control	80	Female
GSM907838	Control	84	Female
GSM907839	Control	77	Male
GSM907840	Control	55	Male
GSM907841	Control	72	Female
GSM907842	Control	89	Male
GSM907843	Control	82	Male
GSM907844	Control	78	Female
GSM907845	Control	83	Male
GSM907846	Control	80	Male
GSM907847	Control	75	Female
GSM907848	Control	89	Male
GSM907849	Control	65	Female
GSM907850	Control	71	Female
GSM907851	Control	74	Male
GSM907852	Control	86	Female

GSM907853	Control	54	Female
GSM915110	Control	85	Male
GSM915111	Control	77	Male
GSM915112	AD	83	Male
GSM915113	AD	67	Male
GSM915114	AD	88	Female
GSM915115	AD	66	Male
GSM915116	Control	79	Female
GSM915117	Control	87	Male
GSM915118	Control	66	Male
GSM915119	AD	69	Male
GSM915120	Control	81	Female
GSM915121	AD	63	Female
GSM915122	AD	78	Male
GSM915123	Control	78	Male
GSM915124	AD	67	Female
GSM915125	Control	88	Female
