# Supplementary material for: Molecular Signatures of Mitochondrial Complexes Involved in Alzheimer's Disease via Oxidative Phosphorylation and Retrograde Endocannabinoid Signaling Pathways
Source: Oxid Med Cell Longev. 2022 Apr 5;2022:9565545. doi: 10.1155/2022/9565545 (PMC9006080; doi:10.1155/2022/9565545)
Supplement: Supplementary 2 — Supplementary Table 2: differentially expressed genes in module-trait relationships. AD: Alzheimer's disease; GS: gene significance; MM: module membership. [file 9565545.f2.pdf]

Gene.Symbol	moduleColor	GS.AD	p.GS.AD	MMyellow	p.MMyellow
ABLIM2	blue	-0.286364071	5.24E-06	-0.460832763	2.77E-14
ACE2	blue	-0.212702365	0.000806028	-0.163157111	0.01053017
ACHE	blue	-0.260621608	3.63E-05	-0.413619043	1.52E-11
ACO2	blue	-0.188032699	0.003131513	-0.541341308	4.70E-20
ACTB	blue	-0.342457816	3.80E-08	-0.643147142	5.37E-30
ADAM32	blue	-0.188017201	0.00313403	-0.243282823	0.00011984
ALDOA	blue	-0.334376774	8.22E-08	-0.616683286	4.63E-27
ANKRD12	blue	0.145114306	0.023097348	0.257775131	4.44E-05
AP2M1	blue	-0.258058544	4.35E-05	-0.478184995	2.11E-15
AP3B2	blue	-0.328951992	1.36E-07	-0.529980099	3.81E-19
APBB3	blue	-0.277199271	1.07E-05	-0.260284062	3.71E-05
APEX1	blue	-0.266219956	2.42E-05	-0.676450015	3.96E-34
APLP1	blue	-0.316918532	4.05E-07	-0.412666109	1.71E-11
APLP2	blue	-0.345634892	2.78E-08	-0.573683157	7.66E-23
ARF3	blue	-0.3572778	8.68E-09	-0.684361388	3.43E-35
ARF5	blue	-0.417530578	9.37E-12	-0.552977012	5.05E-21
ARHGDIG	blue	-0.321549132	2.68E-07	-0.543388257	3.19E-20
ARHGEF11	blue	-0.282594237	7.04E-06	-0.264126189	2.82E-05
ARSG	blue	-0.255957871	5.04E-05	-0.235230663	0.000202897
ATP1A3	blue	-0.374651171	1.39E-09	-0.425574132	3.38E-12
ATP6V0D1	blue	-0.268112875	2.11E-05	-0.515224676	5.16E-18
ATP6V1F	blue	-0.32254584	2.45E-07	-0.39791858	1.01E-10
ATXN10	blue	-0.407322835	3.29E-11	-0.44140563	4.18E-13
BSG	blue	-0.221498337	0.000478071	-0.309647315	7.65E-07
CA11	blue	-0.196114304	0.002042521	-0.382004735	6.21E-10
CABP1	blue	-0.272209323	1.55E-05	-0.308299118	8.59E-07
CACNG3	blue	-0.388633733	2.95E-10	-0.406697103	3.55E-11
CADM3	blue	-0.228030404	0.000320043	-0.351392083	1.57E-08
CALY	blue	-0.321236331	2.76E-07	-0.62841079	2.51E-28
CAMK1G	blue	-0.348253754	2.15E-08	-0.584925426	6.94E-24
CAPNS1	blue	-0.345414006	2.84E-08	-0.593124159	1.14E-24
CARD8	blue	0.314804168	4.89E-07	0.344914292	2.99E-08
CEND1	blue	-0.257202113	4.62E-05	-0.397147937	1.10E-10
CHCHD10	blue	-0.253632669	5.93E-05	-0.418827597	7.97E-12
CHRM1	blue	-0.292095823	3.31E-06	-0.528600342	4.89E-19
CHST1	blue	-0.257118278	4.65E-05	-0.485847779	6.44E-16
CITED1	blue	-0.273131294	1.45E-05	-0.284731886	5.95E-06
CLPTM1	blue	-0.220658571	0.000502965	-0.363081032	4.77E-09
CLSTN1	blue	-0.409870049	2.42E-11	-0.52018432	2.18E-18

CNTNAP1	blue	-0.130415544	0.041389623	-0.252993002	6.20E-05
CORO1A	blue	-0.211268534	0.000875964	-0.423589365	4.36E-12
CORO6	blue	-0.249629521	7.81E-05	-0.188971754	0.002982356
CPLX1	blue	-0.400372353	7.56E-11	-0.397768026	1.03E-10
CPLX2	blue	-0.359924883	6.61E-09	-0.420054496	6.82E-12
CPNE9	blue	-0.214169275	0.000739837	-0.381440302	6.62E-10
CRMP1	blue	-0.359164787	7.15E-09	-0.62490099	6.09E-28
DDT	blue	-0.327863011	1.51E-07	-0.480511445	1.47E-15
DDX24	blue	-0.365200345	3.82E-09	-0.688132848	1.04E-35
DIRAS1	blue	-0.29912642	1.86E-06	-0.39293792	1.80E-10
DNM1	blue	-0.261434272	3.42E-05	-0.25971619	3.87E-05
DOC2A	blue	-0.271986154	1.58E-05	-0.569863645	1.70E-22
DPP6	blue	-0.44313874	3.31E-13	-0.56400597	5.63E-22
DYNC1H1	blue	-0.292972448	3.09E-06	-0.512149273	8.75E-18
ENO2	blue	-0.424951337	3.66E-12	-0.562998585	6.90E-22
ENTPD6	blue	-0.280262496	8.43E-06	-0.508745229	1.56E-17
EPB41L1	blue	-0.238832245	0.000160678	-0.466061574	1.29E-14
FANCE	blue	0.307236923	9.41E-07	0.563986131	5.65E-22
FBXL16	blue	-0.244533454	0.000110251	-0.465678557	1.37E-14
FKBP1A	blue	-0.356987209	8.94E-09	-0.655464995	1.83E-31
GABRD	blue	-0.331912428	1.04E-07	-0.256854453	4.74E-05
GAS7	blue	-0.421541747	5.65E-12	-0.504467645	3.19E-17
GPI	blue	-0.362186395	5.23E-09	-0.565420157	4.22E-22
GRINA	blue	-0.342649857	3.73E-08	-0.503621963	3.67E-17
HMGA1	blue	-0.329741807	1.27E-07	-0.403675789	5.11E-11
HPCA	blue	-0.358243039	7.86E-09	-0.453602185	7.76E-14
HUWE1	blue	-0.352898294	1.35E-08	-0.312752259	5.85E-07
IDH3G	blue	-0.223856568	0.00041414	-0.413132268	1.62E-11
IFT74	blue	0.252245533	6.53E-05	0.456518076	5.14E-14
IGSF21	blue	-0.236323102	0.000189107	-0.018041425	0.778732132
INO80D	blue	0.280036696	8.58E-06	0.520822177	1.95E-18
IRF2BP2	blue	0.223706096	0.00041797	0.30213939	1.45E-06
KCNQ2	blue	-0.195667507	0.00209226	-0.125033026	0.050613138
KCTD13	blue	-0.359327777	7.03E-09	-0.422888651	4.76E-12
KLHDC9	blue	-0.319858265	3.12E-07	-0.51157222	9.66E-18
KLHL35	blue	-0.412497435	1.75E-11	-0.251262687	6.99E-05
L1CAM	blue	-0.341205243	4.28E-08	-0.567719781	2.64E-22
LDOC1	blue	-0.238656034	0.000162536	-0.696456314	6.97E-37
LINGO1	blue	-0.383631771	5.18E-10	-0.472621184	4.89E-15
LY6E	blue	-0.272957782	1.47E-05	-0.461588086	2.48E-14

MAP1A	blue	-0.245606416	0.000102603	-0.407269669	3.31E-11
MAP1S	blue	-0.16630009	0.009110468	-0.37819067	9.47E-10
MAPK8IP2	blue	-0.320701094	2.89E-07	-0.345546077	2.81E-08
MAPRE3	blue	-0.206316765	0.001162737	-0.249943737	7.65E-05
MGRN1	blue	-0.323505634	2.25E-07	-0.453657405	7.70E-14
MICAL2	blue	-0.404698515	4.52E-11	-0.51876256	2.80E-18
MLF2	blue	-0.309590517	7.69E-07	-0.469920881	7.32E-15
MPV17	blue	-0.149747378	0.019018456	-0.362545077	5.04E-09
MRPL2	blue	-0.352197723	1.45E-08	-0.453861651	7.48E-14
NCDN	blue	-0.252401469	6.46E-05	-0.387253109	3.45E-10
NDUFV1	blue	-0.289042253	4.23E-06	-0.625124327	5.75E-28
NECAB2	blue	-0.289247777	4.16E-06	-0.302834403	1.37E-06
NEU1	blue	-0.356717625	9.19E-09	-0.476916767	2.56E-15
NPDC1	blue	-0.306181115	1.03E-06	-0.557239933	2.19E-21
NPM2	blue	-0.239039779	0.000158515	-0.418145066	8.68E-12
NPTXR	blue	-0.39191945	2.03E-10	-0.545588036	2.10E-20
NRGN	blue	-0.227802423	0.00032462	-0.448852831	1.51E-13
NSUN6	blue	0.0197885	0.757941428	0.332860937	9.48E-08
OGDHL	blue	-0.304245242	1.21E-06	-0.407689276	3.15E-11
OLFM2	blue	-0.261277056	3.46E-05	-0.301746479	1.50E-06
P4HTM	blue	-0.219510499	0.000538943	-0.252687458	6.33E-05
PAK2	blue	0.346260011	2.62E-08	0.589967469	2.29E-24
PCDH7	blue	-0.225671297	0.000370449	-0.487919232	4.65E-16
PCSK1N	blue	-0.271564461	1.63E-05	-0.508942659	1.51E-17
PDE1B	blue	-0.286582397	5.15E-06	-0.407236473	3.33E-11
PEX11B	blue	-0.435426084	9.33E-13	-0.742298033	3.84E-44
PFDN1	blue	-0.247779216	8.86E-05	-0.563335795	6.45E-22
PIN1	blue	-0.294387436	2.75E-06	-0.405520943	4.09E-11
PKNOX2	blue	-0.283061945	6.78E-06	-0.425103509	3.59E-12
PNCK	blue	-0.332346117	9.95E-08	-0.22019802	0.000517122
PNKD	blue	-0.320422234	2.97E-07	-0.4712783	5.98E-15
PNMA6A	blue	-0.252658035	6.35E-05	-0.482732608	1.05E-15
PPME1	blue	-0.344303279	3.17E-08	-0.336402879	6.79E-08
PPP1R11	blue	-0.218741832	0.000564349	-0.517483797	3.49E-18
PPP2R1A	blue	-0.292424913	3.23E-06	-0.230393788	0.000276005
PPP2R2A	blue	-0.249903992	7.67E-05	-0.391047473	2.24E-10
PRDX2	blue	-0.226447702	0.000353098	-0.697106143	5.62E-37
PRKCG	blue	-0.33861914	5.49E-08	-0.562073641	8.32E-22
PRTN3	blue	-0.209710982	0.000958241	-0.048763671	0.447361636
PTK2B	blue	-0.311731108	6.39E-07	-0.384554301	4.67E-10

PTPRN	blue	-0.403734019	5.07E-11	-0.583940588	8.60E-24
RGS6	blue	-0.244433506	0.00011099	-0.251003623	7.11E-05
RHBDD2	blue	-0.271516454	1.64E-05	-0.217796345	0.000597117
RNF19B	blue	-0.225814126	0.000367199	-0.597116483	4.62E-25
RNMT	blue	-0.33628726	6.86E-08	-0.530476132	3.49E-19
ROCK1	blue	0.259267955	3.99E-05	0.230579867	0.000272789
RPS6KL1	blue	-0.297012243	2.22E-06	-0.202073678	0.001474563
RUNDC3A	blue	-0.335457803	7.42E-08	-0.415859234	1.15E-11
RWDD2B	blue	-0.257257004	4.60E-05	-0.253217085	6.10E-05
SCAMP5	blue	-0.318432971	3.54E-07	-0.490118753	3.29E-16
SCN1B	blue	-0.268424395	2.06E-05	-0.471247657	6.00E-15
SEZ6L	blue	-0.325717466	1.84E-07	-0.352651188	1.39E-08
SLC17A7	blue	-0.296305956	2.35E-06	-0.261026308	3.52E-05
SLC23A1	blue	-0.243768541	0.000116027	-0.047274526	0.461371039
SLC6A7	blue	-0.199982554	0.001654889	-0.132106509	0.03880169
SLTM	blue	0.344960512	2.97E-08	0.283787883	6.41E-06
SNCB	blue	-0.284206386	6.20E-06	-0.340093969	4.77E-08
SNCG	blue	-0.170800675	0.00737324	-0.115718861	0.070590543
STS	blue	-0.323068449	2.34E-07	-0.325081545	1.95E-07
STX1A	blue	-0.307996011	8.82E-07	-0.626135666	4.46E-28
STXBP1	blue	-0.389902578	2.55E-10	-0.818874647	1.60E-60
SUPT4H1	blue	-0.298195397	2.01E-06	-0.46850194	9.03E-15
SYN1	blue	-0.324298767	2.09E-07	-0.477307681	2.41E-15
SYNGR3	blue	-0.348050508	2.19E-08	-0.656052907	1.55E-31
SYP	blue	-0.381289225	6.73E-10	-0.540533757	5.46E-20
SYT3	blue	-0.278179075	9.89E-06	-0.250791003	7.22E-05
SYT5	blue	-0.277909578	1.01E-05	-0.280019545	8.59E-06
TERF2	blue	-0.245090002	0.00010622	-0.658236893	8.37E-32
THY1	blue	-0.318767144	3.44E-07	-0.250065243	7.58E-05
TM7SF2	blue	-0.35297692	1.34E-08	-0.438205742	6.44E-13
TMEM151A	blue	-0.205291179	0.001231991	-0.174159416	0.006275985
TPI1	blue	-0.328324303	1.45E-07	-0.462061455	2.32E-14
TRIML1	blue	-0.216083673	0.000660995	-0.095889544	0.134475525
TUBG2	blue	-0.365491255	3.70E-09	-0.305009465	1.14E-06
UBA1	blue	-0.208364686	0.001035028	-0.380780165	7.12E-10
USP11	blue	-0.423461822	4.43E-12	-0.486892434	5.47E-16
USP5	blue	-0.266780279	2.32E-05	-0.444089754	2.90E-13
VAMP2	blue	-0.377006111	1.08E-09	-0.659443359	5.94E-32
VSTM2L	blue	-0.298009705	2.04E-06	-0.392843173	1.82E-10
YJEFN3	blue	-0.223808079	0.000415371	-0.489897287	3.40E-16

YWHAE	blue	-0.339979808	4.82E-08	-0.547193558	1.55E-20
ZHX2	blue	0.300427796	1.67E-06	0.583072119	1.04E-23
ZNF532	blue	0.297801975	2.08E-06	0.368101598	2.81E-09
AASS	brown	0.19363704	0.002332506	0.168537601	0.008205958
ABCA1	brown	0.341873259	4.02E-08	0.473454394	4.31E-15
ACACB	brown	0.415957636	1.14E-11	0.702306455	9.87E-38
ACOT11	brown	0.299842563	1.76E-06	0.369412719	2.45E-09
ACSBG1	brown	0.306838311	9.74E-07	0.43598833	8.66E-13
ACSS1	brown	0.356703032	9.20E-09	0.574247364	6.81E-23
ACSS3	brown	0.414324167	1.40E-11	0.370270592	2.23E-09
ACTL6A	brown	0.461608677	2.47E-14	0.47466309	3.60E-15
ADD3	brown	0.200921241	0.001571584	0.419169971	7.63E-12
AHNAK	brown	0.429139651	2.13E-12	0.625615021	5.09E-28
AKR1C3	brown	0.341755176	4.06E-08	0.509779924	1.31E-17
ALDH6A1	brown	0.172738574	0.006720956	0.329478512	1.30E-07
ANTXR1	brown	0.415691405	1.18E-11	0.451749107	1.01E-13
ANTXR2	brown	0.473646701	4.19E-15	0.358351173	7.77E-09
ARHGEF6	brown	0.409145279	2.64E-11	0.479976316	1.60E-15
ASCL1	brown	0.47745084	2.36E-15	0.480703985	1.43E-15
AXL	brown	0.342080927	3.94E-08	0.601788837	1.58E-25
BAMBI	brown	0.295244125	2.56E-06	0.313508941	5.47E-07
BBOX1	brown	0.326254702	1.75E-07	0.286414829	5.21E-06
BCL2	brown	0.274832822	1.28E-05	0.469679225	7.58E-15
BDH2	brown	0.361842228	5.42E-09	0.265705785	2.51E-05
BMP7	brown	0.169185997	0.00795922	0.406469068	3.65E-11
CAT	brown	0.264236281	2.80E-05	0.20274625	0.001420507
CBFB	brown	0.284804638	5.92E-06	0.33371153	8.75E-08
CDC14A	brown	0.361821652	5.43E-09	0.238795752	0.000161061
CDK2AP1	brown	0.474312839	3.79E-15	0.434495241	1.06E-12
CEBPB	brown	0.335419114	7.45E-08	0.349190104	1.96E-08
CGNL1	brown	0.405087016	4.31E-11	0.579829545	2.09E-23
CMBL	brown	0.356148291	9.74E-09	0.230112672	0.00028093
CMTM4	brown	0.333687423	8.77E-08	0.377237497	1.05E-09
COLEC12	brown	0.410039903	2.37E-11	0.128859284	0.043898303
CYBRD1	brown	0.229376426	0.00029422	0.200117949	0.001642629
DBI	brown	0.383372191	5.34E-10	0.396628829	1.17E-10
DDIT4L	brown	0.39956274	8.32E-11	0.555297155	3.21E-21
DNALI1	brown	0.470664181	6.55E-15	0.618028979	3.34E-27
DPY19L4	brown	0.272683171	1.50E-05	0.174828403	0.00607575
EFEMP2	brown	0.403247001	5.37E-11	0.67041868	2.43E-33

EFHC2	brown	0.268781702	2.01E-05	0.257532391	4.51E-05
ELF1	brown	0.473625284	4.20E-15	0.45202856	9.68E-14
EMX2	brown	0.339481949	5.06E-08	0.406587177	3.60E-11
EPS8	brown	0.358253992	7.85E-09	0.331327858	1.09E-07
EYA1	brown	0.220648529	0.00050327	0.237403832	0.00017633
EYA2	brown	0.273579689	1.40E-05	0.389815419	2.58E-10
EZR	brown	0.4781773	2.11E-15	0.618735554	2.81E-27
FAM107A	brown	0.360432392	6.28E-09	0.40090394	7.10E-11
FAM167A	brown	0.342820232	3.66E-08	0.440282417	4.87E-13
FARP1	brown	0.307113341	9.51E-07	0.595948461	6.01E-25
FAT1	brown	0.308129847	8.72E-07	0.518938608	2.71E-18
FERMT2	brown	0.23639547	0.000188225	0.409468698	2.54E-11
FGF2	brown	0.267658273	2.18E-05	0.356797154	9.11E-09
FGFR3	brown	0.172473098	0.006807154	0.403071751	5.49E-11
FOXO1	brown	0.36401055	4.33E-09	0.516529168	4.12E-18
GATM	brown	0.275438922	1.22E-05	0.078948778	0.218193096
GJA1	brown	0.23624904	0.000190013	0.259865898	3.83E-05
GLIS3	brown	0.394035704	1.59E-10	0.582386611	1.20E-23
GNA12	brown	0.399936441	7.96E-11	0.6408407	9.94E-30
GPAM	brown	0.267301649	2.24E-05	0.399114592	8.77E-11
GPNMB	brown	0.220537295	0.000506657	0.098371585	0.124633749
GPR37L1	brown	0.265085608	2.63E-05	0.604946551	7.61E-26
GPT2	brown	0.329170635	1.34E-07	0.617205406	4.08E-27
GRAMD1C	brown	0.293533297	2.95E-06	0.324210068	2.11E-07
GSTM3	brown	0.351236589	1.60E-08	0.383428768	5.30E-10
HDAC1	brown	0.293319958	3.00E-06	0.529364909	4.26E-19
HEPACAM	brown	0.29495639	2.63E-06	0.475110711	3.36E-15
HEPH	brown	0.367057424	3.14E-09	0.411867767	1.89E-11
HSPB8	brown	0.323172921	2.32E-07	0.453836397	7.51E-14
ID4	brown	0.288733566	4.34E-06	0.293735449	2.90E-06
IL17RB	brown	0.405507131	4.10E-11	0.492630478	2.20E-16
INPPL1	brown	0.391984364	2.01E-10	0.71364826	1.94E-39
ITGA6	brown	0.334157741	8.39E-08	0.33386744	8.63E-08
ITGB5	brown	0.43736596	7.20E-13	0.681192763	9.22E-35
ITGB8	brown	0.328578799	1.41E-07	0.206839258	0.001128847
KAT2B	brown	0.334395756	8.21E-08	0.408318505	2.92E-11
KCNJ10	brown	0.38172679	6.41E-10	0.565044482	4.56E-22
KCNJ16	brown	0.313680357	5.39E-07	0.448380488	1.61E-13
KLF15	brown	0.388559064	2.97E-10	0.547265365	1.53E-20
LGALS3	brown	0.144604874	0.023588867	0.431677784	1.53E-12

LIFR	brown	0.244987235	0.000106954	0.328665844	1.40E-07
LRIG1	brown	0.296894948	2.24E-06	0.223384462	0.000426266
MAPKAPK3	brown	0.281798442	7.49E-06	0.545407203	2.18E-20
MAPRE1	brown	0.34466742	3.06E-08	0.411097546	2.08E-11
MEGF10	brown	0.314614813	4.97E-07	0.359573636	6.86E-09
MERTK	brown	0.312445851	6.01E-07	0.367823134	2.90E-09
MFGE8	brown	0.272061169	1.57E-05	0.603976023	9.54E-26
MSRB3	brown	0.43194226	1.48E-12	0.445530671	2.38E-13
MYBPC1	brown	0.367270008	3.07E-09	0.632825749	8.11E-29
MYO10	brown	0.252280902	6.51E-05	0.551426908	6.84E-21
NHS	brown	0.295599003	2.49E-06	0.415617037	1.19E-11
NPL	brown	0.317754897	3.76E-07	0.238639041	0.000162716
OGFRL1	brown	0.304925892	1.15E-06	0.207528968	0.001085499
P2RY14	brown	0.276141051	1.16E-05	0.168224782	0.008327411
PALLD	brown	0.380076645	7.69E-10	0.481123078	1.34E-15
PAPLN	brown	0.241108519	0.000138394	0.458229512	4.02E-14
PAQR8	brown	0.306292284	1.02E-06	0.609802127	2.42E-26
PDK4	brown	0.220067082	0.000521213	0.330610742	1.17E-07
PHGDH	brown	0.379694507	8.03E-10	0.526629289	6.97E-19
PLOD2	brown	0.322234158	2.52E-07	0.408097366	3.00E-11
PLSCR4	brown	0.29608002	2.40E-06	0.305509375	1.09E-06
PMP2	brown	0.185804742	0.003512794	0.140448235	0.027947238
POU3F2	brown	0.35389231	1.22E-08	0.455597221	5.85E-14
PPIC	brown	0.357492826	8.49E-09	0.271603454	1.63E-05
PRDM16	brown	0.347670194	2.28E-08	0.477111358	2.48E-15
PRDX6	brown	0.256162615	4.97E-05	0.410270375	2.30E-11
PRKD1	brown	0.326236657	1.75E-07	0.37619989	1.18E-09
PSAT1	brown	0.228004124	0.000320568	0.217904064	0.000593297
PSD2	brown	0.488762271	4.07E-16	0.487957377	4.63E-16
PTPN13	brown	0.324973941	1.97E-07	0.214413002	0.000729339
PTTG1IP	brown	0.380070159	7.70E-10	0.561521338	9.30E-22
QPRT	brown	0.359640348	6.81E-09	0.318114785	3.65E-07
RAB30	brown	0.342889175	3.64E-08	0.364920229	3.93E-09
RAB31	brown	0.387484828	3.36E-10	0.586101995	5.37E-24
RAMP1	brown	0.358351845	7.77E-09	0.528598475	4.90E-19
RANBP3L	brown	0.332131323	1.02E-07	0.365058145	3.88E-09
RFTN2	brown	0.397086773	1.11E-10	0.518045006	3.17E-18
RHOBTB3	brown	0.270860603	1.72E-05	0.365744374	3.61E-09
RHOJ	brown	0.398852835	9.05E-11	0.58574687	5.81E-24
RHPN2	brown	0.255287061	5.29E-05	0.392912147	1.81E-10

RIN2	brown	0.411658245	1.94E-11	0.397427055	1.07E-10
RXRA	brown	0.343519158	3.42E-08	0.600387879	2.19E-25
RYR3	brown	0.178710918	0.005022551	0.352976881	1.34E-08
SASH1	brown	0.405835456	3.94E-11	0.37039791	2.20E-09
SCARA3	brown	0.306235981	1.03E-06	0.383642752	5.18E-10
SFXN5	brown	0.198836418	0.001762072	0.431725576	1.52E-12
SLC14A1	brown	0.238096259	0.000168573	0.195908709	0.002065273
SLC25A18	brown	0.471786155	5.54E-15	0.609475801	2.62E-26
SLC39A12	brown	0.310336979	7.21E-07	0.142765475	0.025439404
SLC40A1	brown	0.253256857	6.09E-05	0.227626352	0.000328197
SLC44A2	brown	0.302217142	1.44E-06	0.619637306	2.25E-27
SLC9A9	brown	0.245743783	0.000101661	0.342484263	3.79E-08
SNAP23	brown	0.313472981	5.49E-07	0.466473097	1.22E-14
SNX5	brown	0.340379988	4.64E-08	0.282242377	7.23E-06
SOX2	brown	0.153587965	0.016126921	0.344133408	3.22E-08
SOX9	brown	0.303599195	1.28E-06	0.413285684	1.59E-11
SPPL2A	brown	0.234265564	0.000215855	0.187278302	0.003256199
SPR	brown	0.304092188	1.23E-06	0.302075326	1.46E-06
SSPN	brown	0.237763682	0.000172258	0.233093759	0.000232627
STK3	brown	0.237505829	0.000175167	0.325175114	1.93E-07
SYT17	brown	0.187897509	0.003153534	0.251737591	6.76E-05
TBL1X	brown	0.393371677	1.71E-10	0.684260374	3.54E-35
TCF12	brown	0.397686858	1.04E-10	0.405962914	3.88E-11
TFAP2C	brown	0.330987945	1.13E-07	0.424521117	3.87E-12
TGIF2	brown	0.287818218	4.67E-06	0.301406119	1.54E-06
TMED1	brown	0.249413947	7.93E-05	0.404019444	4.90E-11
TMEM123	brown	0.269076042	1.96E-05	0.203592957	0.001355038
TMEM47	brown	0.249890889	7.68E-05	0.383460813	5.28E-10
TNS3	brown	0.409488461	2.53E-11	0.499742314	6.96E-17
TPD52L1	brown	0.312457883	6.00E-07	0.281097329	7.90E-06
TRIM8	brown	0.325935538	1.80E-07	0.297134348	2.20E-06
TST	brown	0.162587275	0.010807544	0.398117002	9.86E-11
TXNIP	brown	0.249912981	7.66E-05	0.463866506	1.78E-14
UNG	brown	0.296247598	2.36E-06	0.474242513	3.83E-15
VCAN	brown	0.405465129	4.12E-11	0.610959915	1.84E-26
WFS1	brown	0.307804363	8.97E-07	0.613969174	8.94E-27
WWC1	brown	0.312790312	5.83E-07	0.55502571	3.38E-21
YAP1	brown	0.310963197	6.83E-07	0.401929357	6.29E-11
YES1	brown	0.350813775	1.67E-08	0.55470638	3.60E-21
ZFP36L1	brown	0.290957112	3.63E-06	0.436421802	8.17E-13

ZNF217	brown	0.350714046	1.68E-08	0.310232236	7.28E-07
ZNHIT6	brown	0.302679693	1.38E-06	0.246790844	9.47E-05
ABCA8	green	0.175062085	0.006007166	0.132227805	0.038621424
ACSL1	green	0.234296448	0.000215429	0.253569308	5.96E-05
ADA	green	0.27914128	9.19E-06	0.339699912	4.95E-08
ADARB2	green	0.222612786	0.000446797	0.356863403	9.05E-09
ADIPOR2	green	0.432345296	1.40E-12	0.51786204	3.27E-18
AIF1L	green	0.185731824	0.003525954	0.467247587	1.09E-14
ANLN	green	0.357410956	8.56E-09	0.353674397	1.25E-08
ANP32B	green	0.256947697	4.70E-05	0.499042814	7.81E-17
ARHGAP17	green	0.381354548	6.68E-10	0.630083438	1.64E-28
ARHGAP22	green	0.297798543	2.08E-06	0.382122766	6.13E-10
ARHGEF10	green	0.356852158	9.06E-09	0.584818714	7.11E-24
AZGP1	green	0.315634159	4.54E-07	0.2214329	0.000479969
BCAS1	green	0.213375355	0.000775011	0.41510059	1.27E-11
BTN2A1	green	-0.180527808	0.004588558	-0.246840553	9.44E-05
C1orf198	green	0.248618264	8.37E-05	0.359159648	7.16E-09
C21orf91	green	0.158063696	0.013248197	0.126470999	0.047995578
CAPN3	green	0.158690649	0.012883339	0.169174122	0.007963679
CCDC88A	green	0.13951868	0.029011212	0.214681835	0.000717918
CLCA4	green	0.264818319	2.68E-05	0.142475654	0.025742079
CLDN11	green	0.120851885	0.058907086	0.128081309	0.045199478
CNKSR3	green	0.369544162	2.41E-09	0.481386322	1.29E-15
COL4A5	green	0.197145665	0.001931795	0.205407307	0.001223964
CRTC3	green	0.242706362	0.000124518	0.550143619	8.77E-21
CTNNA3	green	0.225669646	0.000370487	0.135578839	0.033913796
CYP27A1	green	0.279272843	9.10E-06	0.358609404	7.57E-09
DAAM2	green	0.2018533	0.001492681	0.332277892	1.00E-07
EFHD1	green	0.309205101	7.95E-07	0.28364378	6.48E-06
EPN2	green	0.252135225	6.58E-05	0.537923148	8.89E-20
ERBB3	green	0.25379371	5.87E-05	0.373576948	1.57E-09
FAM107B	green	0.333555389	8.88E-08	0.578563256	2.73E-23
FGF1	green	0.200340591	0.00162265	0.14485859	0.023342956
FLNC	green	0.243751946	0.000116155	0.448789263	1.52E-13
FMNL2	green	0.352390289	1.42E-08	0.363757742	4.44E-09
GAB1	green	0.227573999	0.000329267	0.1647253	0.009799057
GAB2	green	0.192200858	0.002517313	0.326127387	1.77E-07
GLTP	green	0.307237344	9.41E-07	0.494661409	1.59E-16
GPIHBP1	green	0.155235247	0.015009424	0.36449359	4.11E-09
GPRC5B	green	0.321116225	2.79E-07	0.538787649	7.57E-20

HHATL	green	0.240521824	0.000143843	0.467388875	1.06E-14
HIP1	green	0.305130156	1.13E-06	0.205417522	0.00122326
HIP1R	green	0.207461914	0.001089646	0.330044961	1.23E-07
HSPA2	green	0.217821654	0.000596218	0.248808169	8.26E-05
KIF13B	green	0.282288384	7.21E-06	0.287874799	4.64E-06
LAMP2	green	0.214162182	0.000740145	0.311121498	6.74E-07
LHPP	green	0.263423732	2.96E-05	0.564277455	5.33E-22
LLGL1	green	0.262423138	3.19E-05	0.64415556	4.10E-30
LPAR1	green	0.229220543	0.000297108	0.387200719	3.47E-10
LRRC1	green	0.436282342	8.32E-13	0.470092325	7.13E-15
MAP4K4	green	0.34538308	2.85E-08	0.438646251	6.07E-13
MAP4K5	green	0.286255635	5.28E-06	0.434508028	1.05E-12
MYLIP	green	0.171404498	0.00716419	0.222405248	0.000452473
MYO9B	green	0.330244603	1.21E-07	0.539337899	6.83E-20
NASP	green	0.27191695	1.59E-05	0.478611819	1.97E-15
NDRG1	green	0.257564525	4.50E-05	0.449394471	1.40E-13
NEK7	green	0.356746933	9.16E-09	0.110268254	0.084996964
NKX2-2	green	0.294272172	2.78E-06	0.557853151	1.93E-21
NKX6-2	green	0.128040162	0.045269186	0.213958208	0.000749041
P2RX7	green	0.319358294	3.26E-07	0.382435903	5.92E-10
PAQR6	green	0.077350832	0.227683147	0.349327418	1.93E-08
PHLDB1	green	0.22432953	0.000402314	0.435370352	9.40E-13
PKP4	green	0.144451887	0.023738225	0.281399764	7.72E-06
PLP1	green	0.123142395	0.054234526	0.173970511	0.006333585
PLXDC2	green	0.387560387	3.33E-10	0.417078679	9.92E-12
PLXNB1	green	0.510720779	1.12E-17	0.605332015	6.95E-26
PPFIBP2	green	0.34011662	4.76E-08	0.617298893	3.99E-27
PTP4A2	green	0.252766486	6.30E-05	0.577579736	3.37E-23
RAPGEF3	green	0.433737738	1.17E-12	0.643768454	4.55E-30
RASGRP3	green	0.238459608	0.000164631	0.311850575	6.32E-07
RASSF2	green	0.201767913	0.001499756	0.558022366	1.87E-21
RHOU	green	0.258688019	4.16E-05	0.336663269	6.62E-08
RRBP1	green	0.302368706	1.42E-06	0.588607651	3.10E-24
RTKN	green	0.212704306	0.000805936	0.476977995	2.53E-15
S1PR5	green	0.179091528	0.004928696	0.163515165	0.01035912
SH3BP4	green	0.339641069	4.98E-08	0.348609038	2.08E-08
SLAIN1	green	0.158914351	0.01275531	0.326864969	1.65E-07
SLC44A1	green	0.249511897	7.88E-05	0.245470436	0.000103544
SLC5A11	green	0.269138486	1.95E-05	0.32853013	1.42E-07
SLC6A9	green	0.38457896	4.66E-10	0.402974081	5.55E-11

SLCO1A2	green	0.225496988	0.000374452	0.053402294	0.40529753
SNX6	green	0.215306254	0.000692026	0.584782333	7.16E-24
SOX8	green	0.186759233	0.003344583	0.506911706	2.12E-17
SREBF1	green	0.321259108	2.75E-07	0.264052904	2.83E-05
ST18	green	0.182747107	0.004104402	0.142585968	0.02562651
TF	green	0.155065425	0.015121407	0.106684176	0.095695534
TGFB3	green	0.188030347	0.003131895	0.456085463	5.46E-14
THBS2	green	0.282181683	7.27E-06	0.227194999	0.000337114
TJAP1	green	0.246274591	9.81E-05	0.604206691	9.04E-26
TMC6	green	0.361922013	5.38E-09	0.439901439	5.13E-13
TMCC3	green	0.283009514	6.81E-06	0.259127042	4.03E-05
TRIM59	green	0.293460119	2.97E-06	0.318770656	3.44E-07
UGT8	green	0.204940909	0.001256498	0.067056618	0.295832825
VAMP3	green	0.337686711	6.01E-08	0.439615364	5.33E-13
VEZF1	green	0.434923132	9.97E-13	0.566986115	3.07E-22
VRK2	green	0.260032405	3.78E-05	0.289437425	4.10E-06
WSB1	green	0.176695068	0.005546935	0.333797013	8.68E-08
ZCCHC24	green	0.370341716	2.22E-09	0.624602252	6.56E-28
ZEB2	green	0.134064201	0.035977509	0.260886987	3.56E-05
ZNF565	green	0.361270018	5.75E-09	0.570600374	1.46E-22
ABI3BP	grey	0.188784591	0.00301156	0.121674458	0.057192228
ADAMTS9	grey	0.240070715	0.000148169	0.1862151	0.00343956
AKR1C2	grey	0.35823165	7.87E-09	0.312777342	5.83E-07
ALDH1A1	grey	-0.212163013	0.000831707	0.043843079	0.494561506
ALMS1	grey	0.254137011	5.73E-05	0.229689058	0.000288507
ANKDD1A	grey	0.19258483	0.002466642	0.350197394	1.77E-08
ANKRD36B	grey	0.223855162	0.000414176	0.413575205	1.53E-11
ARMC3	grey	0.269623701	1.88E-05	0.139163687	0.029426586
ARMCX4	grey	-0.171877227	0.007004233	-0.211489094	0.000864854
ATF4	grey	0.160425762	0.011919248	0.198512493	0.001793494
C19orf18	grey	0.208121696	0.001049476	0.251631432	6.81E-05
CASP6	grey	0.19218725	0.002519126	0.23026744	0.000278208
CCL2	grey	0.242011992	0.00013038	0.034453926	0.591481301
CD109	grey	0.286626995	5.13E-06	0.072726598	0.256781098
CEP70	grey	0.223180222	0.000431613	0.25436446	5.64E-05
COL11A1	grey	0.270224064	1.80E-05	0.32834861	1.44E-07
CTBS	grey	0.272511381	1.52E-05	0.273410685	1.42E-05
CTSZ	grey	0.139491087	0.029043318	0.238731247	0.00016174
CX3CR1	grey	-0.0527003	0.411508153	-0.323111991	2.33E-07
CXCL2	grey	0.19648318	0.002002271	0.099448276	0.120542126

CXCR4	grey	0.152202502	0.017122398	0.073202275	0.253674705
DSP	grey	0.153096415	0.016474164	0.071894652	0.262276975
EDNRA	grey	0.315877434	4.45E-07	0.200582634	0.001601182
FAM114A1	grey	0.281632058	7.58E-06	0.013453975	0.834041901
FN1	grey	0.268072804	2.11E-05	0.043692647	0.49604502
FOS	grey	0.26631784	2.40E-05	0.045254927	0.480753954
FOSB	grey	-0.088122113	0.169146466	0.084891718	0.185382826
FUBP3	grey	0.094605963	0.139794088	-0.075182789	0.241020984
GADD45B	grey	0.243639033	0.000117032	0.127555146	0.046097686
HIBCH	grey	0.175599084	0.005852182	0.333947201	8.56E-08
IGFBP5	grey	0.256954601	4.70E-05	0.042801456	0.504881833
ITGB2	grey	0.261333935	3.45E-05	0.113662256	0.075773088
KCTD18	grey	0.15106487	0.01797945	0.037130646	0.56298476
MDK	grey	-0.261082393	3.51E-05	-0.127707707	0.045835725
MPL	grey	-0.088594285	0.16686494	-0.122309961	0.0558958
NEDD1	grey	0.225367323	0.000377456	0.037596168	0.558096808
NKX6-3	grey	0.206442149	0.00115452	0.036100008	0.573878859
OSMR	grey	0.260030172	3.78E-05	0.063667851	0.320968146
PART1	grey	-0.204183004	0.001311063	-0.013933781	0.828213542
PAWR	grey	0.264762187	2.69E-05	0.161922126	0.011139462
PCDH18	grey	0.338143246	5.75E-08	0.205108898	0.001244689
PDLIM1	grey	0.156481931	0.014209338	0.060711793	0.343993924
PFKFB3	grey	0.326883921	1.65E-07	0.08987288	0.160802269
PIPOX	grey	0.211580369	0.000860295	0.077546312	0.226506768
RAB20	grey	0.231410268	0.000258858	0.05405775	0.399549203
RAB3B	grey	-0.219913602	0.000526048	-0.214524774	0.00072457
RBMS3	grey	0.265676577	2.52E-05	0.081454852	0.20388446
RBPMS	grey	0.14044465	0.027951276	0.075987026	0.236010904
RGS1	grey	0.301947863	1.47E-06	0.067824204	0.290326497
RHOD	grey	0.136280995	0.032991758	0.074718608	0.243946292
RPH3A	grey	-0.345136595	2.92E-08	0.167230822	0.008723996
SCIN	grey	0.335896774	7.12E-08	0.139944859	0.028519179
SDR16C5	grey	-0.242494452	0.00012628	-0.022662528	0.724121483
SEZ6	grey	-0.312534681	5.96E-07	0.00873783	0.891764499
SGPP2	grey	-0.066503051	0.29984671	0.044046248	0.492561668
SLC16A1	grey	0.191506957	0.002611289	0.030917004	0.630112889
SLIT3	grey	-0.217139466	0.00062091	-0.136458926	0.032761524
SPP1	grey	0.26880353	2.00E-05	0.089648083	0.161856025
ST3GAL1	grey	0.252231495	6.54E-05	0.304953682	1.14E-06
SYS1	grey	0.3130067	5.72E-07	0.207258083	0.001102338

TCEA3	grey	0.230023909	0.000282502	0.305621498	1.08E-06
TFEB	grey	0.194288339	0.00225284	0.084374318	0.188086352
TFG	grey	-0.24795579	8.76E-05	-0.260725391	3.60E-05
TGFBI	grey	0.303724576	1.27E-06	0.151804262	0.017418277
TRIM22	grey	0.288773735	4.32E-06	-0.017402898	0.786371112
TRMT5	grey	-0.362180516	5.24E-09	-0.423452574	4.43E-12
USP46	grey	0.333364941	9.04E-08	0.195595373	0.002100393
ZBBX	grey	-0.232504597	0.000241511	-0.105626436	0.099049847
ZC3H7B	grey	0.138534614	0.03017515	0.099708151	0.119570383
ABCC8	red	-0.238065231	0.000168913	0.157986146	0.013293953
AK5	red	-0.218471911	0.00057353	-0.422145436	5.24E-12
ANO3	red	-0.321996948	2.58E-07	-0.307501018	9.20E-07
AP2S1	red	-0.305357277	1.10E-06	-0.24943361	7.92E-05
AP3M2	red	-0.289192257	4.18E-06	-0.356815938	9.10E-09
CAMKV	red	-0.245841028	0.000100998	-0.306130419	1.03E-06
CBLN2	red	-0.175758521	0.005806861	-0.383842198	5.06E-10
CCK	red	-0.172908387	0.006666332	-0.366581502	3.30E-09
CD200	red	-0.160230935	0.012024236	0.011585169	0.856826564
CHGA	red	-0.107331888	0.093686535	-0.15044569	0.01846143
CHN1	red	-0.38696693	3.56E-10	-0.540601646	5.40E-20
CLIP3	red	-0.449205011	1.44E-13	-0.139018656	0.029597742
DGKB	red	-0.287270001	4.87E-06	-0.32773197	1.53E-07
DLGAP2	red	-0.235235414	0.000202835	-0.188500773	0.003056341
DNAJC5G	red	-0.227204366	0.000336918	-0.183003901	0.004051464
DPF3	red	0.064609488	0.313848603	0.241152919	0.00013799
EGR3	red	-0.238067181	0.000168892	-0.28656436	5.15E-06
FAM81A	red	-0.211350667	0.000871812	-0.167581994	0.008582004
FIBP	red	-0.352684392	1.38E-08	-0.184855873	0.00368756
GDA	red	-0.213252144	0.000780605	-0.205873183	0.001192239
GFRA2	red	-0.229898903	0.00028473	0.023812503	0.710733544
GPD1L	red	-0.223210955	0.000430804	-0.148942914	0.019678206
HECW1	red	-0.18516076	0.003630567	-0.178939254	0.004966054
HHAT	red	0.299413318	1.82E-06	0.148895732	0.01971751
KALRN	red	-0.207069049	0.001114231	-0.266255649	2.41E-05
KCNC2	red	-0.193927702	0.002296641	-0.320950897	2.83E-07
KCNF1	red	-0.357272338	8.68E-09	-0.227224762	0.000336492
KCNS1	red	-0.098866441	0.122740032	0.102898765	0.108130913
KCNS2	red	-0.194972044	0.002171882	-0.168052313	0.008395054
KHDRBS3	red	-0.180368266	0.004625268	-0.142280294	0.025947846
LDB2	red	-0.341958029	3.98E-08	-0.500768384	5.88E-17

LMO4	red	-0.215710222	0.000675738	-0.140868382	0.027477414
LMTK2	red	-0.265051973	2.63E-05	-0.241319785	0.00013648
LRRC4C	red	-0.122957163	0.054600606	-0.32176405	2.63E-07
MEF2C	red	-0.148116461	0.020376586	-0.277402701	1.05E-05
MOXD1	red	-0.073545142	0.251451813	-0.091842273	0.151789821
MTX2	red	-0.324445549	2.06E-07	-0.435299546	9.49E-13
NCALD	red	-0.360212124	6.42E-09	-0.385761076	4.08E-10
NEFL	red	-0.156092171	0.014455359	0.025651974	0.689503254
NME5	red	-0.244954787	0.000107186	-0.32479899	2.00E-07
PDK3	red	-0.199693756	0.00168132	-0.12367952	0.053184488
PFDN4	red	-0.280557436	8.24E-06	-0.257529159	4.52E-05
PLCB1	red	-0.417819619	9.04E-12	-0.29670023	2.28E-06
PNMA3	red	-0.311220912	6.68E-07	-0.144312796	0.023874723
POPDC3	red	-0.177197808	0.005411754	-0.097565924	0.127765204
PPP2R2C	red	-0.396631675	1.17E-10	-0.269587564	1.89E-05
RAB27B	red	-0.122522143	0.055468403	-0.03080879	0.631311583
RAB3C	red	-0.200607657	0.001598977	-0.160020181	0.012138722
RASL10A	red	-0.178974423	0.004957403	-0.19598029	0.002057325
RIMS3	red	-0.141833979	0.026423245	-0.191804636	0.00257059
RPRML	red	-0.314171458	5.16E-07	-0.186430661	0.003401646
SERPINF1	red	-0.170903924	0.007337113	-0.218569804	0.000570184
SH3BGRL2	red	-0.188191749	0.003105784	-0.18653969	0.003382614
SH3KBP1	red	-0.112852203	0.077897281	-0.156995089	0.01389103
SHANK2	red	-0.260169665	3.74E-05	-0.190769354	0.002714668
SLC17A6	red	-0.254879577	5.44E-05	-0.315341692	4.66E-07
SLC30A3	red	-0.388132377	3.12E-10	-0.343962889	3.28E-08
SLIT2	red	-0.084876468	0.185462099	-0.124960843	0.050747606
SMAD1	red	0.194460433	0.002232208	-0.02021676	0.752870814
SORCS1	red	-0.164862137	0.009737447	0.023236017	0.717434183
TBR1	red	-0.214285787	0.000734801	-0.479643287	1.69E-15
TMEM130	red	-0.325293255	1.91E-07	-0.496844007	1.12E-16
TMEM158	red	-0.255437211	5.23E-05	-0.171716259	0.007058338
VIPR1	red	-0.294980112	2.62E-06	-0.00160906	0.980009513
VSTM2A	red	-0.061200787	0.340114324	-0.195368712	0.002126137
WNT10B	red	-0.200647324	0.001595488	-0.198974168	0.001748863
ZIC1	red	0.052593738	0.412455801	0.050766592	0.428903522
AASDHPPT	turquoise	-0.338092574	5.78E-08	-0.584121032	8.27E-24
ABCA5	turquoise	-0.247808879	8.84E-05	-0.321552591	2.68E-07
ABCC12	turquoise	-0.221884344	0.000467016	-0.297377455	2.15E-06
ABCE1	turquoise	-0.26405095	2.83E-05	-0.543083707	3.38E-20

ACOT7	turquoise	-0.318730504	3.45E-07	-0.339091596	5.25E-08
ACP1	turquoise	-0.313504765	5.47E-07	-0.80046816	6.10E-56
ACSF2	turquoise	0.365618901	3.66E-09	0.455118305	6.26E-14
ACSL3	turquoise	-0.310694437	6.99E-07	-0.593972049	9.39E-25
ACSL4	turquoise	-0.315327663	4.67E-07	-0.651751629	5.15E-31
ACTA2	turquoise	0.251158804	7.04E-05	0.168135474	0.008362377
ACTL6B	turquoise	-0.165809873	0.009320069	-0.228731487	0.000306341
ACTN2	turquoise	-0.266782801	2.32E-05	-0.43964261	5.31E-13
ACTR10	turquoise	-0.36131635	5.73E-09	-0.764624671	2.89E-48
ACTR2	turquoise	-0.210168024	0.000933389	-0.529950352	3.84E-19
ACTR6	turquoise	-0.355298662	1.06E-08	-0.424490576	3.88E-12
ACYP2	turquoise	-0.231084677	0.000264239	-0.619089838	2.57E-27
ADAM23	turquoise	-0.295881921	2.43E-06	-0.441782723	3.98E-13
ADAMTS1	turquoise	0.393907784	1.61E-10	0.261743969	3.35E-05
ADARB1	turquoise	-0.279636338	8.85E-06	-0.279847612	8.70E-06
ADCY1	turquoise	-0.356131114	9.75E-09	-0.706090905	2.72E-38
ADCY4	turquoise	0.196908913	0.001956717	0.399866556	8.03E-11
ADCY7	turquoise	0.22720159	0.000336976	0.281125561	7.89E-06
ADCYAP1	turquoise	-0.427499173	2.64E-12	-0.347533568	2.31E-08
ADORA2A	turquoise	0.291123356	3.58E-06	0.488706501	4.11E-16
AEBP1	turquoise	0.472094983	5.29E-15	0.346148978	2.65E-08
AFF1	turquoise	0.378011561	9.66E-10	0.266350169	2.40E-05
AFF2	turquoise	-0.277581625	1.04E-05	-0.398040962	9.95E-11
AFF3	turquoise	-0.196818586	0.001966302	-0.177151126	0.005424181
AFTPH	turquoise	-0.117536204	0.066254875	-0.553243106	4.80E-21
AGFG2	turquoise	0.294678602	2.69E-06	0.347415647	2.34E-08
AGK	turquoise	-0.333944423	8.56E-08	-0.669968606	2.78E-33
AHI1	turquoise	-0.237607334	0.000174016	-0.276538779	1.12E-05
AIFM3	turquoise	0.379156192	8.52E-10	0.345649652	2.78E-08
AIG1	turquoise	-0.341687497	4.09E-08	-0.537807773	9.09E-20
AKAP11	turquoise	-0.384107466	4.91E-10	-0.681680405	7.92E-35
AKAP6	turquoise	-0.274178627	1.34E-05	-0.459935093	3.15E-14
AKAP8L	turquoise	0.169250364	0.00793509	0.278829643	9.41E-06
AKIRIN2	turquoise	-0.282367766	7.16E-06	-0.723677642	5.12E-41
ALDH1L1	turquoise	0.201141442	0.001552606	0.364278671	4.21E-09
ALDH5A1	turquoise	-0.295447608	2.52E-06	-0.486033194	6.26E-16
AMN1	turquoise	-0.265487827	2.55E-05	-0.364884853	3.95E-09
AMPH	turquoise	-0.217717223	0.000599938	-0.609144891	2.83E-26
ANAPC10	turquoise	-0.236524804	0.000186658	-0.391766171	2.06E-10
ANAPC13	turquoise	-0.219662563	0.000534045	-0.495483975	1.39E-16

ANGPT1	turquoise	0.294727692	2.68E-06	0.309623332	7.67E-07
ANGPT2	turquoise	0.317637659	3.80E-07	0.344204009	3.20E-08
ANGPTL4	turquoise	0.15457665	0.015447804	0.381171653	6.82E-10
ANKMY2	turquoise	-0.208771344	0.001011257	-0.58631041	5.13E-24
ANKRD13D	turquoise	0.087650833	0.171446789	0.593707314	9.97E-25
ANKRD18A	turquoise	0.309742339	7.59E-07	0.532109576	2.59E-19
ANKRD40	turquoise	0.231124366	0.000263578	0.372893189	1.69E-09
ANKRD46	turquoise	-0.291215562	3.56E-06	-0.641917212	7.46E-30
ANKRD9	turquoise	0.206063359	0.001179508	0.29205853	3.32E-06
ANXA2	turquoise	0.337396967	6.17E-08	0.324362734	2.08E-07
ANXA6	turquoise	-0.296638408	2.29E-06	-0.340186066	4.73E-08
AP3S1	turquoise	-0.258420805	4.24E-05	-0.707545724	1.65E-38
APBA2	turquoise	-0.231337207	0.000260057	-0.392709884	1.85E-10
APLNR	turquoise	0.41559869	1.19E-11	0.277117101	1.07E-05
APOC1	turquoise	0.293826533	2.88E-06	0.291444684	3.49E-06
APOL3	turquoise	0.194269224	0.002255142	0.384607347	4.65E-10
APOLD1	turquoise	0.393741562	1.64E-10	0.229620605	0.000289749
APOO	turquoise	-0.259901967	3.82E-05	-0.7212559	1.25E-40
AQP1	turquoise	0.220864226	0.00049676	0.136058246	0.033281924
ARAP1	turquoise	0.266683934	2.34E-05	0.434910746	9.99E-13
ARAP3	turquoise	0.212079152	0.000835767	0.511666915	9.50E-18
ARF1	turquoise	-0.243915829	0.000114893	-0.334986026	7.76E-08
ARHGAP20	turquoise	-0.354703141	1.13E-08	-0.58491046	6.97E-24
ARHGAP4	turquoise	0.297496452	2.13E-06	0.387805697	3.24E-10
ARHGEF3	turquoise	-0.317078909	4.00E-07	-0.404924129	4.40E-11
ARID3B	turquoise	0.182092125	0.004242263	0.491983293	2.44E-16
ARL4A	turquoise	-0.153563804	0.016143839	-0.273157175	1.45E-05
ARL6IP1	turquoise	-0.350868058	1.66E-08	-0.694690702	1.25E-36
ARMC10	turquoise	-0.300611971	1.65E-06	-0.749425075	2.07E-45
ARMCX5	turquoise	-0.268485633	2.05E-05	-0.580695901	1.73E-23
ARPC1A	turquoise	-0.144083974	0.024100752	-0.423928115	4.17E-12
ARPC5L	turquoise	-0.274517388	1.31E-05	-0.379850252	7.89E-10
ARRB1	turquoise	0.267085081	2.27E-05	0.48964383	3.54E-16
ARRDC2	turquoise	0.135661148	0.033804591	0.534577969	1.65E-19
ARV1	turquoise	-0.182208916	0.004217379	-0.407555364	3.20E-11
ASAP3	turquoise	0.332502115	9.81E-08	0.607112431	4.58E-26
ASNS	turquoise	-0.436819128	7.75E-13	-0.479836616	1.64E-15
ASTN1	turquoise	-0.263862293	2.87E-05	-0.550183677	8.70E-21
ATAD1	turquoise	-0.307082775	9.54E-07	-0.408555851	2.84E-11
ATAD3A	turquoise	0.209140399	0.000990121	0.333381721	9.03E-08

ATCAY	turquoise	-0.210322323	0.000925134	-0.466040457	1.30E-14
ATL1	turquoise	-0.318265752	3.60E-07	-0.538099215	8.61E-20
ATP10A	turquoise	0.272651016	1.50E-05	0.441291162	4.25E-13
ATP11C	turquoise	0.29058144	3.74E-06	0.275856338	1.18E-05
ATP1A1	turquoise	-0.36558235	3.67E-09	-0.603111324	1.17E-25
ATP1B1	turquoise	-0.411421412	2.00E-11	-0.636220161	3.36E-29
ATP2A2	turquoise	-0.426556375	2.98E-12	-0.71754995	4.81E-40
ATP2B1	turquoise	-0.365549254	3.68E-09	-0.266988252	2.29E-05
ATP2B2	turquoise	-0.335874787	7.14E-08	-0.449796025	1.32E-13
ATP2B3	turquoise	-0.375833707	1.23E-09	-0.660167983	4.83E-32
ATP6AP1	turquoise	-0.329308604	1.32E-07	-0.111358413	0.081940662
ATP6AP2	turquoise	-0.299433326	1.82E-06	-0.654635348	2.31E-31
ATP6V0E1	turquoise	0.300223352	1.70E-06	0.591982361	1.47E-24
ATP6V1B2	turquoise	-0.442578572	3.57E-13	-0.568455712	2.27E-22
ATP6V1C1	turquoise	-0.286104355	5.34E-06	-0.462628136	2.13E-14
ATP6V1D	turquoise	-0.401842515	6.35E-11	-0.627382476	3.26E-28
ATP6V1E1	turquoise	-0.469989982	7.24E-15	-0.506267538	2.36E-17
ATP6V1G2	turquoise	-0.497018798	1.09E-16	-0.736664477	3.61E-43
ATP6V1H	turquoise	-0.300117503	1.72E-06	-0.640195138	1.18E-29
ATRN	turquoise	-0.3530111	1.34E-08	-0.609315143	2.72E-26
ATRNL1	turquoise	-0.222158723	0.000459303	-0.570583773	1.46E-22
AUH	turquoise	-0.281441261	7.70E-06	-0.668486409	4.31E-33
AZIN1	turquoise	-0.236611088	0.00018562	-0.721666916	1.07E-40
B3GALNT1	turquoise	-0.351433727	1.57E-08	-0.540021102	6.01E-20
B3GNT6	turquoise	-0.246878787	9.42E-05	-0.712293688	3.14E-39
B4GALT1	turquoise	0.190693491	0.002725509	0.143282329	0.024907218
B4GALT4	turquoise	0.393800113	1.63E-10	0.593635153	1.01E-24
B4GALT6	turquoise	-0.352308974	1.44E-08	-0.541292734	4.74E-20
BACE2	turquoise	0.390865999	2.29E-10	0.373029432	1.66E-09
BAG3	turquoise	0.272149234	1.56E-05	0.382194105	6.08E-10
BAIAP2	turquoise	-0.249189069	8.05E-05	-0.371305064	2.00E-09
BARD1	turquoise	0.301185287	1.57E-06	0.322109772	2.55E-07
BASP1	turquoise	-0.342285839	3.86E-08	-0.639322119	1.49E-29
BAZ1A	turquoise	0.443206731	3.28E-13	0.420632752	6.34E-12
BAZ2B	turquoise	0.281466012	7.68E-06	0.613057505	1.11E-26
BBX	turquoise	0.315465559	4.61E-07	0.410182804	2.33E-11
BCAN	turquoise	0.183486899	0.003953559	0.3529554	1.35E-08
BCAS2	turquoise	-0.177487165	0.005335297	-0.338521904	5.55E-08
BCAT1	turquoise	-0.273291743	1.43E-05	-0.521991294	1.59E-18
BCL6	turquoise	0.398944821	8.95E-11	0.58049569	1.81E-23

BDNF	turquoise	-0.383642166	5.18E-10	-0.354564633	1.14E-08
BEND6	turquoise	-0.325489718	1.88E-07	-0.553950783	4.18E-21
BEX1	turquoise	-0.433289904	1.24E-12	-0.702147048	1.04E-37
BEX2	turquoise	-0.426896998	2.85E-12	-0.692994113	2.17E-36
BGN	turquoise	0.427584197	2.61E-12	0.600078294	2.35E-25
BHLHB9	turquoise	-0.207591155	0.001081667	-0.49742246	1.02E-16
BHLHE22	turquoise	-0.147873341	0.020586078	-0.441701992	4.02E-13
BMPER	turquoise	-0.259109922	4.04E-05	-0.61017346	2.22E-26
BMPR2	turquoise	-0.168117654	0.00836937	-0.558204537	1.80E-21
BNIP3	turquoise	-0.388687034	2.93E-10	-0.461760476	2.42E-14
BPTF	turquoise	0.466707508	1.18E-14	0.354697877	1.13E-08
BRCA1	turquoise	0.245584082	0.000102757	0.293158335	3.04E-06
BRWD1	turquoise	-0.360266334	6.38E-09	-0.649103871	1.07E-30
BSCL2	turquoise	-0.432099611	1.45E-12	-0.322559685	2.45E-07
BSN	turquoise	-0.378283062	9.37E-10	-0.257321112	4.58E-05
BST2	turquoise	0.129323227	0.04313742	0.594748708	7.88E-25
BTBD10	turquoise	-0.333700247	8.76E-08	-0.722686573	7.38E-41
BTBD11	turquoise	-0.193347462	0.002368744	-0.252097156	6.60E-05
BTBD3	turquoise	-0.328868829	1.37E-07	-0.547055179	1.59E-20
BTF3L4	turquoise	-0.241081493	0.000138641	-0.638935034	1.65E-29
BZW2	turquoise	-0.187581938	0.003205483	-0.470633434	6.58E-15
C11orf58	turquoise	-0.220373821	0.000511675	-0.515858671	4.63E-18
C11orf87	turquoise	-0.227766293	0.000325351	-0.460734085	2.81E-14
C14orf132	turquoise	-0.287617664	4.74E-06	-0.311326341	6.62E-07
C14orf93	turquoise	0.292309591	3.26E-06	0.39574011	1.30E-10
C18orf32	turquoise	-0.218805366	0.000562208	-0.123926942	0.052706504
C19orf54	turquoise	0.28165708	7.57E-06	0.315588755	4.56E-07
C1orf162	turquoise	0.316484092	4.21E-07	0.427969095	2.48E-12
C1orf216	turquoise	-0.235029865	0.000205532	-0.444081259	2.91E-13
C1QTNF1	turquoise	0.352155744	1.46E-08	0.522771957	1.38E-18
C1QTNF5	turquoise	0.375125747	1.32E-09	0.216895603	0.000629964
C2CD2	turquoise	0.317566347	3.83E-07	0.67416898	7.91E-34
C3orf14	turquoise	-0.27577592	1.19E-05	-0.564195936	5.42E-22
C4orf33	turquoise	-0.185363567	0.003593097	-0.324410864	2.07E-07
C5orf22	turquoise	-0.398375894	9.57E-11	-0.639710319	1.34E-29
C7	turquoise	0.286005863	5.39E-06	0.122502391	0.055508075
CA10	turquoise	-0.424142168	4.06E-12	-0.442047538	3.84E-13
CAB39	turquoise	-0.253956449	5.80E-05	-0.609587503	2.55E-26
CAB39L	turquoise	0.416490815	1.07E-11	0.367851721	2.89E-09
CABYR	turquoise	-0.228935509	0.000302457	-0.402560474	5.83E-11

CACNA2D3	turquoise	-0.18374298	0.003902522	-0.397609118	1.05E-10
CACNB2	turquoise	-0.37317719	1.63E-09	-0.560197324	1.21E-21
CACNB4	turquoise	-0.211003597	0.000889483	-0.19305336	0.002406071
CADM4	turquoise	0.32105919	2.80E-07	0.629305728	2.00E-28
CADPS	turquoise	-0.317434909	3.87E-07	-0.727619785	1.17E-41
CADPS2	turquoise	-0.178506101	0.005073718	-0.384797946	4.55E-10
CALB1	turquoise	-0.297967514	2.05E-06	-0.231057401	0.000264695
CALD1	turquoise	0.313375874	5.54E-07	0.739620673	1.12E-43
CALM1	turquoise	-0.333001675	9.36E-08	-0.717546535	4.82E-40
CALML4	turquoise	0.270626225	1.75E-05	0.388245385	3.08E-10
CAMK2D	turquoise	-0.260286268	3.71E-05	-0.219028737	0.000554739
CAMK4	turquoise	-0.331247238	1.10E-07	-0.358803762	7.42E-09
CAMTA1	turquoise	-0.358043825	8.02E-09	-0.389914825	2.55E-10
CAP2	turquoise	-0.403090611	5.47E-11	-0.550216868	8.64E-21
CAPRIN1	turquoise	-0.420330781	6.59E-12	-0.4147225	1.33E-11
CAPRIN2	turquoise	-0.296255267	2.36E-06	-0.426062867	3.17E-12
CAPS	turquoise	0.432481982	1.38E-12	0.691645658	3.36E-36
CARHSP1	turquoise	0.210526604	0.000914308	0.435808391	8.87E-13
CARTPT	turquoise	-0.34160879	4.12E-08	-0.249922117	7.66E-05
CASC3	turquoise	0.299281649	1.84E-06	0.271060136	1.69E-05
CASP7	turquoise	0.174216842	0.006258568	0.272592008	1.51E-05
CAV1	turquoise	0.326782948	1.67E-07	0.272432735	1.53E-05
CBLB	turquoise	0.37709485	1.07E-09	0.525281349	8.87E-19
CBLN4	turquoise	-0.33299962	9.36E-08	-0.605198745	7.17E-26
CBS	turquoise	0.307215011	9.43E-07	0.568999026	2.03E-22
CCDC102A	turquoise	0.38685375	3.61E-10	0.578117762	3.01E-23
CCDC151	turquoise	0.230634018	0.000271859	0.588089983	3.47E-24
CCKBR	turquoise	-0.382533836	5.86E-10	-0.561698098	8.97E-22
CCL5	turquoise	0.370183021	2.25E-09	0.471607963	5.69E-15
CCND1	turquoise	0.183302009	0.003990782	0.395089793	1.40E-10
CCNDBP1	turquoise	-0.269270255	1.93E-05	-0.72043592	1.69E-40
CCNH	turquoise	-0.253555893	5.96E-05	-0.443339731	3.22E-13
CCT4	turquoise	-0.122574468	0.055363422	-0.184165691	0.003819575
CD151	turquoise	0.355142447	1.08E-08	0.708378604	1.23E-38
CD163	turquoise	0.279011104	9.28E-06	0.114175216	0.074452418
CD24	turquoise	-0.178103726	0.005175606	-0.305221526	1.12E-06
CD34	turquoise	0.337840086	5.92E-08	0.389191062	2.77E-10
CD44	turquoise	0.322901179	2.37E-07	0.144966107	0.023239416
CD47	turquoise	-0.296009731	2.41E-06	-0.504844272	3.00E-17
CD63	turquoise	0.282427463	7.13E-06	0.496049765	1.27E-16

CD79B	turquoise	0.249794785	7.73E-05	0.625106096	5.78E-28
CD83	turquoise	-0.262384834	3.20E-05	-0.477101396	2.49E-15
CDC40	turquoise	-0.245563294	0.000102901	-0.504490601	3.18E-17
CDC42SE2	turquoise	-0.278900953	9.36E-06	-0.369745888	2.36E-09
CDH10	turquoise	-0.271169164	1.68E-05	-0.400634993	7.33E-11
CDH12	turquoise	-0.261225	3.47E-05	-0.436841898	7.73E-13
CDH13	turquoise	-0.258751151	4.14E-05	-0.505971314	2.48E-17
CDH23	turquoise	0.297771093	2.08E-06	0.311621575	6.45E-07
CDH8	turquoise	-0.189636037	0.002880766	-0.411124211	2.07E-11
CDK5	turquoise	-0.28604284	5.37E-06	-0.413534602	1.54E-11
CDK5RAP2	turquoise	0.273546906	1.41E-05	0.6033955	1.09E-25
CDK7	turquoise	-0.208055271	0.001053457	-0.05431873	0.39727406
CDKN1A	turquoise	0.249379373	7.95E-05	0.282680729	6.99E-06
CDO1	turquoise	-0.246600146	9.60E-05	-0.386128368	3.92E-10
CDR2L	turquoise	0.125487906	0.049772555	0.575183827	5.59E-23
CEBPD	turquoise	0.323956072	2.16E-07	0.364248784	4.22E-09
CEP164	turquoise	0.230699102	0.000270746	0.583131516	1.03E-23
CEP290	turquoise	0.281239669	7.82E-06	0.621582395	1.39E-27
CFH	turquoise	0.240200716	0.00014691	0.294470806	2.73E-06
CFI	turquoise	0.265090595	2.63E-05	0.276626137	1.11E-05
CFLAR	turquoise	0.399802542	8.09E-11	0.798421707	1.84E-55
CHCHD2	turquoise	-0.348811401	2.04E-08	-0.500591349	6.06E-17
CHCHD6	turquoise	-0.241991897	0.000130553	-0.534338634	1.72E-19
CHD2	turquoise	0.206515912	0.001149711	0.214017582	0.000746442
CHDH	turquoise	0.388297826	3.06E-10	0.542288765	3.93E-20
CHGB	turquoise	-0.412165029	1.82E-11	-0.718256018	3.73E-40
CHL1	turquoise	-0.272484011	1.52E-05	-0.646832145	1.99E-30
CHM	turquoise	-0.210001015	0.0009424	-0.364764044	4.00E-09
CHMP1B	turquoise	-0.129829765	0.042319358	-0.618122736	3.26E-27
CHMP4B	turquoise	-0.383127924	5.48E-10	-0.403755574	5.06E-11
CHRM3	turquoise	-0.098515138	0.124082085	-0.354993	1.09E-08
CHST6	turquoise	0.498355648	8.73E-17	0.504529459	3.16E-17
CHSY1	turquoise	0.236624975	0.000185454	0.369514489	2.42E-09
CHURC1	turquoise	-0.086970332	0.174809279	-0.194060711	0.002280397
CIB2	turquoise	0.287514822	4.78E-06	0.379120147	8.55E-10
CIC	turquoise	0.269141304	1.95E-05	0.12276492	0.054982704
CIRBP	turquoise	-0.335056423	7.71E-08	-0.495099671	1.48E-16
CISD1	turquoise	-0.447319631	1.86E-13	-0.533444442	2.03E-19
CITED2	turquoise	-0.334718955	7.96E-08	-0.433129441	1.26E-12
CLASP2	turquoise	-0.22715261	0.000338003	-0.708713089	1.10E-38

CLCN4	turquoise	-0.225271386	0.000379692	-0.488260366	4.41E-16
CLDN15	turquoise	0.334512929	8.12E-08	0.487108749	5.29E-16
CLDN5	turquoise	0.329414394	1.31E-07	0.516192314	4.37E-18
CLIC1	turquoise	0.373550968	1.57E-09	0.26621875	2.42E-05
CLIP2	turquoise	0.227778985	0.000325094	0.251198046	7.02E-05
CLTA	turquoise	-0.394729592	1.46E-10	-0.468084548	9.60E-15
CLTC	turquoise	-0.343525152	3.42E-08	-0.50324946	3.91E-17
CLUL1	turquoise	-0.223868631	0.000413835	-0.305146587	1.12E-06
CMAS	turquoise	-0.34951019	1.90E-08	-0.680876204	1.02E-34
CMTM3	turquoise	0.179520218	0.004824875	0.403866046	4.99E-11
CNOT7	turquoise	-0.22718126	0.000337402	-0.698300355	3.78E-37
CNPY4	turquoise	0.429842088	1.94E-12	0.487519127	4.96E-16
CNRIP1	turquoise	-0.322263188	2.51E-07	-0.541653236	4.43E-20
CNTNAP5	turquoise	-0.295768521	2.46E-06	-0.260090764	3.77E-05
COBLL1	turquoise	0.336470352	6.74E-08	0.395397074	1.36E-10
COL18A1	turquoise	0.185614475	0.003547225	0.427952901	2.49E-12
COL24A1	turquoise	-0.151203518	0.017873037	-0.414635272	1.34E-11
COL5A2	turquoise	-0.128383951	0.044689515	-0.243110383	0.000121222
COPS2	turquoise	-0.190176119	0.002800495	-0.231572624	0.000256213
COPS3	turquoise	-0.304833562	1.15E-06	-0.543658275	3.03E-20
COPS4	turquoise	-0.306312803	1.02E-06	-0.682201755	6.74E-35
COPS5	turquoise	-0.265696923	2.51E-05	-0.561591862	9.17E-22
COPS8	turquoise	-0.339621054	4.99E-08	-0.519176786	2.60E-18
COX5A	turquoise	-0.321788625	2.62E-07	-0.669155428	3.54E-33
COX6B1	turquoise	-0.391401614	2.15E-10	-0.637767854	2.24E-29
COX6C	turquoise	-0.159471396	0.012441353	-0.465378946	1.43E-14
COX7A2L	turquoise	-0.355702644	1.02E-08	-0.577250088	3.61E-23
CP	turquoise	0.239536735	0.000153446	0.091904326	0.151512197
CPM	turquoise	0.230216164	0.000279107	0.337894898	5.89E-08
CPNE4	turquoise	-0.271352308	1.66E-05	-0.432753561	1.33E-12
CRB1	turquoise	0.201397607	0.001530793	0.369559016	2.41E-09
CRBN	turquoise	-0.422055165	5.30E-12	-0.633804067	6.30E-29
CREBBP	turquoise	0.288639319	4.37E-06	0.353321489	1.30E-08
CREG2	turquoise	-0.310105265	7.36E-07	-0.480075023	1.58E-15
CRH	turquoise	-0.287814638	4.67E-06	-0.380533582	7.32E-10
CRHBP	turquoise	-0.257727442	4.45E-05	-0.369643334	2.39E-09
CRISPLD2	turquoise	0.273437338	1.42E-05	0.274448175	1.31E-05
CRTAP	turquoise	0.42179146	5.48E-12	0.364117395	4.28E-09
CRYAB	turquoise	0.169993673	0.007661119	0.25809171	4.34E-05
CRYM	turquoise	-0.428220629	2.40E-12	-0.602356556	1.39E-25

CSF1R	turquoise	0.319195968	3.31E-07	0.362671454	4.98E-09
CSF3R	turquoise	0.281662846	7.56E-06	0.350207243	1.77E-08
CSK	turquoise	0.278125026	9.94E-06	0.31212531	6.17E-07
CSMD3	turquoise	-0.18517172	0.003628533	-0.385229711	4.33E-10
CSPG4	turquoise	0.249496518	7.88E-05	0.658332907	8.14E-32
CSRNP2	turquoise	-0.184806881	0.003696793	-0.67453906	7.07E-34
CSRP1	turquoise	0.290127939	3.88E-06	0.303909343	1.25E-06
CTDSP1	turquoise	0.312149843	6.16E-07	0.48964155	3.55E-16
CTDSP2	turquoise	0.432757795	1.33E-12	0.714587762	1.39E-39
CTDSPL	turquoise	0.26832893	2.07E-05	0.688704391	8.65E-36
CTNS	turquoise	0.296038114	2.40E-06	0.52357892	1.20E-18
CTSH	turquoise	0.342616923	3.74E-08	0.367134259	3.12E-09
CUEDC1	turquoise	0.335317608	7.52E-08	0.680370235	1.19E-34
CXADR	turquoise	-0.198637806	0.001781278	-0.441571083	4.09E-13
CXCL16	turquoise	0.314897656	4.85E-07	0.387842058	3.23E-10
CYP26B1	turquoise	-0.289048614	4.23E-06	-0.582588387	1.15E-23
CYP2C8	turquoise	-0.259012242	4.07E-05	-0.280918507	8.01E-06
CYP4X1	turquoise	-0.408552878	2.84E-11	-0.523979155	1.12E-18
CYYR1	turquoise	0.19693527	0.001953928	0.292927663	3.10E-06
DAB2	turquoise	0.400185802	7.73E-11	0.416651269	1.05E-11
DACH2	turquoise	-0.296019606	2.41E-06	-0.592350791	1.35E-24
DAPK1	turquoise	-0.127325061	0.046495131	-0.552872541	5.16E-21
DCK	turquoise	-0.293896114	2.86E-06	-0.752390866	5.95E-46
DCLK1	turquoise	-0.349006356	2.00E-08	-0.686789323	1.59E-35
DCN	turquoise	0.452112256	9.57E-14	0.386125443	3.92E-10
DCTN2	turquoise	-0.290153452	3.87E-06	-0.493421566	1.94E-16
DCTN3	turquoise	-0.199119052	0.001735068	-0.63165129	1.10E-28
DCTN6	turquoise	-0.288221962	4.52E-06	-0.448842539	1.51E-13
DCUN1D5	turquoise	-0.382200521	6.08E-10	-0.604809595	7.85E-26
DDAH2	turquoise	0.298451522	1.97E-06	0.398832492	9.07E-11
DDIT4	turquoise	0.425632636	3.35E-12	0.566868872	3.14E-22
DDR1	turquoise	0.089963798	0.160377551	0.17714838	0.005424912
DDR2	turquoise	0.327961614	1.50E-07	0.461252837	2.61E-14
DDX1	turquoise	-0.30799868	8.82E-07	-0.684443864	3.34E-35
DDX10	turquoise	-0.322758425	2.40E-07	-0.560946012	1.04E-21
DDX25	turquoise	-0.189897044	0.002841716	-0.435195811	9.62E-13
DHCR24	turquoise	-0.264171943	2.81E-05	-0.319831472	3.13E-07
DHRS3	turquoise	0.368555093	2.68E-09	0.480623574	1.45E-15
DHRS7	turquoise	-0.111276315	0.082167699	-0.444815065	2.63E-13
DHRS7B	turquoise	-0.316167846	4.33E-07	-0.164648337	0.009833862

DHX34	turquoise	0.242913217	0.00012282	0.569631761	1.78E-22
DHX37	turquoise	0.183759534	0.003899243	0.502315995	4.56E-17
DIRAS2	turquoise	-0.346355137	2.59E-08	-0.664502542	1.38E-32
DLC1	turquoise	0.333821526	8.66E-08	0.671369982	1.83E-33
DLD	turquoise	-0.319620896	3.19E-07	-0.650148616	8.02E-31
DLG3	turquoise	-0.242746716	0.000124185	-0.237793628	0.000171923
DLX1	turquoise	-0.219928138	0.000525588	-0.547238732	1.53E-20
DMXL2	turquoise	-0.185595141	0.003550741	-0.683683109	4.24E-35
DNAJA2	turquoise	-0.344466247	3.12E-08	-0.384083822	4.93E-10
DNAJA4	turquoise	-0.127904174	0.045500212	-0.364087242	4.29E-09
DNAJB2	turquoise	0.193361867	0.002366929	0.542706355	3.63E-20
DNAJB9	turquoise	-0.423967547	4.15E-12	-0.447829004	1.74E-13
DNAJC12	turquoise	-0.221412573	0.00048056	-0.587128923	4.29E-24
DNAJC19	turquoise	-0.281890482	7.43E-06	-0.452168144	9.49E-14
DNER	turquoise	-0.290341564	3.81E-06	-0.580049145	1.99E-23
DNM1L	turquoise	-0.258370034	4.26E-05	-0.664223616	1.50E-32
DNM3	turquoise	-0.451320106	1.07E-13	-0.673007238	1.12E-33
DOCK1	turquoise	0.363037948	4.79E-09	0.449467599	1.38E-13
DOCK3	turquoise	-0.250826182	7.20E-05	-0.768506771	4.98E-49
DOCK6	turquoise	0.319077088	3.35E-07	0.379715241	8.01E-10
DPP10	turquoise	-0.193751118	0.00231837	-0.49738776	1.02E-16
DPP8	turquoise	-0.233881027	0.000221231	-0.744569136	1.53E-44
DPP9	turquoise	0.136911333	0.032182279	0.355077919	1.09E-08
DPY30	turquoise	-0.243705981	0.000116511	-0.608012257	3.70E-26
DRG1	turquoise	-0.255341163	5.27E-05	-0.611529464	1.61E-26
DSE	turquoise	0.464369536	1.66E-14	0.207789643	0.001069519
DST	turquoise	0.400137245	7.77E-11	0.156347516	0.014293764
DSTN	turquoise	-0.363083002	4.77E-09	-0.57348427	7.99E-23
DTNA	turquoise	0.22912201	0.000298947	0.348787703	2.04E-08
DUSP1	turquoise	0.322218654	2.52E-07	0.125901684	0.049018065
DYNC1I1	turquoise	-0.391424887	2.14E-10	-0.762851487	6.39E-48
DYNLT3	turquoise	-0.34281688	3.67E-08	-0.733733664	1.13E-42
EAPP	turquoise	-0.181152565	0.004447313	-0.373163841	1.64E-09
EBNA1BP2	turquoise	-0.333990001	8.53E-08	-0.616012581	5.46E-27
ECHDC2	turquoise	0.282246436	7.23E-06	0.638124996	2.04E-29
EDN1	turquoise	0.15977492	0.012273162	0.297937611	2.06E-06
EEF1D	turquoise	0.18480818	0.003696548	0.281247938	7.81E-06
EEF1E1	turquoise	-0.31493535	4.83E-07	-0.561752621	8.87E-22
EEF2K	turquoise	0.370676148	2.14E-09	0.508169035	1.72E-17
EFCAB7	turquoise	-0.209357547	0.000977875	-0.424267109	4.00E-12

EFNA1	turquoise	0.223467092	0.00042412	0.393883519	1.62E-10
EFNB3	turquoise	-0.106867983	0.09512197	-0.209010192	0.000997531
EGR1	turquoise	-0.238005326	0.000169573	-0.269555327	1.89E-05
EHD1	turquoise	0.217124906	0.000621447	0.694542965	1.31E-36
EHD2	turquoise	0.394040457	1.59E-10	0.517238171	3.65E-18
EHD3	turquoise	-0.373903961	1.51E-09	-0.48454513	7.90E-16
EID2	turquoise	-0.345600998	2.79E-08	-0.71320871	2.27E-39
EIF1B	turquoise	-0.28854899	4.40E-06	-0.733978934	1.03E-42
EIF2B3	turquoise	-0.311500721	6.52E-07	-0.263070018	3.04E-05
EIF4A2	turquoise	-0.382881276	5.64E-10	-0.595666265	6.41E-25
EIF4E3	turquoise	-0.228898347	0.000303161	-0.687901685	1.12E-35
EIF4H	turquoise	-0.392698646	1.85E-10	-0.718270698	3.71E-40
ELAVL2	turquoise	-0.249628773	7.81E-05	-0.516251122	4.33E-18
ELAVL4	turquoise	-0.293155885	3.04E-06	-0.501561542	5.16E-17
ELF2	turquoise	0.362442386	5.10E-09	0.524925238	9.45E-19
ELK1	turquoise	0.366772472	3.24E-09	0.48100789	1.37E-15
ELMO1	turquoise	-0.33962481	4.99E-08	-0.338793467	5.40E-08
ELMOD1	turquoise	-0.28601236	5.38E-06	-0.717315263	5.23E-40
ELOVL4	turquoise	-0.363998253	4.33E-09	-0.481139923	1.34E-15
EML3	turquoise	0.326602723	1.69E-07	0.666799408	7.08E-33
EMP1	turquoise	0.295545273	2.50E-06	0.145923959	0.02233435
EMP3	turquoise	0.26181765	3.33E-05	0.0227198	0.723452697
ENAH	turquoise	0.491558105	2.61E-16	0.601702105	1.62E-25
ENC1	turquoise	-0.328074069	1.48E-07	-0.492254929	2.34E-16
ENHO	turquoise	0.096469954	0.13212223	0.396544992	1.19E-10
ENO3	turquoise	0.273750874	1.38E-05	0.526158092	7.58E-19
ENOPH1	turquoise	-0.248956091	8.18E-05	-0.603166749	1.15E-25
ENPP5	turquoise	-0.343618784	3.39E-08	-0.384372907	4.77E-10
ENTPD2	turquoise	0.27517597	1.24E-05	0.231170625	0.000262809
ENTPD3	turquoise	-0.19497837	0.002171146	-0.581272624	1.53E-23
EPAS1	turquoise	0.263616898	2.92E-05	0.266495349	2.37E-05
EPB41L3	turquoise	-0.35913256	7.18E-09	-0.764488256	3.07E-48
EPB41L4B	turquoise	-0.20777047	0.001070687	-0.39052625	2.38E-10
EPHA3	turquoise	-0.145726951	0.022517973	-0.311390089	6.58E-07
EPHA4	turquoise	-0.304346991	1.20E-06	-0.642891637	5.75E-30
EPHA5	turquoise	-0.203318218	0.001375972	-0.328958538	1.36E-07
EPM2AIP1	turquoise	-0.35500174	1.09E-08	-0.505708418	2.60E-17
EPS15	turquoise	-0.271824883	1.60E-05	-0.480202812	1.55E-15
ERC2	turquoise	-0.265425771	2.56E-05	-0.357775983	8.25E-09
ESAM	turquoise	0.304108832	1.23E-06	0.342499184	3.78E-08

ETS1	turquoise	0.343307889	3.49E-08	0.444473224	2.76E-13
ETS2	turquoise	-0.109454081	0.087338769	-0.218942892	0.000557598
EXOC6	turquoise	-0.260080307	3.77E-05	-0.505270381	2.79E-17
EXOSC9	turquoise	-0.245122846	0.000105986	-0.328869908	1.37E-07
EXTL2	turquoise	-0.238085921	0.000168686	-0.499820842	6.87E-17
FABP3	turquoise	-0.247734382	8.89E-05	-0.35978629	6.71E-09
FAM131A	turquoise	-0.275550448	1.21E-05	-0.224730502	0.000392535
FAM162A	turquoise	-0.226209279	0.000358345	-0.086749721	0.175909792
FAM174A	turquoise	-0.310884441	6.88E-07	-0.652039172	4.76E-31
FAM181B	turquoise	0.260981374	3.53E-05	0.279966509	8.63E-06
FAM49B	turquoise	-0.265874157	2.48E-05	-0.384600304	4.65E-10
FAM71E1	turquoise	-0.212262451	0.000826917	-0.241012866	0.000139269
FAM89A	turquoise	0.280811009	8.08E-06	0.181395231	0.004393516
FANCB	turquoise	0.450715407	1.16E-13	0.494125606	1.73E-16
FAR2	turquoise	-0.11560777	0.070862903	-0.393119609	1.76E-10
FBL	turquoise	0.319876126	3.12E-07	0.47575527	3.05E-15
FBLN1	turquoise	0.219142787	0.000550962	0.35404904	1.20E-08
FBXL2	turquoise	-0.321865032	2.61E-07	-0.555288945	3.21E-21
FBXL7	turquoise	0.262517812	3.17E-05	0.494045723	1.76E-16
FBXO28	turquoise	-0.260778503	3.59E-05	-0.253802953	5.86E-05
FBXO34	turquoise	-0.28438056	6.12E-06	-0.588453737	3.21E-24
FBXW4	turquoise	0.428397065	2.35E-12	0.742506815	3.53E-44
FBXW7	turquoise	-0.407499349	3.22E-11	-0.431293518	1.61E-12
FCGBP	turquoise	0.188444439	0.0030653	0.24349023	0.000118197
FCGRT	turquoise	0.263089342	3.04E-05	0.24700823	9.34E-05
FGD2	turquoise	0.178290384	0.005128115	0.432115131	1.44E-12
FGF12	turquoise	-0.425906836	3.24E-12	-0.530397216	3.54E-19
FGF14	turquoise	-0.238119393	0.000168319	-0.407350062	3.28E-11
FGF9	turquoise	-0.292514525	3.20E-06	-0.118615936	0.063783871
FGFRL1	turquoise	0.277763535	1.02E-05	0.376467801	1.14E-09
FGL1	turquoise	0.138282301	0.030479926	0.392083633	1.99E-10
FGR	turquoise	0.238326729	0.000166062	0.585227966	6.50E-24
FH	turquoise	-0.316096057	4.36E-07	-0.311971943	6.26E-07
FHL2	turquoise	-0.207225268	0.001104394	-0.382918723	5.61E-10
FIG4	turquoise	-0.232965579	0.000234533	-0.402116506	6.15E-11
FKBP1B	turquoise	-0.308163243	8.69E-07	-0.546727453	1.69E-20
FKBP5	turquoise	0.259817751	3.84E-05	0.311473652	6.53E-07
FLI1	turquoise	0.275646061	1.20E-05	0.316995467	4.03E-07
FLRT3	turquoise	-0.150837918	0.018154826	-0.343860741	3.31E-08
FLT1	turquoise	0.294464011	2.73E-06	0.076994691	0.229837489

FMNL3	turquoise	0.260565936	3.64E-05	0.266933308	2.30E-05
FOXC1	turquoise	0.376550504	1.13E-09	0.712344467	3.08E-39
FOXD1	turquoise	0.327453018	1.57E-07	0.454035571	7.30E-14
FOXJ1	turquoise	0.425370803	3.47E-12	0.452055997	9.64E-14
FOXO4	turquoise	0.356763478	9.15E-09	0.584568819	7.50E-24
FOXQ1	turquoise	0.318770566	3.44E-07	0.338570649	5.52E-08
FRMPD2	turquoise	-0.314429671	5.05E-07	-0.247117911	9.27E-05
FSIP1	turquoise	-0.088700235	0.166356163	-0.228582005	0.000309216
FSTL5	turquoise	-0.312174623	6.15E-07	-0.49643742	1.19E-16
FTO	turquoise	-0.33110814	1.12E-07	-0.632385387	9.08E-29
FXYD5	turquoise	0.315561479	4.57E-07	0.524156064	1.08E-18
FXYD6	turquoise	-0.313840304	5.32E-07	-0.467606056	1.03E-14
FYCO1	turquoise	0.438743964	5.99E-13	0.5859158	5.60E-24
FZD9	turquoise	0.392220799	1.96E-10	0.61090427	1.86E-26
G3BP2	turquoise	-0.272446425	1.53E-05	-0.556966724	2.31E-21
GABARAPL1	turquoise	-0.406650444	3.57E-11	-0.770394118	2.09E-49
GABARAPL2	turquoise	-0.247440777	9.07E-05	-0.761827495	1.01E-47
GABBR2	turquoise	-0.397624221	1.04E-10	-0.625516885	5.21E-28
GABRA1	turquoise	-0.385026661	4.43E-10	-0.59469477	7.98E-25
GABRA5	turquoise	-0.22956145	0.000290826	-0.660846791	3.98E-32
GABRG2	turquoise	-0.418213688	8.60E-12	-0.508282625	1.69E-17
GAD1	turquoise	-0.368532502	2.69E-09	-0.608749751	3.11E-26
GAD2	turquoise	-0.341404908	4.20E-08	-0.547124508	1.57E-20
GADD45G	turquoise	0.345877352	2.72E-08	0.536637476	1.13E-19
GAL3ST4	turquoise	0.310482654	7.12E-07	0.49553603	1.38E-16
GALNTL5	turquoise	-0.176084911	0.005715062	-0.232635141	0.000239516
GAS1	turquoise	0.378326837	9.33E-10	0.264816063	2.68E-05
GAS2L3	turquoise	0.375169825	1.32E-09	0.361922302	5.38E-09
GATA2	turquoise	0.235219421	0.000203044	0.136008556	0.033346948
GATAD2A	turquoise	0.259991039	3.79E-05	0.61864802	2.87E-27
GBP2	turquoise	0.051604167	0.421317208	0.190866462	0.002700849
GCNT2	turquoise	0.160279856	0.011997797	0.282363153	7.16E-06
GDAP1	turquoise	-0.3088522	8.20E-07	-0.419747498	7.09E-12
GDPD3	turquoise	0.16013643	0.012075455	0.405215951	4.24E-11
GEM	turquoise	0.482243246	1.13E-15	0.244988652	0.000106943
GFAP	turquoise	0.41220804	1.81E-11	0.354824645	1.11E-08
GGCT	turquoise	-0.319971777	3.09E-07	-0.631706072	1.08E-28
GIMAP1	turquoise	0.156407016	0.014256338	0.319037837	3.36E-07
GIMAP7	turquoise	0.242811031	0.000123656	0.466623472	1.19E-14
GIMAP8	turquoise	0.318290859	3.59E-07	0.438787779	5.95E-13

GJA4	turquoise	0.318646753	3.48E-07	0.410923729	2.12E-11
GLCE	turquoise	-0.232851141	0.000236248	-0.38315939	5.46E-10
GLMN	turquoise	-0.234115642	0.000217936	-0.345551669	2.81E-08
GLRA2	turquoise	-0.260627817	3.62E-05	-0.436978168	7.59E-13
GLRB	turquoise	-0.340647565	4.52E-08	-0.676823555	3.53E-34
GLRX	turquoise	-0.277829941	1.02E-05	-0.599432275	2.72E-25
GLRX2	turquoise	-0.176566764	0.005581916	-0.205472468	0.00121948
GLS	turquoise	-0.301414616	1.54E-06	-0.421516859	5.67E-12
GLS2	turquoise	-0.182272907	0.004203801	-0.397866714	1.02E-10
GMPR	turquoise	0.385132315	4.38E-10	0.338411447	5.60E-08
GNA11	turquoise	0.134907346	0.034815959	0.46360951	1.85E-14
GNAS	turquoise	-0.186015455	0.003475015	-0.61914174	2.54E-27
GNB5	turquoise	-0.156200968	0.014386313	-0.339697981	4.95E-08
GNG11	turquoise	0.319176228	3.32E-07	0.296487692	2.32E-06
GNG2	turquoise	-0.290814832	3.67E-06	-0.704857631	4.15E-38
GNG3	turquoise	-0.356987254	8.94E-09	-0.236633626	0.00018535
GNPDA2	turquoise	-0.240036903	0.000148498	-0.385845716	4.04E-10
GOLIM4	turquoise	0.334358169	8.24E-08	0.594333222	8.66E-25
GOLT1B	turquoise	-0.289433481	4.10E-06	-0.492260473	2.34E-16
GOT1	turquoise	-0.402962659	5.56E-11	-0.695246904	1.04E-36
GOT2	turquoise	-0.310300844	7.23E-07	-0.602455514	1.36E-25
GPM6A	turquoise	-0.331481568	1.08E-07	-0.588857913	2.93E-24
GPN1	turquoise	-0.264294407	2.78E-05	-0.450684598	1.17E-13
GPR158	turquoise	-0.283020623	6.81E-06	-0.051533571	0.421953593
GPR19	turquoise	-0.176128706	0.005702844	-0.362691233	4.97E-09
GPR22	turquoise	-0.19433695	0.002246994	-0.508874412	1.53E-17
GPR4	turquoise	0.429385384	2.06E-12	0.405353932	4.17E-11
GPR83	turquoise	-0.104188358	0.103759109	-0.139735292	0.028760229
GPR88	turquoise	-0.198314841	0.001812917	-0.480946452	1.38E-15
GPRASP1	turquoise	-0.343340584	3.48E-08	-0.365392654	3.74E-09
GPRC5C	turquoise	0.253310357	6.07E-05	0.584845598	7.07E-24
GPT	turquoise	0.361054388	5.88E-09	0.507310236	1.98E-17
GRB14	turquoise	-0.247100782	9.28E-05	-0.307332726	9.34E-07
GRIA1	turquoise	-0.181482215	0.004374375	-0.156027374	0.01449662
GRIA2	turquoise	-0.323912411	2.17E-07	-0.413423726	1.56E-11
GRIA4	turquoise	-0.266445419	2.38E-05	-0.488081068	4.54E-16
GRIN2A	turquoise	-0.379646539	8.07E-10	-0.648200299	1.37E-30
GRIN2C	turquoise	0.148243186	0.020268124	0.582965025	1.06E-23
GRM1	turquoise	-0.227711578	0.000326461	-0.326519731	1.71E-07
GRM8	turquoise	-0.150226843	0.018634452	-0.345572449	2.80E-08

GSDMD	turquoise	0.360057749	6.52E-09	0.669167131	3.53E-33
GSN	turquoise	0.245608577	0.000102588	0.232583891	0.000240297
GSTM2	turquoise	0.115681179	0.070682832	0.457127936	4.71E-14
GSTO1	turquoise	-0.176747781	0.00553262	-0.226763051	0.000346269
GYG2	turquoise	0.311206775	6.69E-07	0.284553827	6.04E-06
GYPC	turquoise	0.209230938	0.000984998	0.29277203	3.14E-06
HABP4	turquoise	-0.321388036	2.72E-07	-0.473119422	4.54E-15
HACL1	turquoise	-0.284537383	6.04E-06	-0.391637721	2.09E-10
HADHA	turquoise	0.230648903	0.000271604	0.416810975	1.03E-11
HAPLN4	turquoise	-0.110924845	0.083145404	-0.123696125	0.053152297
HAVCR2	turquoise	0.295381077	2.54E-06	0.33573227	7.23E-08
HBP1	turquoise	0.447150861	1.91E-13	0.569324086	1.90E-22
HBQ1	turquoise	-0.208531105	0.001025239	-0.196689956	0.001980026
HCCS	turquoise	-0.310232252	7.28E-07	-0.613595134	9.79E-27
HCG9	turquoise	0.060289474	0.347367043	0.625488794	5.25E-28
HCLS1	turquoise	0.187774365	0.003173714	0.059241494	0.355827371
HERC1	turquoise	-0.363454921	4.59E-09	-0.728670947	7.88E-42
HERC5	turquoise	0.253057675	6.17E-05	0.491278066	2.73E-16
HES6	turquoise	0.258755967	4.14E-05	0.562758513	7.25E-22
HEY2	turquoise	0.434959543	9.92E-13	0.681466061	8.47E-35
HEYL	turquoise	0.406181095	3.78E-11	0.419636301	7.19E-12
HIF3A	turquoise	-0.035338222	0.581994379	0.226162178	0.000359389
HINT1	turquoise	-0.333361862	9.05E-08	-0.821038436	4.29E-61
HK1	turquoise	-0.328755295	1.39E-07	-0.501532851	5.19E-17
HLA-DMA	turquoise	0.292717545	3.15E-06	0.346814302	2.48E-08
HLA-DMB	turquoise	0.285756524	5.49E-06	0.237730063	0.000172634
HLA-E	turquoise	0.332501752	9.81E-08	0.322477521	2.47E-07
HLF	turquoise	-0.150298793	0.018577412	-0.544790975	2.45E-20
HMBOX1	turquoise	0.468405112	9.16E-15	0.418277465	8.53E-12
HMGB2	turquoise	0.11036041	0.084735108	0.452334275	9.28E-14
HMGCLL1	turquoise	-0.284101246	6.26E-06	-0.614679939	7.53E-27
HMGCS1	turquoise	-0.241177284	0.000137768	-0.361673814	5.52E-09
HNRNPA0	turquoise	-0.272319783	1.54E-05	-0.67754575	2.83E-34
HOMER1	turquoise	-0.284114771	6.25E-06	-0.471270286	5.98E-15
HOMER3	turquoise	0.395871286	1.28E-10	0.443958016	2.96E-13
HOPX	turquoise	-0.44542529	2.42E-13	-0.645924585	2.54E-30
HPCAL1	turquoise	-0.483384497	9.46E-16	-0.357919801	8.13E-09
HPCAL4	turquoise	-0.308746105	8.27E-07	-0.514316993	6.04E-18
HPRT1	turquoise	-0.42616997	3.13E-12	-0.723847593	4.80E-41
HS3ST2	turquoise	-0.241928299	0.000131103	-0.231435053	0.000258453

HS6ST2	turquoise	-0.315844617	4.46E-07	-0.468147387	9.51E-15
HSP90AB1	turquoise	-0.277921504	1.01E-05	-0.445073914	2.54E-13
HSPA12A	turquoise	-0.233033775	0.000233517	-0.338073381	5.79E-08
HSPA12B	turquoise	0.015200228	0.812875458	0.258720407	4.15E-05
HSPA13	turquoise	-0.250816406	7.20E-05	-0.075280696	0.24040711
HSPA1A	turquoise	0.148884199	0.019727128	0.411676284	1.94E-11
HSPA8	turquoise	-0.217679346	0.000601292	-0.525266445	8.89E-19
HSPB1	turquoise	0.292721125	3.15E-06	0.461897035	2.37E-14
HSPB2	turquoise	0.458960633	3.62E-14	0.464470257	1.63E-14
HSPB3	turquoise	-0.403538629	5.19E-11	-0.388227785	3.09E-10
HSPH1	turquoise	-0.198363963	0.001808072	-0.249501682	7.88E-05
HVCN1	turquoise	0.162208672	0.010995384	0.254894309	5.43E-05
HYAL1	turquoise	0.297615014	2.11E-06	0.521855658	1.63E-18
ICAM2	turquoise	0.361093226	5.86E-09	0.312125828	6.17E-07
ID1	turquoise	0.229241042	0.000296727	0.219794642	0.000529823
ID3	turquoise	0.313323602	5.56E-07	0.291388388	3.51E-06
IDH3A	turquoise	-0.271160917	1.68E-05	-0.528374887	5.10E-19
IDI1	turquoise	-0.175872835	0.00577456	-0.351049067	1.63E-08
IER3	turquoise	0.231653761	0.000254901	0.257162373	4.63E-05
IFI16	turquoise	0.225613898	0.000371763	0.133677137	0.036521644
IFI27L2	turquoise	0.326151142	1.77E-07	0.416383309	1.08E-11
IFIT1	turquoise	-0.206729615	0.001135882	-0.466020382	1.30E-14
IFITM1	turquoise	0.326085845	1.78E-07	0.383067032	5.52E-10
IFITM2	turquoise	0.177064931	0.005447193	0.297710703	2.09E-06
IFITM3	turquoise	0.17327884	0.006548542	0.252488178	6.42E-05
IGDCC3	turquoise	0.138231935	0.030541079	0.605771609	6.27E-26
IGF1	turquoise	-0.208620445	0.001020019	-0.283722872	6.44E-06
IGF2BP2	turquoise	0.284525347	6.05E-06	0.498753053	8.19E-17
IGFBP7	turquoise	0.163169759	0.010524085	0.066199914	0.302059973
IGSF3	turquoise	-0.18753491	0.003213291	-0.372626867	1.73E-09
IL10RB	turquoise	0.231540722	0.000256731	0.558109633	1.84E-21
IL1R1	turquoise	0.281052534	7.93E-06	0.172538175	0.006785933
IL4R	turquoise	0.330995609	1.13E-07	0.490092356	3.30E-16
ILK	turquoise	0.452898559	8.57E-14	0.480005952	1.59E-15
IMP3	turquoise	-0.285706869	5.51E-06	-0.464661323	1.59E-14
IMPA1	turquoise	-0.205406954	0.001223988	-0.265876926	2.48E-05
IMPAD1	turquoise	-0.236011275	0.000192951	-0.398058416	9.93E-11
INA	turquoise	-0.347018823	2.43E-08	-0.59061437	1.99E-24
INHBB	turquoise	0.354289112	1.18E-08	0.498802789	8.12E-17
INPP5D	turquoise	0.264387797	2.77E-05	0.416427875	1.08E-11

INSIG2	turquoise	-0.264152577	2.81E-05	-0.475269016	3.28E-15
INTS1	turquoise	0.24465326	0.000109371	0.686321881	1.85E-35
IPO5	turquoise	-0.211487706	0.000864924	-0.620370717	1.88E-27
IRF7	turquoise	0.196749264	0.001973688	0.576080116	4.63E-23
ISCA1	turquoise	-0.373035603	1.66E-09	-0.651779493	5.11E-31
ISCU	turquoise	-0.326095259	1.77E-07	-0.717778668	4.43E-40
ISG20	turquoise	0.210503925	0.000915504	0.404500698	4.63E-11
ISYNA1	turquoise	0.246661766	9.56E-05	0.347540808	2.31E-08
ITFG1	turquoise	-0.331835676	1.04E-07	-0.825526385	2.63E-62
ITGA10	turquoise	0.479049013	1.85E-15	0.524406823	1.04E-18
ITGA5	turquoise	0.253585128	5.95E-05	0.300184209	1.71E-06
ITGA8	turquoise	0.317674733	3.79E-07	0.439111077	5.70E-13
ITGB1	turquoise	0.438351686	6.31E-13	0.557936911	1.90E-21
ITGB1BP1	turquoise	-0.281718651	7.53E-06	-0.478677662	1.95E-15
ITGB4	turquoise	0.226844799	0.000344519	0.079589004	0.21447124
ITPKB	turquoise	0.480502401	1.48E-15	0.529433321	4.21E-19
ITPR1	turquoise	-0.214174571	0.000739608	-0.640085137	1.21E-29
ITPR3	turquoise	0.30673938	9.82E-07	0.669012606	3.69E-33
ITPRIP	turquoise	0.424378562	3.94E-12	0.563527685	6.20E-22
JAG1	turquoise	0.286340759	5.24E-06	0.460723754	2.81E-14
JAZF1	turquoise	-0.228106268	0.000318533	-0.684937601	2.86E-35
KANK1	turquoise	0.221434918	0.000479911	0.29794888	2.05E-06
KANK2	turquoise	0.434232501	1.09E-12	0.355649528	1.02E-08
KCNA1	turquoise	-0.172601655	0.00676529	-0.175261967	0.005949054
KCNAB1	turquoise	-0.420731594	6.26E-12	-0.49772388	9.68E-17
KCNB2	turquoise	-0.264087629	2.83E-05	-0.35181043	1.51E-08
KCND2	turquoise	-0.337006489	6.41E-08	-0.353860776	1.23E-08
KCNE4	turquoise	0.389625194	2.63E-10	0.265409105	2.57E-05
KCNIP4	turquoise	-0.282083231	7.32E-06	-0.578801435	2.60E-23
KCNJ8	turquoise	0.322671698	2.42E-07	0.361405586	5.67E-09
KCNK1	turquoise	-0.273321378	1.43E-05	-0.51820101	3.08E-18
KCNN3	turquoise	0.351563073	1.55E-08	0.491968578	2.45E-16
KCNV1	turquoise	-0.355156372	1.08E-08	-0.543406603	3.18E-20
KCTD11	turquoise	0.137504345	0.031436238	0.321297843	2.74E-07
KIAA0513	turquoise	-0.430810446	1.71E-12	-0.603716284	1.01E-25
KIF1C	turquoise	0.288079808	4.57E-06	0.493493819	1.92E-16
KIF3A	turquoise	-0.288810336	4.31E-06	-0.259130473	4.03E-05
KIF5B	turquoise	0.373745581	1.54E-09	0.281608756	7.60E-06
KIF6	turquoise	0.215307133	0.00069199	0.168190466	0.008340831
KIFAP3	turquoise	-0.418260305	8.55E-12	-0.555974399	2.81E-21

KIT	turquoise	-0.224274215	0.000403681	-0.349689653	1.87E-08
KITLG	turquoise	-0.205711299	0.001203176	-0.377322946	1.04E-09
KLC1	turquoise	-0.461932724	2.36E-14	-0.462180113	2.28E-14
KLF11	turquoise	0.189335812	0.002926285	0.466967349	1.13E-14
KLF2	turquoise	0.373788629	1.53E-09	0.529597039	4.09E-19
KLF4	turquoise	0.264919715	2.66E-05	0.177458605	0.0053428
KLF6	turquoise	0.321317983	2.74E-07	0.385392804	4.25E-10
KLHDC2	turquoise	-0.1187091	0.063574257	-0.308859433	8.19E-07
KLHL5	turquoise	0.248790649	8.27E-05	0.25570797	5.13E-05
KLHL7	turquoise	-0.110873739	0.083288346	-0.59699525	4.74E-25
KLK7	turquoise	-0.265707908	2.51E-05	-0.304617717	1.18E-06
KPNA1	turquoise	-0.339676445	4.96E-08	-0.650913311	6.50E-31
KRCC1	turquoise	0.3263987	1.73E-07	0.69784509	4.40E-37
KTN1	turquoise	0.290459399	3.78E-06	0.269820792	1.86E-05
LAMA5	turquoise	0.312610828	5.92E-07	0.615801869	5.74E-27
LAMC1	turquoise	0.403116246	5.46E-11	0.555408199	3.14E-21
LANCL1	turquoise	-0.244926448	0.00010739	-0.549518434	9.89E-21
LANCL2	turquoise	-0.240109086	0.000147796	-0.417933029	8.91E-12
LAPTM4B	turquoise	-0.188462613	0.003062407	-0.527353401	6.12E-19
LAT	turquoise	0.199206288	0.001726809	0.584718908	7.26E-24
LATS2	turquoise	0.215173392	0.000697462	0.261231551	3.47E-05
LCAT	turquoise	0.240150439	0.000147395	0.480991461	1.37E-15
LCP1	turquoise	0.317231535	3.94E-07	0.262503502	3.17E-05
LDHA	turquoise	-0.311457239	6.54E-07	-0.74474239	1.42E-44
LDLRAP1	turquoise	0.182500629	0.004155799	0.23449233	0.000212743
LEAP2	turquoise	0.295432903	2.53E-06	0.325912678	1.80E-07
LEF1	turquoise	0.381349516	6.68E-10	0.462852705	2.07E-14
LEPR	turquoise	0.154790654	0.015304144	0.341851513	4.03E-08
LFNG	turquoise	0.142172825	0.026061641	0.515489132	4.93E-18
LIME1	turquoise	0.200273162	0.001628677	0.720151026	1.87E-40
LIMS2	turquoise	0.13868618	0.029993323	0.452252396	9.38E-14
LIN7B	turquoise	-0.317663487	3.80E-07	-0.488147478	4.49E-16
LMBR1	turquoise	-0.228967383	0.000301855	-0.66576182	9.59E-33
LNX1	turquoise	-0.248022658	8.72E-05	-0.47513966	3.35E-15
LONRF2	turquoise	-0.280467551	8.30E-06	-0.363900887	4.38E-09
LPAR4	turquoise	0.216932023	0.000628604	0.302693556	1.38E-06
LPP	turquoise	0.392526167	1.89E-10	0.618675088	2.85E-27
LRFN5	turquoise	-0.246788119	9.48E-05	-0.501186133	5.49E-17
LRMP	turquoise	-0.08316238	0.194531939	-0.297473436	2.14E-06
LRP10	turquoise	0.330081603	1.23E-07	0.426863705	2.86E-12

LRP11	turquoise	-0.274660662	1.29E-05	-0.722957033	6.68E-41
LRPAP1	turquoise	-0.242561628	0.000125719	-0.481959941	1.18E-15
LRPPRC	turquoise	-0.287947192	4.62E-06	-0.508175771	1.72E-17
LRRC32	turquoise	0.389468257	2.68E-10	0.577379789	3.52E-23
LRRC49	turquoise	-0.389955318	2.54E-10	-0.440410314	4.79E-13
LRRCC1	turquoise	0.229701503	0.000288282	0.319792395	3.14E-07
LRRTM1	turquoise	-0.23060598	0.00027234	-0.463807733	1.80E-14
LSM14A	turquoise	0.344636131	3.07E-08	0.549571041	9.79E-21
LSS	turquoise	0.232784894	0.000237246	0.321754327	2.63E-07
LYL1	turquoise	0.29139297	3.51E-06	0.674285151	7.63E-34
LZTFL1	turquoise	-0.314756775	4.91E-07	-0.537345413	9.90E-20
LZTS2	turquoise	0.11464444	0.073260783	0.301543268	1.52E-06
MACF1	turquoise	0.270270629	1.80E-05	0.418171538	8.65E-12
MAD2L1BP	turquoise	-0.252735619	6.31E-05	-0.366424435	3.36E-09
MAGED1	turquoise	-0.323702159	2.21E-07	-0.644900053	3.35E-30
MAGEE1	turquoise	-0.380187636	7.60E-10	-0.550696181	7.88E-21
MAGEH1	turquoise	-0.311249857	6.66E-07	-0.56679982	3.19E-22
MAK16	turquoise	-0.168178154	0.008345651	-0.325308153	1.91E-07
MAL2	turquoise	-0.349279277	1.94E-08	-0.664864061	1.25E-32
MAN1C1	turquoise	-0.115767606	0.070471304	-0.441948868	3.89E-13
MAP1B	turquoise	-0.213756732	0.000757926	-0.505732189	2.59E-17
MAP1LC3B	turquoise	-0.209393155	0.00097588	-0.501885829	4.90E-17
MAP2	turquoise	-0.175289114	0.0059412	-0.199920026	0.001660579
MAP2K1	turquoise	-0.296762254	2.26E-06	-0.751225517	9.73E-46
MAP2K4	turquoise	-0.259068797	4.05E-05	-0.69488275	1.17E-36
MAP3K11	turquoise	0.12005363	0.060611589	0.406161444	3.79E-11
MAP3K5	turquoise	0.344260117	3.18E-08	0.543520096	3.11E-20
MAP3K6	turquoise	0.376322553	1.16E-09	0.643306419	5.15E-30
MAP4	turquoise	-0.254364768	5.64E-05	-0.613580896	9.82E-27
MAPK1	turquoise	-0.249504043	7.88E-05	-0.338362603	5.63E-08
MAPK10	turquoise	-0.363726505	4.46E-09	-0.663482767	1.86E-32
MAPK6	turquoise	-0.30054735	1.66E-06	-0.64371064	4.62E-30
MAPK9	turquoise	-0.329604144	1.28E-07	-0.720348135	1.74E-40
MAPKAPK2	turquoise	0.323176768	2.31E-07	0.495717217	1.34E-16
MAT2B	turquoise	-0.375853967	1.22E-09	-0.563689523	6.00E-22
MATR3	turquoise	-0.381313818	6.71E-10	-0.53521223	1.47E-19
MCF2	turquoise	-0.235202006	0.000203271	-0.308188048	8.68E-07
MCL1	turquoise	0.396595355	1.18E-10	0.634561538	5.17E-29
MCM7	turquoise	0.471822701	5.51E-15	0.648965707	1.11E-30
MDH2	turquoise	-0.395302539	1.37E-10	-0.650893829	6.53E-31

MDM2	turquoise	0.284866091	5.89E-06	0.480329673	1.52E-15
ME1	turquoise	-0.237684148	0.00017315	-0.499608485	7.12E-17
ME3	turquoise	-0.178789148	0.00500313	-0.457512126	4.46E-14
MED10	turquoise	-0.28571898	5.51E-06	-0.38282931	5.67E-10
MED16	turquoise	0.309910818	7.48E-07	0.480848322	1.40E-15
MED31	turquoise	-0.182751329	0.004103526	-0.322474605	2.47E-07
MET	turquoise	-0.290063641	3.90E-06	-0.332694118	9.63E-08
METAP1	turquoise	-0.303016079	1.35E-06	-0.653477693	3.19E-31
METTL7B	turquoise	0.263513765	2.95E-05	0.284814852	5.91E-06
MFNG	turquoise	0.356966751	8.96E-09	0.466563863	1.20E-14
MFSD6	turquoise	-0.265018326	2.64E-05	-0.504566202	3.14E-17
MGAT1	turquoise	0.287152716	4.92E-06	0.511487718	9.80E-18
MGST3	turquoise	-0.298036703	2.04E-06	-0.549328451	1.03E-20
MICALL2	turquoise	0.225091397	0.000383922	0.293348168	2.99E-06
MICB	turquoise	0.202265427	0.001458963	0.424569572	3.84E-12
MID1IP1	turquoise	0.457885903	4.23E-14	0.615444042	6.26E-27
MKKS	turquoise	-0.273273159	1.44E-05	-0.215600688	0.000680119
MKNK2	turquoise	0.262626892	3.14E-05	0.699395366	2.63E-37
MLEC	turquoise	0.304141931	1.22E-06	0.151866265	0.01737192
MLKL	turquoise	0.388036593	3.16E-10	0.532473643	2.43E-19
MLLT6	turquoise	0.43326823	1.24E-12	0.740928361	6.65E-44
MMADHC	turquoise	-0.248380317	8.51E-05	-0.501770368	4.99E-17
MMD	turquoise	-0.279154108	9.18E-06	-0.578235801	2.93E-23
MNAT1	turquoise	-0.111299959	0.08210226	-0.178618159	0.005045666
MOAP1	turquoise	-0.356682553	9.22E-09	-0.749138627	2.33E-45
MORF4L2	turquoise	-0.293080041	3.06E-06	-0.799369117	1.11E-55
MOV10	turquoise	0.32635587	1.73E-07	0.345863346	2.72E-08
MPP1	turquoise	-0.264610505	2.72E-05	-0.512525974	8.21E-18
MPST	turquoise	0.237914323	0.000170579	0.47269346	4.84E-15
MPZL2	turquoise	0.210997859	0.000889778	0.22611736	0.000360386
MRAP2	turquoise	-0.183058763	0.004040234	-0.368132288	2.80E-09
MRPL1	turquoise	-0.355573738	1.03E-08	-0.430420723	1.80E-12
MRPL15	turquoise	-0.355303278	1.06E-08	-0.292665547	3.16E-06
MRPL21	turquoise	-0.320600655	2.92E-07	-0.476081778	2.90E-15
MRPL3	turquoise	-0.256203611	4.96E-05	-0.711782111	3.76E-39
MRPL32	turquoise	-0.251491145	6.88E-05	-0.357445285	8.53E-09
MRPL40	turquoise	-0.209643917	0.000961938	-0.344809704	3.02E-08
MRPL47	turquoise	-0.202506032	0.001439602	-0.225786803	0.000367819
MRPS21	turquoise	-0.251405908	6.92E-05	-0.401292505	6.78E-11
MRPS30	turquoise	-0.450618055	1.18E-13	-0.721614699	1.10E-40

MRVI1	turquoise	0.281225414	7.83E-06	0.288509171	4.42E-06
MS4A14	turquoise	0.371479282	1.96E-09	0.268173739	2.10E-05
MS4A6A	turquoise	0.287215731	4.89E-06	0.170231383	0.007575299
MSH2	turquoise	-0.213620234	0.000764	-0.409139397	2.64E-11
MSI2	turquoise	0.143852333	0.024331442	0.358170862	7.92E-09
MSN	turquoise	0.22695428	0.000342188	0.256785461	4.76E-05
MSRB2	turquoise	0.174005934	0.006322748	0.566954274	3.09E-22
MT1E	turquoise	0.347547673	2.31E-08	0.484338345	8.16E-16
MT1F	turquoise	0.372828974	1.70E-09	0.408239742	2.95E-11
MT1G	turquoise	0.29707535	2.21E-06	0.371216347	2.02E-09
MT1H	turquoise	0.300485857	1.66E-06	0.367968116	2.85E-09
MT1M	turquoise	0.322841173	2.39E-07	0.417576661	9.32E-12
MT1X	turquoise	0.207798317	0.001068991	0.353090354	1.33E-08
MT2A	turquoise	0.347629296	2.29E-08	0.403327712	5.32E-11
MTCH1	turquoise	-0.316579237	4.18E-07	-0.580049552	1.99E-23
MUC1	turquoise	0.372235212	1.81E-09	0.661364938	3.43E-32
MYH10	turquoise	-0.276749351	1.10E-05	-0.355038093	1.09E-08
MYH11	turquoise	0.296952062	2.23E-06	0.255296055	5.28E-05
MYOF	turquoise	0.247749526	8.88E-05	0.207916541	0.001061818
MYOM1	turquoise	0.295224931	2.57E-06	0.526592766	7.02E-19
MYOT	turquoise	0.400354868	7.58E-11	0.486280688	6.02E-16
MYT1L	turquoise	-0.377724071	9.97E-10	-0.665571776	1.01E-32
NACC2	turquoise	0.435663485	9.04E-13	0.677264348	3.09E-34
NAP1L2	turquoise	-0.349173502	1.96E-08	-0.614332354	8.19E-27
NAP1L3	turquoise	-0.354412361	1.16E-08	-0.711677689	3.90E-39
NAP1L5	turquoise	-0.462804564	2.08E-14	-0.717740351	4.49E-40
NAPB	turquoise	-0.37802219	9.65E-10	-0.573403846	8.13E-23
NAPEPLD	turquoise	-0.253637704	5.93E-05	-0.370622599	2.15E-09
NAV2	turquoise	0.370673867	2.14E-09	0.48970275	3.51E-16
NAV3	turquoise	-0.318865888	3.41E-07	-0.580324198	1.88E-23
NBEA	turquoise	-0.316584967	4.18E-07	-0.663015254	2.13E-32
NBEAL2	turquoise	0.30754698	9.17E-07	0.497786473	9.58E-17
NCAM2	turquoise	-0.191817016	0.00256891	-0.472044376	5.33E-15
NCAPD2	turquoise	0.318017014	3.68E-07	0.411594587	1.96E-11
NCKAP1	turquoise	-0.208473562	0.001028614	-0.774612058	2.92E-50
NCOA7	turquoise	-0.331494494	1.08E-07	-0.696524005	6.82E-37
NDE1	turquoise	0.255819405	5.09E-05	0.656641659	1.31E-31
NDEL1	turquoise	-0.375138532	1.32E-09	-0.424099189	4.08E-12
NDFIP1	turquoise	-0.368967969	2.57E-09	-0.665346714	1.08E-32
NDFIP2	turquoise	-0.350410933	1.74E-08	-0.636922093	2.80E-29

NDN	turquoise	-0.187192704	0.003270627	-0.411863746	1.89E-11
NDRG3	turquoise	-0.283265309	6.68E-06	-0.560350675	1.18E-21
NDRG4	turquoise	-0.417820454	9.04E-12	-0.70819413	1.32E-38
NDUFA1	turquoise	-0.223858477	0.000414092	-0.519167279	2.61E-18
NDUFA12	turquoise	-0.207404058	0.001093235	-0.537813411	9.08E-20
NDUFA4	turquoise	-0.245413713	0.000103939	-0.629411875	1.95E-28
NDUFA7	turquoise	-0.112753541	0.07815926	-0.079692619	0.213873195
NDUFA9	turquoise	-0.254536441	5.57E-05	-0.357023537	8.91E-09
NDUFAB1	turquoise	-0.409375785	2.57E-11	-0.621505768	1.42E-27
NDUFB5	turquoise	-0.37387278	1.52E-09	-0.518778178	2.79E-18
NDUFB6	turquoise	-0.275083515	1.25E-05	-0.619331707	2.42E-27
NDUFS5	turquoise	-0.152966938	0.016566709	-0.409047999	2.67E-11
NECAB1	turquoise	-0.207336357	0.001097448	-0.500092793	6.57E-17
NECAP1	turquoise	-0.337666534	6.02E-08	-0.67764334	2.75E-34
NECAP2	turquoise	0.337959447	5.85E-08	0.50204774	4.77E-17
NEDD4L	turquoise	-0.279669471	8.82E-06	-0.224118059	0.000407563
NEDD8	turquoise	-0.370893539	2.09E-09	-0.381998451	6.22E-10
NEFH	turquoise	-0.109722952	0.08655976	-0.301714048	1.50E-06
NEFM	turquoise	-0.358267926	7.84E-09	-0.653355891	3.30E-31
NEK6	turquoise	0.332374898	9.92E-08	0.58740429	4.04E-24
NELL1	turquoise	-0.282101491	7.31E-06	-0.623175578	9.37E-28
NELL2	turquoise	-0.308741879	8.27E-07	-0.682867929	5.47E-35
NES	turquoise	0.256612036	4.82E-05	0.360949459	5.95E-09
NET1	turquoise	0.18136401	0.004400404	0.329731259	1.27E-07
NETO2	turquoise	-0.271729499	1.61E-05	-0.431005986	1.67E-12
NEU4	turquoise	0.100951671	0.115004695	0.430973091	1.68E-12
NEUROD6	turquoise	-0.322756612	2.40E-07	-0.494555933	1.62E-16
NFATC2IP	turquoise	0.223226559	0.000430394	0.715314334	1.07E-39
NFIA	turquoise	0.364778485	3.99E-09	0.398769929	9.13E-11
NFIB	turquoise	0.381767441	6.38E-10	0.421867672	5.42E-12
NFKB1	turquoise	0.477794705	2.24E-15	0.582113097	1.28E-23
NFKBIA	turquoise	0.479714683	1.67E-15	0.604886612	7.71E-26
NFKBIZ	turquoise	0.350188317	1.77E-08	0.485020348	7.33E-16
NGRN	turquoise	-0.307847414	8.93E-07	-0.519387988	2.51E-18
NIF3L1	turquoise	-0.290622374	3.73E-06	-0.557675945	2.00E-21
NINJ1	turquoise	0.494712217	1.58E-16	0.50372225	3.61E-17
NIPBL	turquoise	0.487112266	5.28E-16	0.734950845	7.05E-43
NKIRAS1	turquoise	-0.285214236	5.73E-06	-0.516532834	4.12E-18
NKRF	turquoise	-0.18330312	0.003990557	-0.277340153	1.05E-05
NLK	turquoise	-0.324555512	2.04E-07	-0.323854536	2.18E-07

NME1	turquoise	-0.367217013	3.09E-09	-0.657527306	1.02E-31
NME3	turquoise	0.280279899	8.42E-06	0.588184634	3.40E-24
NME4	turquoise	0.119601053	0.061595879	0.278522627	9.64E-06
NME7	turquoise	0.009382903	0.883828401	-0.319350616	3.27E-07
NMNAT2	turquoise	-0.423208139	4.57E-12	-0.596079872	5.84E-25
NNAT	turquoise	-0.292786862	3.13E-06	-0.386083366	3.94E-10
NNT	turquoise	-0.259326782	3.98E-05	-0.635782828	3.76E-29
NOSTRIN	turquoise	0.237524492	0.000174955	0.327446838	1.57E-07
NOTCH2	turquoise	0.292212538	3.28E-06	0.06386778	0.319447813
NOTCH4	turquoise	0.369444295	2.44E-09	0.479672912	1.68E-15
NOVA1	turquoise	-0.380478829	7.36E-10	-0.494669415	1.59E-16
NPAS3	turquoise	0.206668337	0.001139832	0.366588252	3.30E-09
NPTN	turquoise	-0.387242695	3.45E-10	-0.527511328	5.95E-19
NPTX1	turquoise	-0.30712428	9.50E-07	-0.258939267	4.09E-05
NPTX2	turquoise	-0.440954457	4.45E-13	-0.478763198	1.93E-15
NPY	turquoise	-0.17357992	0.006454184	-0.493915398	1.79E-16
NR2F1	turquoise	0.26203409	3.28E-05	0.615756592	5.80E-27
NRCAM	turquoise	-0.219930059	0.000525528	-0.211433023	0.000867666
NRN1	turquoise	-0.479492217	1.72E-15	-0.428780126	2.23E-12
NRXN1	turquoise	-0.359652211	6.80E-09	-0.606971211	4.73E-26
NRXN3	turquoise	-0.383808933	5.08E-10	-0.295040959	2.61E-06
NSF	turquoise	-0.394811206	1.45E-10	-0.612306248	1.33E-26
NSL1	turquoise	0.25104941	7.09E-05	0.503046843	4.04E-17
NT5DC3	turquoise	-0.386132009	3.92E-10	-0.510652732	1.13E-17
NTNG1	turquoise	-0.205845794	0.001194083	-0.431906778	1.48E-12
NUAK1	turquoise	-0.359259323	7.08E-09	-0.354326642	1.17E-08
NUDT10	turquoise	-0.235269483	0.000202391	-0.216071245	0.000661481
NUDT11	turquoise	-0.32566106	1.85E-07	-0.625265879	5.55E-28
NUDT21	turquoise	-0.330967167	1.13E-07	-0.66002854	5.03E-32
NUDT9	turquoise	-0.085587238	0.181793743	-0.393105754	1.77E-10
NUPR1	turquoise	0.358310319	7.81E-09	0.461025457	2.69E-14
NXN	turquoise	0.277156916	1.07E-05	0.485330534	6.99E-16
OAT	turquoise	-0.160333611	0.011968805	-0.668212722	4.67E-33
OCA2	turquoise	-0.109448171	0.087355956	-0.358611883	7.57E-09
OCIAD2	turquoise	-0.235855709	0.000194896	-0.402954138	5.56E-11
OLA1	turquoise	-0.320564878	2.93E-07	-0.466320754	1.25E-14
OLFM1	turquoise	-0.417377284	9.55E-12	-0.483492461	9.30E-16
OLFM3	turquoise	-0.249180381	8.06E-05	-0.613879898	9.14E-27
OLFM4	turquoise	-0.222579075	0.000447715	-0.13586212	0.033539202
OLIG1	turquoise	0.27420138	1.34E-05	0.455456359	5.97E-14

OPA1	turquoise	-0.314047249	5.22E-07	-0.688028095	1.07E-35
OPCML	turquoise	-0.352240254	1.45E-08	-0.466266054	1.26E-14
OPN3	turquoise	-0.229981299	0.00028326	-0.630285244	1.56E-28
OPTN	turquoise	-0.207457558	0.001089915	-0.554697014	3.61E-21
OR2L13	turquoise	-0.243865527	0.000115279	-0.339920754	4.85E-08
OSBPL10	turquoise	-0.257942939	4.39E-05	-0.563868463	5.79E-22
OSTF1	turquoise	-0.161602349	0.011302213	-0.530462428	3.50E-19
OTOS	turquoise	0.284458293	6.08E-06	0.230845284	0.000268262
OXCT1	turquoise	-0.169258793	0.007931935	-0.57189375	1.11E-22
OXR1	turquoise	-0.282966968	6.83E-06	-0.637492239	2.41E-29
PACSIN1	turquoise	-0.357412648	8.56E-09	-0.270015706	1.83E-05
PACSIN3	turquoise	0.295948342	2.42E-06	0.343424824	3.46E-08
PAIP2	turquoise	-0.234784765	0.000208791	-0.77805769	5.66E-51
PAK1	turquoise	-0.425181094	3.55E-12	-0.630813811	1.36E-28
PAM	turquoise	-0.22918496	0.000297771	-0.525249435	8.92E-19
PAPSS1	turquoise	-0.213855028	0.000753579	-0.147690891	0.020744516
PARP10	turquoise	0.332386899	9.91E-08	0.75159249	8.34E-46
PARP14	turquoise	0.335881618	7.13E-08	0.42338296	4.47E-12
PARP4	turquoise	0.364454383	4.13E-09	0.35133223	1.58E-08
PARP9	turquoise	0.324252293	2.10E-07	0.288553661	4.40E-06
PARVG	turquoise	0.28760128	4.75E-06	0.267731056	2.17E-05
PAX6	turquoise	0.251645896	6.80E-05	0.341506726	4.16E-08
PBXIP1	turquoise	0.196509186	0.00199946	0.29646867	2.32E-06
PCDH17	turquoise	0.210888435	0.000895419	0.577811519	3.21E-23
PCDH8	turquoise	-0.237653936	0.00017349	-0.52403392	1.11E-18
PCLO	turquoise	-0.33599712	7.05E-08	-0.580373651	1.86E-23
PCMT1	turquoise	-0.364694565	4.03E-09	-0.582036365	1.30E-23
PCP4	turquoise	-0.359346	7.02E-09	-0.174425868	0.006195536
PCSK1	turquoise	-0.465003042	1.51E-14	-0.480483354	1.48E-15
PCSK2	turquoise	-0.24017681	0.000147141	-0.403634288	5.13E-11
PCSK5	turquoise	0.250694117	7.26E-05	0.423012536	4.69E-12
PCYOX1L	turquoise	-0.419597346	7.23E-12	-0.484622428	7.80E-16
PDCD6IP	turquoise	-0.125837096	0.049135204	-0.440964053	4.44E-13
PDE5A	turquoise	0.366116192	3.47E-09	0.499615776	7.11E-17
PDE9A	turquoise	0.329587962	1.29E-07	0.68675427	1.61E-35
PDGFRB	turquoise	0.484899438	7.47E-16	0.617125049	4.16E-27
PDHB	turquoise	-0.349816591	1.84E-08	-0.603439174	1.08E-25
PDHX	turquoise	-0.285598861	5.56E-06	-0.676893441	3.46E-34
PDPK1	turquoise	-0.231904051	0.000250892	-0.448653472	1.55E-13
PDPN	turquoise	0.277443055	1.05E-05	0.38011673	7.66E-10

PECAM1	turquoise	0.232140928	0.000247152	0.137737945	0.031146431
PFKFB4	turquoise	0.344433802	3.13E-08	0.554702804	3.60E-21
PFKM	turquoise	-0.241323015	0.000136451	-0.430022146	1.90E-12
PFN2	turquoise	-0.458569321	3.83E-14	-0.777967475	5.91E-51
PGBD5	turquoise	-0.214362348	0.000731509	-0.491127131	2.80E-16
PGRMC1	turquoise	-0.356259313	9.63E-09	-0.69177251	3.22E-36
PHF14	turquoise	-0.321900868	2.60E-07	-0.763742151	4.29E-48
PHF19	turquoise	0.367603728	2.96E-09	0.577489466	3.43E-23
PHF2	turquoise	0.263815828	2.88E-05	0.497556746	9.95E-17
PHKA2	turquoise	0.241476421	0.000135076	0.72481414	3.35E-41
PHYHD1	turquoise	0.323728989	2.20E-07	0.239557377	0.000153239
PI16	turquoise	0.242255976	0.000128291	0.423257261	4.55E-12
PID1	turquoise	-0.175784924	0.005799386	-0.618699833	2.83E-27
PIGO	turquoise	0.299186006	1.85E-06	0.314190203	5.16E-07
PIGQ	turquoise	0.157410469	0.013637972	0.590176063	2.19E-24
PIK3CB	turquoise	-0.195368681	0.00212614	-0.62588806	4.75E-28
PIM1	turquoise	0.200901756	0.001573274	0.263813536	2.88E-05
PITPNB	turquoise	-0.337565488	6.08E-08	-0.509043605	1.48E-17
PJA1	turquoise	-0.347062876	2.42E-08	-0.640578156	1.07E-29
PJA2	turquoise	-0.350581943	1.71E-08	-0.536269212	1.21E-19
PKIA	turquoise	-0.414413579	1.38E-11	-0.440862767	4.50E-13
PKN2	turquoise	0.388003047	3.17E-10	0.617579892	3.72E-27
PLA1A	turquoise	0.243619738	0.000117183	0.304490258	1.19E-06
PLCD1	turquoise	0.264372775	2.77E-05	0.267734734	2.17E-05
PLCD3	turquoise	0.247782433	8.86E-05	0.345800534	2.74E-08
PLCE1	turquoise	0.500603829	6.05E-17	0.404634099	4.55E-11
PLCG1	turquoise	0.371743929	1.91E-09	0.69867826	3.34E-37
PLCG2	turquoise	0.269776017	1.86E-05	0.241605359	0.000133931
PLCL2	turquoise	-0.334530096	8.10E-08	-0.475803338	3.03E-15
PLEKHA7	turquoise	0.355720571	1.02E-08	0.296205771	2.37E-06
PLEKHB1	turquoise	0.14276229	0.025442714	0.190456638	0.002759609
PLEKHH3	turquoise	0.241228404	0.000137305	0.609211659	2.79E-26
PLEKHO2	turquoise	0.359746007	6.74E-09	0.500748654	5.90E-17
PLK2	turquoise	-0.386787075	3.64E-10	-0.356215874	9.67E-09
PLOD1	turquoise	0.334340253	8.25E-08	0.819714462	9.63E-61
PLSCR3	turquoise	0.160537065	0.011859632	0.465327864	1.44E-14
PML	turquoise	0.289230157	4.17E-06	0.382773179	5.70E-10
PMPCB	turquoise	-0.246328747	9.77E-05	-0.462778037	2.09E-14
PNMA1	turquoise	-0.312664949	5.89E-07	-0.739946646	9.85E-44
PNMA2	turquoise	-0.283266621	6.68E-06	-0.626527885	4.04E-28

POGK	turquoise	0.440342587	4.83E-13	0.557292179	2.16E-21
POLA1	turquoise	0.45918824	3.51E-14	0.462133142	2.29E-14
POLR3C	turquoise	-0.207599657	0.001081144	-0.385647764	4.13E-10
POLR3H	turquoise	0.438989874	5.80E-13	0.576324256	4.40E-23
POP7	turquoise	-0.209975182	0.000943802	-0.478213779	2.10E-15
POU2F1	turquoise	0.29976913	1.77E-06	0.632374338	9.11E-29
PPA1	turquoise	-0.272267049	1.55E-05	-0.699673661	2.39E-37
PPEF1	turquoise	-0.337135964	6.33E-08	-0.342625715	3.73E-08
PPFIA1	turquoise	0.456417517	5.21E-14	0.638047345	2.08E-29
PPIA	turquoise	-0.125374297	0.049981398	-0.426080431	3.17E-12
PPM1H	turquoise	-0.264323525	2.78E-05	-0.38551499	4.20E-10
PPP1R14C	turquoise	-0.195421836	0.002120077	-0.305352669	1.11E-06
PPP1R1A	turquoise	0.328376369	1.44E-07	0.363442568	4.59E-09
PPP1R2	turquoise	-0.295575168	2.50E-06	-0.662108845	2.77E-32
PPP2CA	turquoise	-0.271244479	1.67E-05	-0.386276079	3.85E-10
PPP2R1B	turquoise	0.257124292	4.65E-05	0.308367152	8.54E-07
PPP2R2B	turquoise	-0.35211278	1.46E-08	-0.60326318	1.13E-25
PPP3CA	turquoise	-0.36474264	4.01E-09	-0.504130364	3.38E-17
PPP3CB	turquoise	-0.353887806	1.22E-08	-0.611494339	1.62E-26
PPP3R1	turquoise	-0.323997653	2.15E-07	-0.3921006	1.98E-10
PPP4R4	turquoise	-0.193633851	0.002332902	-0.525281243	8.87E-19
PPT1	turquoise	-0.316057413	4.37E-07	-0.315346043	4.66E-07
PRDM2	turquoise	-0.21680725	0.000633274	-0.159330759	0.012519969
PRDX3	turquoise	-0.094492517	0.140271786	-0.618324506	3.10E-27
PREP	turquoise	-0.206018675	0.001182488	-0.475629472	3.11E-15
PREPL	turquoise	-0.359391025	6.99E-09	-0.533374858	2.06E-19
PREX1	turquoise	0.212600204	0.000810835	0.359011216	7.27E-09
PRKACB	turquoise	-0.320093005	3.06E-07	-0.662340902	2.59E-32
PRKCB	turquoise	-0.352311984	1.43E-08	-0.705155639	3.75E-38
PRKCE	turquoise	-0.265654106	2.52E-05	-0.4594116	3.40E-14
PRKX	turquoise	0.433422061	1.22E-12	0.469370114	7.94E-15
PRMT6	turquoise	-0.223766787	0.000416421	-0.497123373	1.07E-16
PRMT8	turquoise	-0.328194964	1.46E-07	-0.139773523	0.028716125
PRNP	turquoise	-0.350919748	1.65E-08	-0.558472086	1.71E-21
PRPS1	turquoise	-0.341594663	4.13E-08	-0.714267338	1.56E-39
PRPS2	turquoise	-0.192855417	0.002431494	-0.319949789	3.10E-07
PSMA1	turquoise	-0.22894975	0.000302188	-0.646043149	2.46E-30
PSMA3	turquoise	-0.283258226	6.68E-06	-0.69421906	1.45E-36
PSMA5	turquoise	-0.279203047	9.15E-06	-0.524730812	9.78E-19
PSMB2	turquoise	-0.362787415	4.92E-09	-0.491980203	2.44E-16

PSMC2	turquoise	-0.197130511	0.001933382	-0.595731591	6.32E-25
PSMD1	turquoise	-0.328845783	1.38E-07	-0.633206963	7.35E-29
PSMD10	turquoise	-0.316538161	4.19E-07	-0.638391225	1.90E-29
PSMD14	turquoise	-0.362374141	5.13E-09	-0.737643125	2.45E-43
PSMD6	turquoise	-0.198081922	0.001836053	-0.534815514	1.58E-19
PSMG1	turquoise	-0.30686705	9.71E-07	-0.721273077	1.24E-40
PTBP1	turquoise	0.426855855	2.86E-12	0.767430608	8.14E-49
PTDSS1	turquoise	-0.25886806	4.11E-05	-0.570256942	1.56E-22
PTDSS2	turquoise	0.2041505	0.001313451	0.507838656	1.82E-17
PTH1R	turquoise	0.311897019	6.30E-07	0.78277095	5.72E-52
PTH2R	turquoise	-0.300284719	1.69E-06	-0.421504168	5.68E-12
PTPN3	turquoise	-0.302124425	1.45E-06	-0.32226373	2.51E-07
PTPRN2	turquoise	-0.310203274	7.29E-07	-0.697116139	5.60E-37
PTPRT	turquoise	-0.12309303	0.054331888	-0.426299666	3.08E-12
PTRH2	turquoise	-0.27781119	1.02E-05	-0.358024583	8.04E-09
PTTG1	turquoise	-0.155544107	0.014807616	-0.129465568	0.042906207
PUM2	turquoise	-0.153307608	0.01632418	-0.501078012	5.59E-17
PXN	turquoise	0.226696277	0.000347705	0.390724125	2.32E-10
PYGM	turquoise	0.313123708	5.66E-07	0.230280559	0.000277979
QKI	turquoise	0.226845178	0.000344511	0.223999752	0.000410526
QPCT	turquoise	-0.288301823	4.49E-06	-0.695694967	8.96E-37
R3HDM1	turquoise	-0.347303927	2.36E-08	-0.444330957	2.81E-13
RAB11A	turquoise	-0.320625946	2.91E-07	-0.421935754	5.38E-12
RAB15	turquoise	-0.255878524	5.07E-05	-0.340931473	4.40E-08
RAB22A	turquoise	-0.25141357	6.91E-05	-0.44388473	2.99E-13
RAB2A	turquoise	-0.270345902	1.79E-05	-0.563243751	6.57E-22
RAB6A	turquoise	-0.400634115	7.33E-11	-0.718973293	2.87E-40
RAB6B	turquoise	-0.233632672	0.000224768	-0.28711059	4.93E-06
RAD23B	turquoise	-0.178896284	0.004976642	-0.610056843	2.28E-26
RAD51C	turquoise	-0.303357159	1.31E-06	-0.61795555	3.40E-27
RAI14	turquoise	0.295976048	2.42E-06	0.45395389	7.39E-14
RALB	turquoise	-0.187988007	0.003138777	-0.500042867	6.63E-17
RALGDS	turquoise	0.30826579	8.62E-07	0.494789925	1.56E-16
RALYL	turquoise	-0.315239989	4.70E-07	-0.476022493	2.93E-15
RAP1GDS1	turquoise	-0.31127698	6.65E-07	-0.729603968	5.53E-42
RAP2A	turquoise	-0.302658933	1.39E-06	-0.517845175	3.28E-18
RAPGEF1	turquoise	0.244366407	0.000111489	0.569628917	1.78E-22
RAPGEF2	turquoise	-0.271150396	1.68E-05	-0.500570475	6.08E-17
RARRES2	turquoise	0.286264258	5.28E-06	0.439153039	5.67E-13
RASA1	turquoise	-0.179218025	0.004897854	-0.581469736	1.47E-23

RASAL3	turquoise	0.302359572	1.42E-06	0.595376041	6.84E-25
RASD1	turquoise	-0.022710889	0.723556743	-0.210200979	0.00093162
RASGRF1	turquoise	-0.374742067	1.38E-09	-0.239216826	0.000156691
RASGRP1	turquoise	-0.36022999	6.41E-09	-0.308130957	8.72E-07
RASL11B	turquoise	-0.286184352	5.31E-06	-0.561648189	9.06E-22
RASL12	turquoise	0.405230285	4.24E-11	0.508540053	1.61E-17
RASSF1	turquoise	0.353458357	1.28E-08	0.385568461	4.17E-10
RASSF4	turquoise	0.327763447	1.52E-07	0.522905719	1.35E-18
RBM11	turquoise	-0.232467923	0.000242074	-0.523659368	1.18E-18
RBM17	turquoise	0.399051138	8.84E-11	0.592483019	1.31E-24
RBMS2	turquoise	0.180585831	0.004575272	0.344103146	3.23E-08
RBP4	turquoise	-0.189432276	0.002911589	-0.424929583	3.67E-12
RBPJ	turquoise	0.465560072	1.39E-14	0.575441224	5.30E-23
RCAN2	turquoise	-0.408734162	2.77E-11	-0.621069211	1.58E-27
RDH5	turquoise	0.230417538	0.000275592	0.442293506	3.71E-13
REEP1	turquoise	-0.346973914	2.44E-08	-0.505998408	2.47E-17
REEP5	turquoise	-0.300707767	1.63E-06	-0.494057467	1.75E-16
RELL1	turquoise	0.163241241	0.010489756	0.337508817	6.11E-08
REPS2	turquoise	-0.261150915	3.49E-05	-0.672563923	1.28E-33
REST	turquoise	0.335863285	7.14E-08	0.283061848	6.78E-06
RFTN1	turquoise	-0.31404872	5.22E-07	-0.27710545	1.07E-05
RFX2	turquoise	0.384616755	4.64E-10	0.342100514	3.93E-08
RGL2	turquoise	0.373982351	1.50E-09	0.459295725	3.45E-14
RGMA	turquoise	0.274904939	1.27E-05	0.322962147	2.36E-07
RGR	turquoise	0.336402379	6.79E-08	0.184112997	0.003829827
RGS2	turquoise	-0.194637366	0.002211174	-0.102532483	0.109398701
RGS4	turquoise	-0.378809496	8.85E-10	-0.664242016	1.49E-32
RGS7	turquoise	-0.443948158	2.96E-13	-0.697200582	5.45E-37
RHBDF1	turquoise	0.37479995	1.37E-09	0.689185308	7.42E-36
RHBDF2	turquoise	0.379081814	8.59E-10	0.530406208	3.53E-19
RHOC	turquoise	0.29623298	2.37E-06	0.451604014	1.03E-13
RIMBP2	turquoise	-0.271455871	1.64E-05	-0.505040052	2.90E-17
RIMS1	turquoise	-0.326382992	1.73E-07	-0.345256823	2.89E-08
RIN3	turquoise	0.213109162	0.000787145	0.117878854	0.065462359
RIPPLY2	turquoise	-0.23451726	0.000212403	-0.347362666	2.35E-08
RIT2	turquoise	-0.223720281	0.000417607	-0.359532561	6.89E-09
RND2	turquoise	0.301837565	1.49E-06	0.390762968	2.31E-10
RNF135	turquoise	0.231562238	0.000256382	0.425076389	3.60E-12
RNF144B	turquoise	0.370520367	2.17E-09	0.277941347	1.01E-05
RNF175	turquoise	-0.346937661	2.45E-08	-0.485911273	6.38E-16

RNF41	turquoise	-0.283939395	6.33E-06	-0.657906946	9.19E-32
RNFT2	turquoise	-0.399027287	8.86E-11	-0.409231782	2.61E-11
ROBO3	turquoise	0.153162534	0.01642708	0.452248384	9.39E-14
RPA3	turquoise	-0.257358608	4.57E-05	-0.559205463	1.48E-21
RPAIN	turquoise	-0.273415006	1.42E-05	-0.406555457	3.61E-11
RPL15	turquoise	-0.413562919	1.54E-11	-0.795209809	1.01E-54
RPN2	turquoise	0.256793258	4.76E-05	0.716698768	6.53E-40
RPP40	turquoise	-0.308387045	8.53E-07	-0.250329319	7.45E-05
RPS6KA1	turquoise	0.244910195	0.000107507	0.441405711	4.18E-13
RRAGA	turquoise	-0.394731858	1.46E-10	-0.76812055	5.94E-49
RRAGB	turquoise	-0.284894615	5.88E-06	-0.474994443	3.42E-15
RSPO2	turquoise	-0.26801848	2.12E-05	-0.541774739	4.33E-20
RTN1	turquoise	-0.445094685	2.53E-13	-0.750065174	1.58E-45
RTN3	turquoise	-0.364607048	4.06E-09	-0.424471546	3.89E-12
RTN4	turquoise	-0.464511969	1.62E-14	-0.765889026	1.64E-48
RTN4IP1	turquoise	-0.277503701	1.04E-05	-0.451075748	1.11E-13
RUFY3	turquoise	-0.321417678	2.71E-07	-0.727886211	1.06E-41
RUNDC1	turquoise	-0.343391309	3.47E-08	-0.566942644	3.10E-22
RWDD2A	turquoise	-0.221900272	0.000466565	-0.438910869	5.86E-13
S100A10	turquoise	0.279566513	8.89E-06	0.215206028	0.000696123
S100A6	turquoise	0.343525907	3.42E-08	0.423195982	4.58E-12
S100PBP	turquoise	0.30626743	1.02E-06	0.531165292	3.08E-19
SAP30L	turquoise	0.409981435	2.38E-11	0.592671543	1.26E-24
SAT1	turquoise	0.173328993	0.006532739	0.24448299	0.000110624
SATB2	turquoise	-0.164638891	0.009838141	-0.380820809	7.09E-10
SCAMP1	turquoise	-0.333264288	9.13E-08	-0.287019413	4.97E-06
SCG2	turquoise	-0.387115822	3.50E-10	-0.593467201	1.05E-24
SCG3	turquoise	-0.426322056	3.07E-12	-0.452129764	9.55E-14
SCG5	turquoise	-0.445007521	2.56E-13	-0.618417674	3.03E-27
SCN2B	turquoise	-0.414993385	1.29E-11	-0.681153508	9.33E-35
SCN3B	turquoise	-0.310015488	7.41E-07	-0.590374687	2.10E-24
SCO1	turquoise	-0.219905563	0.000526302	-0.589779067	2.39E-24
SCOC	turquoise	-0.373466086	1.58E-09	-0.732665957	1.71E-42
SDAD1	turquoise	-0.338985831	5.30E-08	-0.30534061	1.11E-06
SDC2	turquoise	0.214709797	0.00071674	0.485665711	6.63E-16
SDHA	turquoise	-0.304870838	1.15E-06	-0.489821888	3.45E-16
SDHB	turquoise	-0.327489775	1.56E-07	-0.641461115	8.43E-30
SEC61A2	turquoise	-0.319787067	3.14E-07	-0.455540076	5.90E-14
SEC61G	turquoise	-0.372196677	1.82E-09	-0.52933357	4.29E-19
SEH1L	turquoise	-0.421190023	5.91E-12	-0.663717367	1.74E-32

SELENBP1	turquoise	0.354805734	1.12E-08	0.291992103	3.34E-06
SEMA4F	turquoise	-0.2571756	4.63E-05	-0.526520487	7.11E-19
SERINC1	turquoise	-0.325653392	1.85E-07	-0.744642294	1.48E-44
SERPINA3	turquoise	0.225683883	0.000370162	0.134615761	0.035214006
SERPINB6	turquoise	0.369881039	2.33E-09	0.660735283	4.11E-32
SERPINI1	turquoise	-0.304243394	1.21E-06	-0.722573002	7.70E-41
SERTAD1	turquoise	0.241122572	0.000138266	0.337404612	6.17E-08
SEZ6L2	turquoise	-0.427150413	2.76E-12	-0.271324201	1.66E-05
SGIP1	turquoise	-0.303583873	1.28E-06	-0.604836921	7.80E-26
SH3GL2	turquoise	-0.345118085	2.93E-08	-0.50562045	2.63E-17
SH3PXD2A	turquoise	0.214560642	0.000723046	0.596924663	4.82E-25
SHD	turquoise	-0.186700642	0.003354694	0.132664765	0.037977883
SIK1	turquoise	0.265469376	2.56E-05	0.200561669	0.001603031
SIX5	turquoise	0.277840598	1.02E-05	0.609991604	2.32E-26
SKAP2	turquoise	-0.140055844	0.028392224	-0.5367471	1.11E-19
SLBP	turquoise	-0.297064464	2.21E-06	-0.289103237	4.21E-06
SLC11A1	turquoise	0.217094992	0.000622552	0.249324993	7.98E-05
SLC12A5	turquoise	-0.341258332	4.26E-08	-0.629250738	2.03E-28
SLC15A3	turquoise	0.458975761	3.62E-14	0.506030122	2.46E-17
SLC16A14	turquoise	-0.417626793	9.26E-12	-0.719399624	2.46E-40
SLC16A9	turquoise	0.348413119	2.12E-08	0.518091893	3.14E-18
SLC1A1	turquoise	-0.208735987	0.001013304	-0.435021395	9.84E-13
SLC20A1	turquoise	-0.246101969	9.92E-05	-0.532338283	2.49E-19
SLC25A12	turquoise	-0.265259195	2.60E-05	-0.480373777	1.51E-15
SLC25A14	turquoise	-0.302960465	1.35E-06	-0.20096505	0.001567792
SLC25A29	turquoise	0.317195924	3.96E-07	0.502667313	4.30E-17
SLC25A3	turquoise	-0.345298288	2.88E-08	-0.597193536	4.54E-25
SLC25A34	turquoise	0.214636808	0.000719819	0.648453594	1.28E-30
SLC25A4	turquoise	-0.329284115	1.32E-07	-0.726758857	1.62E-41
SLC25A44	turquoise	-0.301788754	1.49E-06	-0.522059259	1.57E-18
SLC25A46	turquoise	-0.280479539	8.29E-06	-0.606652328	5.10E-26
SLC26A6	turquoise	0.223067684	0.000434586	0.590418124	2.08E-24
SLC27A1	turquoise	0.278382963	9.74E-06	0.443668973	3.08E-13
SLC2A1	turquoise	0.3777643	9.92E-10	0.441838985	3.95E-13
SLC2A13	turquoise	-0.21015667	0.000933999	-0.254446375	5.60E-05
SLC35B1	turquoise	-0.272255404	1.55E-05	-0.634594585	5.13E-29
SLC39A1	turquoise	0.428470911	2.32E-12	0.394690293	1.47E-10
SLC48A1	turquoise	0.254695051	5.51E-05	0.522628611	1.42E-18
SLC4A11	turquoise	0.424098788	4.08E-12	0.340404948	4.63E-08
SLC4A1AP	turquoise	-0.282592897	7.04E-06	-0.498088146	9.12E-17

SLC6A12	turquoise	0.447423682	1.84E-13	0.528009722	5.44E-19
SLC6A15	turquoise	-0.279701187	8.80E-06	-0.688621279	8.88E-36
SLC6A16	turquoise	0.262560385	3.16E-05	0.325214586	1.92E-07
SLC7A14	turquoise	-0.136609815	0.032567353	-0.186969644	0.003308497
SLC7A2	turquoise	0.439974275	5.08E-13	0.532179431	2.56E-19
SLC7A5	turquoise	0.223489589	0.000423538	0.520947796	1.91E-18
SLC7A9	turquoise	0.315064282	4.78E-07	0.412361257	1.78E-11
SLC8A3	turquoise	-0.296065626	2.40E-06	-0.241928268	0.000131104
SLC9A3R1	turquoise	0.240932968	0.000140004	0.471777685	5.55E-15
SLC9A6	turquoise	-0.292677069	3.16E-06	-0.480217223	1.54E-15
SLCO2B1	turquoise	0.336356696	6.82E-08	0.363885007	4.38E-09
SLCO4A1	turquoise	0.356008511	9.88E-09	0.363722053	4.46E-09
SLITRK1	turquoise	-0.296912536	2.24E-06	-0.757477806	6.75E-47
SLITRK3	turquoise	-0.139840877	0.028638565	-0.294905094	2.64E-06
SLITRK4	turquoise	-0.367947797	2.86E-09	-0.606328177	5.50E-26
SLITRK5	turquoise	-0.202341303	0.001452832	-0.559272332	1.46E-21
SMAD6	turquoise	0.281796244	7.49E-06	0.42267923	4.89E-12
SMAD9	turquoise	0.268008313	2.12E-05	0.351482046	1.56E-08
SMARCC1	turquoise	0.355950544	9.93E-09	0.429485917	2.04E-12
SMG5	turquoise	0.310979098	6.82E-07	0.496410415	1.20E-16
SMO	turquoise	0.337600014	6.06E-08	0.49103492	2.84E-16
SMOC1	turquoise	0.216740888	0.000635771	0.4425324	3.59E-13
SMOX	turquoise	0.327914901	1.50E-07	0.562863714	7.09E-22
SMPX	turquoise	-0.14140361	0.026888722	-0.272876616	1.48E-05
SMYD2	turquoise	-0.308192454	8.67E-07	-0.539075641	7.18E-20
SMYD3	turquoise	-0.208937114	0.001001712	-0.633340256	7.10E-29
SNAP25	turquoise	-0.458652993	3.79E-14	-0.525686703	8.25E-19
SNAP91	turquoise	-0.335245909	7.57E-08	-0.356612839	9.29E-09
SNCA	turquoise	-0.302840589	1.37E-06	-0.740461918	8.02E-44
SNRPN	turquoise	-0.331653778	1.06E-07	-0.402105089	6.16E-11
SNTB1	turquoise	0.289612785	4.04E-06	0.28725387	4.88E-06
SNX10	turquoise	-0.358818806	7.41E-09	-0.606515313	5.27E-26
SNX22	turquoise	0.212363309	0.000822084	0.451281574	1.07E-13
SORBS2	turquoise	-0.291576206	3.45E-06	-0.453674111	7.68E-14
SOSTDC1	turquoise	-0.071504689	0.264880686	-0.30719888	9.44E-07
SOX13	turquoise	0.29978306	1.76E-06	0.555197955	3.27E-21
SP1	turquoise	0.342922376	3.63E-08	0.506554846	2.25E-17
SP100	turquoise	0.248345814	8.53E-05	0.18975964	0.002862213
SP110	turquoise	0.210562548	0.000912415	0.345366244	2.86E-08
SPARC	turquoise	0.231540686	0.000256731	0.064633836	0.313665891

SPARCL1	turquoise	-0.198644217	0.001780655	-0.538569716	7.89E-20
SPATA13	turquoise	0.392604874	1.87E-10	0.368685508	2.64E-09
SPEN	turquoise	0.351107896	1.62E-08	0.575807362	4.90E-23
SPINT2	turquoise	-0.257091173	4.66E-05	-0.408829369	2.74E-11
SPOCK1	turquoise	-0.293585465	2.94E-06	-0.386365289	3.81E-10
SPSB2	turquoise	0.212743166	0.000804115	0.162661256	0.010771172
SRGAP1	turquoise	0.393894774	1.61E-10	0.606784006	4.95E-26
SRGN	turquoise	0.301757964	1.50E-06	0.086235068	0.178497076
SRP54	turquoise	-0.243314689	0.000119586	-0.629708915	1.80E-28
SRPK2	turquoise	-0.276526704	1.12E-05	-0.485413865	6.90E-16
SRRM2	turquoise	0.223091057	0.000433967	0.567956258	2.51E-22
SSH3	turquoise	0.248489023	8.44E-05	0.499034269	7.82E-17
SST	turquoise	-0.353180341	1.31E-08	-0.610687015	1.96E-26
SSTR1	turquoise	-0.263497444	2.95E-05	-0.272850944	1.48E-05
SSX2IP	turquoise	-0.244980943	0.000106999	-0.429298522	2.09E-12
ST6GALNAC5	turquoise	-0.325515275	1.87E-07	-0.466655817	1.19E-14
ST8SIA3	turquoise	-0.268273019	2.08E-05	-0.261502075	3.40E-05
STAM	turquoise	-0.25085725	7.18E-05	-0.64042682	1.11E-29
STAMBPL1	turquoise	-0.26285107	3.09E-05	-0.616608604	4.72E-27
STARD3	turquoise	0.203240064	0.001381981	0.470320767	6.89E-15
STARD8	turquoise	0.267996307	2.12E-05	0.632914503	7.93E-29
STAT3	turquoise	0.27958267	8.88E-06	0.246860679	9.43E-05
STAT4	turquoise	-0.366142808	3.46E-09	-0.621854976	1.30E-27
STAT5A	turquoise	0.209685341	0.000959653	0.382511303	5.87E-10
STAU2	turquoise	-0.245785702	0.000101375	-0.552620439	5.42E-21
STEAP2	turquoise	-0.265569516	2.54E-05	-0.572857171	9.11E-23
STEAP3	turquoise	0.386256275	3.86E-10	0.427743598	2.55E-12
STH	turquoise	0.227894582	0.000322763	0.450267442	1.24E-13
STK38	turquoise	0.347859979	2.24E-08	0.59115988	1.76E-24
STMN2	turquoise	-0.471502193	5.78E-15	-0.613821644	9.27E-27
STMN3	turquoise	-0.340720184	4.49E-08	-0.290358577	3.81E-06
STMN4	turquoise	-0.285576648	5.57E-06	-0.317932542	3.71E-07
STON2	turquoise	0.345099233	2.93E-08	0.229271202	0.000296167
STX12	turquoise	-0.224025531	0.000409879	-0.501784449	4.98E-17
STXBP5	turquoise	-0.221012832	0.00049232	-0.427519035	2.63E-12
SUB1	turquoise	-0.323785837	2.19E-07	-0.589606543	2.49E-24
SUCLA2	turquoise	-0.31415702	5.17E-07	-0.714208682	1.59E-39
SULT4A1	turquoise	-0.359156296	7.16E-09	-0.514659729	5.69E-18
SUSD1	turquoise	-0.201568042	0.001516436	-0.504422112	3.22E-17
SV2A	turquoise	-0.229118501	0.000299013	-0.090791058	0.15655168

SV2B	turquoise	-0.379134491	8.54E-10	-0.539342537	6.83E-20
SVIL	turquoise	0.304101371	1.23E-06	0.320089919	3.06E-07
SYDE1	turquoise	0.372482739	1.76E-09	0.407878543	3.08E-11
SYMPK	turquoise	0.049832801	0.437453647	0.281237256	7.82E-06
SYN2	turquoise	-0.316149121	4.34E-07	-0.615115704	6.78E-27
SYNGR1	turquoise	-0.545108774	2.30E-20	-0.274248237	1.33E-05
SYNJ1	turquoise	-0.348333056	2.13E-08	-0.401127256	6.91E-11
SYNPR	turquoise	-0.221024807	0.000491964	-0.454153349	7.18E-14
SYT1	turquoise	-0.36226014	5.19E-09	-0.745541666	1.03E-44
SYT13	turquoise	-0.354691888	1.13E-08	-0.540134706	5.89E-20
SYT4	turquoise	-0.382118528	6.14E-10	-0.564620041	4.97E-22
SYTL4	turquoise	0.414025261	1.45E-11	0.55640666	2.58E-21
TAC1	turquoise	-0.328956491	1.36E-07	-0.497573126	9.92E-17
TAF1	turquoise	0.23676956	0.000183727	0.510172196	1.22E-17
TAF4	turquoise	0.347268103	2.37E-08	0.422243919	5.17E-12
TAF6L	turquoise	0.205762752	0.00119969	0.457913444	4.21E-14
TAGLN	turquoise	0.332884002	9.46E-08	0.460120233	3.07E-14
TAGLN2	turquoise	0.090081916	0.159827026	0.010619582	0.868646986
TAGLN3	turquoise	-0.393013516	1.79E-10	-0.702761885	8.46E-38
TAP1	turquoise	0.238596007	0.000163173	0.435989056	8.65E-13
TARBP1	turquoise	-0.29312986	3.05E-06	-0.368471106	2.70E-09
TBC1D14	turquoise	0.389664201	2.62E-10	0.310705132	6.98E-07
TBC1D16	turquoise	0.180266044	0.004648928	0.370257939	2.24E-09
TBC1D19	turquoise	-0.077727002	0.225423227	-0.317976495	3.69E-07
TBC1D2	turquoise	0.305572733	1.08E-06	0.293943364	2.85E-06
TBC1D2B	turquoise	0.363814394	4.42E-09	0.394094436	1.58E-10
TBC1D7	turquoise	-0.197747716	0.00186972	-0.447088916	1.92E-13
TBPL1	turquoise	-0.25890419	4.10E-05	-0.487511589	4.96E-16
TBX2	turquoise	0.369822905	2.34E-09	0.446746167	2.02E-13
TBX3	turquoise	0.376872463	1.09E-09	0.462717822	2.11E-14
TCAP	turquoise	0.243755589	0.000116127	0.515790039	4.68E-18
TCEAL1	turquoise	-0.31767478	3.79E-07	-0.731401021	2.78E-42
TCEAL2	turquoise	-0.443331738	3.22E-13	-0.538286706	8.31E-20
TCEAL7	turquoise	-0.344355381	3.16E-08	-0.825833878	2.17E-62
TCF3	turquoise	0.134306516	0.035640372	0.389355836	2.72E-10
TCF7L2	turquoise	0.15480659	0.015293493	0.273751332	1.38E-05
TCIRG1	turquoise	0.224934538	0.000387644	0.330215781	1.21E-07
TCL1B	turquoise	0.240969189	0.00013967	0.478296014	2.07E-15
TCTEX1D2	turquoise	-0.320009596	3.08E-07	-0.611074058	1.79E-26
TEAD2	turquoise	0.479892985	1.62E-15	0.541550148	4.52E-20

TERF2IP	turquoise	-0.388408479	3.03E-10	-0.528638968	4.86E-19
TFB2M	turquoise	-0.265685646	2.52E-05	-0.473046957	4.59E-15
TFPI	turquoise	0.323093886	2.33E-07	0.293646002	2.92E-06
TGFB1I1	turquoise	0.368835627	2.60E-09	0.34139747	4.21E-08
TGIF1	turquoise	0.328032174	1.49E-07	0.417908723	8.94E-12
TGM2	turquoise	0.207866444	0.001064852	0.249644159	7.81E-05
THAP10	turquoise	-0.239095908	0.000157935	-0.399772924	8.12E-11
THNSL1	turquoise	-0.227219879	0.000336594	-0.550469742	8.23E-21
THOC7	turquoise	-0.351609098	1.54E-08	-0.759182764	3.22E-47
THYN1	turquoise	-0.319849101	3.12E-07	-0.769148027	3.71E-49
TIAM2	turquoise	-0.07851703	0.220728894	-0.25277373	6.29E-05
TIMM23	turquoise	-0.322152996	2.54E-07	-0.561840163	8.72E-22
TIMP1	turquoise	0.254886756	5.44E-05	0.284473062	6.08E-06
TJP1	turquoise	0.363785015	4.43E-09	0.439045116	5.75E-13
TJP2	turquoise	0.397331809	1.08E-10	0.250956931	7.13E-05
TLN2	turquoise	-0.309620983	7.67E-07	-0.338704655	5.45E-08
TLR5	turquoise	0.367790459	2.91E-09	0.368810058	2.61E-09
TM2D2	turquoise	-0.29714227	2.19E-06	-0.586318763	5.12E-24
TM2D3	turquoise	-0.356168435	9.72E-09	-0.621939262	1.27E-27
TM4SF18	turquoise	0.350245362	1.76E-08	0.32042144	2.97E-07
TM9SF2	turquoise	-0.222004619	0.00046362	-0.581931465	1.33E-23
TMEFF2	turquoise	-0.198395125	0.001805005	-0.386698286	3.67E-10
TMEM119	turquoise	0.289829018	3.97E-06	0.156902751	0.01394784
TMEM126A	turquoise	-0.269478799	1.90E-05	-0.568526799	2.24E-22
TMEM132D	turquoise	-0.361931768	5.37E-09	-0.498553542	8.46E-17
TMEM14A	turquoise	-0.311514038	6.51E-07	-0.723574066	5.32E-41
TMEM14B	turquoise	-0.310688963	6.99E-07	-0.473265624	4.44E-15
TMEM155	turquoise	-0.217316792	0.000614402	-0.587657231	3.82E-24
TMEM17	turquoise	-0.316629384	4.16E-07	-0.362674557	4.97E-09
TMEM176A	turquoise	0.226249249	0.00035746	0.297284604	2.17E-06
TMEM200A	turquoise	-0.198475885	0.001797077	-0.449400808	1.40E-13
TMEM216	turquoise	0.375174742	1.32E-09	0.70059409	1.76E-37
TMEM219	turquoise	0.199378435	0.001710618	0.422702966	4.88E-12
TMEM38B	turquoise	0.337119177	6.34E-08	0.371392204	1.98E-09
TMEM59	turquoise	-0.173310241	0.006538643	-0.390662036	2.34E-10
TMEM59L	turquoise	-0.291368836	3.51E-06	0.056746269	0.376485681
TMEM97	turquoise	-0.210927052	0.000893425	-0.28851412	4.41E-06
TMOD1	turquoise	-0.275254649	1.24E-05	-0.496461963	1.19E-16
TMPRSS5	turquoise	0.237809916	0.000171741	0.233806492	0.000222287
TMSB10	turquoise	-0.188776009	0.003012905	-0.40924878	2.61E-11

TNFRSF10B	turquoise	0.382943389	5.60E-10	0.622611393	1.08E-27
TNFRSF1A	turquoise	0.300728642	1.63E-06	0.386824628	3.62E-10
TNFRSF1B	turquoise	0.249881753	7.68E-05	0.518556417	2.90E-18
TNFRSF21	turquoise	-0.230349115	0.000276782	-0.406908649	3.46E-11
TNIP1	turquoise	0.392347354	1.93E-10	0.488567462	4.20E-16
TNS1	turquoise	0.423910312	4.18E-12	0.499492058	7.25E-17
TOB1	turquoise	0.315002991	4.80E-07	0.363573397	4.53E-09
TOB2	turquoise	0.100532456	0.116528398	0.490030036	3.33E-16
TOMM20	turquoise	-0.451051537	1.11E-13	-0.672009114	1.51E-33
TOR1A	turquoise	-0.180201917	0.004663826	-0.654865083	2.17E-31
TP53INP1	turquoise	0.371274356	2.01E-09	0.590810694	1.90E-24
TPM1	turquoise	-0.272608985	1.51E-05	-0.499389167	7.38E-17
TPM2	turquoise	0.310285607	7.24E-07	0.48544153	6.87E-16
TRAP1	turquoise	-0.173629371	0.006438802	-0.513107144	7.43E-18
TRAPPC6B	turquoise	-0.289886309	3.96E-06	-0.268781815	2.01E-05
TRIM36	turquoise	-0.295663128	2.48E-06	-0.398082728	9.90E-11
TRIM37	turquoise	-0.284539926	6.04E-06	-0.740782443	7.05E-44
TRIM47	turquoise	0.219698741	0.000532885	0.127940646	0.045438153
TRIM56	turquoise	0.356266506	9.62E-09	0.555619738	3.01E-21
TRIOBP	turquoise	0.241510862	0.00013477	0.239427108	0.000154551
TRIP10	turquoise	0.387183503	3.48E-10	0.253340034	6.05E-05
TRIP6	turquoise	0.287926623	4.63E-06	0.437243709	7.32E-13
TRO	turquoise	-0.225297139	0.000379091	-0.169567507	0.007817148
TSPAN1	turquoise	-0.258258233	4.29E-05	-0.231020336	0.000265315
TSPAN13	turquoise	-0.28349693	6.56E-06	-0.681110738	9.45E-35
TSPAN4	turquoise	0.190770363	0.002714524	0.582656404	1.14E-23
TSPAN5	turquoise	-0.111425639	0.081755128	-0.106983685	0.094762331
TSPAN7	turquoise	-0.477310577	2.41E-15	-0.726905733	1.53E-41
TSPO	turquoise	0.45659982	5.08E-14	0.64946205	9.69E-31
TSPYL1	turquoise	-0.350227067	1.77E-08	-0.7616536	1.09E-47
TSPYL5	turquoise	-0.243232155	0.000120244	-0.519136389	2.62E-18
TTC19	turquoise	-0.282490563	7.09E-06	-0.610741775	1.94E-26
TTC23	turquoise	0.304472694	1.19E-06	0.516740157	3.98E-18
TTC38	turquoise	0.368403194	2.72E-09	0.548732535	1.15E-20
TTLL3	turquoise	0.196161407	0.00203734	0.153192065	0.016406088
TUBA1B	turquoise	-0.23043571	0.000275277	-0.411241838	2.04E-11
TUBA4A	turquoise	-0.367831454	2.89E-09	-0.282448486	7.12E-06
TUBB	turquoise	-0.121445539	0.05766527	-0.217088569	0.00062279
TUBB2A	turquoise	-0.468853674	8.57E-15	-0.650309008	7.67E-31
TUBB3	turquoise	-0.323703391	2.21E-07	-0.456254054	5.33E-14

TUBG1	turquoise	-0.319064586	3.35E-07	-0.160778244	0.011731354
TUSC3	turquoise	-0.31996859	3.09E-07	-0.67914338	1.74E-34
TXNDC9	turquoise	-0.278219545	9.86E-06	-0.3771178	1.07E-09
TXNL1	turquoise	-0.193546869	0.002343735	-0.308464019	8.47E-07
UBE2E2	turquoise	-0.275971964	1.17E-05	-0.494454989	1.64E-16
UBE2E3	turquoise	-0.29243902	3.22E-06	-0.693992524	1.57E-36
UBE2N	turquoise	-0.351443484	1.57E-08	-0.603156378	1.15E-25
UBE2T	turquoise	-0.266109677	2.44E-05	-0.503209444	3.93E-17
UBE2V2	turquoise	-0.364097331	4.29E-09	-0.625786698	4.87E-28
UBL3	turquoise	-0.25516868	5.33E-05	-0.442169986	3.77E-13
UBL5	turquoise	-0.198061864	0.001838058	-0.215168736	0.000697653
UBLCP1	turquoise	-0.329846244	1.26E-07	-0.321167338	2.77E-07
UBQLN1	turquoise	-0.332471668	9.83E-08	-0.630952213	1.31E-28
UBQLN2	turquoise	-0.357838929	8.19E-09	-0.503077008	4.02E-17
UCHL1	turquoise	-0.330661932	1.16E-07	-0.731150124	3.06E-42
UCHL5	turquoise	-0.279426863	8.99E-06	-0.668593037	4.18E-33
UGP2	turquoise	-0.24577825	0.000101425	-0.701053761	1.51E-37
UHRF1	turquoise	0.371795845	1.90E-09	0.481004772	1.37E-15
UNC5B	turquoise	0.178010205	0.005199549	0.155910001	0.014571621
UNKL	turquoise	0.361660587	5.53E-09	0.420834058	6.18E-12
UPF3A	turquoise	0.16127627	0.01147033	0.526771794	6.80E-19
UQCRC2	turquoise	-0.392320379	1.93E-10	-0.713732838	1.88E-39
UQCRFS1	turquoise	-0.260928082	3.55E-05	-0.637897857	2.16E-29
VAMP1	turquoise	-0.241753351	0.000132628	-0.374203895	1.46E-09
VAMP5	turquoise	0.316958187	4.04E-07	0.349757656	1.85E-08
VAPA	turquoise	-0.395408612	1.35E-10	-0.276873635	1.09E-05
VASH1	turquoise	0.259830893	3.84E-05	0.480476712	1.48E-15
VASN	turquoise	0.226791636	0.000345656	0.335589753	7.33E-08
VAT1L	turquoise	-0.190768389	0.002714806	-0.464420782	1.64E-14
VAV2	turquoise	0.225920871	0.000364787	0.528665827	4.84E-19
VBP1	turquoise	-0.280105118	8.53E-06	-0.584130175	8.26E-24
VCAM1	turquoise	0.131743314	0.03934571	0.177930164	0.005220119
VDAC1	turquoise	-0.388290489	3.07E-10	-0.804812218	5.60E-57
VGF	turquoise	-0.449488677	1.38E-13	-0.392464959	1.90E-10
VIP	turquoise	-0.3873027	3.43E-10	-0.435254875	9.54E-13
VKORC1L1	turquoise	-0.308393477	8.52E-07	-0.563045037	6.84E-22
VLDLR	turquoise	-0.23290937	0.000235374	-0.527872229	5.58E-19
VPS29	turquoise	-0.228754444	0.000305902	-0.707272049	1.81E-38
VPS35	turquoise	-0.280229248	8.45E-06	-0.576070847	4.64E-23
VSNL1	turquoise	-0.360714827	6.10E-09	-0.588424777	3.23E-24

VTA1	turquoise	-0.30176347	1.50E-06	-0.462423778	2.20E-14
WAS	turquoise	0.194312258	0.002249962	0.331738787	1.05E-07
WASF1	turquoise	-0.380917056	7.01E-10	-0.631400679	1.17E-28
WASF2	turquoise	0.215325593	0.000691238	0.304565422	1.18E-06
WASL	turquoise	-0.322764081	2.40E-07	-0.59113362	1.77E-24
WDR20	turquoise	0.319623043	3.19E-07	0.586593821	4.82E-24
WDR37	turquoise	-0.226632492	0.000349082	-0.442654888	3.53E-13
WDR47	turquoise	-0.33503482	7.73E-08	-0.746628893	6.58E-45
WDR54	turquoise	-0.364626352	4.06E-09	-0.48924562	3.77E-16
WDR61	turquoise	-0.283096222	6.77E-06	-0.661763886	3.06E-32
WDR7	turquoise	-0.211876272	0.000845665	-0.630506913	1.47E-28
WWC3	turquoise	0.409784704	2.44E-11	0.52119497	1.83E-18
WWTR1	turquoise	0.320820184	2.86E-07	0.027582434	0.667481376
XAF1	turquoise	0.174401355	0.006202898	0.560368668	1.17E-21
XK	turquoise	-0.295515376	2.51E-06	-0.615165357	6.70E-27
YARS2	turquoise	-0.143863531	0.024320246	-0.497719467	9.69E-17
YPEL5	turquoise	-0.266505642	2.37E-05	-0.635441431	4.12E-29
YTHDF2	turquoise	-0.34333251	3.49E-08	-0.598456891	3.40E-25
YWHAB	turquoise	-0.424119802	4.07E-12	-0.611154153	1.76E-26
YWHAG	turquoise	-0.413753636	1.50E-11	-0.664466813	1.40E-32
YWHAH	turquoise	-0.316639284	4.16E-07	-0.639161809	1.55E-29
YWHAZ	turquoise	-0.393224606	1.74E-10	-0.778896113	3.78E-51
ZBTB20	turquoise	0.420794094	6.22E-12	0.664997843	1.20E-32
ZBTB34	turquoise	0.314384655	5.07E-07	0.318911309	3.40E-07
ZC3H15	turquoise	-0.237634225	0.000173712	-0.64775648	1.54E-30
ZC3HAV1	turquoise	0.272152879	1.56E-05	0.439931303	5.11E-13
ZCCHC12	turquoise	-0.3519432	1.49E-08	-0.686687153	1.64E-35
ZCCHC17	turquoise	-0.267635056	2.18E-05	-0.508365733	1.66E-17
ZDHHC11	turquoise	0.276093085	1.16E-05	0.361866296	5.41E-09
ZDHHC17	turquoise	-0.247967977	8.75E-05	-0.481477432	1.27E-15
ZDHHC23	turquoise	-0.243498956	0.000118129	-0.365888731	3.55E-09
ZFAND1	turquoise	-0.297309742	2.16E-06	-0.597839384	3.92E-25
ZFAND5	turquoise	-0.370136208	2.26E-09	-0.502890539	4.15E-17
ZFP36	turquoise	0.310931995	6.85E-07	0.187170669	0.00327435
ZFP36L2	turquoise	0.269526846	1.90E-05	0.015469105	0.809628021
ZFPM2	turquoise	-0.203333711	0.001374783	-0.400292317	7.63E-11
ZIC2	turquoise	0.229617348	0.000289808	0.365837476	3.57E-09
ZMAT4	turquoise	-0.096173683	0.13331948	-0.055664764	0.385663524
ZMIZ2	turquoise	0.333579503	8.86E-08	0.58817507	3.41E-24
ZMYND15	turquoise	0.254198786	5.70E-05	0.306234955	1.03E-06

ZNF184	turquoise	-0.400555179	7.40E-11	-0.573925143	7.28E-23
ZNF205	turquoise	0.313523151	5.47E-07	0.330519195	1.18E-07
ZNF215	turquoise	-0.332349287	9.95E-08	-0.210406548	0.000920656
ZNF25	turquoise	-0.376342247	1.16E-09	-0.600785111	2.00E-25
ZNF326	turquoise	0.285571484	5.57E-06	0.312900032	5.77E-07
ZNF365	turquoise	-0.363723783	4.46E-09	-0.471257266	6.00E-15
ZNF385B	turquoise	-0.25174909	6.76E-05	-0.655307809	1.91E-31
ZNF395	turquoise	0.391140086	2.22E-10	0.416750389	1.03E-11
ZNF397	turquoise	0.381317744	6.71E-10	0.479168997	1.81E-15
ZNF621	turquoise	0.373447613	1.59E-09	0.678045581	2.43E-34
ZNHIT3	turquoise	-0.238750559	0.000161537	-0.625619932	5.08E-28
ZRANB2	turquoise	-0.306001991	1.05E-06	-0.410123097	2.34E-11
ZWILCH	turquoise	-0.330400609	1.19E-07	-0.465430062	1.42E-14
ZYG11B	turquoise	-0.317476448	3.86E-07	-0.274371029	1.32E-05
ABL1	yellow	0.340303933	4.67E-08	0.798667906	1.61E-55
ADAM17	yellow	0.218565341	0.000570336	0.799877104	8.40E-56
ADH4	yellow	0.048086061	0.453706363	0.664546106	1.37E-32
AJAP1	yellow	0.192199396	0.002517507	0.404917851	4.40E-11
ARAP2	yellow	0.252480228	6.42E-05	0.530344592	3.57E-19
ARID4B	yellow	0.271704824	1.61E-05	0.764905538	2.55E-48
ATG12	yellow	0.169850502	0.007713225	0.761286881	1.28E-47
BLZF1	yellow	0.234117366	0.000217912	0.773079606	6.00E-50
C11orf1	yellow	-0.141976763	0.026270349	-0.561764472	8.85E-22
C21orf58	yellow	0.298539202	1.96E-06	0.81188484	1.01E-58
C9orf64	yellow	0.23413043	0.00021773	0.627619333	3.07E-28
CATSPER2	yellow	0.300063212	1.72E-06	0.636660222	2.99E-29
CCDC125	yellow	0.303444797	1.30E-06	0.775606197	1.83E-50
CD68	yellow	0.259478563	3.93E-05	0.776313422	1.30E-50
CDC42BPA	yellow	0.239354464	0.000155287	0.519046603	2.66E-18
CDK10	yellow	0.319563644	3.20E-07	0.795765562	7.57E-55
CDKN2AIP	yellow	0.219117429	0.000551799	0.676373516	4.05E-34
CENPB	yellow	0.283126734	6.75E-06	0.742497811	3.54E-44
CETN3	yellow	0.189700825	0.002871028	0.630280662	1.56E-28
CHST12	yellow	0.32176105	2.63E-07	0.71519981	1.12E-39
CLCN7	yellow	0.259108231	4.04E-05	0.813038965	5.15E-59
CLK3	yellow	0.241722469	0.000132899	0.743314891	2.54E-44
CLN8	yellow	0.327011746	1.63E-07	0.490901323	2.90E-16
CNNM3	yellow	0.335670176	7.28E-08	0.77645704	1.22E-50
CRCP	yellow	0.186945838	0.003312562	0.668542083	4.24E-33
CTSB	yellow	0.273590766	1.40E-05	0.783276174	4.46E-52

DBT	yellow	0.180904248	0.004502977	0.7334475	1.26E-42
DCLRE1C	yellow	0.278872112	9.38E-06	0.917236585	4.06E-99
DDX27	yellow	0.276607137	1.12E-05	0.84573405	3.13E-68
DENND4C	yellow	0.370859651	2.10E-09	0.768471648	5.06E-49
DENR	yellow	0.295321945	2.55E-06	0.922707758	1.40E-102
DFFA	yellow	0.344119985	3.23E-08	0.822290008	1.99E-61
DPP7	yellow	0.204892342	0.001259931	0.728395874	8.74E-42
DSEL	yellow	0.21740767	0.000611091	0.808532647	6.91E-58
DTWD2	yellow	0.259424212	3.95E-05	0.833362473	1.65E-64
EIF2AK4	yellow	0.257990452	4.37E-05	0.884324548	2.38E-82
ELAVL3	yellow	0.095686882	0.135304768	0.724346296	3.99E-41
ERCC5	yellow	0.318452614	3.54E-07	0.711933234	3.56E-39
ERGIC1	yellow	0.313955798	5.26E-07	0.779976785	2.24E-51
EVI5	yellow	0.203595004	0.001354883	0.84169732	5.55E-67
FAM43A	yellow	0.176883384	0.005495948	0.407285413	3.31E-11
FASTK	yellow	0.021098934	0.74245939	0.611078555	1.79E-26
FCF1	yellow	0.195988713	0.002056392	0.61999886	2.06E-27
FXR1	yellow	0.207873091	0.001064449	0.371320471	2.00E-09
GGA3	yellow	0.260556602	3.64E-05	0.532369175	2.47E-19
GLO1	yellow	-0.313428916	5.51E-07	-0.799463822	1.05E-55
GNB4	yellow	0.180822556	0.004521426	0.540274918	5.74E-20
GNL3L	yellow	0.20997965	0.000943559	0.780196012	2.02E-51
GPSM1	yellow	0.215623262	0.000679214	0.71294795	2.49E-39
GSDMB	yellow	0.312525924	5.96E-07	0.785158566	1.75E-52
HMGN2	yellow	-0.317788359	3.75E-07	-0.414111323	1.43E-11
HSCB	yellow	0.253397824	6.03E-05	0.80253949	1.97E-56
HYDIN	yellow	0.40683087	3.49E-11	0.710772689	5.35E-39
IFNAR2	yellow	0.236856414	0.000182697	0.689574724	6.55E-36
IL17RD	yellow	0.201297482	0.001539285	0.717994733	4.10E-40
ITSN1	yellow	0.456391849	5.23E-14	0.814478497	2.21E-59
JAKMIP3	yellow	0.362230821	5.21E-09	0.756852088	8.85E-47
KAT2A	yellow	0.291888043	3.37E-06	0.705095926	3.82E-38
KCNK12	yellow	0.437850459	6.75E-13	0.695367263	9.98E-37
KDSR	yellow	0.242578691	0.000125577	0.596687217	5.09E-25
KHDRBS1	yellow	-0.297721591	2.09E-06	-0.794381343	1.57E-54
KHSRP	yellow	0.106613645	0.095916353	0.648963525	1.11E-30
KLKB1	yellow	0.300961431	1.60E-06	0.501935987	4.86E-17
LCOR	yellow	0.042999345	0.502912529	0.528703457	4.80E-19
LLPH	yellow	0.269086169	1.96E-05	0.83402582	1.06E-64
LRCH4	yellow	0.301546502	1.52E-06	0.748875121	2.60E-45

LRP5	yellow	0.351444108	1.57E-08	0.763730516	4.32E-48
MAFF	yellow	0.279652895	8.84E-06	0.352654941	1.39E-08
MAX	yellow	0.208913618	0.00100306	0.67365583	9.23E-34
METTL2B	yellow	0.215262753	0.000693802	0.628832349	2.26E-28
MLXIPL	yellow	0.215316119	0.000691624	0.65480687	2.20E-31
NFIC	yellow	0.210760354	0.000902064	0.72014914	1.87E-40
NOP14	yellow	0.143677878	0.024506433	0.750304315	1.43E-45
NOTCH1	yellow	0.405339988	4.18E-11	0.738162255	2.00E-43
NR1H3	yellow	0.298144975	2.02E-06	0.669251381	3.44E-33
NUBPL	yellow	0.275855396	1.18E-05	0.799395588	1.09E-55
OPA3	yellow	0.251657804	6.80E-05	0.778302382	5.04E-51
PAPOLA	yellow	0.306623891	9.92E-07	0.634625219	5.09E-29
PDCD4	yellow	0.242706085	0.00012452	0.8627925	6.15E-74
PHAX	yellow	0.147627458	0.020799848	0.449744537	1.33E-13
PHF21A	yellow	0.344780534	3.03E-08	0.790740419	1.04E-53
PIAS4	yellow	0.283789355	6.41E-06	0.788934468	2.62E-53
PIGX	yellow	0.270323042	1.79E-05	0.760461629	1.84E-47
PLA2G2D	yellow	0.245398527	0.000104045	0.859142423	1.18E-72
PLEKHO1	yellow	0.276590772	1.12E-05	0.726770407	1.61E-41
POLR1B	yellow	0.233562152	0.000225782	0.681779099	7.68E-35
PPFIBP1	yellow	0.09922257	0.121391078	0.538804401	7.55E-20
PPP1R16A	yellow	0.417767358	9.10E-12	0.723601626	5.26E-41
PPP2R3A	yellow	0.188277143	0.003092049	0.798292694	1.97E-55
PRMT2	yellow	0.315747602	4.50E-07	0.759916004	2.33E-47
PRR14	yellow	0.246183393	9.87E-05	0.844309665	8.71E-68
PTGR2	yellow	0.229463705	0.000292615	0.588705846	3.03E-24
RAB11FIP3	yellow	0.151425627	0.017703711	0.648991827	1.10E-30
RAD51	yellow	0.211975059	0.000840832	0.819292414	1.25E-60
RAX2	yellow	0.186240308	0.003435107	0.685275147	2.57E-35
RBM39	yellow	0.207265975	0.001101844	0.709173233	9.36E-39
RBM6	yellow	0.44900419	1.48E-13	0.834561872	7.41E-65
RFX4	yellow	0.274748936	1.28E-05	0.708070249	1.37E-38
RNF213	yellow	0.255678949	5.14E-05	0.864837268	1.13E-74
RNF216	yellow	0.333869892	8.62E-08	0.725240534	2.86E-41
RPS6KB2	yellow	0.143181577	0.0250102	0.71261372	2.80E-39
RYBP	yellow	0.185129159	0.003636437	0.413611336	1.53E-11
SCD5	yellow	-0.194699891	0.002203785	-0.452642655	8.88E-14
SCRIB	yellow	0.382819113	5.68E-10	0.85635737	1.07E-71
SLC12A9	yellow	0.278685211	9.52E-06	0.717661094	4.62E-40
SLC38A2	yellow	0.376674195	1.12E-09	0.443800477	3.02E-13

SLC5A3	yellow	0.388100232	3.13E-10	0.705782845	3.02E-38
SLCO3A1	yellow	0.280418982	8.33E-06	0.765342926	2.09E-48
SNAPC1	yellow	0.200294801	0.001626741	0.776447821	1.22E-50
SORBS1	yellow	0.347366253	2.35E-08	0.783345307	4.31E-52
SPCS1	yellow	-0.413528349	1.54E-11	-0.772668774	7.28E-50
SPOP	yellow	0.237361902	0.00017681	0.698455142	3.59E-37
STK36	yellow	0.331522515	1.07E-07	0.721092444	1.33E-40
SYNJ2BP	yellow	0.30029561	1.69E-06	0.913954683	3.73E-97
THOC2	yellow	0.363251757	4.68E-09	0.845214858	4.55E-68
TMEM91	yellow	0.214454683	0.000727557	0.683223634	4.90E-35
TNFSF14	yellow	0.244046681	0.000113894	0.873594412	5.77E-78
TP53I13	yellow	0.319759951	3.15E-07	0.792531081	4.12E-54
TRABD	yellow	0.383363967	5.34E-10	0.855128199	2.77E-71
TSC22D1	yellow	-0.298694369	1.93E-06	-0.669208047	3.48E-33
TSPAN31	yellow	0.211260587	0.000876367	0.645973361	2.51E-30
TSPAN33	yellow	0.226173417	0.00035914	0.611877496	1.48E-26
TUBA1C	yellow	-0.336804439	6.53E-08	-0.80051434	5.95E-56
TXLNA	yellow	0.384629003	4.64E-10	0.838018759	7.13E-66
U2AF2	yellow	0.237721047	0.000172736	0.637733426	2.26E-29
UBE2H	yellow	0.271935168	1.59E-05	0.386469542	3.77E-10
UBXN2A	yellow	0.315232295	4.71E-07	0.897559625	2.13E-88
USP49	yellow	0.25552459	5.20E-05	0.715714692	9.30E-40
VGLL4	yellow	0.360897977	5.98E-09	0.742988644	2.90E-44
WWOX	yellow	0.340780912	4.46E-08	0.810859493	1.82E-58
XRCC2	yellow	0.151476819	0.017664884	0.353371964	1.29E-08
YY1	yellow	0.381325055	6.70E-10	0.494946606	1.52E-16
ZDHHC8	yellow	0.334765838	7.93E-08	0.818382863	2.16E-60
ZHX3	yellow	0.450191403	1.25E-13	0.765963602	1.58E-48
ZNF148	yellow	0.283393579	6.61E-06	0.723527717	5.41E-41
ZNF160	yellow	0.345485604	2.83E-08	0.763078907	5.77E-48
ZNF223	yellow	0.211373036	0.000870684	0.77384458	4.19E-50
ZNF320	yellow	0.15514756	0.015067155	0.650557768	7.17E-31
ZNF462	yellow	0.317026469	4.02E-07	0.762080983	8.99E-48
ZNF652	yellow	0.348756872	2.05E-08	0.829279021	2.39E-63
ZNF669	yellow	0.225721318	0.000369308	0.806440018	2.26E-57
ZNF672	yellow	0.314290683	5.11E-07	0.698764361	3.24E-37
ZNF682	yellow	0.254407435	5.62E-05	0.629363319	1.97E-28
ZNF738	yellow	0.25279178	6.29E-05	0.742828733	3.10E-44
ZNF771	yellow	0.347813646	2.25E-08	0.85575104	1.71E-71
ZNF777	yellow	0.173762736	0.006397484	0.554147302	4.02E-21

ZNF786	yellow	0.33653914	6.70E-08	0.915130733	7.54E-98
ZNF787	yellow	0.243748706	0.00011618	0.730520167	3.90E-42
ZNF827	yellow	0.127752392	0.045759234	0.668185753	4.71E-33
ZNF93	yellow	0.208271111	0.00104057	0.763573217	4.63E-48

MMbrown	p.MMbrown	MMgreen	p.MMgreen	MMblue	p.MMblue
-0.468969101	8.42E-15	-0.445472738	2.40E-13	0.674037898	8.23E-34
-0.088711297	0.16630311	-0.023776313	0.711153542	0.369011634	2.55E-09
-0.265714765	2.51E-05	-0.141686062	0.026582442	0.693106764	2.09E-36
-0.13751998	0.03141677	-0.114240093	0.074286728	0.738126601	2.03E-43
-0.3976382	1.04E-10	-0.100668665	0.116031607	0.743735376	2.14E-44
-0.123407151	0.05371482	-0.079439857	0.215334192	0.396335987	1.21E-10
-0.424542704	3.86E-12	-0.276118092	1.16E-05	0.858779191	1.58E-72
0.06656187	0.299418516	0.064643786	0.313591246	-0.450282059	1.24E-13
-0.477411097	2.37E-15	-0.237142867	0.000179339	0.848103139	5.56E-69
-0.464057933	1.73E-14	-0.247744279	8.88E-05	0.928814413	9.31E-107
-0.305794842	1.06E-06	-0.040680636	0.526238307	0.639941843	1.26E-29
-0.288824237	4.31E-06	-0.152265446	0.017076037	0.710377312	6.15E-39
-0.280120839	8.52E-06	0.144315089	0.023872468	0.86901974	3.24E-76
-0.388908848	2.86E-10	-0.233331402	0.00022913	0.834197969	9.45E-65
-0.624727197	6.36E-28	-0.523091596	1.31E-18	0.736286233	4.19E-43
-0.551265136	7.05E-21	-0.318124328	3.64E-07	0.784314738	2.67E-52
-0.392242379	1.95E-10	-0.202446305	0.001444386	0.881634467	3.28E-81
-0.362460309	5.09E-09	-0.182788837	0.004095757	0.416553071	1.06E-11
-0.230415856	0.000275621	-0.153933164	0.015886901	0.364593842	4.07E-09
-0.35820846	7.89E-09	-0.156604093	0.014132987	0.741328059	5.67E-44
-0.509330091	1.41E-17	-0.242894731	0.000122971	0.875081835	1.51E-78
-0.475117218	3.36E-15	-0.332915134	9.43E-08	0.650737938	6.82E-31
-0.36419167	4.25E-09	-0.271273493	1.67E-05	0.55563414	3.00E-21
-0.069136352	0.2810734	0.089840581	0.160953358	0.741077448	6.27E-44
-0.286282927	5.27E-06	-0.123504956	0.05352388	0.829285322	2.38E-63
-0.304476736	1.19E-06	-0.108455306	0.090281902	0.787330866	5.89E-53
-0.298873675	1.90E-06	-0.245849562	0.00010094	0.740824885	6.93E-44
-0.291681744	3.42E-06	-0.149209492	0.019457415	0.821052009	4.26E-61
-0.507636248	1.88E-17	-0.326357696	1.73E-07	0.832232639	3.48E-64
-0.442539252	3.59E-13	-0.25915233	4.03E-05	0.854429209	4.76E-71
-0.356762847	9.15E-09	-0.156282039	0.01433505	0.8715472	3.57E-77
0.383340966	5.35E-10	0.218374955	0.000576861	-0.476318248	2.80E-15
-0.331846497	1.04E-07	-0.134636055	0.035186177	0.8972316	3.08E-88
-0.186023559	0.003473569	-0.073222576	0.253542711	0.850942147	6.74E-70
-0.441316247	4.23E-13	-0.306572909	9.96E-07	0.80191161	2.78E-56
-0.301261022	1.56E-06	-0.334862186	7.85E-08	0.729649836	5.43E-42
-0.221045243	0.000491357	-0.101617735	0.112615866	0.417536476	9.37E-12
-0.124450671	0.051706502	0.047413153	0.460056685	0.799275835	1.16E-55
-0.561123096	1.01E-21	-0.416308118	1.09E-11	0.776651432	1.11E-50

-0.272881433	1.48E-05	-0.16711809	0.008770018	0.662986776	2.15E-32
-0.320886349	2.85E-07	-0.195715147	0.002086905	0.719325728	2.53E-40
-0.116571122	0.068529323	0.053861048	0.401269139	0.646035999	2.47E-30
-0.434374514	1.07E-12	-0.208398229	0.001033048	0.863712542	2.88E-74
-0.33810985	5.77E-08	-0.153581136	0.016131701	0.741120928	6.16E-44
-0.425766002	3.30E-12	-0.257453837	4.54E-05	0.623611565	8.40E-28
-0.592652502	1.26E-24	-0.471035936	6.20E-15	0.800750363	5.23E-56
-0.340543451	4.57E-08	-0.234937106	0.00020676	0.534218475	1.76E-19
-0.647287231	1.76E-30	-0.361642683	5.54E-09	0.804952081	5.18E-57
-0.27200883	1.58E-05	-0.152475304	0.016922254	0.691928829	3.06E-36
-0.362310458	5.17E-09	-0.175165004	0.005977181	0.826466036	1.45E-62
-0.559535366	1.38E-21	-0.434999138	9.87E-13	0.681672011	7.94E-35
-0.658168391	8.53E-32	-0.553089853	4.94E-21	0.731572537	2.61E-42
-0.618236228	3.17E-27	-0.280470192	8.30E-06	0.68265046	5.86E-35
-0.722402303	8.20E-41	-0.43385952	1.15E-12	0.773069545	6.03E-50
-0.482855436	1.03E-15	-0.272382005	1.53E-05	0.848503829	4.14E-69
-0.308098355	8.74E-07	-0.136320844	0.032940077	0.867314891	1.40E-75
0.337434438	6.15E-08	0.289056016	4.23E-06	-0.664159936	1.53E-32
-0.198912535	0.001754762	-0.332505619	9.80E-08	0.741996932	4.33E-44
-0.410263596	2.30E-11	-0.336480325	6.74E-08	0.844884828	5.77E-68
-0.630014666	1.67E-28	-0.456622906	5.06E-14	0.467497695	1.05E-14
-0.591812666	1.52E-24	-0.185784176	0.003516501	0.794146835	1.77E-54
-0.526823839	6.73E-19	-0.248821345	8.26E-05	0.878790494	4.90E-80
-0.225249707	0.000380199	-0.09750046	0.128022295	0.814095631	2.77E-59
-0.381888984	6.29E-10	-0.224466586	0.000398946	0.563669461	6.03E-22
-0.386974858	3.56E-10	-0.204467189	0.001290353	0.924581983	7.99E-104
-0.210618743	0.000909464	-0.082924847	0.195813893	0.657943991	9.09E-32
-0.313511884	5.47E-07	-0.064246896	0.316577769	0.870020646	1.36E-76
0.405495798	4.10E-11	0.212415308	0.000819603	-0.466401546	1.23E-14
-0.159464346	0.012445284	-0.064495788	0.314702739	0.520302676	2.14E-18
0.218498299	0.000572626	0.213953398	0.000749252	-0.612531495	1.26E-26
0.273165988	1.45E-05	0.069207552	0.28057706	-0.555782461	2.91E-21
-0.267883491	2.14E-05	-0.31593554	4.42E-07	0.485276508	7.05E-16
-0.280537035	8.25E-06	-0.033744728	0.599140387	0.857575185	4.10E-72
-0.440575022	4.68E-13	-0.362323534	5.16E-09	0.630252696	1.57E-28
-0.3378035	5.94E-08	-0.24383978	0.000115477	0.451829124	9.96E-14
-0.375008266	1.34E-09	-0.276911759	1.09E-05	0.776078137	1.46E-50
-0.400961997	7.05E-11	-0.181229005	0.004430303	0.851893931	3.29E-70
-0.567671574	2.67E-22	-0.359399856	6.98E-09	0.728711836	7.76E-42
-0.378918652	8.74E-10	-0.144063554	0.024121012	0.863207092	4.37E-74

-0.236336393	0.000188945	-0.087669957	0.171352995	0.898659969	6.14E-89
-0.180668835	0.004556326	-0.064092131	0.317747359	0.836187941	2.48E-65
-0.34359218	3.40E-08	-0.143522551	0.024663149	0.628267948	2.60E-28
-0.149802142	0.01897425	0.067094534	0.295559211	0.839339057	2.87E-66
-0.221639714	0.000473995	-0.028087251	0.661768332	0.774159284	3.62E-50
-0.612668091	1.22E-26	-0.448997056	1.48E-13	0.745011296	1.28E-44
-0.328012545	1.49E-07	-0.117677703	0.065926655	0.899743904	1.78E-89
-0.088655426	0.166571193	0.071814382	0.262811478	0.761814435	1.01E-47
-0.279340661	9.05E-06	-0.049057205	0.444628804	0.786348469	9.66E-53
-0.258937663	4.09E-05	-0.126827983	0.047363568	0.895832983	1.46E-87
-0.531489442	2.90E-19	-0.213997125	0.000747336	0.8134753	3.99E-59
-0.176497317	0.005600933	-0.27260758	1.51E-05	0.57348643	7.99E-23
-0.478332323	2.06E-15	-0.333250019	9.14E-08	0.549050106	1.08E-20
-0.364290435	4.20E-09	-0.238176987	0.000167689	0.738511858	1.74E-43
-0.295720922	2.47E-06	-0.229501912	0.000291915	0.694183381	1.47E-36
-0.401376834	6.71E-11	-0.378900588	8.76E-10	0.787985973	4.23E-53
-0.234890479	0.00020738	-0.109069213	0.088463632	0.837258756	1.20E-65
0.21671082	0.000636905	0.195884887	0.002067924	-0.365832774	3.57E-09
-0.393160037	1.76E-10	-0.311679926	6.42E-07	0.714974941	1.21E-39
0.035774152	0.57734377	-0.012213564	0.849150895	0.607193448	4.49E-26
-0.237174773	0.000178969	-0.100146021	0.117946874	0.588159296	3.42E-24
0.563328082	6.46E-22	0.477954902	2.18E-15	-0.703322074	7.00E-38
-0.126443854	0.048043922	-0.081010818	0.206368958	0.774154875	3.62E-50
-0.298642096	1.94E-06	-0.150043137	0.018780778	0.905562056	1.80E-92
-0.329693532	1.27E-07	-0.125202359	0.050298852	0.856190649	1.21E-71
-0.642110541	7.09E-30	-0.482038093	1.17E-15	0.750584298	1.27E-45
-0.203388572	0.001370583	-0.184801502	0.003697808	0.714143788	1.63E-39
-0.424666023	3.80E-12	-0.300849902	1.61E-06	0.754209795	2.75E-46
-0.328319968	1.45E-07	-0.288693516	4.35E-06	0.729586533	5.57E-42
-0.152666637	0.0167831	-0.14998671	0.018825923	0.662606733	2.40E-32
-0.360883722	5.99E-09	-0.195666306	0.002092395	0.87002102	1.36E-76
-0.463005194	2.02E-14	-0.297890482	2.06E-06	0.680213083	1.25E-34
-0.588297806	3.32E-24	-0.238154697	0.000167933	0.661907259	2.93E-32
-0.303483269	1.29E-06	-0.209471993	0.000971477	0.722697169	7.35E-41
-0.23722599	0.000178375	-0.021609382	0.736456264	0.814919489	1.71E-59
-0.232029604	0.000248903	0.087700703	0.171202279	0.536164372	1.23E-19
-0.408104763	2.99E-11	-0.360828201	6.02E-09	0.6946981	1.24E-36
-0.395578871	1.33E-10	-0.282939353	6.85E-06	0.81081872	1.87E-58
-0.0912474	0.154470865	0.023016731	0.719988705	0.368201082	2.78E-09
-0.405194539	4.26E-11	-0.24758287	8.98E-05	0.85447393	4.60E-71

-0.424482786	3.89E-12	-0.244065401	0.000113752	0.914077724	3.16E-97
-0.152078195	0.01721428	-0.068034886	0.288827223	0.613570629	9.84E-27
-0.365672285	3.63E-09	-0.177855073	0.005239484	0.773416491	5.13E-50
-0.391615933	2.10E-10	-0.24305155	0.000121696	0.619460443	2.35E-27
-0.54475045	2.47E-20	-0.317800391	3.75E-07	0.675486927	5.31E-34
0.36945316	2.44E-09	0.174363939	0.006214151	-0.533605802	1.97E-19
-0.211950332	0.000842039	-0.008328267	0.89680885	0.601897649	1.54E-25
-0.33614251	6.96E-08	-0.368599543	2.67E-09	0.785986999	1.16E-52
-0.205729547	0.001201939	-0.095381183	0.136563035	0.493786368	1.83E-16
-0.510278218	1.20E-17	-0.331134993	1.11E-07	0.862264161	9.48E-74
-0.372654849	1.73E-09	-0.243826083	0.000115582	0.687900148	1.12E-35
-0.304439044	1.19E-06	-0.237507515	0.000175147	0.643854869	4.44E-30
-0.329630998	1.28E-07	-0.297228362	2.18E-06	0.618647157	2.87E-27
-0.06210617	0.333005038	0.046881214	0.465111442	0.405568734	4.07E-11
-0.40946426	2.54E-11	-0.315613502	4.55E-07	0.564911842	4.68E-22
0.281548768	7.63E-06	0.424712288	3.77E-12	-0.541467943	4.59E-20
-0.292134298	3.30E-06	-0.071459364	0.265184452	0.823383055	1.01E-61
-0.00624112	0.922575235	-0.003219328	0.960016498	0.58515681	6.60E-24
-0.332141649	1.01E-07	-0.212592856	0.000811182	0.485493185	6.81E-16
-0.477709633	2.27E-15	-0.265373917	2.57E-05	0.910271159	4.82E-95
-0.643714817	4.61E-30	-0.488459069	4.27E-16	0.840249473	1.53E-66
-0.257470143	4.53E-05	-0.226950729	0.000342263	0.675789041	4.84E-34
-0.349975013	1.81E-08	-0.19722737	0.001923262	0.883798958	4.00E-82
-0.567399696	2.82E-22	-0.404469443	4.64E-11	0.806636594	2.02E-57
-0.422926372	4.74E-12	-0.197971923	0.001847072	0.947810718	1.25E-122
-0.519018894	2.67E-18	-0.296893388	2.24E-06	0.5060449	2.45E-17
-0.233290661	0.000229726	-0.087363234	0.172861955	0.508872948	1.53E-17
-0.458385258	3.93E-14	-0.323085704	2.33E-07	0.747463554	4.66E-45
-0.236753047	0.000183924	-0.111369734	0.081909393	0.718996829	2.85E-40
-0.246982767	9.35E-05	-0.050752862	0.429028534	0.669548887	3.15E-33
-0.12948484	0.042874984	0.329740339	1.27E-07	0.579138939	2.42E-23
-0.512095664	8.83E-18	-0.350113846	1.79E-08	0.595072387	7.33E-25
-0.067252585	0.294420481	0.042392146	0.508967902	0.459585447	3.31E-14
-0.457420098	4.52E-14	-0.137559512	0.031367591	0.713052144	2.40E-39
-0.249898493	7.67E-05	-0.005878373	0.927062471	0.853891147	7.20E-71
-0.410317857	2.29E-11	-0.241742495	0.000132724	0.848072533	5.69E-69
-0.397632617	1.04E-10	-0.079875509	0.212820509	0.898954958	4.39E-89
-0.506774264	2.17E-17	-0.276755428	1.10E-05	0.933235375	5.06E-110
-0.298477554	1.97E-06	-0.221007452	0.00049248	0.665092637	1.17E-32
-0.370847325	2.10E-09	-0.320497316	2.95E-07	0.664971321	1.21E-32

-0.271144636	1.68E-05	-0.068136723	0.288104388	0.823825448	7.65E-62
0.633354421	7.07E-29	0.438748388	5.99E-13	-0.680799305	1.04E-34
0.384102657	4.92E-10	0.303236917	1.32E-06	-0.586982646	4.43E-24
0.530021377	3.79E-19	0.436306087	8.30E-13	-0.15856178	0.012957605
0.772771674	6.93E-50	0.50501384	2.91E-17	-0.432545978	1.36E-12
0.769831055	2.71E-49	0.561695019	8.98E-22	-0.553627196	4.45E-21
0.562050197	8.36E-22	0.274632728	1.30E-05	-0.228532745	0.000310169
0.758400429	4.52E-47	0.413470902	1.55E-11	-0.204219181	0.00130841
0.720968853	1.39E-40	0.560236501	1.20E-21	-0.315892301	4.44E-07
0.671316669	1.86E-33	0.327773585	1.52E-07	-0.310556985	7.08E-07
0.675860021	4.74E-34	0.591488921	1.64E-24	-0.548309762	1.25E-20
0.739414308	1.22E-43	0.446084541	2.21E-13	-0.507486444	1.93E-17
0.809759705	3.43E-58	0.602486239	1.35E-25	-0.431675799	1.53E-12
0.744011594	1.92E-44	0.42576454	3.30E-12	-0.418244931	8.57E-12
0.72405543	4.45E-41	0.353283987	1.30E-08	-0.034047275	0.595867518
0.840513614	1.27E-66	0.512070897	8.87E-18	-0.516801954	3.93E-18
0.689969514	5.77E-36	0.450003855	1.28E-13	-0.383909138	5.03E-10
0.872404355	1.67E-77	0.566892467	3.13E-22	-0.451818761	9.97E-14
0.80781326	1.04E-57	0.425662142	3.34E-12	-0.396318134	1.22E-10
0.7447229	1.44E-44	0.443249267	3.26E-13	-0.428948026	2.18E-12
0.645356342	2.96E-30	0.51989502	2.30E-18	-0.315652852	4.53E-07
0.807671134	1.13E-57	0.440267653	4.88E-13	-0.259000171	4.07E-05
0.745707319	9.60E-45	0.384092808	4.92E-10	-0.401286108	6.79E-11
0.692505473	2.54E-36	0.280576636	8.23E-06	-0.330064888	1.23E-07
0.687782095	1.16E-35	0.433529906	1.20E-12	-0.261622404	3.38E-05
0.742962534	2.93E-44	0.465050353	1.50E-14	-0.187331577	0.003247248
0.650431156	7.42E-31	0.593802247	9.76E-25	-0.445903287	2.27E-13
0.740494248	7.91E-44	0.338876102	5.36E-08	-0.385468861	4.22E-10
0.723075313	6.39E-41	0.449527561	1.37E-13	-0.334289792	8.29E-08
0.563413473	6.35E-22	0.282965702	6.84E-06	-0.139067691	0.029539779
0.861114706	2.42E-73	0.548362193	1.24E-20	-0.47116947	6.07E-15
0.566743007	3.22E-22	0.140694689	0.027670816	-0.244373417	0.000111437
0.477715438	2.26E-15	0.214645159	0.000719466	-0.195272145	0.002137192
0.594211556	8.90E-25	0.340516514	4.58E-08	-0.211161516	0.000881402
0.769231598	3.57E-49	0.340626308	4.53E-08	-0.29491237	2.64E-06
0.70905355	9.76E-39	0.517856786	3.27E-18	-0.256012148	5.02E-05
0.652678284	3.99E-31	0.313175379	5.63E-07	-0.364187665	4.25E-09
0.805047272	4.92E-57	0.445773236	2.31E-13	-0.532494069	2.42E-19
0.585304133	6.39E-24	0.427234567	2.73E-12	-0.312566487	5.94E-07
0.740318042	8.49E-44	0.560791075	1.08E-21	-0.51970575	2.37E-18

0.484009634	8.58E-16	0.205925393	0.001188732	-0.333331054	9.07E-08
0.666188136	8.47E-33	0.522136502	1.55E-18	-0.546552224	1.75E-20
0.821309261	3.63E-61	0.428831837	2.22E-12	-0.254801348	5.47E-05
0.646051237	2.46E-30	0.303711655	1.27E-06	-0.52969283	4.02E-19
0.582855958	1.09E-23	0.173244491	0.006559385	-0.292358895	3.24E-06
0.801791929	2.96E-56	0.354867117	1.11E-08	-0.159454748	0.012450636
0.840945991	9.40E-67	0.52498793	9.34E-19	-0.442372473	3.67E-13
0.734325221	9.00E-43	0.518928348	2.72E-18	-0.226627801	0.000349183
0.739530586	1.16E-43	0.413750669	1.50E-11	-0.301234127	1.56E-06
0.730061825	4.64E-42	0.449374808	1.40E-13	-0.280618648	8.20E-06
0.792554788	4.07E-54	0.357035165	8.90E-09	-0.472017561	5.35E-15
0.77709912	8.97E-51	0.398529347	9.40E-11	-0.526036566	7.75E-19
0.755398536	1.65E-46	0.27082418	1.72E-05	-0.345592075	2.80E-08
0.648138866	1.39E-30	0.38615433	3.91E-10	-0.191335576	0.002634984
0.830226807	1.29E-63	0.543838019	2.93E-20	-0.453390236	8.00E-14
0.678513847	2.11E-34	0.357131812	8.81E-09	-0.19585064	0.002071741
0.777338306	8.00E-51	0.204733774	0.0012712	-0.334110957	8.43E-08
0.703971169	5.61E-38	0.391683816	2.08E-10	-0.480933868	1.38E-15
0.847079796	1.18E-68	0.537872126	8.98E-20	-0.407925071	3.06E-11
0.791647349	6.51E-54	0.327242402	1.60E-07	-0.309189907	7.96E-07
0.542559176	3.73E-20	0.223172168	0.000431825	-0.112061033	0.080018225
0.756115464	1.22E-46	0.49906557	7.78E-17	-0.284604065	6.01E-06
0.79223682	4.80E-54	0.530001023	3.80E-19	-0.422307609	5.13E-12
0.766768609	1.10E-48	0.276013721	1.17E-05	-0.358291225	7.82E-09
0.611498958	1.62E-26	0.305479508	1.09E-06	-0.298674113	1.93E-06
0.775913825	1.58E-50	0.732633367	1.73E-42	-0.239973382	0.000149118
0.757779726	5.92E-47	0.597467405	4.26E-25	-0.255102505	5.35E-05
0.79149878	7.03E-54	0.471487294	5.79E-15	-0.306991742	9.61E-07
0.710536115	5.82E-39	0.375030542	1.34E-09	-0.446625037	2.05E-13
0.678228287	2.30E-34	0.295313238	2.55E-06	-0.395763688	1.30E-10
0.69348318	1.85E-36	0.358323187	7.80E-09	-0.422020488	5.32E-12
0.711500491	4.15E-39	0.549383921	1.02E-20	-0.334789592	7.91E-08
0.67084397	2.14E-33	0.371527051	1.95E-09	-0.44075813	4.57E-13
0.843530782	1.52E-67	0.541037147	4.97E-20	-0.524371951	1.04E-18
0.67077293	2.19E-33	0.377955099	9.72E-10	-0.176622021	0.005566826
0.796454446	5.25E-55	0.57120976	1.28E-22	-0.448832397	1.51E-13
0.707908792	1.45E-38	0.468317212	9.28E-15	-0.337937305	5.86E-08
0.624696174	6.41E-28	0.179884653	0.004738167	-0.378838781	8.82E-10
0.747260634	5.07E-45	0.549963903	9.08E-21	-0.263199986	3.01E-05
0.547525428	1.45E-20	0.258592501	4.19E-05	-0.528205537	5.25E-19

0.730924975	3.34E-42	0.407340945	3.29E-11	-0.408238829	2.95E-11
0.690797604	4.42E-36	0.389332326	2.72E-10	-0.215420276	0.000687392
0.730485281	3.95E-42	0.508315895	1.68E-17	-0.359091692	7.21E-09
0.80694594	1.70E-57	0.527804844	5.65E-19	-0.393964568	1.60E-10
0.766185154	1.43E-48	0.674211401	7.81E-34	-0.279182711	9.16E-06
0.756299803	1.12E-46	0.268118537	2.11E-05	-0.326703399	1.68E-07
0.637465191	2.42E-29	0.4408442	4.51E-13	-0.324779134	2.00E-07
0.767644531	7.39E-49	0.381619878	6.49E-10	-0.468084608	9.60E-15
0.802352309	2.18E-56	0.469838	7.41E-15	-0.495833848	1.32E-16
0.812463932	7.20E-59	0.613853017	9.20E-27	-0.390676254	2.34E-10
0.603395235	1.09E-25	0.203340818	0.001374239	-0.415917178	1.15E-11
0.615390934	6.34E-27	0.30224335	1.44E-06	-0.325319031	1.91E-07
0.496730465	1.14E-16	0.165009803	0.009671346	-0.300975593	1.60E-06
0.524977528	9.36E-19	0.282228178	7.24E-06	-0.153115958	0.016460235
0.82278696	1.46E-61	0.414274368	1.41E-11	-0.439095558	5.71E-13
0.739896459	1.00E-43	0.383970952	4.99E-10	-0.352776935	1.37E-08
0.745658487	9.79E-45	0.644897451	3.35E-30	-0.407838475	3.09E-11
0.698650771	3.37E-37	0.503350294	3.84E-17	-0.343181675	3.54E-08
0.816559618	6.45E-60	0.699992001	2.15E-37	-0.424229764	4.01E-12
0.683287351	4.80E-35	0.334866965	7.85E-08	-0.348316407	2.14E-08
0.707785945	1.51E-38	0.317298439	3.92E-07	-0.414688663	1.34E-11
0.520455034	2.08E-18	0.367845811	2.89E-09	-0.116143369	0.069557626
0.731246767	2.95E-42	0.452850773	8.63E-14	-0.353680019	1.25E-08
0.660008905	5.05E-32	0.314375046	5.07E-07	-0.355892454	9.99E-09
0.726830029	1.58E-41	0.400923854	7.08E-11	-0.322220383	2.52E-07
0.816496357	6.69E-60	0.409809239	2.43E-11	-0.388274028	3.07E-10
0.628932077	2.20E-28	0.328616044	1.41E-07	-0.449259511	1.42E-13
0.725553365	2.55E-41	0.47479973	3.52E-15	-0.082336466	0.199015837
0.767771485	6.97E-49	0.393893737	1.61E-10	-0.274607946	1.30E-05
0.660830555	3.99E-32	0.427220882	2.73E-12	-0.314920245	4.84E-07
0.804685078	6.01E-57	0.651646672	5.30E-31	-0.468715628	8.75E-15
0.504996813	2.92E-17	0.42201826	5.32E-12	-0.190447536	0.002760927
0.564086289	5.54E-22	0.330201615	1.22E-07	-0.289753134	4.00E-06
0.877891085	1.14E-79	0.475060783	3.39E-15	-0.440374899	4.81E-13
0.740312696	8.51E-44	0.407178147	3.35E-11	-0.306849363	9.73E-07
0.818261276	2.32E-60	0.282391872	7.15E-06	-0.372501298	1.76E-09
0.825333146	2.97E-62	0.662975584	2.16E-32	-0.508556325	1.61E-17
0.810379753	2.40E-58	0.442383822	3.66E-13	-0.341560267	4.14E-08
0.777432395	7.64E-51	0.490789342	2.95E-16	-0.370513055	2.18E-09
0.63238811	9.08E-29	0.26823522	2.09E-05	-0.363784658	4.43E-09

0.841376942	6.96E-67	0.559969621	1.27E-21	-0.340606402	4.54E-08
0.785891341	1.22E-52	0.533599399	1.97E-19	-0.211701754	0.000854265
0.670162438	2.62E-33	0.400991052	7.03E-11	-0.294241502	2.78E-06
0.788730981	2.90E-53	0.418587773	8.21E-12	-0.383791886	5.09E-10
0.552738197	5.30E-21	0.245267884	0.000104961	-0.305796455	1.06E-06
0.598537532	3.34E-25	0.431632457	1.54E-12	0.083631688	0.192017118
0.619141355	2.54E-27	0.32532781	1.90E-07	-0.164736018	0.00979422
0.767303628	8.62E-49	0.542855424	3.53E-20	-0.226095593	0.000360871
0.733826843	1.09E-42	0.246656362	9.56E-05	-0.195301001	0.002133883
0.652151892	4.61E-31	0.341551026	4.14E-08	-0.362567182	5.03E-09
0.761796052	1.02E-47	0.590082433	2.24E-24	-0.436040032	8.60E-13
0.659806084	5.35E-32	0.541782125	4.32E-20	-0.276811162	1.10E-05
0.547903191	1.35E-20	0.343938066	3.29E-08	-0.634780564	4.89E-29
0.700873066	1.60E-37	0.394059013	1.58E-10	-0.391686231	2.08E-10
0.635183247	4.40E-29	0.290039708	3.91E-06	-0.373212702	1.63E-09
0.838204792	6.28E-66	0.438495349	6.19E-13	-0.258383498	4.25E-05
0.567900501	2.54E-22	0.321380467	2.72E-07	-0.245540525	0.000103058
0.455283239	6.12E-14	0.301373099	1.55E-06	-0.077333999	0.227784649
0.666290013	8.22E-33	0.379763993	7.96E-10	-0.35218376	1.45E-08
0.626640357	3.93E-28	0.502616544	4.34E-17	-0.296002536	2.41E-06
0.46176675	2.42E-14	0.27207887	1.57E-05	0.057754534	0.368051112
0.795574639	8.37E-55	0.591772076	1.54E-24	-0.383986035	4.98E-10
0.697199607	5.45E-37	0.562631455	7.43E-22	-0.519315726	2.54E-18
0.586892581	4.52E-24	0.173545627	0.006464869	-0.419045492	7.75E-12
0.565117371	4.49E-22	0.32723017	1.60E-07	-0.303998839	1.24E-06
0.605198212	7.17E-26	0.494148144	1.73E-16	-0.2555876	5.18E-05
0.695555859	9.38E-37	0.534273931	1.74E-19	-0.411749675	1.92E-11
0.78425458	2.75E-52	0.29941632	1.82E-06	-0.335097901	7.68E-08
0.763040461	5.87E-48	0.429124302	2.13E-12	-0.32837246	1.44E-07
0.763646974	4.48E-48	0.321223758	2.76E-07	-0.138047227	0.030766241
0.605057408	7.41E-26	0.370480265	2.18E-09	-0.027781699	0.665223979
0.631659613	1.09E-28	0.511050678	1.06E-17	-0.247497233	9.03E-05
0.657790329	9.50E-32	0.489785178	3.47E-16	-0.447827636	1.74E-13
0.786860973	7.47E-53	0.334210962	8.35E-08	-0.351473726	1.56E-08
0.782715404	5.88E-52	0.604621542	8.20E-26	-0.507203568	2.02E-17
0.649609589	9.30E-31	0.302584358	1.40E-06	-0.360419603	6.28E-09
0.737182386	2.94E-43	0.372524159	1.75E-09	-0.375647406	1.25E-09
0.812360401	7.65E-59	0.324629403	2.03E-07	-0.420582288	6.38E-12
0.748599837	2.91E-45	0.517266136	3.63E-18	-0.56963035	1.78E-22
0.694636445	1.27E-36	0.286638056	5.12E-06	-0.494479324	1.64E-16

0.656184768	1.49E-31	0.412801273	1.69E-11	-0.449053852	1.47E-13
0.505331486	2.76E-17	0.437752395	6.84E-13	-0.228549545	0.000309844
0.332788086	9.55E-08	0.725854307	2.28E-41	-0.036622205	0.568346703
0.347488162	2.32E-08	0.437569468	7.01E-13	-0.386517625	3.75E-10
0.358667404	7.53E-09	0.60403368	9.41E-26	-0.037193629	0.562322244
0.487417906	5.04E-16	0.656111126	1.53E-31	-0.251407247	6.92E-05
0.596615923	5.17E-25	0.871815315	2.82E-77	-0.344425776	3.13E-08
0.377096673	1.07E-09	0.807174361	1.49E-57	-0.168223221	0.008328021
0.441356451	4.21E-13	0.870367995	1.00E-76	-0.272118279	1.57E-05
0.526937705	6.60E-19	0.770383226	2.10E-49	-0.386944642	3.57E-10
0.655409296	1.86E-31	0.747765388	4.12E-45	-0.419781524	7.06E-12
0.408667484	2.80E-11	0.648570843	1.24E-30	-0.11071433	0.083735478
0.736762044	3.47E-43	0.775075978	2.35E-50	-0.362740493	4.94E-09
0.387039213	3.53E-10	0.474618917	3.62E-15	-0.168999519	0.008029496
0.354043042	1.21E-08	0.801784562	2.98E-56	-0.06479919	0.312426894
-0.37595724	1.21E-09	-0.42079371	6.22E-12	-0.181878032	0.004288222
0.615244241	6.57E-27	0.760264382	2.00E-47	0.003456689	0.957071244
0.254031723	5.77E-05	0.512506992	8.23E-18	-0.155087041	0.015107112
0.23332269	0.000229258	0.814367169	2.36E-59	0.052954263	0.409254864
0.377600543	1.01E-09	0.513787533	6.61E-18	-0.293617113	2.93E-06
0.328918077	1.37E-07	0.716347015	7.41E-40	-0.143345663	0.024842668
0.24525361	0.000105061	0.750812818	1.16E-45	-0.110972062	0.083013514
0.55634869	2.61E-21	0.766995471	9.92E-49	-0.339718056	4.94E-08
0.448603536	1.56E-13	0.808601841	6.65E-58	-0.14791779	0.020547639
0.488925952	3.97E-16	0.786058272	1.12E-52	-0.191600328	0.00259846
0.133109549	0.037332151	0.452009921	9.71E-14	-0.190778556	0.002713356
0.545698471	2.06E-20	0.800263914	6.81E-56	-0.026444802	0.68042604
0.43248038	1.38E-12	0.820279276	6.83E-61	0.008196656	0.898430721
0.59355195	1.03E-24	0.832368461	3.18E-64	-0.02433564	0.704672164
0.489486571	3.63E-16	0.691547651	3.47E-36	-0.01277656	0.84228607
0.270624149	1.75E-05	0.70186332	1.15E-37	-0.398283099	9.67E-11
0.40719451	3.34E-11	0.862429386	8.28E-74	-0.391556806	2.11E-10
0.415584549	1.19E-11	0.66318143	2.03E-32	-0.101002265	0.11482186
0.351073699	1.62E-08	0.623703613	8.21E-28	-0.443215729	3.27E-13
0.611806894	1.50E-26	0.684511272	3.27E-35	-0.339019548	5.29E-08
0.412290807	1.80E-11	0.627540845	3.13E-28	-0.144685691	0.023510295
0.361982291	5.35E-09	0.838926295	3.82E-66	-0.054364567	0.396875267
0.598281629	3.54E-25	0.881717638	3.03E-81	-0.366767509	3.24E-09
0.27709368	1.07E-05	0.715596493	9.70E-40	-0.226870289	0.000343975
0.623132661	9.47E-28	0.81057914	2.14E-58	-0.403170376	5.42E-11

0.441193516	4.31E-13	0.578846472	2.57E-23	-0.263106036	3.03E-05
0.458542048	3.85E-14	0.668051671	4.90E-33	-0.121389468	0.057781628
0.466610489	1.19E-14	0.61997297	2.07E-27	-0.044846717	0.484724582
0.429556444	2.02E-12	0.80547859	3.87E-57	-0.137828548	0.031034643
0.463306565	1.93E-14	0.833535727	1.47E-64	-0.003100368	0.961492793
0.465986569	1.31E-14	0.823170339	1.15E-61	-0.290614544	3.73E-06
0.529027353	4.53E-19	0.84210551	4.17E-67	-0.377019108	1.08E-09
0.493398919	1.95E-16	0.698217359	3.89E-37	-0.408268047	2.94E-11
0.425638739	3.35E-12	0.905094391	3.18E-92	-0.221108218	0.00048949
0.690576757	4.74E-36	0.797056313	3.82E-55	-0.419243618	7.56E-12
0.465006039	1.51E-14	0.819999372	8.10E-61	-0.211986623	0.000840268
0.429367185	2.07E-12	0.676223817	4.24E-34	-0.361231474	5.78E-09
0.255872865	5.07E-05	0.467981282	9.75E-15	-0.310171436	7.31E-07
0.440314461	4.85E-13	0.767862134	6.69E-49	-0.137404937	0.03156026
0.326146415	1.77E-07	0.633201966	7.36E-29	-0.420524304	6.43E-12
0.528765231	4.75E-19	0.877424058	1.75E-79	-0.166009466	0.009234219
0.497234143	1.05E-16	0.53409012	1.80E-19	-0.265522837	2.55E-05
0.627949041	2.82E-28	0.803775498	9.96E-57	-0.222327507	0.000454617
0.249087521	8.11E-05	0.843203168	1.92E-67	0.015872393	0.804763293
0.33322735	9.16E-08	0.606824314	4.90E-26	-0.126315208	0.04827359
0.375999575	1.20E-09	0.691588247	3.42E-36	0.030149701	0.638633085
0.38504547	4.42E-10	0.858977884	1.35E-72	-0.185404583	0.003585562
0.22052781	0.000506947	0.650348443	7.59E-31	-0.089660016	0.16179996
0.264127016	2.82E-05	0.755532707	1.56E-46	-0.184514489	0.003752334
0.599253825	2.84E-25	0.59253642	1.30E-24	-0.19130854	0.00263874
0.761898483	9.75E-48	0.749175085	2.29E-45	-0.225999477	0.000363021
0.559777147	1.32E-21	0.824503045	5.01E-62	-0.374318964	1.44E-09
0.450751392	1.16E-13	0.773910845	4.06E-50	-0.187591622	0.003203878
0.697147266	5.55E-37	0.755829044	1.38E-46	-0.247051477	9.31E-05
0.391042284	2.24E-10	0.834121493	9.95E-65	-0.279305232	9.08E-06
0.46760338	1.03E-14	0.817739023	3.18E-60	-0.415202899	1.25E-11
0.588252265	3.35E-24	0.822913941	1.35E-61	-0.263329617	2.99E-05
0.557419754	2.11E-21	0.76834437	5.37E-49	-0.173603075	0.006446977
0.509738669	1.32E-17	0.875252292	1.29E-78	-0.299974491	1.74E-06
0.249075346	8.11E-05	0.795097526	1.08E-54	-0.013579428	0.832517093
0.615192973	6.65E-27	0.762267554	8.28E-48	-0.180348891	0.004629744
0.379558573	8.15E-10	0.786376672	9.53E-53	-0.345857581	2.72E-08
0.372232447	1.81E-09	0.870562196	8.48E-77	-0.151815592	0.017409799
0.232285977	0.000244888	0.710635352	5.62E-39	-0.137790973	0.031080962
0.428597234	2.29E-12	0.686519419	1.73E-35	-0.124296244	0.05199971

0.297437112	2.14E-06	0.663786011	1.70E-32	-0.193469692	0.002353386
0.456285829	5.31E-14	0.721663218	1.08E-40	-0.409037014	2.67E-11
0.475663714	3.09E-15	0.860821623	3.06E-73	-0.192437621	0.002485958
0.495011764	1.50E-16	0.602815929	1.25E-25	-0.036018474	0.574744907
0.105249731	0.100266737	0.654138728	2.65E-31	-0.254835531	5.45E-05
0.148603952	0.019962087	0.744785051	1.40E-44	-0.036607197	0.568505345
0.405074356	4.32E-11	0.746041134	8.37E-45	-0.166752804	0.008920628
0.305381843	1.10E-06	0.678059697	2.42E-34	-0.173563769	0.006459214
0.477000191	2.52E-15	0.843751885	1.30E-67	-0.311041724	6.78E-07
0.478827786	1.91E-15	0.850675482	8.24E-70	-0.158666689	0.012897119
0.349981923	1.81E-08	0.607078148	4.61E-26	-0.172518063	0.006792485
0.327014744	1.63E-07	0.613607458	9.76E-27	-0.184130606	0.003826399
0.234831555	0.000208165	0.730680048	3.67E-42	-0.025683569	0.689140658
0.607302977	4.38E-26	0.788941159	2.61E-53	-0.214957923	0.000706362
0.616576765	4.76E-27	0.80174596	3.04E-56	-0.455871681	5.63E-14
0.404733032	4.50E-11	0.699084189	2.91E-37	-0.27210469	1.57E-05
0.349433268	1.91E-08	0.627709411	3.00E-28	-0.110195108	0.08520527
0.63305582	7.64E-29	0.819811219	9.08E-61	-0.257798634	4.43E-05
0.383224824	5.42E-10	0.774265989	3.44E-50	-0.132408773	0.038353792
0.457591211	4.41E-14	0.585294213	6.41E-24	-0.274081357	1.35E-05
0.130835208	0.040734165	0.107044326	0.094574273	-0.28843368	4.44E-06
0.269850834	1.85E-05	0.208166571	0.001046793	-0.188328725	0.003083779
0.310219374	7.28E-07	0.18388246	0.003874974	-0.436090954	8.54E-13
0.149138175	0.019516271	0.115604731	0.070870367	-0.050175975	0.434300151
0.3143497	5.08E-07	0.296986092	2.22E-06	-0.074298321	0.246616275
0.333846531	8.64E-08	0.099928309	0.118751931	-0.169644313	0.00778882
0.174382291	0.00620863	0.22630726	0.00035618	-0.19560628	0.002099162
0.307628005	9.10E-07	0.168816005	0.008099194	-0.231890795	0.000251103
-0.257780452	4.44E-05	-0.051115652	0.425732365	0.250545928	7.34E-05
0.207947664	0.001059937	0.108989229	0.088698854	-0.163626788	0.010306299
0.329874177	1.25E-07	0.347644236	2.28E-08	-0.208080591	0.001051938
0.35747105	8.51E-09	0.174453325	0.006187299	-0.396795265	1.15E-10
0.188935523	0.002987989	0.14317651	0.025015389	-0.022881504	0.721565557
0.344457381	3.12E-08	0.1808106	0.004524132	-0.167982194	0.008422695
0.360058983	6.52E-09	0.200902881	0.001573176	-0.315914244	4.43E-07
0.448057703	1.68E-13	0.384802909	4.55E-10	-0.069958177	0.275380428
0.403039843	5.51E-11	0.31656568	4.18E-07	-0.350234078	1.77E-08
0.268870047	1.99E-05	0.231990703	0.000249518	-0.198249064	0.001819424
0.029002615	0.651459043	-0.013420264	0.834451748	0.168845364	0.008088007
0.221366571	0.0004819	0.128480034	0.044528624	-0.191367304	0.002630583

0.04507419	0.482509797	-0.016453724	0.797764146	-0.250553607	7.33E-05
0.167602975	0.008573586	0.029333268	0.647751159	-0.155001616	0.015163672
0.418900249	7.89E-12	0.409635819	2.49E-11	-0.283684362	6.46E-06
0.270668156	1.74E-05	0.237039686	0.000180542	-0.181127406	0.004452925
0.267357345	2.23E-05	0.18224354	0.004210027	-0.166423714	0.009058276
0.262372058	3.20E-05	0.234403859	0.000213952	-0.014584022	0.820329876
-0.01800735	0.779139262	0.022278903	0.728606572	0.045637577	0.477047994
0.317761714	3.76E-07	0.223433833	0.000424983	-0.196216771	0.002031267
0.182203635	0.004218501	0.1576352	0.013502756	-0.061113993	0.34080089
0.427216037	2.73E-12	0.2842192	6.20E-06	-0.30595411	1.05E-06
0.264194676	2.80E-05	0.301075303	1.58E-06	0.086510253	0.177110176
0.452484072	9.08E-14	0.338025419	5.82E-08	-0.065686105	0.305836001
0.249422357	7.92E-05	0.263080335	3.04E-05	-0.209675546	0.000960193
-0.160074545	0.012109099	-0.024517575	0.702568486	0.224168299	0.00040631
0.070319864	0.272899888	0.080750613	0.207834994	-0.001843433	0.977098475
0.43691636	7.65E-13	0.227664416	0.00032742	-0.160077142	0.012107685
0.091780632	0.152065985	0.088938431	0.165216571	-0.151792624	0.017426991
0.242233264	0.000128484	0.169383293	0.007885463	-0.150754864	0.018219377
-0.335998193	7.05E-08	-0.284896625	5.88E-06	-0.037961062	0.554279833
0.331012017	1.13E-07	0.249960585	7.64E-05	-0.258816992	4.12E-05
0.433165577	1.26E-12	0.171108501	0.007265997	-0.30470377	1.17E-06
0.230483889	0.000274443	0.197956889	0.001848583	-0.019004076	0.767255789
0.339535363	5.03E-08	0.272024682	1.58E-05	-0.035286865	0.582543407
0.423949588	4.16E-12	0.21658336	0.000641735	0.004463571	0.944584476
0.173885554	0.006359643	0.088297894	0.168294392	-0.058651598	0.360645913
-0.276293284	1.14E-05	-0.144015113	0.024169133	0.265421422	2.57E-05
0.27937528	9.03E-06	0.142730551	0.025475714	-0.166139935	0.009178481
0.230277254	0.000278037	0.172899508	0.006669178	-0.119319401	0.062215046
0.271331054	1.66E-05	0.143935929	0.024247971	-0.229764865	0.000287137
0.196221804	0.002030715	0.10388562	0.10477266	-0.05230866	0.414997284
-0.208593942	0.001021565	-0.093734302	0.143496565	0.294317207	2.77E-06
0.347737609	2.26E-08	0.284143498	6.23E-06	-0.162767001	0.010719371
-0.335039546	7.72E-08	-0.272734913	1.49E-05	-0.104246808	0.103564318
-0.317650024	3.80E-07	-0.205063882	0.001247843	0.364568121	4.08E-09
-0.1600873	0.012102158	-0.270486162	1.77E-05	-0.201427315	0.001528281
0.233445765	0.000227465	0.166122286	0.009186003	-0.162323919	0.010937903
-0.377402948	1.03E-09	-0.276513448	1.12E-05	0.16876706	0.008117873
0.300586481	1.65E-06	0.451887297	9.88E-14	0.017386529	0.786567205
0.263357989	2.98E-05	0.244359726	0.000111539	-0.112616747	0.078523674
0.426374584	3.05E-12	0.316103645	4.36E-07	-0.221313879	0.000483439

0.438670558	6.05E-13	0.283798331	6.41E-06	-0.224510801	0.000397865
0.124602995	0.051418635	0.217536485	0.000606427	-0.094694167	0.139423537
-0.038888569	0.544635166	-0.091757383	0.152170244	0.098209922	0.125257287
0.293461531	2.97E-06	0.187301812	0.003252246	-0.182373191	0.004182601
0.412829085	1.68E-11	0.262842348	3.09E-05	-0.095283984	0.136964981
-0.031398491	0.624791176	0.002783196	0.965429551	0.255562168	5.18E-05
0.296876986	2.24E-06	-0.006942772	0.913902784	-0.337451913	6.14E-08
-0.237568305	0.000174458	-0.213934818	0.000750068	-0.007583483	0.905992427
0.315720202	4.51E-07	0.16884649	0.008087578	0.024177908	0.706497803
-0.136291453	0.032978188	-0.074402818	0.245950536	0.09721979	0.129129091
-0.223587975	0.000420999	-0.155821502	0.014628398	0.411793489	1.91E-11
-0.198825015	0.00176317	-0.283654543	6.48E-06	0.333209254	9.18E-08
-0.21223728	0.000828127	-0.122137488	0.056245218	0.556806656	2.38E-21
-0.285245204	5.72E-06	-0.30237202	1.42E-06	0.376122336	1.19E-09
-0.194641531	0.002210682	-0.229576854	0.000290546	0.421000273	6.06E-12
-0.210137216	0.000935045	-0.237755243	0.000172352	0.347594174	2.30E-08
-0.244754518	0.000108633	-0.259201178	4.01E-05	0.403257446	5.37E-11
-0.101334478	0.11362697	-0.040210084	0.531038145	0.335557771	7.35E-08
-0.101768952	0.112078983	-0.012527862	0.845317097	0.380299034	7.51E-10
-0.379729075	8.00E-10	-0.373449497	1.59E-09	0.513505905	6.94E-18
-0.30499102	1.14E-06	-0.211707646	0.000853974	0.463576235	1.86E-14
-0.250353345	7.44E-05	-0.421897837	5.40E-12	0.265284272	2.59E-05
-0.079793898	0.213289789	-0.142582745	0.02562988	0.378879928	8.78E-10
-0.163124502	0.010545871	-0.142535879	0.02567893	0.350915776	1.65E-08
-0.094717034	0.139327597	-0.096782817	0.130866938	-0.187068621	0.003291644
-0.202793658	0.001416766	-0.275272138	1.23E-05	0.32970473	1.27E-07
-0.111281372	0.082153699	-0.16291684	0.010646348	0.308620503	8.36E-07
-0.23395307	0.000220214	-0.057789137	0.36776373	0.412651829	1.72E-11
-0.221198668	0.00048682	-0.210629477	0.000908901	0.286116664	5.34E-06
-0.17825874	0.005136138	-0.088055943	0.169468048	0.25705532	4.67E-05
-0.335694883	7.26E-08	-0.228211732	0.000316446	0.283067064	6.78E-06
-0.191664006	0.002589744	-0.15289067	0.016621435	0.391299515	2.18E-10
0.384053105	4.94E-10	0.218404266	0.000575852	-0.020673508	0.747474567
-0.079508196	0.214938483	-0.133391687	0.036927381	0.365118125	3.85E-09
-0.088772457	0.166010016	-0.072165796	0.260476982	0.496361776	1.21E-16
-0.269357645	1.92E-05	-0.106905826	0.095004224	0.486928107	5.44E-16
0.097119332	0.129527029	0.044323775	0.48983689	0.248048262	8.70E-05
-0.123928983	0.052702576	-0.119309262	0.062237428	0.292373925	3.24E-06
-0.151190062	0.017883341	-0.084677267	0.186499894	0.431614283	1.54E-12
-0.374634833	1.40E-09	-0.370995145	2.07E-09	0.487217653	5.20E-16

-0.057974649	0.366225422	-0.106583543	0.096010719	0.341805907	4.04E-08
-0.251559948	6.84E-05	-0.250134049	7.55E-05	0.280778224	8.10E-06
-0.092277413	0.149851092	-0.137323233	0.031662507	0.288648059	4.37E-06
-0.180820017	0.004522	-0.254250114	5.68E-05	0.316614604	4.16E-07
0.284709779	5.96E-06	0.122273146	0.055970233	0.136699789	0.032452037
-0.274171156	1.34E-05	-0.281912386	7.42E-06	0.410396738	2.27E-11
-0.343695241	3.37E-08	-0.257332669	4.58E-05	0.452474839	9.09E-14
-0.109719594	0.086569456	-0.036434212	0.570335377	0.454632548	6.71E-14
-0.218416909	0.000575417	-0.283970929	6.32E-06	0.276843979	1.10E-05
-0.105690227	0.098844946	-0.12232039	0.05587473	0.272732644	1.49E-05
-0.186727306	0.003350089	-0.248437469	8.47E-05	0.220673886	0.0005025
-0.257373478	4.57E-05	-0.305353097	1.11E-06	0.429402689	2.06E-12
-0.158761828	0.01284248	-0.094790245	0.139020768	0.358742077	7.47E-09
-0.141217605	0.027092069	-0.089709517	0.161567536	0.182465261	0.004163222
-0.197961878	0.001848082	-0.316389073	4.25E-07	0.301888955	1.48E-06
0.103675587	0.105480416	0.045587307	0.477533971	0.233312004	0.000229414
-0.10864629	0.089713071	-0.12386163	0.05283233	0.304836321	1.15E-06
-0.054220747	0.398127335	-0.087589369	0.171748502	0.371279152	2.00E-09
-0.20408466	0.0013183	-0.289146281	4.20E-06	0.317269824	3.93E-07
-0.221386284	0.000481326	-0.145414107	0.02281224	0.382149955	6.11E-10
-0.158503512	0.012991308	-0.170131016	0.007611429	0.26641144	2.39E-05
-0.202193954	0.00146476	-0.288786255	4.32E-06	0.133431444	0.036870643
0.028421393	0.65799754	0.086750896	0.175903918	0.39722658	1.09E-10
-0.118055151	0.065057637	-0.278705371	9.50E-06	0.210178469	0.000932828
-0.256979625	4.69E-05	-0.349366509	1.93E-08	0.361604385	5.56E-09
-0.249750638	7.75E-05	-0.217232811	0.000617476	0.542941867	3.47E-20
-0.045416397	0.479188221	-0.059145316	0.356610228	0.276239431	1.15E-05
0.077574251	0.226338985	0.072635654	0.257377982	-0.127472926	0.046239384
-0.075452224	0.239334282	-0.111635738	0.081177473	0.042242878	0.510462294
-0.176701561	0.00554517	-0.153677877	0.016064101	0.542653269	3.67E-20
-0.460238898	3.02E-14	-0.40390802	4.97E-11	0.632540952	8.73E-29
-0.049765099	0.438077318	-0.17046359	0.007492294	0.309841302	7.53E-07
-0.120555301	0.059535702	-0.042279388	0.510096565	0.362880012	4.87E-09
0.076558183	0.232497573	0.003177839	0.960531362	0.383105691	5.50E-10
-0.14113035	0.027187911	-0.171598743	0.007098073	0.379950135	7.80E-10
-0.105656765	0.098952386	-0.09656968	0.131721101	-0.188004523	0.003136091
-0.423552339	4.38E-12	-0.485181032	7.15E-16	0.257232115	4.61E-05
-0.482669894	1.06E-15	-0.464262346	1.68E-14	0.11384088	0.075311062
-0.323839605	2.18E-07	-0.339537043	5.03E-08	0.03182801	0.620060204
-0.314165834	5.17E-07	-0.279862518	8.69E-06	0.260827803	3.57E-05

-0.543747501	2.98E-20	-0.480804545	1.41E-15	0.236005622	0.000193021
-0.49110261	2.81E-16	-0.49361011	1.88E-16	0.446990871	1.95E-13
0.520349489	2.12E-18	0.37154643	1.95E-09	-0.350156449	1.78E-08
-0.317428747	3.88E-07	-0.447163875	1.90E-13	0.269656913	1.88E-05
-0.515015713	5.35E-18	-0.471083319	6.15E-15	0.312342962	6.06E-07
0.400633582	7.33E-11	0.321808597	2.62E-07	-0.167631299	0.008562233
-0.300687106	1.64E-06	-0.309432262	7.80E-07	0.343540564	3.42E-08
-0.448270111	1.63E-13	-0.382140861	6.12E-10	0.293877046	2.87E-06
-0.599061419	2.96E-25	-0.539429507	6.72E-20	0.360300707	6.36E-09
-0.323152423	2.32E-07	-0.473407975	4.34E-15	0.093267987	0.145507743
-0.328181348	1.47E-07	-0.305163735	1.12E-06	0.151984836	0.017283569
-0.260260433	3.72E-05	-0.318602346	3.49E-07	0.503971182	3.47E-17
-0.34803028	2.20E-08	-0.370880001	2.09E-09	0.447678709	1.77E-13
0.41268004	1.71E-11	0.412072334	1.84E-11	-0.304115724	1.23E-06
-0.452383869	9.21E-14	-0.488915112	3.98E-16	0.27970022	8.80E-06
-0.622047301	1.24E-27	-0.587746934	3.75E-24	0.633616308	6.61E-29
0.309437876	7.79E-07	0.368664722	2.65E-09	-0.3058414	1.06E-06
0.276142307	1.16E-05	0.090330353	0.158673748	-0.426778216	2.89E-12
-0.387926101	3.20E-10	-0.326682098	1.68E-07	0.39976698	8.12E-11
0.284733613	5.95E-06	0.326280316	1.74E-07	-0.19104827	0.002675147
0.649403863	9.84E-31	0.48105515	1.36E-15	-0.150178057	0.018673214
0.561212115	9.89E-22	0.260414085	3.68E-05	-0.486209466	6.09E-16
-0.470762497	6.45E-15	-0.459704695	3.26E-14	0.152319797	0.017036093
-0.113815687	0.075376087	-0.315443547	4.62E-07	0.135440453	0.034098078
-0.483281437	9.61E-16	-0.461673066	2.45E-14	0.35529599	1.06E-08
0.377062092	1.07E-09	0.208827035	0.001008041	-0.12305614	0.054404741
-0.567099358	3.00E-22	-0.563870664	5.79E-22	0.32394288	2.16E-07
-0.437586839	6.99E-13	-0.294028813	2.83E-06	0.210796523	0.000900183
0.565806812	3.90E-22	0.419439879	7.37E-12	-0.030188164	0.638204841
-0.302827352	1.37E-06	-0.224666991	0.000394069	0.503760197	3.59E-17
-0.532806127	2.28E-19	-0.565659724	4.02E-22	0.309023399	8.08E-07
-0.389416108	2.70E-10	-0.376213154	1.18E-09	0.414316988	1.40E-11
0.155540051	0.014810251	0.204746888	0.001270264	0.036807148	0.566393515
-0.412854499	1.68E-11	-0.526043378	7.74E-19	0.362361539	5.14E-09
0.616903794	4.39E-27	0.430632783	1.75E-12	-0.034533187	0.590628098
-0.079944432	0.212424766	-0.401985896	6.24E-11	0.22364883	0.000419436
-0.252471344	6.43E-05	-0.33876279	5.42E-08	0.059481594	0.353877729
-0.656367145	1.42E-31	-0.631363447	1.18E-28	0.245437759	0.000103771
-0.1663925	0.009071428	-0.304224324	1.22E-06	0.101998521	0.111267749
-0.165165472	0.009602093	-0.275774703	1.19E-05	0.30290848	1.36E-06

0.374342984	1.44E-09	0.265714804	2.51E-05	-0.439711269	5.26E-13
0.35802028	8.04E-09	0.316250242	4.30E-07	-0.269515824	1.90E-05
0.371577422	1.94E-09	0.315562594	4.57E-07	-0.116364711	0.069023967
-0.353497469	1.27E-08	-0.421661708	5.57E-12	0.298090071	2.03E-06
0.07915419	0.216993971	0.11984736	0.061058579	-0.353449917	1.28E-08
0.323455296	2.26E-07	0.582699252	1.13E-23	-0.239404878	0.000154776
0.391774336	2.06E-10	0.63838371	1.90E-29	-0.126540165	0.047872578
-0.49878424	8.14E-17	-0.510982585	1.07E-17	0.315066633	4.77E-07
0.343566446	3.41E-08	0.234904299	0.000207196	0.075056115	0.241816843
0.452239852	9.40E-14	0.379996719	7.76E-10	-0.329870894	1.25E-07
-0.503213916	3.93E-17	-0.420549186	6.41E-12	0.396359718	1.21E-10
-0.469556583	7.72E-15	-0.573611079	7.78E-23	0.253309166	6.07E-05
-0.445855842	2.28E-13	-0.302870919	1.36E-06	0.599254359	2.83E-25
0.461927682	2.36E-14	0.467476983	1.05E-14	-0.248231884	8.59E-05
0.464451763	1.64E-14	0.40532905	4.19E-11	-0.204904862	0.001259045
0.373373718	1.60E-09	0.379372983	8.32E-10	-0.244653456	0.00010937
0.487425375	5.03E-16	0.328490534	1.42E-07	-0.221813514	0.000469027
-0.505576001	2.65E-17	-0.546711186	1.70E-20	0.417277271	9.67E-12
0.199028566	0.001743672	0.347579515	2.30E-08	-0.129181476	0.043368713
0.188408865	0.003070971	0.279372712	9.03E-06	-0.274550144	1.30E-05
0.320486195	2.95E-07	0.311680171	6.42E-07	-0.477458337	2.35E-15
-0.31473965	4.91E-07	-0.329491217	1.30E-07	0.434792779	1.01E-12
-0.580264209	1.90E-23	-0.510426811	1.17E-17	0.346504681	2.56E-08
0.359838205	6.67E-09	0.428562839	2.30E-12	-0.253291427	6.07E-05
-0.443724756	3.05E-13	-0.438135686	6.50E-13	0.366513244	3.33E-09
0.39761077	1.05E-10	0.376549767	1.13E-09	-0.193740866	0.002319637
-0.204353739	0.001298584	-0.346207089	2.63E-08	0.143180612	0.025011189
-0.445649086	2.35E-13	-0.578323133	2.88E-23	0.376469056	1.14E-09
-0.393068938	1.77E-10	-0.421767985	5.49E-12	0.424096369	4.08E-12
-0.515587287	4.85E-18	-0.445763335	2.31E-13	0.207250419	0.001102818
-0.389120267	2.79E-10	-0.325693433	1.84E-07	0.391503254	2.13E-10
-0.572702265	9.41E-23	-0.506531364	2.26E-17	0.258857953	4.11E-05
0.544904095	2.40E-20	0.523627526	1.19E-18	0.007440313	0.907759262
0.157274645	0.01372027	0.398714494	9.19E-11	-0.343296778	3.50E-08
-0.132226942	0.038622705	-0.220000375	0.00052331	0.203340527	0.001374261
0.65828645	8.25E-32	0.485077284	7.27E-16	-0.436937041	7.63E-13
-0.61169989	1.54E-26	-0.476233172	2.84E-15	0.416582238	1.06E-11
-0.197108256	0.001935714	-0.36156194	5.58E-09	0.530907567	3.22E-19
-0.354723457	1.13E-08	-0.49370057	1.86E-16	-0.003956972	0.950865528
0.196477597	0.002002874	0.392661003	1.86E-10	-0.046799459	0.465891041

-0.420341091	6.58E-12	-0.408686505	2.79E-11	0.509976323	1.27E-17
-0.645772633	2.65E-30	-0.557435481	2.10E-21	0.346978071	2.44E-08
0.385192165	4.35E-10	0.309437153	7.79E-07	-0.452525682	9.03E-14
0.342383025	3.82E-08	0.224316325	0.00040264	-0.496761695	1.13E-16
-0.665395192	1.07E-32	-0.470744293	6.47E-15	0.542097932	4.07E-20
-0.592107656	1.43E-24	-0.421837876	5.45E-12	0.485356604	6.96E-16
-0.573290494	8.32E-23	-0.571679756	1.16E-22	0.534655416	1.63E-19
-0.4199511	6.91E-12	-0.465443768	1.42E-14	0.122679773	0.055152646
-0.595596621	6.51E-25	-0.596865834	4.89E-25	0.385309926	4.29E-10
-0.694457763	1.34E-36	-0.648385353	1.30E-30	0.440375643	4.81E-13
-0.388247974	3.08E-10	-0.227000456	0.000341209	0.272467116	1.52E-05
-0.330602993	1.17E-07	-0.394993115	1.42E-10	0.368943892	2.57E-09
0.685024116	2.78E-35	0.509028079	1.49E-17	-0.484621098	7.81E-16
-0.561530856	9.28E-22	-0.460024657	3.11E-14	0.515265362	5.13E-18
-0.491390766	2.68E-16	-0.485367496	6.95E-16	0.404738007	4.50E-11
-0.560295957	1.19E-21	-0.559373706	1.43E-21	0.408577507	2.83E-11
-0.521808637	1.64E-18	-0.405537387	4.08E-11	0.449075314	1.46E-13
-0.684056557	3.77E-35	-0.621002736	1.61E-27	0.64648485	2.18E-30
-0.506639845	2.22E-17	-0.613093521	1.10E-26	0.334151095	8.40E-08
-0.450619815	1.18E-13	-0.372095477	1.84E-09	0.537979778	8.80E-20
-0.451754413	1.01E-13	-0.445269171	2.47E-13	0.314512889	5.01E-07
-0.488242432	4.42E-16	-0.501459485	5.25E-17	0.287938992	4.62E-06
-0.396147971	1.24E-10	-0.441839652	3.94E-13	0.219774333	0.000530471
-0.459482143	3.36E-14	-0.589244131	2.69E-24	0.279358216	9.04E-06
-0.417061074	9.94E-12	-0.416395807	1.08E-11	0.489591264	3.57E-16
0.25692238	4.71E-05	0.157279227	0.013717486	-0.202544417	0.001436535
0.580638399	1.75E-23	0.368040031	2.83E-09	-0.54374465	2.98E-20
-0.555099903	3.33E-21	-0.592902361	1.19E-24	0.280811464	8.08E-06
0.448741283	1.53E-13	0.442578354	3.57E-13	-0.222245256	0.000456895
0.558999945	1.54E-21	0.274617942	1.30E-05	-0.293284861	3.01E-06
-0.459387725	3.41E-14	-0.426745035	2.91E-12	0.443914503	2.97E-13
0.481352117	1.30E-15	0.496122403	1.26E-16	-0.171591205	0.007100629
-0.544529209	2.57E-20	-0.479744456	1.66E-15	0.572131878	1.06E-22
0.625654491	5.04E-28	0.620567169	1.79E-27	-0.424483166	3.89E-12
0.649271172	1.02E-30	0.642982105	5.61E-30	-0.424080174	4.09E-12
0.429414838	2.06E-12	0.555071806	3.35E-21	-0.26685044	2.31E-05
0.543894738	2.90E-20	0.491028676	2.84E-16	0.086992007	0.174701427
-0.268499946	2.05E-05	-0.362913767	4.85E-09	-0.052155322	0.416368104
-0.409576981	2.50E-11	-0.426100502	3.16E-12	0.166672506	0.008954042
0.530839803	3.26E-19	0.353418589	1.28E-08	-0.393209298	1.75E-10

-0.590328867	2.12E-24	-0.509945102	1.27E-17	0.108918942	0.088905969
-0.53179067	2.75E-19	-0.538061034	8.67E-20	0.379005668	8.66E-10
-0.647317714	1.74E-30	-0.575852357	4.86E-23	0.448024869	1.69E-13
-0.651033403	6.28E-31	-0.544115554	2.78E-20	0.451285515	1.07E-13
0.637943642	2.14E-29	0.608488907	3.31E-26	-0.392972872	1.79E-10
-0.472314858	5.12E-15	-0.517829102	3.29E-18	0.209731942	0.000957088
-0.448218346	1.65E-13	-0.580373861	1.86E-23	0.127756909	0.045751508
-0.591387914	1.67E-24	-0.56147137	9.39E-22	0.286823266	5.05E-06
-0.368988478	2.56E-09	-0.504044507	3.42E-17	0.236572561	0.000186083
-0.329451935	1.30E-07	-0.394365896	1.53E-10	0.270760605	1.73E-05
0.359243891	7.09E-09	0.479886856	1.62E-15	-0.250040903	7.60E-05
0.150851548	0.018144252	0.191880395	0.002560324	-0.286949881	5.00E-06
-0.552262024	5.81E-21	-0.55153935	6.69E-21	0.291963145	3.35E-06
-0.507180769	2.03E-17	-0.421818019	5.46E-12	0.561348324	9.63E-22
-0.33574999	7.22E-08	-0.437388146	7.18E-13	0.405530497	4.09E-11
0.514172329	6.19E-18	0.472411802	5.04E-15	-0.389094755	2.80E-10
-0.626240885	4.35E-28	-0.615898063	5.61E-27	0.356735078	9.17E-09
-0.367413386	3.03E-09	-0.348216184	2.16E-08	0.274017676	1.36E-05
-0.691215135	3.86E-36	-0.568825534	2.10E-22	0.253167669	6.13E-05
-0.446135251	2.19E-13	-0.462895932	2.05E-14	0.191043625	0.002675801
-0.322704377	2.42E-07	-0.321871104	2.60E-07	0.268459194	2.05E-05
-0.418236616	8.58E-12	-0.483930532	8.69E-16	0.05074527	0.429097666
-0.492252212	2.34E-16	-0.518807504	2.78E-18	0.289553871	4.06E-06
-0.257804964	4.43E-05	-0.585405015	6.26E-24	0.167486812	0.008620285
0.508335292	1.67E-17	0.506221816	2.38E-17	-0.210821029	0.00089891
0.053323423	0.405992526	-0.135494035	0.034026624	-0.12609408	0.048670508
0.339274254	5.16E-08	0.34644434	2.57E-08	-0.196831656	0.001964913
0.584292778	7.97E-24	0.347161646	2.40E-08	-0.261422482	3.42E-05
-0.485442811	6.87E-16	-0.434200691	1.10E-12	0.477253306	2.43E-15
0.486666642	5.67E-16	0.463681104	1.83E-14	-0.224567818	0.000396476
0.552322078	5.74E-21	0.540428128	5.57E-20	0.035621668	0.578968527
0.658198456	8.46E-32	0.418770767	8.02E-12	-0.415405388	1.22E-11
-0.414983331	1.29E-11	-0.489630217	3.55E-16	0.315333967	4.66E-07
-0.230795999	0.000269097	-0.156011022	0.014507048	0.175858746	0.005778532
-0.396165439	1.24E-10	-0.347410042	2.34E-08	0.477503322	2.34E-15
0.233271568	0.000230006	0.118573114	0.063880411	-0.269746173	1.87E-05
-0.638182893	2.01E-29	-0.649996769	8.36E-31	0.302920228	1.36E-06
-0.45903722	3.58E-14	-0.503113228	4.00E-17	0.235029365	0.000205538
0.427977549	2.48E-12	0.261681177	3.36E-05	-0.282703142	6.98E-06
-0.404755639	4.49E-11	-0.246924548	9.39E-05	0.218546528	0.000570978

-0.307509203	9.20E-07	-0.321447361	2.71E-07	0.277727623	1.02E-05
-0.575793056	4.92E-23	-0.657178892	1.13E-31	0.449165866	1.44E-13
-0.481720776	1.22E-15	-0.393054748	1.78E-10	0.068059586	0.288651796
0.487788181	4.75E-16	0.536468938	1.16E-19	-0.292402189	3.23E-06
-0.613565255	9.86E-27	-0.553780346	4.32E-21	0.44958632	1.36E-13
-0.574961361	5.86E-23	-0.505858784	2.53E-17	0.108824223	0.089185693
-0.466392678	1.23E-14	-0.468360543	9.22E-15	0.060023957	0.349498436
0.457008747	4.79E-14	0.49476391	1.56E-16	-0.438395003	6.28E-13
-0.576251587	4.46E-23	-0.561202913	9.91E-22	0.450442709	1.21E-13
0.473457471	4.31E-15	0.293027839	3.07E-06	-0.237250523	0.000178092
-0.408460488	2.87E-11	-0.433284506	1.24E-12	0.140435432	0.027961662
-0.560910319	1.05E-21	-0.494349197	1.67E-16	0.202342519	0.001452734
-0.508494038	1.63E-17	-0.40747542	3.23E-11	0.334458929	8.16E-08
-0.458113929	4.09E-14	-0.454627789	6.71E-14	0.417980619	8.86E-12
-0.340939339	4.40E-08	-0.374998629	1.34E-09	0.288054473	4.58E-06
-0.410531594	2.23E-11	-0.288077531	4.57E-06	0.296238107	2.36E-06
0.619903275	2.11E-27	0.58558291	6.02E-24	-0.554354258	3.86E-21
0.562208372	8.10E-22	0.49820768	8.95E-17	-0.231377935	0.000259388
-0.361035957	5.90E-09	-0.323005078	2.35E-07	0.290469443	3.78E-06
0.408047424	3.02E-11	0.556275626	2.64E-21	0.038169383	0.552106396
0.450155207	1.26E-13	0.196707579	0.001978141	-0.17611567	0.005706479
0.461021785	2.69E-14	0.57633205	4.39E-23	-0.317413527	3.88E-07
0.334821571	7.88E-08	0.371650828	1.93E-09	-0.507823836	1.82E-17
-0.476819717	2.59E-15	-0.580049046	1.99E-23	0.406408806	3.68E-11
0.687866958	1.13E-35	0.45298117	8.47E-14	-0.42896623	2.18E-12
0.548383077	1.23E-20	0.378185403	9.48E-10	-0.517223413	3.66E-18
0.51982061	2.33E-18	0.427511592	2.63E-12	-0.122581621	0.055349083
-0.454055001	7.28E-14	-0.428656015	2.27E-12	0.625158174	5.71E-28
0.442365174	3.67E-13	0.406332209	3.71E-11	-0.384784579	4.56E-10
0.360131318	6.47E-09	0.473145771	4.52E-15	-0.07321647	0.253582402
-0.519083937	2.64E-18	-0.485444925	6.86E-16	0.452458538	9.11E-14
-0.337133029	6.33E-08	-0.353181421	1.31E-08	0.009828102	0.878357969
-0.114175488	0.074451724	-0.243468117	0.000118371	0.014145519	0.82564445
0.665303898	1.10E-32	0.536146236	1.24E-19	-0.440179434	4.94E-13
0.373059041	1.66E-09	0.179096893	0.004927384	-0.126754197	0.047493626
-0.21957245	0.000536943	-0.304171635	1.22E-06	0.152764875	0.016712042
0.440108378	4.99E-13	0.35212651	1.46E-08	-0.445374999	2.44E-13
0.429142452	2.13E-12	0.423273384	4.54E-12	-0.19743295	0.001901944
-0.4307294	1.73E-12	-0.370190161	2.25E-09	0.230053044	0.000281985
0.56003214	1.25E-21	0.449680519	1.34E-13	-0.386777106	3.64E-10

0.352732709	1.38E-08	0.298305023	1.99E-06	-0.231526364	0.000256964
-0.544340805	2.67E-20	-0.578501327	2.77E-23	0.341702697	4.08E-08
-0.399032566	8.86E-11	-0.440738978	4.58E-13	0.289105511	4.21E-06
-0.300290397	1.69E-06	-0.28273084	6.96E-06	0.167900624	0.008454951
-0.351838848	1.50E-08	-0.52492068	9.46E-19	0.139575689	0.028944977
-0.439492183	5.42E-13	-0.479782095	1.65E-15	0.286589353	5.14E-06
-0.333885375	8.61E-08	-0.349550782	1.89E-08	0.378931851	8.73E-10
0.432971819	1.29E-12	0.444907844	2.60E-13	-0.147666737	0.02076557
-0.453747131	7.60E-14	-0.522713008	1.40E-18	0.215423485	0.000687262
-0.59691921	4.83E-25	-0.503412373	3.80E-17	0.400986342	7.03E-11
0.516833586	3.91E-18	0.663069269	2.10E-32	-0.456375095	5.24E-14
-0.195926514	0.002063293	-0.316598848	4.17E-07	-0.043464006	0.498304318
0.357541604	8.45E-09	0.268636524	2.03E-05	-0.169753237	0.007748803
-0.112293984	0.079388947	-0.372412991	1.77E-09	0.190134607	0.002806592
0.157657546	0.013489375	0.531399829	2.95E-19	-0.429791283	1.96E-12
0.494909967	1.53E-16	0.39997112	7.93E-11	-0.109913503	0.086011055
0.320998732	2.82E-07	0.469003205	8.38E-15	-0.367724591	2.93E-09
0.458384539	3.94E-14	0.47901279	1.86E-15	-0.275150092	1.25E-05
0.425377601	3.46E-12	0.261165366	3.49E-05	-0.273614533	1.40E-05
0.403017201	5.52E-11	0.344663163	3.06E-08	-0.300117498	1.72E-06
0.528630644	4.87E-19	0.464910852	1.53E-14	-0.571140965	1.30E-22
-0.276177884	1.15E-05	-0.356225831	9.66E-09	0.241102086	0.000138453
-0.394693359	1.47E-10	-0.309969978	7.44E-07	0.520992173	1.89E-18
0.183321874	0.003986767	0.220080702	0.000520786	-0.279515659	8.93E-06
0.743333848	2.52E-44	0.555143449	3.31E-21	-0.373708159	1.54E-09
-0.746086454	8.22E-45	-0.628073069	2.74E-28	0.433866968	1.15E-12
-0.369479965	2.43E-09	-0.474302172	3.80E-15	0.347133594	2.40E-08
-0.317259592	3.93E-07	-0.415273852	1.24E-11	0.162960246	0.010625276
-0.274992668	1.26E-05	-0.371009171	2.06E-09	0.34447523	3.12E-08
-0.381295156	6.72E-10	-0.354522288	1.15E-08	0.470122511	7.10E-15
-0.393590106	1.67E-10	-0.346514995	2.55E-08	0.238020617	0.000169404
0.516545392	4.11E-18	0.567507475	2.76E-22	-0.396279089	1.22E-10
0.531539692	2.87E-19	0.267504159	2.20E-05	-0.457908464	4.21E-14
-0.056633887	0.377433095	-0.109422248	0.08743137	0.06923558	0.280381843
0.280305706	8.40E-06	0.19316032	0.002392434	-0.160943523	0.011644154
0.145872721	0.022381982	0.227789721	0.000324877	0.30310645	1.34E-06
-0.447797963	1.74E-13	-0.277921206	1.01E-05	0.304232295	1.22E-06
-0.566054093	3.71E-22	-0.486784968	5.56E-16	0.499683469	7.03E-17
-0.588315229	3.31E-24	-0.509580189	1.35E-17	0.393826383	1.63E-10
-0.40056813	7.39E-11	-0.176476879	0.00560654	0.321601715	2.67E-07

-0.476700857	2.64E-15	-0.303062394	1.34E-06	0.287485982	4.79E-06
0.429533451	2.02E-12	0.508470821	1.63E-17	-0.223296495	0.000428561
0.550663251	7.93E-21	0.543139443	3.35E-20	-0.225247587	0.000380249
0.506651162	2.22E-17	0.394253362	1.55E-10	-0.207232542	0.001103938
0.287959624	4.61E-06	0.526427446	7.23E-19	0.321128724	2.78E-07
-0.544966856	2.37E-20	-0.245222598	0.00010528	0.595707358	6.35E-25
-0.382386995	5.96E-10	-0.494154993	1.72E-16	0.132531882	0.038172623
-0.421447352	5.72E-12	-0.415557299	1.20E-11	0.084914102	0.185266514
-0.629332764	1.99E-28	-0.580008222	2.01E-23	0.361725188	5.49E-09
0.228559881	0.000309644	0.24810622	8.67E-05	-0.334784374	7.91E-08
-0.486579385	5.75E-16	-0.565090795	4.52E-22	0.185615444	0.003547049
0.480152223	1.56E-15	0.409957316	2.39E-11	-0.418060232	8.77E-12
-0.505436241	2.72E-17	-0.589139993	2.76E-24	0.371049375	2.05E-09
-0.317555463	3.83E-07	-0.298977991	1.89E-06	0.449542577	1.37E-13
0.469339759	7.97E-15	0.323222341	2.31E-07	-0.442501291	3.61E-13
0.192666131	0.002456033	0.205915187	0.001189417	-0.362051859	5.31E-09
-0.374109772	1.48E-09	-0.479688234	1.67E-15	0.136845804	0.032265636
-0.288205429	4.52E-06	-0.353419305	1.28E-08	0.146408765	0.021887986
-0.2278425	0.000323811	-0.285588315	5.57E-06	-0.130164321	0.041786232
-0.564430721	5.17E-22	-0.477220333	2.44E-15	0.228669139	0.000307537
-0.444537565	2.73E-13	-0.507970398	1.78E-17	0.227920147	0.000322249
-0.306627259	9.92E-07	-0.298591384	1.95E-06	0.196764859	0.001972024
-0.268019536	2.12E-05	-0.46019791	3.03E-14	0.286426737	5.21E-06
-0.51541708	5.00E-18	-0.521857826	1.63E-18	0.424901566	3.68E-12
-0.487927617	4.65E-16	-0.439861191	5.15E-13	0.58182915	1.36E-23
-0.246438477	9.70E-05	-0.344501285	3.11E-08	0.248032194	8.71E-05
-0.51591246	4.59E-18	-0.49835979	8.73E-17	0.396635309	1.17E-10
0.279894069	8.67E-06	0.243920984	0.000114853	-0.142444681	0.025774608
0.235696454	0.000196906	0.218473611	0.000573471	-0.126442938	0.048045555
-0.275795321	1.19E-05	-0.245138578	0.000105874	0.370789947	2.11E-09
0.208050446	0.001053747	0.06898987	0.282096396	-0.454125028	7.21E-14
-0.372180627	1.82E-09	-0.378165789	9.50E-10	0.291561728	3.46E-06
0.224694299	0.000393409	0.055374283	0.388151544	-0.389304382	2.73E-10
-0.378280233	9.38E-10	-0.357481508	8.50E-09	0.397066738	1.12E-10
-0.462544997	2.16E-14	-0.359020532	7.26E-09	0.2472294	9.20E-05
-0.268907413	1.99E-05	-0.055916386	0.383516179	0.295730066	2.46E-06
0.351767449	1.52E-08	0.288772895	4.32E-06	-0.220514757	0.000507346
0.520391997	2.10E-18	0.458469552	3.89E-14	-0.358496396	7.66E-09
0.355742598	1.01E-08	0.632997469	7.76E-29	0.026756851	0.676865831
-0.692274552	2.74E-36	-0.628990091	2.17E-28	0.489116216	3.85E-16

0.604286117	8.87E-26	0.42900926	2.17E-12	-0.226550812	0.000350852
0.528974242	4.57E-19	0.366600763	3.30E-09	-0.364815883	3.98E-09
0.391289835	2.18E-10	0.519930651	2.28E-18	0.175859247	0.005778391
-0.391898445	2.03E-10	-0.506881766	2.13E-17	0.116869702	0.067818933
0.526822829	6.73E-19	0.535013701	1.52E-19	-0.448040974	1.69E-13
-0.493671636	1.86E-16	-0.437082379	7.48E-13	0.450562996	1.19E-13
0.547167757	1.55E-20	0.637835408	2.20E-29	0.174274528	0.006241115
0.468551406	8.96E-15	0.523959905	1.12E-18	-0.196655518	0.001983715
0.641989085	7.32E-30	0.535502333	1.39E-19	-0.515896858	4.60E-18
0.617175088	4.11E-27	0.364872868	3.95E-09	-0.319378624	3.26E-07
0.506949482	2.11E-17	0.577869255	3.17E-23	-0.453872588	7.47E-14
0.623644339	8.33E-28	0.426856925	2.86E-12	-0.10317159	0.107194135
0.533283352	2.09E-19	0.742699369	3.26E-44	-0.337460849	6.14E-08
-0.350205908	1.77E-08	-0.217683126	0.000601157	0.364769969	4.00E-09
0.649730813	9.00E-31	0.423584342	4.36E-12	-0.398149773	9.82E-11
-0.514724145	5.63E-18	-0.529514758	4.15E-19	0.34708701	2.41E-08
-0.219080013	0.000553038	-0.163605157	0.010316517	0.423338051	4.50E-12
-0.64863802	1.21E-30	-0.618635899	2.88E-27	0.218204415	0.000582764
0.491505959	2.64E-16	0.34199962	3.97E-08	-0.323455786	2.26E-07
0.397104252	1.11E-10	0.297508839	2.13E-06	-0.433197291	1.25E-12
-0.428743238	2.24E-12	-0.478163791	2.11E-15	0.429394804	2.06E-12
-0.280753334	8.12E-06	-0.404067776	4.87E-11	0.402198511	6.09E-11
-0.444884434	2.61E-13	-0.538902982	7.41E-20	0.259605579	3.90E-05
-0.593821726	9.71E-25	-0.530358427	3.56E-19	0.444577753	2.72E-13
0.663001738	2.14E-32	0.485601352	6.70E-16	-0.327607566	1.54E-07
-0.538519745	7.96E-20	-0.305318228	1.11E-06	0.4794965	1.72E-15
-0.459263374	3.47E-14	-0.423585299	4.36E-12	0.492449288	2.27E-16
-0.151668256	0.017520341	-0.252044021	6.62E-05	0.179851364	0.004746029
-0.394815948	1.45E-10	-0.464062236	1.73E-14	0.305611482	1.08E-06
0.595524973	6.62E-25	0.453113683	8.31E-14	-0.149710981	0.019047884
0.56060942	1.12E-21	0.436579342	8.00E-13	-0.273428273	1.42E-05
0.272195923	1.56E-05	0.408908313	2.72E-11	0.146563403	0.021747242
0.590458897	2.06E-24	0.362269094	5.19E-09	-0.260938926	3.54E-05
-0.586083166	5.39E-24	-0.540823598	5.18E-20	0.368571134	2.68E-09
-0.582981035	1.06E-23	-0.523397548	1.24E-18	0.331699626	1.06E-07
-0.234635405	0.000210801	-0.320264026	3.01E-07	0.275422734	1.22E-05
-0.548447009	1.22E-20	-0.278957345	9.32E-06	0.211582606	0.000860183
0.723224709	6.05E-41	0.495120486	1.48E-16	-0.260935155	3.55E-05
-0.092993897	0.146699839	-0.292970305	3.09E-06	-0.072891179	0.255703347
-0.303529006	1.29E-06	-0.182441742	0.004168164	0.282510105	7.08E-06

0.331359307	1.09E-07	0.364096706	4.29E-09	-0.170610359	0.007440248
0.302837562	1.37E-06	0.377375554	1.04E-09	-0.422097407	5.27E-12
-0.644312189	3.93E-30	-0.504703344	3.07E-17	0.391832392	2.05E-10
0.645884841	2.57E-30	0.666155073	8.55E-33	-0.322161848	2.54E-07
-0.439920387	5.11E-13	-0.577311334	3.57E-23	0.183454378	0.003960084
-0.414047093	1.45E-11	-0.331978991	1.03E-07	0.261176956	3.48E-05
-0.372390138	1.78E-09	-0.410641898	2.20E-11	0.320309864	3.00E-07
-0.4466101	2.06E-13	-0.472981185	4.63E-15	0.34960062	1.88E-08
-0.247208594	9.21E-05	-0.323164262	2.32E-07	0.319745464	3.15E-07
-0.306834382	9.74E-07	-0.375339814	1.29E-09	0.10322616	0.107007531
0.38407549	4.93E-10	0.568384001	2.30E-22	0.031794118	0.620432937
-0.392673613	1.86E-10	-0.530237456	3.64E-19	0.106952956	0.094857742
-0.489452511	3.65E-16	-0.538218208	8.42E-20	0.277767215	1.02E-05
-0.218830597	0.000561359	-0.342311694	3.85E-08	0.186108665	0.003458421
-0.154884972	0.015241201	-0.057165243	0.372966493	0.282912969	6.86E-06
-0.596447735	5.37E-25	-0.542749933	3.60E-20	0.372031499	1.85E-09
-0.637022515	2.72E-29	-0.404642678	4.55E-11	0.415349535	1.23E-11
0.711445683	4.23E-39	0.746338325	7.41E-45	-0.252479676	6.42E-05
-0.633165615	7.43E-29	-0.545603724	2.10E-20	0.468089318	9.60E-15
0.483934895	8.69E-16	0.491405226	2.68E-16	-0.116329185	0.069109395
-0.291516113	3.47E-06	-0.268994752	1.97E-05	0.323896464	2.17E-07
-0.355274772	1.06E-08	-0.411260386	2.04E-11	0.45517827	6.21E-14
0.225927969	0.000364627	0.388561827	2.97E-10	0.151603473	0.017569139
-0.260296314	3.71E-05	-0.391284244	2.18E-10	0.238928	0.000159677
-0.377334033	1.04E-09	-0.322385918	2.49E-07	0.424880354	3.69E-12
0.592652028	1.26E-24	0.323238239	2.30E-07	-0.315112858	4.75E-07
0.330246897	1.21E-07	0.341729607	4.07E-08	-0.071204295	0.266898389
-0.5027796	4.22E-17	-0.473262172	4.44E-15	0.489616073	3.56E-16
0.542181067	4.01E-20	0.320611614	2.92E-07	-0.318014247	3.68E-07
0.210329623	0.000924745	0.173578475	0.006454633	-0.044947986	0.483737901
-0.681974857	7.23E-35	-0.553965831	4.17E-21	0.446120861	2.20E-13
-0.492561184	2.23E-16	-0.50970157	1.33E-17	0.512722359	7.94E-18
-0.374214425	1.46E-09	-0.454661233	6.68E-14	-0.173345423	0.006527569
-0.40330285	5.34E-11	-0.368156978	2.80E-09	0.395435597	1.35E-10
0.652186901	4.57E-31	0.439418068	5.47E-13	-0.467513245	1.04E-14
0.267857971	2.15E-05	0.291115846	3.58E-06	-0.161754865	0.01122433
0.311404075	6.57E-07	0.527470935	5.99E-19	0.004192593	0.947943795
-0.339893381	4.86E-08	-0.375633859	1.25E-09	0.355937176	9.95E-09
0.417559232	9.34E-12	0.193215347	0.002385446	-0.400561274	7.39E-11
-0.232367518	0.000243623	-0.339921263	4.85E-08	0.136928898	0.032159967

0.391752276	2.07E-10	0.60106118	1.87E-25	-0.253339689	6.05E-05
-0.190192145	0.002798145	-0.251076748	7.08E-05	0.144305114	0.023882282
-0.332049635	1.02E-07	-0.223473668	0.00042395	0.279818667	8.72E-06
0.435206444	9.60E-13	0.406927808	3.45E-11	-0.590103294	2.23E-24
0.54556413	2.11E-20	0.506961947	2.10E-17	-0.265001701	2.64E-05
-0.543376921	3.20E-20	-0.481963826	1.18E-15	0.594160631	9.00E-25
-0.413159839	1.61E-11	-0.42254672	4.98E-12	0.511199279	1.03E-17
-0.621691953	1.35E-27	-0.59147932	1.64E-24	0.377747647	9.94E-10
-0.513230359	7.27E-18	-0.367555518	2.98E-09	0.037725328	0.556744275
-0.506606979	2.23E-17	-0.52326905	1.27E-18	0.328068685	1.48E-07
-0.447479412	1.82E-13	-0.510526899	1.15E-17	0.378057719	9.61E-10
-0.479432735	1.74E-15	-0.333800916	8.68E-08	0.642081797	7.14E-30
-0.517296935	3.61E-18	-0.464482077	1.63E-14	0.328750786	1.39E-07
-0.446866076	1.98E-13	-0.383320392	5.37E-10	0.601626132	1.64E-25
0.432269896	1.42E-12	0.492348443	2.30E-16	-0.606994746	4.71E-26
0.433290299	1.24E-12	0.184907512	0.00367785	-0.179853271	0.004745578
-0.532279339	2.51E-19	-0.29806247	2.03E-06	0.332878673	9.47E-08
-0.64193056	7.44E-30	-0.65611344	1.53E-31	0.367004606	3.16E-09
-0.545784626	2.03E-20	-0.486209311	6.09E-16	0.352223786	1.45E-08
0.597404577	4.32E-25	0.634201673	5.68E-29	-0.472449298	5.02E-15
0.448071636	1.68E-13	0.268816498	2.00E-05	-0.210534095	0.000913913
0.370082008	2.28E-09	0.275046139	1.26E-05	0.195563006	0.002104052
0.668901054	3.81E-33	0.578346069	2.86E-23	-0.454572413	6.77E-14
-0.37185477	1.88E-09	-0.400095936	7.81E-11	0.441437562	4.17E-13
0.495231676	1.45E-16	0.409610579	2.49E-11	0.048251622	0.452151509
0.247372528	9.11E-05	0.189673367	0.002875151	-0.381138825	6.84E-10
-0.266401709	2.39E-05	-0.237119916	0.000179606	0.239409304	0.000154731
-0.183140515	0.004023552	-0.379439913	8.25E-10	0.249806385	7.72E-05
0.399604055	8.28E-11	0.309629371	7.67E-07	-0.118475071	0.064101891
-0.629632357	1.84E-28	-0.536000835	1.27E-19	0.247778766	8.86E-05
0.571981429	1.09E-22	0.404985967	4.36E-11	-0.150294737	0.018580624
-0.635228413	4.35E-29	-0.474982165	3.43E-15	0.433944808	1.13E-12
-0.467905108	9.86E-15	-0.518528781	2.91E-18	0.381322504	6.70E-10
-0.396659479	1.17E-10	-0.383586713	5.21E-10	-0.01971002	0.758871768
-0.585055679	6.75E-24	-0.623554508	8.52E-28	0.367978914	2.85E-09
-0.315462956	4.61E-07	-0.273177303	1.45E-05	0.295867676	2.44E-06
-0.504128125	3.38E-17	-0.348926202	2.01E-08	0.497682595	9.75E-17
-0.31441639	5.05E-07	-0.325531075	1.87E-07	0.244699519	0.000109034
-0.320895454	2.84E-07	-0.349194805	1.96E-08	0.291925494	3.36E-06
0.535156004	1.48E-19	0.454690045	6.66E-14	-0.125609483	0.049549872

0.604630039	8.19E-26	0.487531728	4.95E-16	-0.369203355	2.50E-09
-0.14311678	0.025076625	-0.241236286	0.000137233	0.033870673	0.597776964
-0.516300428	4.29E-18	-0.526898332	6.64E-19	0.256113839	4.99E-05
-0.309556113	7.71E-07	-0.226233383	0.000357811	0.061128986	0.340682231
-0.40833925	2.91E-11	-0.357092955	8.84E-09	0.431068589	1.66E-12
-0.466931083	1.14E-14	-0.361617479	5.55E-09	0.274065684	1.35E-05
-0.304004942	1.24E-06	-0.326552779	1.70E-07	0.236813303	0.000183208
-0.023610901	0.713074325	-0.17430916	0.006230659	-0.003830834	0.952429933
-0.552423145	5.63E-21	-0.46192748	2.36E-14	0.468310169	9.29E-15
0.555959461	2.81E-21	0.410674626	2.19E-11	-0.080322067	0.210265823
-0.30514723	1.12E-06	-0.308505329	8.44E-07	0.154710453	0.015357845
-0.350654865	1.69E-08	-0.348301329	2.14E-08	0.25821231	4.30E-05
0.548009898	1.32E-20	0.297655365	2.10E-06	-0.066293904	0.30137258
0.307269696	9.39E-07	0.481092984	1.35E-15	-0.484621712	7.80E-16
-0.279541168	8.91E-06	-0.229187616	0.000297722	0.333495076	8.93E-08
0.42604654	3.18E-12	0.483419351	9.41E-16	-0.426857056	2.86E-12
0.463030296	2.01E-14	0.414016795	1.45E-11	0.127989424	0.045355269
-0.431871709	1.49E-12	-0.434888295	1.00E-12	0.422170515	5.22E-12
0.559171355	1.49E-21	0.453355989	8.03E-14	-0.410286556	2.30E-11
-0.189331492	0.002926944	-0.303398801	1.30E-06	0.121980607	0.056564614
-0.463429164	1.90E-14	-0.527033873	6.48E-19	0.383654184	5.17E-10
0.538072855	8.65E-20	0.640144812	1.20E-29	-0.503182826	3.95E-17
-0.413185291	1.61E-11	-0.421156503	5.94E-12	0.415126534	1.26E-11
0.345404592	2.85E-08	0.308138363	8.71E-07	-0.077620516	0.226061346
0.618398888	3.05E-27	0.458458134	3.89E-14	0.018682475	0.771084285
0.42688667	2.85E-12	0.280549001	8.25E-06	-0.328344973	1.44E-07
-0.541465045	4.59E-20	-0.506355818	2.33E-17	0.502955969	4.10E-17
-0.607688384	4.00E-26	-0.562759112	7.25E-22	0.218770642	0.000563377
-0.494382301	1.66E-16	-0.402154262	6.12E-11	0.207904874	0.001062524
0.547327451	1.51E-20	0.425807229	3.28E-12	0.077533972	0.226580898
0.265831177	2.49E-05	0.284430006	6.10E-06	-0.09347583	0.1446087
0.434530337	1.05E-12	0.339512869	5.04E-08	-0.482143048	1.15E-15
-0.219556995	0.000537441	-0.240646803	0.000142666	0.182869288	0.004079137
-0.490133034	3.28E-16	-0.478314078	2.07E-15	0.036249456	0.57229302
-0.338267093	5.68E-08	-0.333922074	8.58E-08	0.331952241	1.03E-07
-0.529251998	4.35E-19	-0.532010301	2.64E-19	0.350103586	1.79E-08
0.467417219	1.06E-14	0.342478513	3.79E-08	-0.167849119	0.008475374
0.448984236	1.48E-13	0.2531558	6.13E-05	-0.470740632	6.48E-15
-0.485340504	6.98E-16	-0.46257867	2.15E-14	0.092807976	0.147512676
0.203941705	0.001328886	0.18630449	0.003423792	-0.203479671	0.001363634

0.365915393	3.54E-09	0.248317243	8.54E-05	-0.347002278	2.43E-08
0.583274526	9.94E-24	0.53637036	1.19E-19	-0.426930172	2.84E-12
0.450073419	1.27E-13	0.362388492	5.12E-09	-0.400658911	7.31E-11
0.500009104	6.67E-17	0.303140488	1.33E-06	-0.361524621	5.61E-09
0.544807031	2.44E-20	0.686704175	1.64E-35	-0.236436434	0.000187727
0.434854513	1.01E-12	0.227560889	0.000329536	-0.340939785	4.40E-08
-0.244712949	0.000108936	-0.238820812	0.000160798	0.283272943	6.67E-06
-0.20128217	0.001540588	-0.284824323	5.91E-06	-0.004613437	0.942727012
-0.634443243	5.34E-29	-0.636747332	2.93E-29	0.18287081	0.004078823
-0.52336806	1.25E-18	-0.335870865	7.14E-08	0.632550398	8.71E-29
0.555965553	2.81E-21	0.437551127	7.03E-13	-0.32017925	3.03E-07
-0.523670875	1.18E-18	-0.378215001	9.45E-10	0.595539821	6.60E-25
0.704527805	4.64E-38	0.58494822	6.91E-24	-0.498492049	8.54E-17
0.617019688	4.27E-27	0.467784818	1.00E-14	-0.333517759	8.91E-08
-0.522876938	1.36E-18	-0.443079196	3.33E-13	0.300584137	1.65E-06
-0.676807759	3.55E-34	-0.559922254	1.28E-21	0.607781177	3.91E-26
-0.313485127	5.48E-07	-0.385352719	4.27E-10	0.265689804	2.52E-05
-0.570986543	1.34E-22	-0.567078576	3.01E-22	0.530122564	3.72E-19
-0.702284933	9.94E-38	-0.658254214	8.33E-32	0.318376669	3.56E-07
-0.405944647	3.89E-11	-0.447377132	1.85E-13	0.451323212	1.07E-13
-0.554318726	3.89E-21	-0.513432279	7.03E-18	0.411185348	2.06E-11
-0.584919818	6.95E-24	-0.510511677	1.16E-17	0.44514469	2.51E-13
-0.538543293	7.92E-20	-0.451325947	1.07E-13	0.397050735	1.12E-10
0.566490333	3.40E-22	0.300751974	1.63E-06	-0.442392399	3.66E-13
0.585477329	6.16E-24	0.379614726	8.10E-10	-0.469347448	7.97E-15
-0.236083082	0.000192059	-0.338712011	5.45E-08	-0.054274389	0.397660063
0.462085887	2.31E-14	0.328794453	1.38E-07	-0.310726208	6.97E-07
0.455331762	6.08E-14	0.35131585	1.59E-08	-0.3002798	1.69E-06
0.343355473	3.48E-08	0.169824466	0.007722734	-0.252583501	6.38E-05
0.572754961	9.31E-23	0.46780958	1.00E-14	-0.211333042	0.000872701
0.342492406	3.78E-08	0.324059707	2.14E-07	-0.050792135	0.428671004
0.099489561	0.120387339	0.028033588	0.662374724	-0.392654866	1.86E-10
-0.512000491	8.98E-18	-0.494556452	1.62E-16	0.216992466	0.000626353
0.162255253	0.010972119	0.19103103	0.002677575	-0.272062846	1.57E-05
0.552881599	5.15E-21	0.268638087	2.03E-05	-0.259120726	4.03E-05
0.513813529	6.58E-18	0.607736802	3.95E-26	0.01046257	0.870571909
-0.527967805	5.48E-19	-0.598711328	3.21E-25	0.309298737	7.89E-07
0.454601989	6.74E-14	0.30668402	9.87E-07	-0.400270518	7.65E-11
0.592982296	1.17E-24	0.529086936	4.48E-19	-0.355811206	1.01E-08
0.583080836	1.04E-23	0.435682478	9.01E-13	-0.400688924	7.28E-11

0.495698474	1.34E-16	0.344367785	3.15E-08	-0.270998024	1.70E-05
-0.512231292	8.63E-18	-0.39327709	1.73E-10	0.113919259	0.07510905
-0.31455326	4.99E-07	-0.326161672	1.76E-07	0.157945264	0.013318129
-0.520221313	2.17E-18	-0.548309101	1.25E-20	0.075972088	0.236103285
-0.524334398	1.05E-18	-0.492719808	2.17E-16	0.482198245	1.14E-15
-0.501548401	5.18E-17	-0.508366027	1.66E-17	0.373975389	1.50E-09
-0.130358228	0.041479829	-0.212110376	0.000834254	0.154583404	0.015443252
-0.553819361	4.29E-21	-0.510506038	1.16E-17	0.300401469	1.68E-06
-0.434694555	1.03E-12	-0.43317919	1.26E-12	0.312721443	5.86E-07
0.589400799	2.60E-24	0.332434261	9.87E-08	-0.333781727	8.70E-08
0.346988836	2.44E-08	0.297038377	2.21E-06	0.023752682	0.711427842
-0.370941339	2.08E-09	-0.343146495	3.55E-08	0.547895471	1.35E-20
-0.490239998	3.22E-16	-0.494477825	1.64E-16	0.252939766	6.22E-05
0.637433152	2.44E-29	0.475488868	3.17E-15	-0.346819593	2.48E-08
-0.414669901	1.34E-11	-0.490716214	2.99E-16	0.437013322	7.55E-13
-0.417986466	8.85E-12	-0.417708602	9.17E-12	0.245029561	0.000106651
-0.198279733	0.001816387	-0.389677826	2.62E-10	-0.033640953	0.600264852
0.729822857	5.09E-42	0.477805851	2.23E-15	-0.324845655	1.99E-07
-0.298658502	1.94E-06	-0.355358635	1.06E-08	0.164146899	0.010063311
-0.630434608	1.50E-28	-0.539422397	6.73E-20	0.542427806	3.83E-20
-0.629406812	1.95E-28	-0.464844353	1.55E-14	0.598489613	3.38E-25
-0.251770214	6.75E-05	-0.541651437	4.43E-20	0.346039873	2.68E-08
-0.330540826	1.18E-07	-0.431794602	1.51E-12	0.238463083	0.000164594
-0.39833694	9.61E-11	-0.210397912	0.000921114	0.250253955	7.49E-05
-0.243400924	0.000118902	-0.476615324	2.68E-15	0.150563891	0.018368562
-0.461167946	2.64E-14	-0.536716113	1.11E-19	0.257466477	4.54E-05
0.467670434	1.02E-14	0.344412192	3.14E-08	-0.386365059	3.81E-10
-0.266127221	2.44E-05	-0.512638673	8.05E-18	0.041334389	0.519606504
-0.392794735	1.83E-10	-0.502640934	4.32E-17	0.188401892	0.003072083
-0.477753362	2.25E-15	-0.469454345	7.84E-15	0.090750883	0.156735878
0.423419241	4.45E-12	0.288569204	4.39E-06	-0.679947089	1.36E-34
0.406629728	3.58E-11	0.309907857	7.48E-07	-0.464233354	1.69E-14
-0.232509167	0.000241441	-0.471380455	5.89E-15	0.023498507	0.714380475
-0.182495994	0.004156771	-0.201267436	0.001541842	0.372659989	1.73E-09
-0.410448621	2.25E-11	-0.417611522	9.28E-12	0.423015275	4.69E-12
-0.578579218	2.72E-23	-0.621733169	1.34E-27	0.270054326	1.83E-05
-0.556196071	2.69E-21	-0.492024758	2.43E-16	0.556930891	2.32E-21
0.444934033	2.59E-13	0.248441679	8.47E-05	-0.334798819	7.90E-08
-0.338343848	5.64E-08	-0.455119168	6.26E-14	0.15832136	0.013097164
-0.159098448	0.012650788	-0.200168013	0.001638117	0.238629628	0.000162816

0.621926127	1.28E-27	0.5176884	3.37E-18	-0.523065307	1.31E-18
0.527750415	5.70E-19	0.645437914	2.90E-30	-0.029233783	0.648865864
0.46808897	9.60E-15	0.360006702	6.56E-09	-0.276101237	1.16E-05
-0.299375446	1.82E-06	-0.122618162	0.055275885	0.266269447	2.41E-05
0.253507526	5.98E-05	0.148048127	0.020435281	-0.369223884	2.50E-09
0.371570619	1.94E-09	0.332771966	9.56E-08	-0.17362087	0.006441444
-0.368366703	2.73E-09	-0.203076378	0.001394644	0.528447835	5.03E-19
-0.376020027	1.20E-09	-0.22225568	0.000456606	0.135722194	0.033723791
0.499838654	6.85E-17	0.448791586	1.52E-13	-0.312250064	6.11E-07
-0.312068376	6.21E-07	-0.305427698	1.10E-06	0.321192158	2.77E-07
0.525238782	8.93E-19	0.461877231	2.38E-14	-0.259405888	3.95E-05
0.608598796	3.22E-26	0.559330046	1.44E-21	-0.522216261	1.53E-18
-0.295507108	2.51E-06	-0.279478785	8.96E-06	0.175694973	0.005824887
-0.548379172	1.23E-20	-0.580063842	1.98E-23	0.354601683	1.14E-08
0.333322046	9.08E-08	0.288623679	4.38E-06	-0.24579572	0.000101306
0.372820448	1.70E-09	0.256329309	4.91E-05	0.062158748	0.332595136
-0.541617537	4.46E-20	-0.404924883	4.40E-11	0.628138549	2.69E-28
0.608083799	3.64E-26	0.581345829	1.51E-23	-0.418997236	7.80E-12
0.569359899	1.88E-22	0.411704176	1.93E-11	-0.383512266	5.25E-10
0.662746991	2.30E-32	0.566989919	3.07E-22	-0.5470383	1.59E-20
0.542113525	4.06E-20	0.416290343	1.09E-11	-0.371968253	1.86E-09
0.124691681	0.05125165	0.17664516	0.005560519	0.045567979	0.477720893
-0.559617281	1.36E-21	-0.512195256	8.68E-18	0.511754402	9.36E-18
-0.502643566	4.32E-17	-0.469415579	7.89E-15	0.663879603	1.66E-32
0.452234926	9.41E-14	0.437112214	7.45E-13	-0.205864666	0.001192813
0.443283605	3.24E-13	0.336965498	6.43E-08	-0.289044432	4.23E-06
0.474265994	3.82E-15	0.44283255	3.45E-13	-0.068199562	0.287658964
-0.480492949	1.48E-15	-0.567117136	2.99E-22	0.241495128	0.00013491
0.557064491	2.26E-21	0.357766377	8.25E-09	-0.255046944	5.37E-05
0.419367705	7.44E-12	0.354224864	1.18E-08	-0.278645755	9.55E-06
-0.456345977	5.26E-14	-0.457429439	4.51E-14	0.299575449	1.79E-06
-0.371458081	1.97E-09	-0.275436423	1.22E-05	0.140949375	0.027387627
-0.46895524	8.44E-15	-0.546234981	1.86E-20	0.20272552	0.001422146
-0.47100079	6.23E-15	-0.579175286	2.40E-23	0.198067445	0.0018375
0.348870032	2.02E-08	0.393687189	1.65E-10	-0.281260777	7.80E-06
-0.580663767	1.74E-23	-0.66671913	7.25E-33	0.262942887	3.07E-05
-0.400676561	7.29E-11	-0.468478268	9.06E-15	0.409219097	2.62E-11
-0.420614547	6.36E-12	-0.570716095	1.42E-22	0.463301827	1.94E-14
-0.660891229	3.93E-32	-0.592616725	1.27E-24	0.482795996	1.04E-15
-0.239524167	0.000153572	-0.114798895	0.072871938	0.254521623	5.58E-05

-0.382648215	5.78E-10	-0.416619138	1.05E-11	0.049527394	0.440271076
-0.480056285	1.58E-15	-0.341674374	4.09E-08	0.567653605	2.68E-22
-0.375777236	1.23E-09	-0.360157699	6.46E-09	0.275849614	1.18E-05
0.343151161	3.55E-08	0.368190378	2.79E-09	-0.064232544	0.316686117
-0.145953039	0.022307356	-0.289022	4.24E-06	0.007283725	0.909692201
0.335062137	7.71E-08	0.24639726	9.73E-05	-0.234438502	0.000213478
-0.295369387	2.54E-06	-0.398033074	9.96E-11	0.273886167	1.37E-05
0.534262121	1.75E-19	0.396490944	1.19E-10	-0.085890847	0.180243196
0.485202709	7.13E-16	0.370071291	2.28E-09	-0.289738315	4.00E-06
-0.513562917	6.87E-18	-0.451068697	1.11E-13	0.339980355	4.82E-08
-0.17894378	0.00496494	-0.270753232	1.73E-05	0.17336362	0.006521848
0.304461216	1.19E-06	0.196788405	0.001969514	-0.271107066	1.69E-05
0.463611752	1.85E-14	0.419973148	6.89E-12	-0.438696751	6.03E-13
0.47286573	4.71E-15	0.406710265	3.55E-11	-0.143646978	0.024537542
0.488130005	4.50E-16	0.373529068	1.57E-09	-0.205499782	0.001217605
0.468742904	8.71E-15	0.475538044	3.15E-15	-0.064672069	0.313379126
-0.5210813	1.86E-18	-0.348275659	2.15E-08	0.579406036	2.28E-23
-0.245910722	0.000100526	-0.229929157	0.000284189	0.002348529	0.970825996
0.301396974	1.54E-06	0.303965156	1.24E-06	-0.131403375	0.039860712
0.422999413	4.70E-12	0.289097346	4.21E-06	-0.234652719	0.000210567
0.622329237	1.16E-27	0.606730938	5.01E-26	-0.12831921	0.044798198
-0.333161096	9.22E-08	-0.348062269	2.19E-08	0.239023669	0.000158682
0.50200609	4.80E-17	0.533626068	1.96E-19	-0.22680434	0.000345384
0.431591707	1.55E-12	0.410529903	2.23E-11	-0.020526603	0.74920885
0.447625153	1.79E-13	0.42589187	3.24E-12	0.046011571	0.473440943
0.238862418	0.000160362	0.274279182	1.33E-05	-0.397796694	1.02E-10
-0.308308372	8.59E-07	-0.382880821	5.64E-10	0.002590607	0.967820385
0.362998803	4.81E-09	0.316534515	4.19E-07	-0.327737163	1.53E-07
0.539669455	6.42E-20	0.40442068	4.67E-11	0.053655984	0.403066877
-0.358926315	7.33E-09	-0.416952557	1.01E-11	0.23035883	0.000276613
0.494429839	1.65E-16	0.569745068	1.74E-22	-0.355814604	1.01E-08
0.392476439	1.90E-10	0.209347702	0.000978427	-0.305683746	1.07E-06
0.452988418	8.46E-14	0.458885046	3.66E-14	-0.305021433	1.14E-06
0.577931875	3.13E-23	0.527406581	6.06E-19	-0.101352123	0.113563775
-0.369683289	2.38E-09	-0.373200774	1.63E-09	0.382165923	6.10E-10
-0.188863242	0.002999256	-0.237547275	0.000174696	0.087253726	0.173403075
-0.396683406	1.17E-10	-0.425776954	3.29E-12	0.093316374	0.145298061
-0.530294501	3.60E-19	-0.444684731	2.68E-13	0.606271107	5.58E-26
0.677281356	3.07E-34	0.504917633	2.96E-17	-0.160174247	0.012054936
0.331023833	1.13E-07	0.284904658	5.87E-06	-0.33310502	9.27E-08

-0.375682198	1.25E-09	-0.521922378	1.61E-18	0.187341128	0.003245646
0.417601296	9.29E-12	0.367983117	2.85E-09	-0.316892963	4.06E-07
-0.495342897	1.42E-16	-0.526365901	7.31E-19	0.221497849	0.000478085
0.416483402	1.07E-11	0.472023919	5.35E-15	-0.314814919	4.88E-07
-0.48740102	5.05E-16	-0.560704247	1.10E-21	0.337043134	6.39E-08
-0.471218462	6.03E-15	-0.514303886	6.05E-18	0.512436562	8.33E-18
0.396017874	1.26E-10	0.487563245	4.92E-16	-0.382049641	6.18E-10
0.329457792	1.30E-07	0.283791747	6.41E-06	-0.000974596	0.987891106
-0.577844774	3.18E-23	-0.565812282	3.90E-22	0.41556497	1.20E-11
0.506251463	2.37E-17	0.394847766	1.44E-10	-0.501978731	4.82E-17
0.378432331	9.22E-10	0.332266599	1.00E-07	-0.200388261	0.001618401
0.410606616	2.21E-11	0.277229193	1.06E-05	-0.382163104	6.11E-10
0.717819183	4.36E-40	0.701578829	1.26E-37	-0.492861676	2.12E-16
-0.336480171	6.74E-08	-0.279302271	9.08E-06	0.463063474	2.00E-14
0.403221354	5.39E-11	0.408877382	2.73E-11	0.081005272	0.206400127
0.660848579	3.97E-32	0.630073707	1.64E-28	-0.119474411	0.061873652
-0.459918905	3.16E-14	-0.596407768	5.42E-25	0.342738535	3.69E-08
0.60127376	1.78E-25	0.476830687	2.59E-15	-0.517372856	3.56E-18
0.625715393	4.96E-28	0.493813475	1.82E-16	-0.36846519	2.71E-09
0.390207185	2.47E-10	0.126950079	0.04714901	-0.482716552	1.05E-15
-0.611219132	1.73E-26	-0.624557378	6.63E-28	0.281608224	7.60E-06
0.631216692	1.23E-28	0.564785036	4.81E-22	-0.044573277	0.487394156
0.476649437	2.66E-15	0.278749648	9.47E-06	-0.380132651	7.65E-10
-0.516497713	4.15E-18	-0.385069095	4.41E-10	0.134482912	0.035396636
-0.572911991	9.01E-23	-0.450721024	1.16E-13	0.317105666	3.99E-07
-0.350285476	1.76E-08	-0.369129051	2.52E-09	0.304987675	1.14E-06
-0.525269339	8.89E-19	-0.607169736	4.52E-26	0.194537487	0.002223025
0.389387171	2.71E-10	0.257107193	4.65E-05	-0.228670427	0.000307512
-0.611542633	1.60E-26	-0.611246408	1.72E-26	0.350221414	1.77E-08
0.494898528	1.53E-16	0.37611027	1.19E-09	-0.29081657	3.67E-06
-0.649039901	1.09E-30	-0.627107121	3.49E-28	0.204347375	0.001299048
0.718482938	3.43E-40	0.482301521	1.12E-15	-0.175191304	0.00596954
-0.550579381	8.06E-21	-0.562254924	8.02E-22	0.369437833	2.44E-09
0.198599421	0.001785012	0.150226111	0.018635033	-0.388094788	3.14E-10
-0.676393959	4.03E-34	-0.666266761	8.28E-33	0.569185322	1.95E-22
0.528335377	5.13E-19	0.690792046	4.42E-36	-0.242273215	0.000128145
-0.277297966	1.06E-05	-0.267604544	2.19E-05	0.280731019	8.13E-06
0.55859532	1.67E-21	0.505467431	2.70E-17	-0.267978572	2.13E-05
0.231964243	0.000249937	0.277129215	1.07E-05	-0.315402588	4.63E-07
-0.484127484	8.43E-16	-0.372132022	1.83E-09	0.529405626	4.23E-19

-0.362959497	4.83E-09	-0.350814905	1.67E-08	0.248268614	8.57E-05
-0.185443707	0.003578388	-0.348927042	2.01E-08	0.184285092	0.003796436
-0.661508181	3.29E-32	-0.503486546	3.76E-17	0.40073036	7.25E-11
0.372513939	1.76E-09	0.239825739	0.000150568	-0.556509489	2.52E-21
0.6068272	4.90E-26	0.446673569	2.04E-13	-0.413677395	1.51E-11
0.311496953	6.52E-07	0.096690177	0.131237673	-0.293317051	3.00E-06
0.427942598	2.49E-12	0.378819747	8.84E-10	-0.218573469	0.000570059
-0.118574173	0.063878021	-0.18905161	0.002969974	0.1023304	0.110103136
0.580054644	1.99E-23	0.632953245	7.85E-29	-0.176110407	0.005707946
-0.277542148	1.04E-05	-0.437317542	7.25E-13	0.212347599	0.000822835
-0.401814588	6.37E-11	-0.461492939	2.52E-14	0.020824236	0.745696461
-0.386202897	3.88E-10	-0.442031416	3.84E-13	0.436002523	8.64E-13
0.631002306	1.30E-28	0.643660903	4.68E-30	-0.335602165	7.32E-08
0.459273923	3.46E-14	0.393755741	1.64E-10	-0.442890936	3.42E-13
0.449461708	1.39E-13	0.342956342	3.62E-08	-0.372468763	1.76E-09
0.633305021	7.16E-29	0.349602424	1.88E-08	-0.633877453	6.18E-29
-0.49902916	7.83E-17	-0.258198611	4.31E-05	0.118038712	0.065095289
-0.170413011	0.007510304	-0.307427312	9.26E-07	0.266474583	2.38E-05
-0.100922202	0.115111292	-0.173196654	0.006574512	0.416722133	1.04E-11
0.413492813	1.55E-11	0.318565552	3.50E-07	-0.394494487	1.50E-10
0.242523219	0.000126039	0.227740273	0.000325878	-0.258041364	4.36E-05
0.439719775	5.25E-13	0.354069751	1.20E-08	-0.376053824	1.20E-09
0.512746482	7.90E-18	0.294465343	2.73E-06	-0.242860191	0.000123253
-0.559004014	1.54E-21	-0.503370861	3.83E-17	0.539098188	7.15E-20
0.112907928	0.077749625	0.439177227	5.65E-13	-0.177290492	0.005387158
0.273403374	1.42E-05	0.237575481	0.000174376	-0.418239804	8.58E-12
0.632530485	8.75E-29	0.419802972	7.04E-12	-0.424110224	4.08E-12
0.47530135	3.27E-15	0.329502707	1.30E-07	-0.474143245	3.89E-15
0.355032955	1.09E-08	0.39180535	2.05E-10	-0.24473397	0.000108782
0.354025134	1.21E-08	0.407261301	3.32E-11	-0.315650559	4.53E-07
0.343262481	3.51E-08	0.343856906	3.31E-08	-0.335285363	7.55E-08
-0.404305984	4.73E-11	-0.29497991	2.62E-06	0.537485347	9.65E-20
-0.43370077	1.17E-12	-0.596046511	5.88E-25	0.229107806	0.000299213
-0.542968633	3.46E-20	-0.626865119	3.71E-28	0.202085103	0.001473629
-0.423078664	4.65E-12	-0.548578176	1.19E-20	0.266726214	2.33E-05
0.344532666	3.10E-08	0.305620149	1.08E-06	-0.320680642	2.90E-07
0.783930767	3.23E-52	0.484977047	7.38E-16	-0.493767372	1.84E-16
-0.380790078	7.11E-10	-0.421076917	6.00E-12	0.358428691	7.71E-09
-0.076729925	0.231448396	-0.165817971	0.009316572	0.069407884	0.279183716
0.573672776	7.68E-23	0.542309242	3.91E-20	-0.070535186	0.2714304

-0.480824228	1.41E-15	-0.487632206	4.87E-16	0.392274254	1.94E-10
-0.375673584	1.25E-09	-0.328547053	1.42E-07	0.451615056	1.03E-13
-0.614449118	7.97E-27	-0.472283832	5.14E-15	0.317465458	3.86E-07
0.540238681	5.77E-20	0.477683607	2.27E-15	-0.450697246	1.17E-13
-0.420127237	6.76E-12	-0.414148661	1.43E-11	0.338702721	5.45E-08
0.51209333	8.83E-18	0.607208654	4.48E-26	-0.247454118	9.06E-05
-0.344801308	3.02E-08	-0.449739935	1.33E-13	0.349105868	1.98E-08
0.406597518	3.59E-11	0.415755735	1.17E-11	-0.54839831	1.23E-20
0.266118255	2.44E-05	0.56725601	2.90E-22	0.099981926	0.118553269
0.467273689	1.08E-14	0.416082184	1.12E-11	-0.591760755	1.54E-24
-0.431031158	1.66E-12	-0.551423262	6.84E-21	0.379794513	7.94E-10
0.253847672	5.84E-05	0.555564872	3.04E-21	0.064058661	0.318000669
0.37587358	1.22E-09	0.349164482	1.96E-08	-0.038963175	0.543863002
-0.444009614	2.94E-13	-0.356884025	9.03E-09	0.048054197	0.454005957
-0.402488311	5.88E-11	-0.429530184	2.03E-12	0.498431451	8.63E-17
-0.570748455	1.41E-22	-0.530529364	3.45E-19	0.428760338	2.24E-12
-0.462268056	2.25E-14	-0.422270551	5.15E-12	0.186414948	0.003404397
-0.169424838	0.00787001	-0.198100097	0.001834238	0.128193719	0.045009496
-0.635051816	4.55E-29	-0.660812896	4.02E-32	0.325825718	1.82E-07
-0.27178201	1.60E-05	-0.396840883	1.15E-10	0.364147507	4.27E-09
-0.573621859	7.76E-23	-0.47069255	6.52E-15	0.444802832	2.63E-13
-0.359603727	6.84E-09	-0.340689974	4.50E-08	0.395446892	1.35E-10
-0.263626972	2.92E-05	-0.444384697	2.79E-13	0.226557338	0.00035071
-0.64467606	3.56E-30	-0.594400145	8.53E-25	0.434024639	1.12E-12
-0.581468375	1.47E-23	-0.53872481	7.66E-20	0.489393596	3.69E-16
0.361865791	5.41E-09	0.642491198	6.40E-30	0.025561376	0.690543388
0.62783662	2.90E-28	0.289828713	3.97E-06	-0.310753133	6.96E-07
0.450490742	1.20E-13	0.350714524	1.68E-08	-0.410984751	2.11E-11
-0.505570084	2.66E-17	-0.471062963	6.17E-15	0.204167964	0.001312167
-0.355263689	1.07E-08	-0.414618502	1.35E-11	0.284715521	5.96E-06
-0.444498249	2.75E-13	-0.491624826	2.59E-16	0.614439931	7.98E-27
-0.482721074	1.05E-15	-0.515145271	5.24E-18	0.307663792	9.08E-07
-0.681510241	8.35E-35	-0.585786196	5.76E-24	0.455086841	6.29E-14
0.52282663	1.37E-18	0.459805344	3.21E-14	-0.204799218	0.001266538
-0.456066085	5.48E-14	-0.522082999	1.56E-18	0.26219198	3.24E-05
-0.501362087	5.34E-17	-0.416456623	1.07E-11	0.296291402	2.35E-06
-0.364308365	4.19E-09	-0.390554464	2.37E-10	0.188528195	0.003051988
0.671604473	1.71E-33	0.432146482	1.44E-12	-0.530751504	3.32E-19
0.474810099	3.52E-15	0.740368514	8.32E-44	-0.426452023	3.02E-12
-0.555301028	3.20E-21	-0.51500305	5.37E-18	0.368391882	2.73E-09

0.415539453	1.20E-11	0.350362468	1.74E-08	-0.249409516	7.93E-05
-0.040811036	0.524912079	-0.395592606	1.32E-10	0.294125857	2.81E-06
-0.392511063	1.89E-10	-0.307732866	9.02E-07	0.32533909	1.90E-07
-0.33488671	7.84E-08	-0.391576919	2.11E-10	0.269599116	1.89E-05
0.449583382	1.36E-13	0.48967522	3.53E-16	-0.042239264	0.5104985
-0.183054096	0.004041188	-0.253675609	5.91E-05	0.320545124	2.93E-07
-0.483752736	8.93E-16	-0.460923721	2.73E-14	0.175472653	0.005888345
-0.50284524	4.18E-17	-0.440202869	4.92E-13	0.499444858	7.31E-17
0.367538996	2.99E-09	0.331087088	1.12E-07	-0.150170626	0.018679124
0.488424847	4.30E-16	0.405328999	4.19E-11	-0.384573847	4.66E-10
-0.567510419	2.76E-22	-0.283829697	6.39E-06	0.23617773	0.00019089
0.474269336	3.82E-15	0.550804751	7.71E-21	-0.075805665	0.237134263
-0.279406941	9.00E-06	-0.411092342	2.08E-11	0.3083375	8.57E-07
0.462939064	2.04E-14	0.391122631	2.22E-10	0.063404791	0.322975684
0.301752054	1.50E-06	0.142254531	0.025975087	-0.350128378	1.79E-08
0.492078307	2.41E-16	0.755027864	1.94E-46	-0.377359811	1.04E-09
-0.220392943	0.000511085	-0.303752636	1.27E-06	0.169638026	0.007791135
0.429434551	2.05E-12	0.347829479	2.24E-08	-0.301366934	1.55E-06
0.307531571	9.18E-07	0.307564998	9.15E-07	0.336298267	6.85E-08
0.531625546	2.83E-19	0.450548004	1.19E-13	-0.407505106	3.22E-11
0.569909207	1.68E-22	0.46425717	1.68E-14	-0.386672743	3.68E-10
-0.263735104	2.90E-05	-0.405209941	4.25E-11	0.08363653	0.191991295
-0.303057659	1.34E-06	-0.406235213	3.75E-11	0.316400125	4.24E-07
0.008948152	0.889175763	-0.196380471	0.002013404	-0.060402609	0.346461378
-0.655305206	1.91E-31	-0.607258165	4.42E-26	0.478066614	2.15E-15
-0.567012995	3.05E-22	-0.545488629	2.14E-20	0.41140038	2.00E-11
0.422370139	5.09E-12	0.443489918	3.15E-13	-0.192446671	0.002484767
-0.509610502	1.35E-17	-0.352382028	1.42E-08	0.567794977	2.60E-22
0.425306218	3.50E-12	0.45846009	3.89E-14	-0.256347434	4.91E-05
0.231903414	0.000250902	0.177483211	0.005336335	-0.293506473	2.95E-06
-0.360567832	6.19E-09	-0.171100456	0.007268783	0.324885168	1.98E-07
-0.416343271	1.09E-11	-0.470387414	6.82E-15	0.114387422	0.073911569
-0.12972966	0.042479987	-0.273151587	1.45E-05	0.158797299	0.012822161
-0.379297726	8.39E-10	-0.264907044	2.66E-05	0.548581669	1.19E-20
-0.269791232	1.86E-05	-0.341769753	4.06E-08	0.339253969	5.17E-08
-0.215922728	0.000667312	-0.265815574	2.49E-05	0.124479178	0.051652526
-0.097492574	0.128053293	-0.064515988	0.314550885	0.347524198	2.31E-08
-0.126635329	0.047703776	-0.296779474	2.26E-06	-0.151980112	0.017287082
-0.166755148	0.008919654	-0.280995333	7.97E-06	0.253393563	6.03E-05
-0.630963953	1.31E-28	-0.606281357	5.56E-26	0.454198679	7.13E-14

0.612044851	1.42E-26	0.401534147	6.59E-11	-0.10748805	0.093207236
0.462693804	2.11E-14	0.338860611	5.37E-08	-0.259386602	3.96E-05
0.378565168	9.09E-10	0.178909053	0.004973494	-0.19715305	0.001931023
-0.333044845	9.32E-08	-0.335012106	7.74E-08	0.057684595	0.368632375
0.521247494	1.81E-18	0.236784851	0.000183545	-0.158146769	0.013199337
0.583407168	9.66E-24	0.474878675	3.48E-15	0.003172088	0.960602734
0.616300991	5.09E-27	0.436147805	8.47E-13	-0.276542355	1.12E-05
0.631012404	1.29E-28	0.373585685	1.56E-09	-0.399687309	8.20E-11
0.624539135	6.66E-28	0.403469776	5.23E-11	-0.309174713	7.97E-07
0.589726323	2.42E-24	0.303357631	1.31E-06	-0.235258132	0.000202539
0.576029708	4.68E-23	0.287808817	4.67E-06	-0.271682248	1.62E-05
0.629339916	1.98E-28	0.290574691	3.74E-06	-0.311968308	6.26E-07
0.483969232	8.64E-16	0.269762201	1.86E-05	-0.218133128	0.000585248
0.62444311	6.83E-28	0.435875561	8.79E-13	-0.133205342	0.037194302
-0.402515463	5.86E-11	-0.388043383	3.15E-10	0.389796617	2.58E-10
0.513077356	7.47E-18	0.394261872	1.55E-10	-0.443355851	3.21E-13
-0.280865514	8.05E-06	-0.407577106	3.19E-11	0.345398873	2.85E-08
0.371604153	1.94E-09	0.30862891	8.35E-07	-0.304092312	1.23E-06
0.378143842	9.52E-10	0.259263221	3.99E-05	-0.233038229	0.000233451
0.639376572	1.47E-29	0.614171	8.52E-27	-0.33889636	5.35E-08
0.455469222	5.96E-14	0.733578112	1.20E-42	-0.457606563	4.40E-14
-0.553177302	4.86E-21	-0.524672251	9.88E-19	0.552471239	5.58E-21
0.696221883	7.53E-37	0.499782	6.92E-17	-0.535627937	1.36E-19
-0.546344256	1.82E-20	-0.558236617	1.79E-21	0.403657099	5.12E-11
-0.579730536	2.13E-23	-0.556543845	2.51E-21	0.425702488	3.32E-12
-0.642789716	5.91E-30	-0.541310669	4.72E-20	0.609674441	2.50E-26
-0.601237433	1.80E-25	-0.53304573	2.18E-19	0.422754422	4.85E-12
-0.34784827	2.24E-08	-0.365408888	3.74E-09	-0.017353116	0.786967536
0.530598848	3.41E-19	0.317987105	3.69E-07	-0.322509269	2.46E-07
-0.32152537	2.69E-07	-0.377686007	1.00E-09	0.343292389	3.50E-08
-0.557640398	2.02E-21	-0.571775916	1.14E-22	0.359262475	7.08E-09
0.307978073	8.83E-07	0.333037267	9.33E-08	-0.308623942	8.36E-07
-0.152884608	0.016625791	-0.19273666	0.002446864	0.287566382	4.76E-06
0.326694632	1.68E-07	0.441997622	3.86E-13	-0.26383188	2.88E-05
-0.408723145	2.78E-11	-0.558384279	1.74E-21	0.32651763	1.71E-07
-0.647891054	1.49E-30	-0.458359676	3.95E-14	0.377005992	1.08E-09
0.405650974	4.03E-11	0.720818745	1.47E-40	-0.477507752	2.34E-15
-0.468512488	9.01E-15	-0.464837229	1.55E-14	0.287404387	4.82E-06
-0.541638476	4.44E-20	-0.552997892	5.03E-21	0.415660523	1.18E-11
-0.413417291	1.56E-11	-0.464096423	1.72E-14	0.365352084	3.76E-09

-0.255581761	5.18E-05	-0.206386605	0.001158153	0.340915247	4.41E-08
-0.712064413	3.40E-39	-0.536145412	1.24E-19	0.489826216	3.44E-16
-0.623504462	8.63E-28	-0.563736323	5.95E-22	0.61305616	1.11E-26
-0.194565748	0.002219666	-0.42168398	5.55E-12	0.292541103	3.19E-06
-0.241018379	0.000139219	-0.418862066	7.93E-12	0.201338566	0.001535795
-0.323441546	2.26E-07	-0.452616099	8.92E-14	0.298831382	1.91E-06
-0.042280279	0.510087644	-0.079391051	0.215617118	0.020018005	0.755222785
-0.422006538	5.33E-12	-0.342103141	3.93E-08	0.387692493	3.28E-10
-0.509263763	1.43E-17	-0.515311787	5.09E-18	0.392288657	1.94E-10
-0.278917257	9.35E-06	-0.425293575	3.50E-12	0.192735389	0.002447028
-0.400474415	7.47E-11	-0.477663088	2.28E-15	0.252857462	6.26E-05
-0.220627143	0.000503919	-0.299883412	1.75E-06	0.269584792	1.89E-05
-0.322185927	2.53E-07	-0.296075767	2.40E-06	0.315004591	4.80E-07
-0.632396183	9.06E-29	-0.584262934	8.02E-24	0.42960241	2.01E-12
0.621161198	1.54E-27	0.589389972	2.61E-24	-0.327874671	1.51E-07
-0.47545632	3.19E-15	-0.540562963	5.43E-20	0.246097034	9.93E-05
-0.309367057	7.84E-07	-0.294101146	2.82E-06	0.455694824	5.77E-14
-0.456931567	4.84E-14	-0.412096562	1.84E-11	0.200091293	0.001645037
-0.560075579	1.24E-21	-0.507647711	1.88E-17	0.496816441	1.12E-16
0.632048523	9.91E-29	0.34397116	3.28E-08	-0.342327544	3.84E-08
-0.459849187	3.19E-14	-0.465628844	1.38E-14	0.467834988	9.96E-15
-0.539952625	6.09E-20	-0.511895467	9.14E-18	0.471810702	5.52E-15
0.349791566	1.85E-08	0.477328035	2.40E-15	-0.020505365	0.749459681
0.329124687	1.34E-07	0.39389758	1.61E-10	-0.425887492	3.25E-12
-0.418195136	8.62E-12	-0.517802482	3.31E-18	0.2838227	6.39E-06
0.414562966	1.36E-11	0.435459496	9.29E-13	-0.220244002	0.000515692
-0.512233719	8.63E-18	-0.470607958	6.60E-15	0.412693395	1.71E-11
0.386388599	3.80E-10	0.415361617	1.23E-11	-0.443713028	3.06E-13
0.655479633	1.82E-31	0.396796277	1.15E-10	-0.300141788	1.71E-06
0.572552839	9.71E-23	0.277046066	1.08E-05	-0.220469344	0.000508737
0.585417428	6.24E-24	0.53079495	3.29E-19	-0.50192445	4.86E-17
0.561316222	9.69E-22	0.501993317	4.81E-17	-0.345095428	2.94E-08
0.503761291	3.59E-17	0.31908894	3.34E-07	-0.371915517	1.87E-09
-0.40042973	7.51E-11	-0.426766292	2.90E-12	0.399267637	8.61E-11
-0.35160414	1.54E-08	-0.356969225	8.96E-09	0.239079591	0.000158103
0.605564212	6.58E-26	0.486982124	5.39E-16	-0.242511657	0.000126136
0.630573193	1.45E-28	0.506933533	2.11E-17	-0.641835347	7.63E-30
-0.501123084	5.55E-17	-0.537798255	9.10E-20	0.242376012	0.000127275
-0.441803539	3.96E-13	-0.433931551	1.14E-12	0.18761615	0.003199814
-0.382818174	5.68E-10	-0.394458486	1.51E-10	0.190812674	0.002708495

-0.551873195	6.27E-21	-0.412811126	1.68E-11	0.666319314	8.15E-33
0.386471666	3.77E-10	0.450290911	1.23E-13	-0.13944718	0.029094469
0.481799274	1.21E-15	0.439779699	5.21E-13	0.240508757	0.000143967
-0.002421347	0.969921842	-0.280353392	8.37E-06	-0.037540507	0.55868016
-0.693119292	2.08E-36	-0.621145045	1.55E-27	0.40214014	6.13E-11
-0.461489515	2.52E-14	-0.378458205	9.20E-10	0.49825668	8.88E-17
-0.196602728	0.001989382	-0.42837633	2.35E-12	0.371750242	1.91E-09
0.436940031	7.62E-13	0.420419443	6.52E-12	-0.36209669	5.28E-09
0.454444914	6.89E-14	0.221727547	0.000471478	-0.173985716	0.006328931
0.404696666	4.52E-11	0.488844799	4.02E-16	-0.169646809	0.007787901
-0.456854854	4.90E-14	-0.321936507	2.59E-07	0.08445872	0.187643373
0.603673848	1.02E-25	0.342982244	3.61E-08	-0.173580414	0.00645403
-0.497459299	1.01E-16	-0.491658084	2.57E-16	0.420572036	6.39E-12
-0.387525577	3.34E-10	-0.335927738	7.10E-08	0.368673025	2.65E-09
-0.441599718	4.08E-13	-0.329285777	1.32E-07	0.505537682	2.67E-17
-0.218516661	0.000571998	-0.036711646	0.567401719	0.346487751	2.56E-08
0.590756659	1.93E-24	0.404947409	4.38E-11	-0.106879292	0.095086772
-0.092346926	0.149543129	-0.289611361	4.04E-06	0.0180849	0.778212781
-0.648963656	1.11E-30	-0.569024328	2.02E-22	0.294904572	2.64E-06
-0.606382209	5.43E-26	-0.716795182	6.31E-40	0.264514369	2.74E-05
-0.621950707	1.27E-27	-0.542506174	3.77E-20	0.040298406	0.530135548
-0.599897339	2.45E-25	-0.545814491	2.01E-20	0.467561221	1.04E-14
0.368220254	2.78E-09	0.387202719	3.47E-10	-0.165675494	0.009378267
-0.516571192	4.09E-18	-0.432152564	1.44E-12	0.575972036	4.74E-23
-0.377397209	1.03E-09	-0.413752663	1.50E-11	0.272681374	1.50E-05
-0.483152457	9.81E-16	-0.358507783	7.65E-09	0.309479223	7.76E-07
-0.195692761	0.00208942	-0.242718174	0.00012442	0.098040785	0.125912234
-0.572578587	9.65E-23	-0.596593978	5.20E-25	0.401062631	6.97E-11
-0.52232561	1.50E-18	-0.560059086	1.25E-21	0.198190967	0.001825188
-0.124236815	0.052112913	-0.287454841	4.80E-06	0.100488054	0.116690703
0.61344355	1.02E-26	0.456938568	4.84E-14	-0.274801773	1.28E-05
0.431472509	1.57E-12	0.341825065	4.04E-08	-0.350164165	1.78E-08
-0.283037934	6.80E-06	-0.329146785	1.34E-07	0.297481515	2.13E-06
-0.197898429	0.001854468	-0.225282019	0.000379444	0.186006935	0.003476535
-0.311035238	6.79E-07	-0.422897852	4.76E-12	0.196783941	0.00196999
-0.429502504	2.03E-12	-0.425000659	3.64E-12	0.213449427	0.000771665
-0.396751223	1.16E-10	-0.481373552	1.29E-15	0.58789613	3.62E-24
-0.578857227	2.57E-23	-0.572890406	9.05E-23	0.254767615	5.48E-05
-0.162989774	0.010610963	-0.306979374	9.62E-07	0.019676746	0.759266321
0.531467266	2.91E-19	0.651023546	6.30E-31	-0.134424969	0.035476543

-0.467446823	1.06E-14	-0.458226853	4.02E-14	0.425050542	3.61E-12
-0.521702373	1.67E-18	-0.4970415	1.08E-16	0.413040523	1.64E-11
-0.487884929	4.68E-16	-0.462219432	2.27E-14	0.34458668	3.08E-08
-0.467804518	1.00E-14	-0.480247274	1.54E-15	0.251839586	6.71E-05
-0.296950708	2.23E-06	-0.212560877	0.000812692	0.239727441	0.000151541
-0.461616466	2.47E-14	-0.444297254	2.82E-13	0.262112139	3.26E-05
-0.322799152	2.40E-07	-0.292531264	3.20E-06	0.232019923	0.000249056
0.329862239	1.25E-07	0.273973848	1.36E-05	-0.153057728	0.016501769
-0.416039849	1.13E-11	-0.512327578	8.49E-18	0.1959389	0.002061917
-0.536624359	1.13E-19	-0.545710608	2.05E-20	0.280499082	8.28E-06
-0.36562326	3.65E-09	-0.46620868	1.27E-14	0.305003881	1.14E-06
0.513981998	6.40E-18	0.684477425	3.30E-35	-0.090541215	0.157699819
-0.378022174	9.65E-10	-0.430470289	1.79E-12	0.264357666	2.77E-05
-0.647722099	1.56E-30	-0.565484643	4.17E-22	0.529985617	3.81E-19
-0.438616484	6.09E-13	-0.522968596	1.34E-18	0.245732184	0.00010174
-0.111363825	0.081925712	-0.187660024	0.003192557	-0.124209574	0.052164873
0.534800979	1.58E-19	0.465162798	1.48E-14	-0.566831831	3.17E-22
0.536535493	1.15E-19	0.412537168	1.74E-11	-0.421684549	5.55E-12
0.560414001	1.16E-21	0.521603629	1.70E-18	-0.42490685	3.68E-12
0.648122218	1.40E-30	0.437504217	7.07E-13	-0.273881744	1.37E-05
0.42068542	6.30E-12	0.368360959	2.74E-09	-0.00644568	0.920045882
0.470574669	6.64E-15	0.098017266	0.126003513	-0.526612726	6.99E-19
0.574981019	5.84E-23	0.39441266	1.52E-10	0.038966553	0.543828049
0.459381476	3.41E-14	0.487603605	4.89E-16	-0.217079462	0.000623127
-0.435408121	9.35E-13	-0.454025754	7.31E-14	0.228661746	0.000307679
-0.578391774	2.84E-23	-0.550719946	7.84E-21	0.368200689	2.78E-09
-0.558151102	1.82E-21	-0.507798791	1.83E-17	0.317071018	4.00E-07
-0.423279554	4.53E-12	-0.312519376	5.97E-07	0.300552775	1.65E-06
-0.507823932	1.82E-17	-0.410906644	2.13E-11	0.374857578	1.36E-09
-0.456167317	5.40E-14	-0.489057508	3.89E-16	0.10282398	0.108388818
0.404870086	4.42E-11	0.276094783	1.16E-05	-0.354543862	1.15E-08
-0.616351888	5.02E-27	-0.424097396	4.08E-12	0.283196923	6.71E-06
-0.296944089	2.23E-06	-0.276894181	1.09E-05	0.199524171	0.001697019
0.548361288	1.24E-20	0.375241757	1.31E-09	-0.365890361	3.55E-09
0.602981907	1.20E-25	0.577448222	3.46E-23	-0.401558987	6.57E-11
0.780053526	2.16E-51	0.629219358	2.04E-28	-0.349368375	1.93E-08
-0.361507198	5.62E-09	-0.350919808	1.65E-08	0.361106619	5.85E-09
-0.448459288	1.59E-13	-0.549893374	9.20E-21	0.353659169	1.25E-08
-0.155866293	0.014599638	-0.377335587	1.04E-09	0.334804733	7.90E-08
0.276901121	1.09E-05	0.186865127	0.003326378	-0.341308427	4.24E-08

0.396817185	1.15E-10	0.350199104	1.77E-08	-0.025661623	0.689392505
0.465649282	1.37E-14	0.4756095	3.12E-15	-0.482298488	1.12E-15
-0.345957678	2.70E-08	-0.591185174	1.75E-24	0.044895372	0.484250395
-0.611101472	1.78E-26	-0.559156077	1.49E-21	0.606749491	4.99E-26
-0.368374707	2.73E-09	-0.414766332	1.32E-11	0.545295217	2.22E-20
-0.576293978	4.42E-23	-0.414581412	1.35E-11	0.377961767	9.71E-10
-0.646427574	2.22E-30	-0.585690525	5.88E-24	0.313196523	5.62E-07
0.525474669	8.57E-19	0.628419892	2.50E-28	-0.359755806	6.73E-09
0.429382963	2.06E-12	0.432711132	1.34E-12	-0.257365484	4.57E-05
0.530315854	3.59E-19	0.576945959	3.85E-23	-0.387591684	3.32E-10
0.417223723	9.74E-12	0.30301623	1.35E-06	-0.032240275	0.615533919
0.301952393	1.47E-06	0.492023674	2.43E-16	-0.364209251	4.24E-09
-0.190139984	0.002805802	-0.348943477	2.01E-08	0.383061282	5.52E-10
0.396506896	1.19E-10	0.374157475	1.47E-09	-0.266608288	2.35E-05
0.344473433	3.12E-08	0.395575223	1.33E-10	-0.256333378	4.91E-05
-0.3755979	1.26E-09	-0.38299864	5.56E-10	0.37159773	1.94E-09
0.228629065	0.000308308	0.178831396	0.00499267	-0.140483066	0.027908029
-0.47008719	7.14E-15	-0.367268992	3.07E-09	0.414374606	1.39E-11
-0.659667837	5.57E-32	-0.571976702	1.09E-22	0.370938118	2.08E-09
-0.432711244	1.34E-12	-0.349566972	1.89E-08	0.320914591	2.84E-07
-0.184641666	0.003728084	-0.378556384	9.10E-10	0.220093066	0.000520399
0.491305627	2.72E-16	0.395276788	1.37E-10	-0.383661094	5.17E-10
0.344935527	2.98E-08	0.310885466	6.88E-07	-0.207757457	0.00107148
0.485118185	7.22E-16	0.381727199	6.41E-10	-0.065380692	0.308095184
0.366795894	3.23E-09	0.353376315	1.29E-08	-0.144496269	0.023694812
0.649910167	8.56E-31	0.420118235	6.77E-12	-0.389702875	2.61E-10
0.548067102	1.31E-20	0.41714933	9.83E-12	-0.523242398	1.27E-18
0.509464821	1.38E-17	0.427643699	2.59E-12	-0.314958395	4.82E-07
-0.641564683	8.20E-30	-0.597790488	3.96E-25	0.398725344	9.18E-11
0.607820841	3.87E-26	0.409065233	2.66E-11	-0.383253647	5.41E-10
0.349915374	1.82E-08	0.59187686	1.50E-24	0.293453733	2.97E-06
0.276734055	1.10E-05	0.319052407	3.35E-07	-0.396675526	1.17E-10
0.708230093	1.30E-38	0.481645878	1.24E-15	-0.228751766	0.000305953
-0.541693673	4.40E-20	-0.450870015	1.14E-13	0.259552962	3.91E-05
0.577247604	3.62E-23	0.457343072	4.57E-14	-0.568858455	2.09E-22
0.507158608	2.04E-17	0.405195619	4.26E-11	-0.330297616	1.20E-07
0.308788412	8.24E-07	0.339709606	4.95E-08	-0.304923642	1.15E-06
-0.316973679	4.03E-07	-0.414601983	1.35E-11	0.045299873	0.480317851
-0.503905542	3.50E-17	-0.600764112	2.00E-25	0.386296063	3.84E-10
-0.468059483	9.64E-15	-0.474196734	3.86E-15	0.400918613	7.09E-11

0.395213125	1.38E-10	0.507274233	2.00E-17	-0.347922926	2.22E-08
0.578620303	2.70E-23	0.532318836	2.49E-19	-0.527707904	5.75E-19
-0.320637851	2.91E-07	-0.206143696	0.001174167	0.179198682	0.004902559
0.58071976	1.72E-23	0.408579307	2.83E-11	-0.399434201	8.45E-11
-0.44608428	2.21E-13	-0.3136157	5.42E-07	0.524281266	1.06E-18
0.508731793	1.56E-17	0.463536774	1.87E-14	-0.353606114	1.26E-08
-0.434639526	1.04E-12	-0.4335867	1.19E-12	0.281324669	7.77E-06
-0.434560434	1.05E-12	-0.353136757	1.32E-08	0.083058595	0.195091308
0.614103522	8.66E-27	0.584282616	7.99E-24	-0.484133393	8.42E-16
-0.325289525	1.91E-07	-0.316622693	4.16E-07	0.130077473	0.041924082
-0.572434901	9.95E-23	-0.363680641	4.48E-09	0.171591703	0.00710046
-0.323377469	2.27E-07	-0.335320453	7.52E-08	0.176281475	0.005660407
0.453755018	7.60E-14	0.321829554	2.61E-07	-0.02726093	0.671129893
-0.521897771	1.61E-18	-0.504475912	3.19E-17	0.245893433	0.000100643
-0.356561554	9.34E-09	-0.359222056	7.11E-09	0.310922197	6.85E-07
0.394673015	1.47E-10	0.27602344	1.17E-05	-0.296563089	2.30E-06
-0.545920034	1.97E-20	-0.511130594	1.04E-17	0.202724821	0.001422201
-0.534010903	1.83E-19	-0.587953041	3.58E-24	0.192042826	0.002538441
-0.626396798	4.18E-28	-0.56024316	1.20E-21	0.433595508	1.19E-12
-0.485786077	6.51E-16	-0.447166596	1.90E-13	0.182331492	0.004191404
-0.266575712	2.36E-05	-0.487442259	5.02E-16	0.256599096	4.82E-05
0.033495676	0.601840615	-0.156136067	0.014427466	0.149091596	0.019554795
-0.372885129	1.69E-09	-0.269701141	1.87E-05	0.205632178	0.001208555
-0.061353672	0.338907096	-0.346213434	2.63E-08	0.174465286	0.006183714
-0.455405553	6.01E-14	-0.46082352	2.77E-14	0.259085015	4.05E-05
-0.494477128	1.64E-16	-0.505144634	2.85E-17	0.32224096	2.52E-07
0.441632848	4.06E-13	0.65218625	4.57E-31	0.066334441	0.301076432
-0.495246444	1.45E-16	-0.404519123	4.61E-11	0.263293286	2.99E-05
-0.596817892	4.94E-25	-0.594049229	9.23E-25	0.441693754	4.02E-13
-0.547493349	1.46E-20	-0.541448163	4.60E-20	0.227390059	0.000333054
0.639698273	1.35E-29	0.505234052	2.81E-17	-0.435680911	9.02E-13
-0.300044259	1.73E-06	-0.350902374	1.65E-08	0.147788987	0.0206592
-0.295073978	2.60E-06	-0.207604354	0.001080855	0.255490227	5.21E-05
-0.275277369	1.23E-05	-0.331123616	1.12E-07	0.394896985	1.44E-10
-0.623059345	9.64E-28	-0.503550063	3.72E-17	0.467720618	1.01E-14
-0.347493947	2.32E-08	-0.283452897	6.58E-06	0.144248644	0.023937907
-0.415725406	1.17E-11	-0.46227424	2.25E-14	0.265025459	2.64E-05
-0.331428884	1.08E-07	-0.412185529	1.82E-11	0.275117142	1.25E-05
-0.241122035	0.000138271	-0.242092963	0.000129683	0.351489279	1.56E-08
-0.447368052	1.85E-13	-0.198269404	0.001817409	0.612740671	1.20E-26

-0.32878307	1.39E-07	-0.322831367	2.39E-07	0.238372514	0.000165568
-0.481646852	1.24E-15	-0.379021248	8.64E-10	0.468054473	9.65E-15
-0.276358038	1.14E-05	-0.355959359	9.93E-09	0.333946987	8.56E-08
-0.490518247	3.08E-16	-0.511964887	9.03E-18	0.391866744	2.04E-10
-0.195371051	0.00212587	-0.319617147	3.19E-07	0.242273835	0.000128139
-0.528133965	5.32E-19	-0.54696474	1.62E-20	0.280390017	8.35E-06
0.65876444	7.20E-32	0.605998499	5.95E-26	-0.490114515	3.29E-16
-0.311719788	6.40E-07	-0.335147687	7.64E-08	0.554043204	4.10E-21
0.369666	2.38E-09	0.540909343	5.09E-20	0.012449743	0.846269653
0.58600817	5.48E-24	0.498203113	8.95E-17	-0.471926691	5.42E-15
-0.44666053	2.04E-13	-0.492406535	2.28E-16	0.289546987	4.06E-06
-0.39429951	1.54E-10	-0.397578427	1.05E-10	0.300017658	1.73E-06
-0.356833378	9.08E-09	-0.408429034	2.88E-11	0.700806564	1.64E-37
-0.246212355	9.85E-05	-0.231487311	0.0002576	0.441599477	4.08E-13
-0.497475953	1.01E-16	-0.428176179	2.41E-12	0.186401293	0.003406789
-0.281928411	7.41E-06	-0.309636222	7.66E-07	0.01135106	0.859689637
-0.508555588	1.61E-17	-0.591799784	1.53E-24	0.193834633	0.00230807
0.261278744	3.46E-05	0.212248196	0.000827602	-0.408503389	2.85E-11
0.422065737	5.29E-12	0.249851845	7.70E-05	0.002738316	0.965986668
0.673422447	9.90E-34	0.665933595	9.12E-33	-0.236054229	0.000192417
-0.442117412	3.80E-13	-0.438274221	6.38E-13	0.40001459	7.89E-11
-0.386059254	3.95E-10	-0.412722693	1.70E-11	0.375670668	1.25E-09
-0.474375647	3.76E-15	-0.492087933	2.40E-16	0.158032902	0.01326635
-0.452457569	9.12E-14	-0.483940245	8.68E-16	0.468721485	8.74E-15
-0.281994842	7.37E-06	-0.421193989	5.91E-12	0.241825109	0.000132001
-0.375918365	1.21E-09	-0.461085513	2.67E-14	0.199326416	0.001715496
-0.5698689	1.70E-22	-0.557833112	1.94E-21	0.475761621	3.05E-15
-0.353919347	1.22E-08	-0.419988555	6.88E-12	0.365373075	3.75E-09
-0.372703515	1.72E-09	-0.408557819	2.83E-11	0.242924534	0.000122728
-0.458746789	3.74E-14	-0.500841264	5.81E-17	0.253694465	5.91E-05
0.5005808	6.07E-17	0.569322267	1.90E-22	-0.302667433	1.39E-06
-0.242411818	0.000126974	-0.337003331	6.41E-08	0.276351679	1.14E-05
0.423091105	4.64E-12	0.567920306	2.53E-22	-0.1800344	0.004702947
-0.50650778	2.27E-17	-0.491020828	2.85E-16	0.421339769	5.80E-12
-0.674822666	6.49E-34	-0.394515512	1.50E-10	0.563474469	6.27E-22
-0.297047438	2.21E-06	-0.361369969	5.70E-09	0.255085373	5.36E-05
0.481125691	1.34E-15	0.250794511	7.21E-05	-0.271451128	1.64E-05
-0.348433706	2.11E-08	-0.489232687	3.78E-16	0.235420905	0.00020043
0.558238294	1.79E-21	0.533092698	2.17E-19	-0.090442766	0.158153976
-0.542061827	4.10E-20	-0.578016458	3.07E-23	0.049744674	0.438265575

0.342802087	3.67E-08	0.312226954	6.12E-07	-0.496762428	1.13E-16
-0.030332923	0.636594201	-0.211327287	0.000872992	0.315574155	4.57E-07
-0.500267429	6.39E-17	-0.299092603	1.87E-06	0.387304476	3.43E-10
-0.457199927	4.66E-14	-0.35181934	1.51E-08	0.058946629	0.358230899
-0.607513091	4.17E-26	-0.533614329	1.97E-19	0.296442933	2.32E-06
0.628574516	2.41E-28	0.632591125	8.61E-29	-0.398070669	9.92E-11
0.402881745	5.61E-11	0.439016704	5.77E-13	-0.20552088	0.001216159
0.546790139	1.67E-20	0.552260798	5.81E-21	-0.326336485	1.74E-07
-0.515767677	4.70E-18	-0.479860613	1.63E-15	0.221457271	0.000479262
0.645432645	2.90E-30	0.522442504	1.47E-18	-0.551003491	7.42E-21
0.490639597	3.03E-16	0.270196935	1.81E-05	-0.414004027	1.45E-11
-0.282574526	7.05E-06	-0.422824176	4.80E-12	0.440414847	4.78E-13
0.591624634	1.59E-24	0.649113994	1.07E-30	-0.472277972	5.15E-15
-0.621948169	1.27E-27	-0.527881953	5.57E-19	0.488335887	4.36E-16
0.413518356	1.54E-11	0.420508921	6.44E-12	-0.340701585	4.50E-08
-0.47833883	2.06E-15	-0.416745459	1.03E-11	0.485901429	6.39E-16
-0.422064466	5.29E-12	-0.390906952	2.28E-10	0.289221142	4.17E-06
0.375966529	1.21E-09	0.228904707	0.000303041	-0.419686329	7.15E-12
-0.525716107	8.21E-19	-0.486308393	6.00E-16	0.357592508	8.40E-09
0.455390184	6.03E-14	0.393327039	1.72E-10	-0.278803011	9.43E-06
-0.359090247	7.21E-09	-0.418055957	8.78E-12	0.274810015	1.28E-05
0.526518375	7.11E-19	0.368431865	2.72E-09	-0.199817212	0.001669974
0.542311609	3.91E-20	0.414137857	1.43E-11	-0.312667033	5.89E-07
0.503649731	3.66E-17	0.470236979	6.98E-15	0.096726288	0.131093065
0.44594785	2.25E-13	0.156082118	0.014461754	-0.166411809	0.00906329
-0.234638041	0.000210765	-0.246069267	9.95E-05	0.142477049	0.025740615
-0.527587106	5.87E-19	-0.496922911	1.10E-16	0.473081611	4.56E-15
-0.59058786	2.00E-24	-0.568794136	2.12E-22	0.479424399	1.74E-15
0.692722557	2.37E-36	0.514611405	5.74E-18	-0.572778571	9.26E-23
0.55088053	7.60E-21	0.457319694	4.58E-14	-0.312004548	6.24E-07
0.52145937	1.74E-18	0.480989368	1.37E-15	-0.165919538	0.009272812
-0.307104466	9.52E-07	-0.391238117	2.19E-10	0.423017971	4.69E-12
-0.324323761	2.09E-07	-0.357298218	8.66E-09	0.42752329	2.63E-12
0.244424283	0.000111059	0.238289354	0.000166467	-0.156495007	0.014201148
-0.374357268	1.44E-09	-0.398922401	8.97E-11	0.185150147	0.003632537
-0.538932661	7.37E-20	-0.462583052	2.15E-14	0.188182121	0.003107336
0.446537539	2.08E-13	0.440971566	4.44E-13	0.067573801	0.292115197
0.516191234	4.37E-18	0.343322539	3.49E-08	-0.359823492	6.68E-09
0.483555972	9.21E-16	0.299147608	1.86E-06	-0.351198555	1.60E-08
-0.530567312	3.43E-19	-0.556265796	2.65E-21	0.366473667	3.34E-09

-0.628427314	2.50E-28	-0.558411294	1.73E-21	0.606241668	5.62E-26
-0.574945797	5.88E-23	-0.516278909	4.31E-18	0.441721433	4.01E-13
0.495304215	1.43E-16	0.536180591	1.23E-19	-0.074236391	0.24701142
-0.282995654	6.82E-06	-0.275202192	1.24E-05	0.193422282	0.002359332
-0.262290605	3.22E-05	-0.199704764	0.001680305	0.205667136	0.001206176
-0.549745405	9.47E-21	-0.506085937	2.44E-17	0.465901918	1.32E-14
0.598500836	3.37E-25	0.445544413	2.38E-13	-0.50846725	1.63E-17
-0.305389212	1.10E-06	-0.272192326	1.56E-05	0.252292214	6.51E-05
0.24039359	0.000145061	0.411618879	1.95E-11	-0.376939495	1.09E-09
-0.566048073	3.72E-22	-0.409459353	2.54E-11	0.646831386	1.99E-30
-0.284145103	6.23E-06	-0.30996973	7.44E-07	0.117411897	0.066544321
-0.446702347	2.03E-13	-0.449041395	1.47E-13	0.325466036	1.88E-07
-0.653901411	2.83E-31	-0.559248186	1.47E-21	0.544294307	2.69E-20
-0.450724098	1.16E-13	-0.386121774	3.92E-10	0.455099674	6.28E-14
-0.580715936	1.73E-23	-0.477425742	2.37E-15	0.480782232	1.41E-15
-0.565342158	4.29E-22	-0.442866748	3.43E-13	0.130642607	0.041033887
-0.627058907	3.54E-28	-0.469078998	8.29E-15	0.383053234	5.53E-10
-0.469479237	7.81E-15	-0.46864579	8.84E-15	0.406972671	3.43E-11
-0.327625129	1.54E-07	-0.382402067	5.95E-10	0.27105102	1.69E-05
0.460083287	3.08E-14	0.383815836	5.08E-10	-0.168018313	0.008408447
0.271936834	1.59E-05	0.272126807	1.56E-05	-0.281977097	7.38E-06
0.453127581	8.30E-14	0.478804371	1.92E-15	-0.392965544	1.80E-10
0.600973602	1.91E-25	0.486955607	5.42E-16	-0.468455959	9.09E-15
0.486112895	6.18E-16	0.22622937	0.0003579	-0.187529336	0.003214218
-0.195520343	0.002108883	-0.278579935	9.60E-06	0.234289571	0.000215524
-0.331897287	1.04E-07	-0.33090816	1.14E-07	0.234522819	0.000212328
-0.5913917	1.67E-24	-0.505652406	2.62E-17	0.394568585	1.49E-10
-0.189980327	0.002829358	-0.409015466	2.68E-11	0.199366255	0.001711759
-0.604771514	7.92E-26	-0.480300415	1.52E-15	0.438091964	6.54E-13
-0.644901036	3.35E-30	-0.586657123	4.76E-24	0.558454902	1.72E-21
-0.367343426	3.05E-09	-0.381129925	6.85E-10	0.586954853	4.46E-24
-0.248270562	8.57E-05	-0.425635406	3.35E-12	0.10680306	0.095324248
-0.452235044	9.41E-14	-0.49642109	1.20E-16	0.339676835	4.96E-08
-0.310210941	7.29E-07	-0.225419722	0.000376239	0.382428404	5.93E-10
0.540159362	5.86E-20	0.281614984	7.59E-06	-0.160514359	0.011871772
-0.588010843	3.53E-24	-0.41729771	9.65E-12	0.286284988	5.27E-06
-0.361116902	5.85E-09	-0.384069811	4.94E-10	0.471545955	5.74E-15
-0.488116329	4.51E-16	-0.357940668	8.11E-09	0.449517901	1.37E-13
-0.364743149	4.01E-09	-0.395182176	1.39E-10	0.254733873	5.49E-05
-0.628786882	2.28E-28	-0.548100798	1.30E-20	0.590228111	2.17E-24

0.646663146	2.08E-30	0.386802249	3.63E-10	-0.084804914	0.185834394
-0.537554331	9.52E-20	-0.542137071	4.04E-20	0.414329961	1.40E-11
-0.445330973	2.45E-13	-0.45362699	7.73E-14	0.326012963	1.79E-07
0.292997988	3.08E-06	0.303310726	1.31E-06	-0.083839564	0.19091082
0.699825198	2.27E-37	0.725244344	2.86E-41	-0.363940988	4.36E-09
-0.450986828	1.12E-13	-0.431981811	1.47E-12	0.461090371	2.67E-14
0.377050472	1.07E-09	0.338928896	5.33E-08	-0.086300646	0.178165844
-0.492382313	2.29E-16	-0.316458953	4.22E-07	0.470735572	6.48E-15
-0.329058156	1.35E-07	-0.428045217	2.46E-12	0.32509679	1.94E-07
-0.588954472	2.87E-24	-0.528437862	5.04E-19	0.380677721	7.20E-10
0.347871976	2.23E-08	0.673591439	9.41E-34	-0.298229283	2.01E-06
-0.182479298	0.004160275	-0.17163951	0.007084266	-0.055033609	0.39108183
0.285119402	5.77E-06	0.183688735	0.003913283	-0.187038119	0.00329683
0.563586151	6.13E-22	0.438551644	6.15E-13	-0.322887031	2.38E-07
-0.217245665	0.000617005	-0.297778116	2.08E-06	0.264712636	2.70E-05
-0.240379116	0.000145199	-0.377836978	9.85E-10	0.119821954	0.061113821
0.399119857	8.77E-11	0.359739819	6.74E-09	-0.115085302	0.072155345
-0.626374607	4.20E-28	-0.579393344	2.29E-23	0.501170694	5.51E-17
0.633240013	7.29E-29	0.492791738	2.15E-16	-0.403838805	5.01E-11
-0.575445366	5.29E-23	-0.567188578	2.94E-22	0.407956701	3.05E-11
0.717712924	4.53E-40	0.450146797	1.26E-13	-0.413215882	1.60E-11
-0.435428745	9.32E-13	-0.514057809	6.31E-18	0.187810811	0.003167729
-0.364675482	4.04E-09	-0.380715718	7.17E-10	0.408175045	2.97E-11
-0.49113032	2.80E-16	-0.427005817	2.81E-12	0.316268591	4.29E-07
-0.347719806	2.27E-08	-0.265013733	2.64E-05	0.219800749	0.000529629
0.408225505	2.95E-11	0.431047042	1.66E-12	-0.154516443	0.015488432
-0.439608315	5.33E-13	-0.407649874	3.16E-11	0.476819524	2.59E-15
0.498518628	8.51E-17	0.314420529	5.05E-07	-0.481748854	1.22E-15
-0.575336684	5.41E-23	-0.571948387	1.10E-22	0.432502362	1.37E-12
-0.626731423	3.84E-28	-0.493682203	1.86E-16	0.354482367	1.15E-08
-0.381933852	6.26E-10	-0.421151985	5.94E-12	0.178861369	0.004985261
0.305691851	1.07E-06	0.336101558	6.98E-08	-0.522212828	1.53E-18
0.435004657	9.86E-13	0.623002999	9.78E-28	0.008848718	0.89039949
0.563508425	6.23E-22	0.429929631	1.92E-12	-0.387726445	3.27E-10
-0.273132961	1.45E-05	-0.293937523	2.85E-06	0.232541687	0.000240943
-0.436820999	7.75E-13	-0.366353483	3.38E-09	0.504289752	3.29E-17
0.615857203	5.66E-27	0.519381757	2.51E-18	-0.241045956	0.000138966
0.448688299	1.54E-13	0.491149418	2.79E-16	-0.517379025	3.56E-18
0.434428298	1.06E-12	0.331879892	1.04E-07	-0.150238206	0.018625434
-0.558764694	1.61E-21	-0.436838446	7.73E-13	0.190082265	0.002814297

0.584660907	7.36E-24	0.616845239	4.46E-27	-0.131788208	0.039278117
-0.64286893	5.79E-30	-0.514044941	6.33E-18	0.338592941	5.51E-08
0.290988971	3.62E-06	0.186804719	0.003336752	-0.330824216	1.15E-07
-0.359993	6.57E-09	-0.248515342	8.43E-05	0.064036118	0.318171355
0.673246804	1.04E-33	0.443451285	3.17E-13	-0.340008817	4.81E-08
0.404007184	4.91E-11	0.492280579	2.33E-16	-0.074646305	0.244404174
0.285017587	5.82E-06	0.166057465	0.009213678	-0.379696323	8.02E-10
-0.514096818	6.27E-18	-0.357340485	8.62E-09	0.075724459	0.237638475
0.544080825	2.80E-20	0.437433469	7.14E-13	-0.112472439	0.078909591
-0.41262729	1.72E-11	-0.431068183	1.66E-12	0.374698649	1.39E-09
0.520112134	2.21E-18	0.399663091	8.22E-11	-0.280265541	8.43E-06
0.395917763	1.28E-10	0.345258963	2.89E-08	-0.128500127	0.04449504
-0.605553566	6.60E-26	-0.409876948	2.41E-11	0.555985427	2.80E-21
-0.410118906	2.34E-11	-0.416497053	1.07E-11	0.277563069	1.04E-05
-0.654128999	2.66E-31	-0.644918336	3.34E-30	0.421253367	5.86E-12
-0.58264378	1.14E-23	-0.567254493	2.90E-22	0.314343089	5.09E-07
0.403841452	5.01E-11	0.289069607	4.22E-06	-0.302373552	1.42E-06
0.41179177	1.91E-11	0.326087415	1.78E-07	-0.345655444	2.78E-08
0.516518442	4.13E-18	0.685108586	2.71E-35	-0.319847602	3.12E-07
0.39168874	2.08E-10	0.420459749	6.48E-12	0.004274119	0.946933014
0.588806527	2.97E-24	0.503894021	3.51E-17	-0.260648679	3.62E-05
0.456002485	5.53E-14	0.519221678	2.58E-18	-0.170213673	0.007581663
0.631626108	1.10E-28	0.503312099	3.87E-17	-0.169424165	0.00787026
-0.418408939	8.40E-12	-0.363762099	4.44E-09	0.063745279	0.3203788
-0.481927391	1.19E-15	-0.453425235	7.96E-14	0.442636475	3.54E-13
-0.562502213	7.63E-22	-0.50874183	1.56E-17	0.274316739	1.33E-05
-0.449983785	1.29E-13	-0.358164596	7.92E-09	0.515973759	4.54E-18
-0.476442055	2.75E-15	-0.476040137	2.92E-15	0.279675965	8.82E-06
-0.589582164	2.50E-24	-0.49003195	3.33E-16	0.511153817	1.04E-17
-0.464899457	1.53E-14	-0.379468206	8.23E-10	0.427300759	2.70E-12
0.612547443	1.26E-26	0.288729128	4.34E-06	-0.415815197	1.16E-11
-0.521670942	1.68E-18	-0.58487933	7.01E-24	0.37131111	2.00E-09
0.382515084	5.87E-10	0.661515998	3.28E-32	-0.180341536	0.004631444
-0.470748316	6.47E-15	-0.571815217	1.13E-22	0.200939238	0.001570025
-0.147310554	0.021078195	-0.032381925	0.613982066	0.405626058	4.04E-11
0.60588699	6.10E-26	0.503217348	3.93E-17	-0.309219324	7.94E-07
0.459117878	3.54E-14	0.47289761	4.69E-15	-0.306656434	9.89E-07
0.335586965	7.33E-08	0.339984671	4.82E-08	-0.196328295	0.002019082
0.565183787	4.43E-22	0.490436934	3.12E-16	-0.358175709	7.92E-09
0.439886053	5.14E-13	0.297772941	2.08E-06	-0.137603489	0.03131296

0.085914476	0.180122933	-0.192927895	0.002422157	0.273707654	1.39E-05
0.483673185	9.05E-16	0.45337754	8.01E-14	-0.268741758	2.01E-05
0.389689437	2.62E-10	0.425369711	3.47E-12	-0.274101554	1.35E-05
-0.565449861	4.20E-22	-0.462715914	2.11E-14	0.264340273	2.77E-05
-0.366202353	3.44E-09	0.072428297	0.258742479	0.440001922	5.06E-13
0.240324699	0.000145719	0.1712656	0.007211804	-0.137142031	0.031890276
0.615450885	6.25E-27	0.477786284	2.24E-15	-0.305999189	1.05E-06
0.431563079	1.55E-12	0.224352775	0.000401741	-0.186071882	0.003464961
-0.325907622	1.81E-07	-0.225715499	0.000369441	0.22108103	0.000490295
-0.414839788	1.31E-11	-0.381910798	6.28E-10	0.054639779	0.39448592
0.405345037	4.18E-11	0.570518614	1.48E-22	-0.115581719	0.070926897
0.29484135	2.65E-06	0.445108464	2.53E-13	-0.264733039	2.70E-05
-0.569472376	1.84E-22	-0.380476323	7.36E-10	0.514835727	5.52E-18
-0.362676338	4.97E-09	-0.478296755	2.07E-15	-0.085603534	0.181710269
-0.465117001	1.49E-14	-0.509710642	1.32E-17	0.172151626	0.006912854
-0.330576308	1.17E-07	-0.476881815	2.57E-15	0.369737223	2.36E-09
-0.33371576	8.75E-08	-0.312852513	5.80E-07	0.37529531	1.30E-09
-0.473218015	4.47E-15	-0.352596026	1.39E-08	0.245932264	0.000100381
-0.434054877	1.12E-12	-0.41295835	1.65E-11	0.430722078	1.73E-12
0.372666249	1.73E-09	0.380484271	7.36E-10	-0.128068118	0.045221815
0.44340446	3.19E-13	0.393098396	1.77E-10	-0.509060663	1.48E-17
0.442201895	3.76E-13	0.397142247	1.11E-10	-0.056170446	0.381355431
-0.562370566	7.84E-22	-0.480672968	1.44E-15	0.454741695	6.61E-14
0.28040926	8.34E-06	0.365400646	3.74E-09	-0.3148779	4.85E-07
-0.53910013	7.14E-20	-0.572277898	1.03E-22	0.232113543	0.000247582
-0.493136518	2.03E-16	-0.608807386	3.07E-26	0.334106583	8.43E-08
0.342129643	3.92E-08	0.193849927	0.002306188	-0.355976399	9.91E-09
0.266421698	2.38E-05	0.361908858	5.39E-09	-0.131861249	0.039168356
0.558513731	1.70E-21	0.455066863	6.31E-14	-0.388757281	2.91E-10
-0.692755396	2.34E-36	-0.582275839	1.23E-23	0.477632275	2.29E-15
-0.384465258	4.72E-10	-0.471117204	6.12E-15	0.206763764	0.001133686
-0.428440978	2.33E-12	-0.015310428	0.811544096	0.340901354	4.41E-08
0.609918953	2.36E-26	0.308164226	8.69E-07	-0.299356033	1.83E-06
-0.288416524	4.45E-06	-0.267268621	2.24E-05	0.154684559	0.015375219
-0.431685554	1.53E-12	-0.499095419	7.74E-17	0.172572578	0.006774739
-0.344513655	3.11E-08	-0.555605534	3.02E-21	0.249233212	8.03E-05
-0.482234275	1.13E-15	-0.505125398	2.86E-17	0.304635534	1.17E-06
-0.69251512	2.53E-36	-0.594475049	8.39E-25	0.375429684	1.28E-09
-0.366534539	3.32E-09	-0.339675032	4.97E-08	0.309144964	7.99E-07
-0.392616951	1.87E-10	-0.272446752	1.53E-05	0.463220587	1.96E-14

-0.650862371	6.59E-31	-0.552502379	5.55E-21	0.447340248	1.86E-13
0.46717174	1.10E-14	0.293614871	2.93E-06	-0.352364167	1.43E-08
0.587953482	3.58E-24	0.474328335	3.78E-15	-0.270280512	1.79E-05
0.306390894	1.01E-06	0.370119523	2.27E-09	0.232188771	0.000246403
-0.646424328	2.22E-30	-0.614257605	8.34E-27	0.403174201	5.42E-11
-0.54801899	1.32E-20	-0.453449364	7.93E-14	0.533156636	2.14E-19
-0.421721428	5.53E-12	-0.433650415	1.18E-12	0.261502379	3.40E-05
-0.315605369	4.55E-07	-0.240330751	0.000145661	0.369287024	2.48E-09
-0.612682426	1.22E-26	-0.546155132	1.89E-20	0.494981402	1.51E-16
-0.583247152	1.00E-23	-0.574793709	6.07E-23	0.360327303	6.34E-09
-0.63022152	1.58E-28	-0.502714984	4.27E-17	0.407723367	3.14E-11
0.715477238	1.01E-39	0.520276692	2.15E-18	-0.448504898	1.58E-13
-0.488529185	4.23E-16	-0.377159304	1.06E-09	0.258764341	4.14E-05
0.31538181	4.64E-07	0.311171464	6.71E-07	-0.23945822	0.000154237
0.380404708	7.42E-10	0.318060173	3.66E-07	-0.362144923	5.26E-09
0.439652508	5.30E-13	0.501413515	5.29E-17	0.036001274	0.574927681
0.520413121	2.10E-18	0.50329121	3.88E-17	-0.329002837	1.36E-07
0.251132205	7.05E-05	0.343122668	3.56E-08	0.239691897	0.000151895
-0.617345614	3.94E-27	-0.52460199	1.00E-18	0.641799154	7.70E-30
0.478678581	1.95E-15	0.403792399	5.03E-11	-0.299490914	1.81E-06
-0.378828496	8.83E-10	-0.344560055	3.09E-08	0.160470859	0.011895061
0.58689318	4.52E-24	0.380151121	7.63E-10	0.014753048	0.818283489
0.495324436	1.43E-16	0.296486385	2.32E-06	-0.285358417	5.67E-06
-0.09800447	0.126053198	-0.227763093	0.000325416	0.060891679	0.342563488
0.392806346	1.83E-10	0.555927433	2.83E-21	-0.315757032	4.49E-07
0.490550731	3.07E-16	0.368502476	2.70E-09	-0.172928014	0.006660044
-0.510378743	1.18E-17	-0.398477476	9.45E-11	0.173546068	0.006464732
-0.411740018	1.92E-11	-0.44998894	1.29E-13	0.252017851	6.63E-05
0.46865579	8.82E-15	0.47848083	2.01E-15	-0.20026979	0.001628979
0.412127078	1.83E-11	0.274740825	1.29E-05	-0.423526971	4.39E-12
0.382116728	6.14E-10	0.240964052	0.000139718	-0.302745336	1.38E-06
-0.483013045	1.00E-15	-0.532200323	2.55E-19	0.477271332	2.42E-15
-0.555217872	3.26E-21	-0.464934266	1.53E-14	0.402186272	6.10E-11
-0.587901652	3.62E-24	-0.397169848	1.10E-10	0.497184535	1.06E-16
0.286006911	5.38E-06	0.310203059	7.29E-07	0.00497073	0.938299924
0.246668126	9.55E-05	0.354759033	1.12E-08	0.080363237	0.210031411
0.211234377	0.000877696	0.247021462	9.33E-05	-0.082588094	0.197641866
0.221575093	0.000475854	0.306593433	9.94E-07	-0.447050567	1.93E-13
-0.38265978	5.78E-10	-0.48079147	1.41E-15	0.386868179	3.60E-10
0.623434473	8.78E-28	0.458482196	3.88E-14	-0.336103293	6.98E-08

-0.572864343	9.10E-23	-0.3993377	8.54E-11	0.560806063	1.07E-21
-0.503237778	3.92E-17	-0.469123404	8.23E-15	-0.042201968	0.510872262
0.428238889	2.40E-12	0.306997122	9.61E-07	-0.31116167	6.71E-07
0.394842568	1.45E-10	0.369518079	2.42E-09	-0.329514224	1.30E-07
0.655377113	1.88E-31	0.496944912	1.10E-16	-0.322291185	2.51E-07
0.410486279	2.24E-11	0.412599774	1.73E-11	0.047594838	0.458337245
-0.226478314	0.00035243	-0.352303506	1.44E-08	0.189110504	0.002960872
-0.368806146	2.61E-09	-0.460169115	3.05E-14	0.179388641	0.004856529
-0.439419935	5.47E-13	-0.508999297	1.49E-17	0.410392469	2.27E-11
-0.62106108	1.58E-27	-0.559749647	1.33E-21	0.437069481	7.49E-13
-0.207556167	0.001083822	-0.352474535	1.41E-08	0.30835978	8.55E-07
-0.306214783	1.03E-06	-0.348773282	2.04E-08	0.47100398	6.23E-15
0.345614767	2.79E-08	0.379773215	7.96E-10	-0.06777687	0.290664057
0.725450707	2.65E-41	0.756265675	1.14E-46	-0.330383362	1.19E-07
0.544796911	2.44E-20	0.622327428	1.16E-27	-0.338732852	5.43E-08
-0.376634962	1.12E-09	-0.307970825	8.84E-07	0.544110325	2.78E-20
0.45840068	3.93E-14	0.296952936	2.23E-06	-0.335028242	7.73E-08
-0.498847131	8.06E-17	-0.55038351	8.37E-21	0.418588952	8.21E-12
-0.594030802	9.27E-25	-0.464083145	1.73E-14	0.354893391	1.11E-08
0.434156446	1.10E-12	0.360091806	6.50E-09	-0.34446514	3.12E-08
-0.277088257	1.08E-05	-0.381136688	6.84E-10	0.200586458	0.001600845
-0.351954842	1.49E-08	-0.015598059	0.808071679	0.245990678	1.00E-04
0.471973917	5.39E-15	0.409594217	2.50E-11	0.074133826	0.247666797
-0.318060506	3.66E-07	-0.520216111	2.17E-18	0.295134591	2.59E-06
-0.379444822	8.25E-10	-0.413012588	1.64E-11	0.442392868	3.66E-13
-0.421807088	5.47E-12	-0.480682515	1.44E-15	0.346628373	2.53E-08
-0.185331979	0.00359891	-0.265124044	2.62E-05	0.194542335	0.002222449
-0.503077637	4.02E-17	-0.511092092	1.05E-17	0.366821815	3.22E-09
-0.418893339	7.90E-12	-0.383684348	5.15E-10	0.192725036	0.002448373
0.370141508	2.26E-09	0.214232933	0.000737082	-0.170465572	0.007491589
-0.369346711	2.46E-09	-0.520451058	2.08E-18	0.07589318	0.236591723
0.686224702	1.90E-35	0.462942832	2.04E-14	-0.561169154	9.98E-22
0.559607532	1.36E-21	0.563473445	6.27E-22	0.090673098	0.157092971
0.451201144	1.09E-13	0.480402836	1.50E-15	-0.398114651	9.87E-11
-0.026837138	0.675950991	0.003110306	0.961369465	0.063220062	0.324390292
-0.300170747	1.71E-06	-0.317052639	4.01E-07	-0.014077107	0.826474312
-0.404882053	4.42E-11	-0.416820471	1.02E-11	0.083225822	0.194190583
-0.560334575	1.18E-21	-0.652233538	4.51E-31	0.188108997	0.003119146
0.351337082	1.58E-08	0.457904013	4.21E-14	-0.119094333	0.062713491
-0.258403622	4.25E-05	-0.213259534	0.000780269	0.350292866	1.76E-08

0.644351057	3.89E-30	0.459587577	3.31E-14	-0.457256169	4.62E-14
0.530733245	3.33E-19	0.412306014	1.79E-11	-0.064040138	0.318140913
0.372163824	1.82E-09	0.364343729	4.18E-09	-0.449548414	1.37E-13
-0.365498015	3.70E-09	-0.320696618	2.89E-07	0.348185352	2.17E-08
0.35287427	1.36E-08	0.349210776	1.96E-08	-0.307057766	9.56E-07
0.474383049	3.75E-15	0.458942793	3.63E-14	-0.371703612	1.92E-09
0.626160724	4.43E-28	0.427749483	2.55E-12	-0.136433292	0.032794608
0.190813305	0.002708406	0.235434858	0.00020025	-0.384359396	4.78E-10
-0.589301578	2.66E-24	-0.460306103	2.99E-14	0.452131244	9.54E-14
-0.42444996	3.90E-12	-0.43374622	1.17E-12	0.347431978	2.33E-08
0.614388698	8.08E-27	0.534031195	1.82E-19	-0.615011013	6.95E-27
-0.514087269	6.28E-18	-0.585793034	5.75E-24	0.173879563	0.006361484
0.448581259	1.57E-13	0.446412994	2.11E-13	-0.175348059	0.005924179
-0.390272908	2.45E-10	-0.277293649	1.06E-05	0.549334014	1.03E-20
-0.460788269	2.79E-14	-0.332055181	1.02E-07	0.189859207	0.002847347
-0.511375391	9.98E-18	-0.577178595	3.67E-23	0.010173539	0.874117341
-0.705599075	3.22E-38	-0.636735021	2.94E-29	0.393829334	1.63E-10
0.3330138	9.35E-08	0.397575178	1.05E-10	0.17868271	0.00502957
0.671293105	1.87E-33	0.683219538	4.90E-35	-0.300347363	1.68E-06
0.427470836	2.65E-12	0.404475283	4.64E-11	0.023464814	0.7147722
0.460155117	3.05E-14	0.310141816	7.33E-07	-0.152510545	0.016896549
0.638430512	1.88E-29	0.608394301	3.38E-26	-0.148889256	0.01972291
-0.289331494	4.14E-06	-0.237170033	0.000179024	0.27004445	1.83E-05
-0.500476352	6.17E-17	-0.497649751	9.80E-17	-0.001898079	0.976419777
-0.447543964	1.81E-13	-0.474815602	3.51E-15	0.423340009	4.50E-12
0.454535572	6.80E-14	0.348629111	2.07E-08	-0.490825612	2.94E-16
-0.375785078	1.23E-09	-0.323541811	2.24E-07	0.062810164	0.327543493
-0.416300264	1.09E-11	-0.484904769	7.47E-16	0.657794392	9.49E-32
0.701319596	1.38E-37	0.606157514	5.73E-26	-0.424313996	3.97E-12
-0.635356639	4.21E-29	-0.642282207	6.77E-30	0.420372661	6.56E-12
-0.43885656	5.90E-13	-0.42861806	2.28E-12	0.272046736	1.57E-05
-0.515217326	5.17E-18	-0.436324419	8.28E-13	0.20996721	0.000944234
0.716981547	5.90E-40	0.612673808	1.22E-26	-0.368412286	2.72E-09
0.648948046	1.12E-30	0.572377383	1.01E-22	-0.299555638	1.80E-06
0.241589627	0.000134071	0.433625216	1.18E-12	0.116787172	0.068014685
-0.323746488	2.20E-07	-0.234826008	0.000208239	0.505798759	2.56E-17
-0.463801246	1.80E-14	-0.393208181	1.75E-10	0.254042549	5.76E-05
-0.252314014	6.50E-05	-0.278263695	9.83E-06	0.078033751	0.223592187
-0.539794994	6.27E-20	-0.481130595	1.34E-15	0.514149167	6.21E-18
-0.456557295	5.11E-14	-0.358884273	7.36E-09	0.492901667	2.11E-16

-0.451789026	1.00E-13	-0.367927287	2.86E-09	0.050499716	0.431337225
-0.518022864	3.18E-18	-0.490169766	3.26E-16	0.432146696	1.44E-12
-0.358412143	7.73E-09	-0.37323257	1.62E-09	0.032278795	0.615111739
-0.224589746	0.000395942	-0.333296485	9.10E-08	0.02841275	0.658094966
-0.508504491	1.62E-17	-0.477789564	2.24E-15	0.430095353	1.88E-12
-0.379592039	8.12E-10	-0.37717123	1.06E-09	0.441131544	4.34E-13
-0.455076339	6.30E-14	-0.501966049	4.83E-17	0.241048382	0.000138944
-0.520943236	1.91E-18	-0.536451514	1.17E-19	0.24068693	0.00014229
-0.567338408	2.85E-22	-0.582965803	1.06E-23	0.354643109	1.13E-08
-0.334186469	8.37E-08	-0.262187338	3.24E-05	0.058015254	0.365889249
-0.00975602	0.879243306	-0.190866998	0.002700773	-0.036783617	0.566641856
-0.252575312	6.38E-05	-0.298471713	1.97E-06	0.12386038	0.052834739
-0.585545456	6.07E-24	-0.513566748	6.87E-18	0.344119926	3.23E-08
-0.396476683	1.19E-10	-0.343302995	3.50E-08	0.4414692	4.15E-13
-0.55746757	2.09E-21	-0.489014849	3.92E-16	0.611351136	1.68E-26
-0.516228462	4.34E-18	-0.517046549	3.77E-18	0.241071117	0.000138736
-0.276517277	1.12E-05	-0.542476567	3.79E-20	0.265375423	2.57E-05
0.42333106	4.50E-12	0.464830963	1.55E-14	-0.394179935	1.56E-10
0.109990595	0.085789859	0.382724042	5.74E-10	0.04001723	0.533011701
0.286487705	5.18E-06	0.225994651	0.000363129	-0.217755793	0.000598561
0.286340664	5.24E-06	0.444163036	2.88E-13	-0.113878421	0.07521425
-0.476021855	2.93E-15	-0.537624243	9.40E-20	0.305219976	1.12E-06
-0.282558712	7.06E-06	-0.381242244	6.76E-10	0.33279968	9.54E-08
-0.563446524	6.31E-22	-0.379935088	7.82E-10	0.224441777	0.000399554
0.578518648	2.76E-23	0.439320962	5.54E-13	-0.222204534	0.000458027
-0.38690539	3.59E-10	-0.383534595	5.24E-10	0.146938261	0.021409309
0.373089157	1.65E-09	0.566384496	3.47E-22	-0.076802657	0.231005084
0.489608906	3.56E-16	0.380853642	7.06E-10	0.036648357	0.568070325
-0.44313957	3.31E-13	-0.428474431	2.32E-12	0.123209655	0.054102105
0.420970917	6.08E-12	0.384608044	4.65E-10	-0.271315744	1.66E-05
-0.277529047	1.04E-05	-0.325315299	1.91E-07	0.179502961	0.004829016
0.333529797	8.90E-08	0.306492787	1.00E-06	-0.266992705	2.29E-05
-0.521735615	1.66E-18	-0.565411426	4.23E-22	0.522633068	1.42E-18
-0.418340096	8.47E-12	-0.336882257	6.48E-08	0.457515036	4.46E-14
-0.436608982	7.97E-13	-0.52077443	1.97E-18	0.241920153	0.000131174
-0.253189954	6.12E-05	-0.371408021	1.98E-09	0.425665945	3.34E-12
-0.484666457	7.75E-16	-0.311807186	6.35E-07	0.221467438	0.000478967
-0.320520262	2.94E-07	-0.472728823	4.81E-15	0.193767687	0.002316323
-0.355954484	9.93E-09	-0.388481898	3.00E-10	0.328239007	1.46E-07
-0.520598708	2.03E-18	-0.493257842	1.99E-16	0.430207822	1.85E-12

-0.453027122	8.41E-14	-0.391796118	2.05E-10	0.271924207	1.59E-05
0.399654632	8.23E-11	0.360595894	6.17E-09	-0.151645919	0.017537153
-0.584595104	7.46E-24	-0.569979973	1.66E-22	0.453225219	8.18E-14
0.401400088	6.69E-11	0.435297738	9.49E-13	0.031785104	0.620532098
-0.444938315	2.59E-13	-0.496924965	1.10E-16	0.38302223	5.55E-10
0.40280293	5.67E-11	0.688591897	8.97E-36	-0.435517407	9.21E-13
-0.297359132	2.16E-06	-0.390245272	2.45E-10	0.32047728	2.95E-07
-0.525243479	8.93E-19	-0.536351156	1.19E-19	0.439971063	5.08E-13
-0.596089522	5.83E-25	-0.358982064	7.29E-09	0.50549334	2.69E-17
-0.446802411	2.00E-13	-0.356806364	9.11E-09	0.365554639	3.68E-09
-0.517318812	3.60E-18	-0.453365973	8.02E-14	0.485011484	7.34E-16
0.485988113	6.30E-16	0.561602763	9.15E-22	-0.263419245	2.97E-05
0.362847559	4.89E-09	0.205324712	0.001229668	-0.205197313	0.001238515
0.438779369	5.96E-13	0.40825056	2.94E-11	-0.312478056	5.99E-07
-0.521249152	1.81E-18	-0.537310187	9.96E-20	0.401607075	6.53E-11
-0.26071414	3.60E-05	-0.359324074	7.04E-09	0.27533329	1.23E-05
-0.386873626	3.60E-10	-0.424992762	3.64E-12	0.423767549	4.26E-12
-0.58212284	1.27E-23	-0.591049533	1.80E-24	0.182416243	0.004173529
-0.494175279	1.72E-16	-0.452893829	8.57E-14	0.416426034	1.08E-11
-0.587075229	4.34E-24	-0.578296227	2.89E-23	0.403770586	5.05E-11
-0.457884773	4.23E-14	-0.475504805	3.17E-15	0.437507881	7.07E-13
-0.555080528	3.35E-21	-0.478641304	1.96E-15	0.551231062	7.10E-21
0.689671357	6.35E-36	0.606362973	5.46E-26	-0.370266667	2.23E-09
0.247479578	9.04E-05	0.169585279	0.007810585	-0.440077169	5.01E-13
-0.413357892	1.57E-11	-0.481899055	1.19E-15	0.138885158	0.029756039
0.591796355	1.53E-24	0.313985401	5.25E-07	-0.50235928	4.53E-17
-0.675377084	5.49E-34	-0.662961374	2.16E-32	0.389585275	2.65E-10
-0.389223185	2.76E-10	-0.330105405	1.23E-07	0.377722166	9.97E-10
0.210568616	0.000912096	0.385831068	4.05E-10	-0.289603557	4.05E-06
-0.392761151	1.84E-10	-0.374460629	1.42E-09	0.109009948	0.088637872
-0.227605267	0.000328627	-0.365255948	3.80E-09	0.252956272	6.22E-05
-0.424011613	4.13E-12	-0.454891936	6.47E-14	0.241990457	0.000130566
-0.249591267	7.83E-05	-0.34421623	3.20E-08	0.310775482	6.94E-07
0.348775058	2.04E-08	0.266278583	2.41E-05	-0.18196363	0.004269793
0.466743559	1.17E-14	0.342446781	3.80E-08	-0.104668193	0.10216853
-0.488885856	4.00E-16	-0.552003436	6.11E-21	0.11505193	0.072238547
0.431846063	1.50E-12	0.193238204	0.002382548	-0.282773761	6.94E-06
-0.213164685	0.000784599	-0.185965523	0.003483934	0.101484647	0.113090051
0.205865307	0.001192769	0.161867617	0.011167057	-0.327817863	1.51E-07
0.38154605	6.54E-10	0.347251464	2.37E-08	-0.273927795	1.37E-05

-0.519221348	2.58E-18	-0.484613039	7.81E-16	0.480023263	1.59E-15
0.376872791	1.09E-09	0.391814947	2.05E-10	-0.215501099	0.000684125
-0.219868831	0.000527466	-0.220113031	0.000519774	0.175904636	0.005765603
-0.604543674	8.35E-26	-0.521111235	1.85E-18	0.325315808	1.91E-07
0.311871241	6.31E-07	0.492674062	2.19E-16	-0.173040558	0.006624091
-0.440365426	4.82E-13	-0.34369922	3.36E-08	0.407843215	3.09E-11
-0.55637407	2.59E-21	-0.564110271	5.51E-22	0.336835011	6.51E-08
0.577173603	3.67E-23	0.428431011	2.34E-12	-0.305584811	1.08E-06
0.457500817	4.46E-14	0.588878567	2.92E-24	-0.269294508	1.93E-05
0.662971852	2.16E-32	0.713598454	1.98E-39	-0.431058649	1.66E-12
-0.507122145	2.05E-17	-0.56435838	5.24E-22	0.203549112	0.001358359
-0.263455678	2.96E-05	-0.284619067	6.01E-06	0.166308843	0.009106764
-0.514252169	6.11E-18	-0.48884959	4.02E-16	0.117054524	0.067382222
-0.38153483	6.55E-10	-0.449779848	1.33E-13	0.182723908	0.004109215
0.640786792	1.01E-29	0.608758347	3.10E-26	-0.570556115	1.47E-22
0.319663609	3.18E-07	0.247451831	9.06E-05	-0.51202328	8.94E-18
0.320022951	3.08E-07	0.383749766	5.12E-10	-0.36307272	4.77E-09
0.20691559	0.001123972	0.070419407	0.272219875	-0.10918434	0.088125938
0.363292984	4.66E-09	0.373519235	1.57E-09	-0.366382991	3.37E-09
0.429331485	2.08E-12	0.473705152	4.15E-15	-0.416984597	1.00E-11
0.325218987	1.92E-07	0.370408674	2.20E-09	-0.288453232	4.44E-06
0.42071129	6.28E-12	0.292863972	3.11E-06	-0.49653742	1.17E-16
-0.058384097	0.362844327	-0.142185793	0.026047887	0.412856816	1.67E-11
0.481812654	1.21E-15	0.499946796	6.73E-17	-0.396121362	1.25E-10
0.394825974	1.45E-10	0.268164372	2.10E-05	-0.40359964	5.15E-11
0.304964886	1.14E-06	0.213998692	0.000747268	-0.469167307	8.18E-15
0.411898454	1.88E-11	0.297899767	2.06E-06	-0.490706917	2.99E-16
0.444106165	2.90E-13	0.357370154	8.60E-09	-0.483122354	9.85E-16
0.201137385	0.001552954	0.25726184	4.60E-05	-0.159560465	0.012391788
0.460236925	3.02E-14	0.379806943	7.93E-10	-0.449132721	1.45E-13
0.397423369	1.07E-10	0.309523201	7.74E-07	-0.470405366	6.81E-15
0.539544196	6.58E-20	0.648647722	1.21E-30	-0.507253964	2.00E-17
0.44540921	2.42E-13	0.445023645	2.56E-13	-0.356626729	9.27E-09
0.36192477	5.38E-09	0.251560224	6.84E-05	-0.514215878	6.14E-18
0.513104955	7.43E-18	0.539856397	6.20E-20	-0.337854398	5.91E-08
0.576683055	4.07E-23	0.550581043	8.06E-21	-0.336346605	6.82E-08
0.24602677	9.97E-05	0.354379629	1.17E-08	-0.382252063	6.05E-10
0.439979359	5.07E-13	0.587316924	4.12E-24	-0.324708543	2.01E-07
0.334485227	8.14E-08	0.306900993	9.69E-07	-0.343423523	3.46E-08
0.412966737	1.65E-11	0.294182432	2.80E-06	-0.449220753	1.43E-13

0.466718711	1.17E-14	0.411192611	2.06E-11	-0.353509067	1.27E-08
0.488886295	4.00E-16	0.415365499	1.23E-11	-0.505555674	2.66E-17
0.468428679	9.13E-15	0.476952677	2.54E-15	-0.35076569	1.68E-08
0.544962103	2.37E-20	0.481497476	1.27E-15	-0.516791873	3.94E-18
0.531703748	2.79E-19	0.517630883	3.41E-18	-0.534120718	1.79E-19
0.490903703	2.90E-16	0.441776582	3.98E-13	-0.473333721	4.39E-15
0.435524429	9.21E-13	0.458930224	3.64E-14	-0.375310682	1.30E-09
0.399703115	8.18E-11	0.426737787	2.91E-12	-0.405688952	4.01E-11
0.469593649	7.68E-15	0.328594396	1.41E-07	-0.504888601	2.98E-17
0.438552257	6.15E-13	0.348209777	2.16E-08	-0.518884574	2.74E-18
0.299295802	1.84E-06	0.344644613	3.07E-08	-0.304214382	1.22E-06
0.456170878	5.40E-14	0.564910212	4.69E-22	-0.329521421	1.29E-07
0.319055183	3.35E-07	0.19312266	0.002397227	-0.592068866	1.44E-24
0.373558058	1.57E-09	0.313701231	5.38E-07	-0.401189008	6.86E-11
0.250523533	7.35E-05	0.222895437	0.000439173	-0.334795265	7.90E-08
0.349519865	1.90E-08	0.313498548	5.48E-07	-0.268173561	2.10E-05
0.250031371	7.60E-05	0.158511457	0.012986708	-0.365027552	3.89E-09
0.342704233	3.71E-08	0.24620829	9.85E-05	-0.355102753	1.08E-08
0.228744542	0.000306091	0.194357569	0.002244519	-0.121142233	0.058297002
-0.315945144	4.42E-07	-0.359993094	6.57E-09	0.528920764	4.62E-19
0.219169746	0.000550072	0.124169152	0.052242052	-0.437131942	7.43E-13
0.335859419	7.15E-08	0.34491072	2.99E-08	-0.281172705	7.86E-06
0.406056986	3.84E-11	0.316485601	4.21E-07	-0.444859541	2.61E-13
0.422026981	5.32E-12	0.327746997	1.52E-07	-0.46941431	7.89E-15
-0.034488786	0.591105983	0.023054722	0.719545909	0.222572856	0.000447884
0.438001966	6.62E-13	0.289646594	4.03E-06	-0.651403465	5.67E-31
0.574731457	6.15E-23	0.499888011	6.80E-17	-0.376016328	1.20E-09
0.44023221	4.90E-13	0.485510788	6.79E-16	-0.400627597	7.34E-11
0.360265737	6.38E-09	0.237623331	0.000173835	-0.480461424	1.49E-15
0.716135037	8.00E-40	0.430847906	1.71E-12	-0.622635573	1.07E-27
0.440375178	4.81E-13	0.433457189	1.21E-12	-0.444127117	2.89E-13
0.449676531	1.34E-13	0.419118222	7.68E-12	-0.361347422	5.71E-09
0.489649939	3.54E-16	0.280901877	8.02E-06	-0.376620616	1.12E-09
0.281518641	7.65E-06	0.279459479	8.97E-06	-0.556161019	2.70E-21
-0.459306075	3.45E-14	-0.376273789	1.17E-09	0.312258216	6.10E-07
0.470504707	6.71E-15	0.490749593	2.97E-16	-0.107511252	0.093136189
0.563756396	5.92E-22	0.39343773	1.70E-10	-0.211434825	0.000867576
0.182196791	0.004219956	0.161922862	0.01113909	-0.473966237	3.99E-15
0.488368416	4.34E-16	0.369766495	2.36E-09	-0.47811365	2.13E-15
0.59117588	1.75E-24	0.553116493	4.92E-21	-0.448322547	1.62E-13

0.561656639	9.05E-22	0.430547067	1.77E-12	-0.518891521	2.73E-18
0.125816595	0.049172434	0.090494168	0.15791673	-0.244953405	0.000107196
0.367997001	2.84E-09	0.308009455	8.81E-07	-0.407977211	3.04E-11
0.356340464	9.55E-09	0.202983318	0.00140189	-0.313708855	5.38E-07
0.326414406	1.72E-07	0.310767858	6.95E-07	-0.253742813	5.89E-05
0.478384958	2.04E-15	0.446558307	2.07E-13	-0.255631156	5.16E-05
0.261154701	3.49E-05	0.235245874	0.000202699	-0.473838337	4.07E-15
0.775909076	1.58E-50	0.672120843	1.46E-33	-0.600111588	2.33E-25
0.620640395	1.76E-27	0.516278759	4.31E-18	-0.471669415	5.64E-15
0.415106205	1.27E-11	0.285763094	5.49E-06	-0.545449271	2.16E-20
0.418868608	7.92E-12	0.313674238	5.39E-07	-0.44369302	3.07E-13
0.547520621	1.45E-20	0.306294464	1.02E-06	-0.589829233	2.37E-24
0.419262514	7.54E-12	0.362239983	5.20E-09	-0.518204689	3.08E-18
0.097211914	0.129160255	0.03538378	0.581507534	-0.263418382	2.97E-05
0.620175468	1.97E-27	0.565700263	3.99E-22	-0.535332634	1.44E-19
0.486953243	5.42E-16	0.504049093	3.42E-17	-0.477197266	2.45E-15
0.268644096	2.03E-05	0.14928189	0.019397824	-0.544830537	2.43E-20
0.433248385	1.24E-12	0.347703248	2.27E-08	-0.559243523	1.47E-21
0.435107462	9.73E-13	0.280927022	8.01E-06	-0.478046392	2.15E-15
0.240382868	0.000145163	0.221728552	0.000471449	-0.359866101	6.65E-09
0.16420144	0.010038127	0.222004794	0.000463616	-0.174399535	0.006203446
0.553852068	4.26E-21	0.283213408	6.70E-06	-0.631563679	1.12E-28
0.37123525	2.01E-09	0.360707398	6.10E-09	-0.454761828	6.59E-14
0.549354848	1.02E-20	0.67325171	1.04E-33	-0.327311789	1.59E-07
0.516361855	4.24E-18	0.465669807	1.37E-14	-0.588638869	3.08E-24
0.267986976	2.13E-05	0.15849366	0.012997014	-0.379275341	8.41E-10
0.420169696	6.73E-12	0.276677444	1.11E-05	-0.256695816	4.79E-05
0.364068695	4.30E-09	0.259432359	3.95E-05	-0.498935806	7.95E-17
0.278064326	9.98E-06	0.223941727	0.000411987	-0.50735782	1.97E-17
0.508530557	1.62E-17	0.503714107	3.62E-17	-0.271432171	1.65E-05
0.506405429	2.31E-17	0.498701223	8.26E-17	-0.542909236	3.50E-20
0.365599816	3.66E-09	0.275083137	1.25E-05	-0.374454842	1.42E-09
0.441737867	4.00E-13	0.409703968	2.47E-11	-0.562767552	7.23E-22
0.466575921	1.20E-14	0.429168109	2.12E-12	-0.494777197	1.56E-16
0.350459602	1.73E-08	0.459922436	3.16E-14	-0.364562708	4.08E-09
0.321777191	2.63E-07	0.401215177	6.84E-11	-0.215529546	0.000682978
0.091548955	0.153107348	0.042378958	0.50909984	0.220422973	0.000510161
0.679839398	1.40E-34	0.590319646	2.12E-24	-0.619518077	2.32E-27
0.523021288	1.32E-18	0.685848749	2.14E-35	-0.424190242	4.03E-12
0.295017211	2.61E-06	0.298453722	1.97E-06	-0.40707385	3.39E-11

0.477045468	2.51E-15	0.332315662	9.98E-08	-0.329274202	1.32E-07
0.516249349	4.33E-18	0.628766425	2.29E-28	-0.30782285	8.95E-07
0.359703597	6.77E-09	0.222144478	0.0004597	-0.562468436	7.68E-22
0.5657638	3.94E-22	0.432671049	1.34E-12	-0.449319116	1.41E-13
-0.395615794	1.32E-10	-0.33115504	1.11E-07	0.555198063	3.27E-21
0.420779453	6.23E-12	0.42235765	5.10E-12	-0.259828087	3.84E-05
0.482870902	1.02E-15	0.338226349	5.70E-08	-0.4371669	7.40E-13
0.480939953	1.38E-15	0.459180181	3.51E-14	-0.499074519	7.77E-17
0.578982562	2.50E-23	0.57169736	1.16E-22	-0.456321792	5.28E-14
0.522701084	1.40E-18	0.468190067	9.45E-15	-0.339885806	4.87E-08
0.479062025	1.84E-15	0.381366868	6.67E-10	-0.538916461	7.39E-20
0.631718896	1.08E-28	0.492097883	2.40E-16	-0.641016911	9.49E-30
0.556200061	2.68E-21	0.514504697	5.85E-18	-0.631545699	1.13E-28
-0.237065783	0.000180237	-0.291994062	3.34E-06	0.478921155	1.88E-15
0.4988877	8.01E-17	0.396510881	1.19E-10	-0.55881177	1.60E-21
0.533850383	1.89E-19	0.418097279	8.73E-12	-0.513487013	6.96E-18
-0.442619465	3.55E-13	-0.393041562	1.78E-10	0.700231986	1.99E-37
0.530068696	3.75E-19	0.513378039	7.09E-18	-0.59171112	1.56E-24
0.359111018	7.19E-09	0.274778193	1.28E-05	-0.4324027	1.39E-12
0.33977912	4.92E-08	0.143325803	0.024862894	-0.247285995	9.16E-05
0.564257955	5.35E-22	0.456191287	5.38E-14	-0.526179713	7.55E-19
0.341502914	4.16E-08	0.213715076	0.000759775	-0.519358959	2.52E-18
0.581068173	1.60E-23	0.378769755	8.89E-10	-0.473152387	4.51E-15
0.50574582	2.58E-17	0.519025939	2.67E-18	-0.425966404	3.21E-12
0.059285119	0.355472633	0.003651034	0.954660184	-0.201379467	0.001532328
0.449593106	1.36E-13	0.281298444	7.78E-06	-0.387937997	3.19E-10
0.555837687	2.88E-21	0.511402614	9.94E-18	-0.458487388	3.88E-14
0.598192539	3.61E-25	0.419698126	7.14E-12	-0.624694299	6.41E-28
0.392782609	1.83E-10	0.328332287	1.44E-07	-0.332152336	1.01E-07
0.44857622	1.57E-13	0.344684805	3.06E-08	-0.468822983	8.61E-15
0.346237388	2.62E-08	0.271029479	1.70E-05	-0.455131297	6.25E-14
0.203369813	0.001372018	0.184868643	0.003685157	-0.331787747	1.05E-07
0.583689964	9.08E-24	0.562709506	7.32E-22	-0.443983949	2.95E-13
0.506167765	2.40E-17	0.379874179	7.87E-10	-0.584986889	6.85E-24
0.343732586	3.35E-08	0.326337926	1.74E-07	-0.413020902	1.64E-11
0.546004911	1.94E-20	0.523635755	1.19E-18	-0.43738571	7.18E-13
0.320770311	2.88E-07	0.238884454	0.000160131	-0.439571922	5.36E-13
0.319580896	3.20E-07	0.261995443	3.29E-05	-0.543419832	3.17E-20
0.455164089	6.22E-14	0.32677394	1.67E-07	-0.44721752	1.89E-13
0.188258315	0.003095072	0.108088632	0.091382096	-0.469138401	8.22E-15

0.500074652	6.59E-17	0.413701855	1.51E-11	-0.553261267	4.78E-21
0.555521834	3.07E-21	0.499866379	6.82E-17	-0.291928523	3.36E-06
0.462787493	2.09E-14	0.362872238	4.87E-09	-0.218251921	0.000581114
0.347079637	2.42E-08	0.273224712	1.44E-05	-0.405325978	4.19E-11

MMred	p.MMred	MMturquoise	p.MMturquoise	MMgrey	p.MMgrey
0.6500823	8.17E-31	0.644118714	4.14E-30	-0.312825286	5.81E-07
-0.088561402	0.167023082	-0.093160164	0.145975814	-0.153252021	0.016363541
0.089899477	0.160677937	0.167473521	0.008625643	-0.139467711	0.029070541
0.166733287	0.008928739	0.21082067	0.000898929	-0.035236222	0.583085038
0.338140015	5.75E-08	0.428629405	2.28E-12	-0.084412744	0.18788458
0.03140384	0.624732162	0.045462749	0.478739267	-0.188350633	0.003080273
0.381894851	6.29E-10	0.467812314	1.00E-14	-0.219684646	0.000533337
-0.053814258	0.401678909	-0.017444669	0.785870744	0.089533574	0.162394785
0.31236833	6.05E-07	0.383586048	5.21E-10	-0.318846048	3.42E-07
0.288044408	4.58E-06	0.341811331	4.04E-08	-0.276302029	1.14E-05
0.13587743	0.033519059	0.088914594	0.165330348	-0.23209302	0.000247904
0.261331309	3.45E-05	0.419943666	6.92E-12	-0.064209521	0.316859969
0.291114813	3.58E-06	0.185950187	0.003486677	-0.169778488	0.007739552
0.153724299	0.016031751	0.29274898	3.14E-06	-0.25672481	4.78E-05
0.280245055	8.44E-06	0.569198824	1.95E-22	-0.44101247	4.41E-13
0.359600581	6.84E-09	0.464430783	1.64E-14	-0.395178332	1.39E-10
0.196456633	0.002005143	0.271749887	1.61E-05	-0.217879925	0.000594151
-0.265280773	2.59E-05	-0.03322169	0.604817458	-0.352757868	1.37E-08
0.024672052	0.700784074	0.106366648	0.096692851	-0.159353927	0.012506988
0.083655094	0.191892321	0.148875557	0.019734337	-0.270069268	1.82E-05
0.375172491	1.32E-09	0.43378199	1.16E-12	-0.327151288	1.61E-07
0.492898676	2.11E-16	0.498274287	8.85E-17	-0.3906423	2.35E-10
0.127188425	0.046732507	0.266358847	2.40E-05	-0.376746851	1.11E-09
-0.075651661	0.23809112	-0.141149117	0.027167272	-0.029037426	0.651068278
0.055576846	0.386415535	0.060370414	0.346718951	-0.19597095	0.002058361
0.271109332	1.69E-05	0.189826186	0.00285227	-0.242465924	0.000126519
0.815392717	1.29E-59	0.605487682	6.70E-26	-0.26617521	2.43E-05
0.039155416	0.541875794	0.073551132	0.251413097	-0.222202134	0.000458093
0.151505714	0.017643	0.376029861	1.20E-09	-0.281657338	7.57E-06
0.555536977	3.06E-21	0.520178133	2.18E-18	-0.307039522	9.57E-07
0.287732737	4.70E-06	0.35827591	7.83E-09	-0.215852436	0.000670088
-0.280646103	8.18E-06	-0.382128594	6.13E-10	0.31389614	5.29E-07
0.291823938	3.39E-06	0.235965148	0.000193526	-0.214535221	0.000724126
0.238322	0.000166113	0.148464662	0.020079768	-0.114407221	0.07386127
0.636175297	3.40E-29	0.59182985	1.52E-24	-0.299709427	1.78E-06
0.46362477	1.85E-14	0.452590817	8.95E-14	-0.2518419	6.71E-05
0.234352019	0.000214664	0.219910221	0.000526155	-0.082188517	0.19982692
0.05948361	0.353861389	-0.031730472	0.621133184	-0.055181845	0.389805151
0.650185982	7.94E-31	0.685799832	2.18E-35	-0.448564109	1.57E-13

0.686763968	1.60E-35	0.481636844	1.24E-15	-0.167985392	0.008421432
-0.011392425	0.859183618	0.117271587	0.066872272	-0.171234744	0.00722242
0.597104409	4.63E-25	0.263148083	3.02E-05	-0.075439851	0.239411559
0.291466242	3.48E-06	0.278029813	1.00E-05	-0.36807048	2.82E-09
-0.048148677	0.453117965	0.052023282	0.417550644	-0.272594275	1.51E-05
0.336849073	6.51E-08	0.427853396	2.52E-12	-0.269715857	1.87E-05
0.423674974	4.31E-12	0.619642408	2.25E-27	-0.411137444	2.07E-11
0.221417102	0.000480429	0.408721919	2.78E-11	-0.238299919	0.000166352
0.468816771	8.62E-15	0.655718322	1.70E-31	-0.341457829	4.18E-08
0.201671808	0.001507755	0.186760674	0.003344334	-0.136960137	0.032120317
0.398874538	9.02E-11	0.295341982	2.54E-06	-0.291536515	3.46E-06
0.2765734	1.12E-05	0.516970289	3.82E-18	-0.327581564	1.55E-07
0.596697039	5.08E-25	0.733769416	1.12E-42	-0.499834353	6.86E-17
0.456808979	4.93E-14	0.632942287	7.87E-29	-0.402671854	5.76E-11
0.609831568	2.41E-26	0.733795391	1.11E-42	-0.510374602	1.18E-17
0.367972741	2.85E-09	0.441717734	4.01E-13	-0.27461294	1.30E-05
0.491766515	2.53E-16	0.382583369	5.83E-10	-0.149074246	0.019569161
-0.436716204	7.86E-13	-0.529454523	4.19E-19	0.172183453	0.006902325
0.410348615	2.28E-11	0.342040094	3.95E-08	-0.147256342	0.021126133
0.397983593	1.00E-10	0.490416492	3.14E-16	-0.170084311	0.007628295
0.438753646	5.98E-13	0.565167809	4.45E-22	-0.55769096	2.00E-21
0.374733614	1.38E-09	0.485057393	7.29E-16	-0.401348263	6.74E-11
0.49971437	7.00E-17	0.514076155	6.29E-18	-0.321785452	2.62E-07
0.094348891	0.14087835	0.140799396	0.027554089	-0.147666363	0.020765896
-0.184926588	0.00367427	0.027479545	0.668648151	-0.274025415	1.36E-05
0.358158039	7.93E-09	0.304869637	1.15E-06	-0.325379885	1.89E-07
0.107632573	0.092765403	0.078660887	0.219881654	-0.215136081	0.000698996
0.286090638	5.35E-06	0.237667151	0.000173341	-0.153370281	0.016279902
0.003256278	0.959557971	-0.208971107	0.000999765	0.272486222	1.52E-05
0.39956209	8.32E-11	0.187635926	0.003196541	-0.283312821	6.65E-06
-0.350331685	1.75E-08	-0.374690806	1.39E-09	0.133473761	0.036810332
-0.080108553	0.211484536	-0.067873019	0.289978658	0.204746092	0.001270321
0.507538004	1.91E-17	0.364265186	4.21E-09	-0.268130169	2.10E-05
0.118808215	0.063351871	0.102607457	0.109138252	-0.238359083	0.000165713
0.31244383	6.01E-07	0.451851028	9.93E-14	-0.385165001	4.37E-10
0.285254676	5.71E-06	0.264100381	2.82E-05	-0.390845679	2.29E-10
0.062394159	0.330763817	0.226432638	0.000353428	-0.246507633	9.66E-05
0.285484706	5.61E-06	0.434566395	1.05E-12	-0.105948977	0.098017264
0.542171392	4.02E-20	0.578149788	2.98E-23	-0.471007094	6.22E-15
0.042927437	0.503627658	0.126893408	0.047248495	-0.215138297	0.000698904

0.312559809	5.95E-07	0.197312523	0.001914406	-0.133229673	0.037159358
0.342408791	3.81E-08	0.216512844	0.000644421	-0.033527478	0.601495505
-0.122229679	0.056058222	0.011503516	0.85782494	-0.309317759	7.87E-07
0.172194255	0.006898754	0.013001895	0.839541774	-0.076812282	0.230946465
0.028391785	0.658331321	0.04857187	0.449152436	-0.127560718	0.046088097
0.444206151	2.86E-13	0.614469648	7.93E-27	-0.425983444	3.21E-12
0.205196941	0.001238541	0.210194561	0.000931964	-0.198178048	0.001826472
0.263146635	3.02E-05	0.142636493	0.025573726	0.001093147	0.986418302
0.313225866	5.61E-07	0.260539204	3.65E-05	-0.14553989	0.022693529
0.359099524	7.20E-09	0.231839814	0.000251915	-0.132998853	0.037491984
0.173140223	0.006592397	0.387564901	3.33E-10	-0.317597397	3.82E-07
0.56675614	3.22E-22	0.414722001	1.33E-11	-0.203827482	0.0013374
0.174492385	0.006175597	0.405241122	4.23E-11	-0.263013668	3.05E-05
0.050927208	0.427442657	0.209875396	0.000949231	-0.257243558	4.61E-05
0.633594077	6.65E-29	0.497182227	1.06E-16	-0.222321255	0.00045479
0.23108729	0.000264196	0.358068916	8.00E-09	-0.323839121	2.18E-07
0.582603663	1.15E-23	0.382448636	5.91E-10	-0.1121852	0.079682309
0.117150365	0.067156669	-0.062013659	0.333727055	0.001385752	0.98278336
0.586944036	4.47E-24	0.517427148	3.53E-18	-0.389839324	2.57E-10
0.009590647	0.881275019	-0.061902691	0.334594441	-0.030076934	0.639443582
0.71686711	6.15E-40	0.459502821	3.35E-14	-0.198959717	0.001750245
-0.32366992	2.21E-07	-0.490844039	2.93E-16	0.414351018	1.39E-11
0.606917295	4.79E-26	0.421786094	5.48E-12	0.013514779	0.833302791
0.216283022	0.000653248	0.205173202	0.001240196	-0.165919112	0.009272995
0.428273194	2.38E-12	0.318735165	3.45E-07	-0.220738574	0.000500542
0.630563073	1.45E-28	0.827701184	6.60E-63	-0.43339722	1.22E-12
0.523353906	1.25E-18	0.502611667	4.34E-17	-0.083758077	0.191343932
0.658534252	7.69E-32	0.576385773	4.34E-23	-0.38036704	7.45E-10
0.627373436	3.27E-28	0.504041266	3.43E-17	-0.130906807	0.040623214
0.694223565	1.45E-36	0.368222348	2.78E-09	-0.203479803	0.001363624
0.511315724	1.01E-17	0.394725048	1.47E-10	-0.220456984	0.000509117
0.701689521	1.22E-37	0.645822555	2.61E-30	-0.361023985	5.90E-09
0.236250545	0.000189995	0.353183266	1.31E-08	-0.488393025	4.32E-16
0.441232909	4.28E-13	0.450082775	1.27E-13	-0.163873332	0.010190478
0.336483176	6.74E-08	0.158488519	0.012999993	-0.223075546	0.000434378
0.297008578	2.22E-06	0.291450163	3.49E-06	-0.07117112	0.267121868
0.35464884	1.13E-08	0.553622296	4.46E-21	-0.206581203	0.00114547
0.138847164	0.029801223	0.281637782	7.58E-06	-0.284385105	6.12E-06
-0.125065808	0.050552167	-0.187786889	0.003171657	-0.116394972	0.068951266
0.389284575	2.74E-10	0.328590877	1.41E-07	-0.315553843	4.57E-07

0.355800665	1.01E-08	0.381351571	6.68E-10	-0.297205339	2.18E-06
0.340224755	4.71E-08	0.149801982	0.018974378	-0.124249862	0.052088044
0.28026716	8.43E-06	0.199813742	0.001670292	-0.350726906	1.68E-08
0.02856972	0.656326427	0.286399659	5.22E-06	-0.032325997	0.614594587
0.626243691	4.34E-28	0.696109257	7.82E-37	-0.431299711	1.61E-12
-0.251633443	6.81E-05	-0.224681099	0.000393728	0.378117172	9.55E-10
0.043202025	0.500899728	-0.027600531	0.667276246	-0.245566471	0.000102879
0.565357141	4.28E-22	0.481701896	1.23E-15	-0.280629799	8.19E-06
0.169294841	0.007918455	0.15069304	0.018267557	-0.269699498	1.87E-05
0.495863981	1.31E-16	0.517505711	3.48E-18	-0.342784913	3.68E-08
0.284928232	5.86E-06	0.32275811	2.40E-07	-0.141072365	0.027251765
0.497402653	1.02E-16	0.404648011	4.54E-11	-0.285474556	5.62E-06
0.695141898	1.07E-36	0.525598968	8.38E-19	-0.312796522	5.82E-07
0.013098961	0.838360228	-0.114002523	0.074894932	-0.081069633	0.20603862
0.38786158	3.22E-10	0.322248624	2.52E-07	-0.435391474	9.37E-13
-0.311404706	6.57E-07	-0.279508637	8.93E-06	0.286723868	5.09E-06
0.220190154	0.000517367	0.156731194	0.014053932	-0.217393561	0.000611604
0.571427169	1.23E-22	0.207933027	0.001060821	-0.092796199	0.147564283
0.26218362	3.24E-05	0.305302241	1.11E-06	-0.319824064	3.13E-07
0.21949873	0.000539324	0.37090278	2.09E-09	-0.256565078	4.83E-05
0.414009314	1.45E-11	0.697621312	4.74E-37	-0.306490366	1.00E-06
0.546597452	1.73E-20	0.499204174	7.60E-17	-0.166689682	0.008946885
0.596939343	4.80E-25	0.461196917	2.63E-14	-0.241425583	0.00013553
0.39828149	9.67E-11	0.601833368	1.57E-25	-0.319657565	3.18E-07
0.458115224	4.09E-14	0.430508192	1.78E-12	-0.265979449	2.46E-05
-0.072039608	0.261313618	0.132121863	0.038778832	-0.565336767	4.30E-22
-0.038781988	0.545739226	0.014321158	0.823514769	-0.249684607	7.78E-05
0.195376298	0.002125271	0.459655818	3.28E-14	-0.23931403	0.000155699
0.782634612	6.12E-52	0.468197581	9.44E-15	-0.238974022	0.000159197
0.197560389	0.001888837	0.204786151	0.001267467	-0.269092535	1.96E-05
0.486309415	5.99E-16	0.162124473	0.011037548	-0.072415067	0.258829708
0.178711384	0.005022435	0.380017214	7.75E-10	-0.313011325	5.72E-07
-0.090579556	0.157523218	-0.182105186	0.004239474	-0.125492654	0.049763843
0.448533793	1.58E-13	0.361322516	5.72E-09	-0.437457986	7.12E-13
0.031029307	0.628869919	0.042077314	0.512122494	-0.116532812	0.068620911
0.556402684	2.58E-21	0.491992445	2.44E-16	-0.342033945	3.95E-08
0.21615595	0.000658177	0.23356591	0.000225728	-0.269992616	1.83E-05
0.307447123	9.24E-07	0.444554477	2.73E-13	-0.28876444	4.33E-06
0.567821509	2.58E-22	0.452972477	8.48E-14	-0.20063066	0.001596953
0.169878756	0.007702917	0.341744835	4.07E-08	-0.215678977	0.000676985

0.053061038	0.408309683	0.126269497	0.048355419	-0.136299068	0.03296831
-0.143765232	0.024418676	-0.431727549	1.52E-12	0.348128829	2.18E-08
-0.284120255	6.25E-06	-0.283119988	6.75E-06	0.273596141	1.40E-05
0.198535078	0.001791286	-0.162410927	0.010894682	0.550308233	8.49E-21
-0.194675417	0.002206675	-0.547436366	1.48E-20	0.515306459	5.09E-18
-0.356549659	9.35E-09	-0.731355306	2.83E-42	0.385781037	4.07E-10
-0.073175492	0.253848915	-0.357249777	8.70E-09	0.270392303	1.78E-05
-0.239102503	0.000157867	-0.594617059	8.12E-25	0.381397759	6.65E-10
-0.311923139	6.28E-07	-0.661472344	3.32E-32	0.285137874	5.77E-06
-0.10101491	0.114776202	-0.38722679	3.46E-10	0.532824213	2.27E-19
-0.375457538	1.28E-09	-0.588814341	2.96E-24	0.540564093	5.43E-20
-0.291282383	3.54E-06	-0.516019216	4.50E-18	0.369577074	2.40E-09
-0.474949905	3.44E-15	-0.827006445	1.03E-62	0.539906205	6.15E-20
-0.219288007	0.000546186	-0.561449604	9.43E-22	0.50450729	3.17E-17
-0.137940152	0.030897414	-0.493842192	1.81E-16	0.342530457	3.77E-08
-0.2730951	1.45E-05	-0.586588075	4.83E-24	0.611867731	1.48E-26
-0.103845979	0.104905952	-0.433913016	1.14E-12	0.6431634	5.35E-30
-0.140409781	0.027990581	-0.569142311	1.97E-22	0.63079924	1.37E-28
-0.157010839	0.01388136	-0.527970276	5.48E-19	0.4911088	2.81E-16
-0.213501403	0.000769325	-0.634367816	5.44E-29	0.401561032	6.57E-11
-0.024028043	0.708233934	-0.365065825	3.87E-09	0.55984313	1.30E-21
-0.000370095	0.995401598	-0.35980646	6.69E-09	0.545445029	2.16E-20
-0.18158795	0.00435121	-0.528443766	5.03E-19	0.42361466	4.34E-12
0.05537998	0.388102657	-0.236721608	0.000184298	0.512114464	8.80E-18
0.062758692	0.327940843	-0.393956028	1.60E-10	0.337229121	6.27E-08
-0.011194085	0.861610408	-0.34521431	2.90E-08	0.51944798	2.48E-18
-0.340914733	4.41E-08	-0.547886351	1.35E-20	0.587224223	4.20E-24
-0.157717627	0.013453457	-0.388126615	3.12E-10	0.626346935	4.23E-28
-0.216399922	0.000648744	-0.535424305	1.41E-19	0.504265126	3.30E-17
0.114330165	0.074057183	-0.325703225	1.84E-07	0.646591535	2.12E-30
-0.319431537	3.24E-07	-0.728605714	8.08E-42	0.546208554	1.87E-20
0.152648655	0.016796136	-0.129826363	0.042324808	0.41025342	2.31E-11
0.378597309	9.06E-10	-0.069643087	0.277553816	0.283706	6.45E-06
-0.157851108	0.013373959	-0.373016513	1.66E-09	0.715616882	9.63E-40
-0.073903916	0.249140284	-0.33491788	7.81E-08	0.492736916	2.17E-16
-0.245999912	9.99E-05	-0.581134046	1.58E-23	0.53525763	1.46E-19
-0.218415831	0.000575454	-0.556915401	2.33E-21	0.443450693	3.17E-13
-0.081411011	0.204128797	-0.522685301	1.41E-18	0.563040945	6.84E-22
0.191922428	0.002554645	-0.109452918	0.08734215	0.529553763	4.12E-19
-0.365332965	3.77E-09	-0.72737052	1.29E-41	0.47003483	7.19E-15

0.169683768	0.007774304	-0.076300317	0.234079176	0.301061705	1.59E-06
-0.369318885	2.47E-09	-0.581450689	1.47E-23	0.603076293	1.18E-25
-0.187125253	0.003282037	-0.580035638	2.00E-23	0.493345688	1.96E-16
-0.27220218	1.56E-05	-0.4136645	1.52E-11	0.605290403	7.02E-26
0.079957627	0.212349063	-0.190703464	0.002724082	0.420273581	6.64E-12
-0.136040246	0.033305466	-0.534090822	1.80E-19	0.450453083	1.21E-13
-0.048551265	0.449345068	-0.570091978	1.62E-22	0.525984139	7.82E-19
-0.357945026	8.11E-09	-0.708370588	1.24E-38	0.488340254	4.36E-16
-0.234506128	0.000212555	-0.55847747	1.71E-21	0.376166149	1.18E-09
0.071747343	0.263258455	-0.477890235	2.20E-15	0.414855906	1.31E-11
-0.234140825	0.000217586	-0.546978026	1.61E-20	0.402269142	6.04E-11
-0.111831838	0.080641279	-0.405783014	3.96E-11	0.479996239	1.60E-15
-0.172869854	0.006678692	-0.477732454	2.26E-15	0.580859323	1.67E-23
-0.072361414	0.25918366	-0.432232513	1.42E-12	0.173465867	0.006489783
-0.096289485	0.132850526	-0.532173389	2.56E-19	0.579143378	2.42E-23
-0.222411986	0.000452288	-0.348355445	2.13E-08	0.466726932	1.17E-14
-0.07736045	0.227625168	-0.308872027	8.18E-07	0.408938514	2.71E-11
-0.150767655	0.018209423	-0.561421009	9.49E-22	0.556738625	2.41E-21
-0.325069471	1.95E-07	-0.760193116	2.07E-47	0.566147484	3.64E-22
-0.03149839	0.623689453	-0.375936592	1.21E-09	0.322119915	2.55E-07
0.019697927	0.759015157	-0.242113554	0.000129506	0.481716845	1.22E-15
-0.230828094	0.000268553	-0.661234015	3.56E-32	0.279441382	8.98E-06
-0.144917745	0.02328594	-0.6218647	1.30E-27	0.414087699	1.44E-11
0.100049495	0.11830328	-0.239178951	0.00015708	0.454552625	6.79E-14
0.061914965	0.33449843	-0.288671424	4.36E-06	0.370622564	2.15E-09
-0.168203801	0.008335614	-0.701474256	1.31E-37	0.542693471	3.64E-20
-0.274277849	1.33E-05	-0.630239129	1.58E-28	0.367194856	3.10E-09
0.041453111	0.518406785	-0.391904575	2.03E-10	0.529872699	3.89E-19
0.057698933	0.36851317	-0.338145088	5.75E-08	0.508293958	1.68E-17
-0.117777663	0.065695592	-0.316437332	4.23E-07	0.446828078	1.99E-13
-0.264838926	2.68E-05	-0.526173111	7.56E-19	0.424975657	3.65E-12
-0.267419878	2.22E-05	-0.769469206	3.20E-49	0.424382067	3.94E-12
-0.084508853	0.187380613	-0.365197993	3.82E-09	0.591036166	1.81E-24
-0.396526305	1.19E-10	-0.774644325	2.88E-50	0.515437594	4.98E-18
0.22183861	0.000468314	-0.165490899	0.009458738	0.574807747	6.05E-23
-0.29979186	1.76E-06	-0.568299286	2.34E-22	0.459737668	3.24E-14
-0.022497583	0.726048766	-0.447258029	1.88E-13	0.349125671	1.97E-08
-0.241503684	0.000134834	-0.42220645	5.20E-12	0.196456318	0.002005177
-0.346007767	2.68E-08	-0.736120047	4.47E-43	0.503238317	3.91E-17
-0.161624236	0.011291007	-0.369574233	2.40E-09	0.433519616	1.20E-12

-0.217050078	0.000624215	-0.416993547	1.00E-11	0.439874496	5.14E-13
-0.108830678	0.089166609	-0.384769257	4.56E-10	0.57778323	3.23E-23
-0.069295106	0.279967533	-0.573528127	7.92E-23	0.428134219	2.43E-12
-0.113123954	0.077179363	-0.504915907	2.96E-17	0.580549458	1.79E-23
0.023468068	0.714734358	-0.392701062	1.85E-10	0.464499931	1.63E-14
-0.254462841	5.60E-05	-0.519185582	2.60E-18	0.477301284	2.41E-15
-0.083486291	0.192793685	-0.558257852	1.79E-21	0.256115578	4.99E-05
-0.263263913	3.00E-05	-0.519247203	2.57E-18	0.536163159	1.23E-19
-0.245401429	0.000104025	-0.613809246	9.29E-27	0.380848456	7.07E-10
-0.119416896	0.062000142	-0.577058866	3.76E-23	0.398722918	9.18E-11
0.007975289	0.901159625	-0.325831563	1.82E-07	0.469938277	7.30E-15
-0.269343049	1.92E-05	-0.422204248	5.20E-12	0.439416851	5.47E-13
-0.219448295	0.000540959	-0.294904277	2.64E-06	0.49939098	7.38E-17
0.057765229	0.36796227	-0.211673176	0.000855681	0.428912458	2.19E-12
-0.333616211	8.83E-08	-0.657392253	1.06E-31	0.608269748	3.48E-26
-0.116851992	0.0678609	-0.46778558	1.00E-14	0.354858573	1.11E-08
-0.265154795	2.62E-05	-0.670634577	2.28E-33	0.344857272	3.00E-08
-0.221364271	0.000481967	-0.49835659	8.73E-17	0.454835209	6.52E-14
-0.250877627	7.17E-05	-0.631151271	1.25E-28	0.452163186	9.50E-14
-0.061166488	0.340385534	-0.445198621	2.50E-13	0.611210519	1.73E-26
-0.337862172	5.91E-08	-0.510095694	1.24E-17	0.517760679	3.33E-18
-0.0506423	0.430035982	-0.327918334	1.50E-07	0.3947488	1.46E-10
0.068098934	0.28837247	-0.356298029	9.59E-09	0.378382648	9.27E-10
-0.026446986	0.680401104	-0.298813347	1.91E-06	0.601508885	1.69E-25
-0.183793755	0.003892473	-0.533433274	2.04E-19	0.361317007	5.73E-09
-0.16194415	0.011128329	-0.485384045	6.93E-16	0.394138982	1.57E-10
0.165778454	0.009333648	-0.174774911	0.006091548	0.389759691	2.59E-10
0.106708162	0.095620532	-0.330238634	1.21E-07	0.439116341	5.70E-13
-0.174463261	0.00618432	-0.527361873	6.11E-19	0.430732454	1.73E-12
0.006985172	0.91337904	-0.314537244	5.00E-07	0.674833535	6.47E-34
-0.389620452	2.64E-10	-0.742516484	3.51E-44	0.630210716	1.59E-28
0.092643356	0.148235241	-0.293855449	2.87E-06	0.442260508	3.73E-13
-0.083713493	0.191581199	-0.324815118	1.99E-07	0.408886828	2.72E-11
-0.258950736	4.08E-05	-0.689710802	6.27E-36	0.483523654	9.26E-16
-0.088123601	0.16913924	-0.489204956	3.80E-16	0.469506547	7.78E-15
-0.018957948	0.767804575	-0.358536423	7.63E-09	0.47376943	4.11E-15
-0.349616823	1.88E-08	-0.649260434	1.02E-30	0.521170923	1.84E-18
-0.28469089	5.97E-06	-0.551420341	6.85E-21	0.490958983	2.88E-16
-0.305179086	1.12E-06	-0.717220726	5.42E-40	0.614119785	8.62E-27
-0.121446265	0.057663765	-0.407380035	3.27E-11	0.451674602	1.02E-13

-0.19183147	0.00256695	-0.574048913	7.10E-23	0.605747847	6.31E-26
-0.203648986	0.001350805	-0.718813401	3.04E-40	0.426765527	2.90E-12
-0.082527908	0.19796987	-0.396375745	1.21E-10	0.369857683	2.33E-09
-0.178611152	0.005047416	-0.472686168	4.84E-15	0.505061683	2.89E-17
-0.135195638	0.034426169	-0.413987056	1.46E-11	0.611864554	1.48E-26
-0.159201187	0.012592786	-0.599054784	2.97E-25	0.204176965	0.001311506
-0.084426461	0.187812589	-0.330585505	1.17E-07	0.402644849	5.77E-11
-0.287191738	4.90E-06	-0.726130498	2.05E-41	0.400042816	7.86E-11
-0.056615394	0.377589132	-0.293981895	2.84E-06	0.502435881	4.47E-17
-0.150896621	0.018109322	-0.353193173	1.31E-08	0.445580114	2.37E-13
-0.306752168	9.81E-07	-0.696045639	7.98E-37	0.381559294	6.53E-10
-0.071192288	0.266979257	-0.428822787	2.22E-12	0.518440743	2.96E-18
-0.170397682	0.00751577	-0.368617306	2.66E-09	0.52189918	1.61E-18
-0.066662421	0.298687447	-0.332637962	9.68E-08	0.494700567	1.58E-16
-0.239789181	0.00015093	-0.43965587	5.30E-13	0.266611577	2.35E-05
-0.194632094	0.002211799	-0.570236493	1.57E-22	0.474876537	3.48E-15
0.144116317	0.024068693	-0.147391437	0.021006848	0.619694661	2.22E-27
0.04717565	0.462309782	-0.301839223	1.49E-06	0.458325255	3.97E-14
-0.132787436	0.037798856	-0.338601149	5.50E-08	0.44207815	3.82E-13
0.023920951	0.709475494	-0.390955274	2.26E-10	0.574264398	6.78E-23
0.392661631	1.86E-10	-0.103493217	0.106098011	0.363278434	4.67E-09
-0.292286002	3.26E-06	-0.773221628	5.62E-50	0.515801964	4.68E-18
-0.325555415	1.86E-07	-0.544119707	2.78E-20	0.582126976	1.27E-23
-0.139990346	0.028467087	-0.34977383	1.85E-08	0.27927457	9.10E-06
-0.069910366	0.275709467	-0.350429568	1.73E-08	0.484857391	7.52E-16
0.191478581	0.002615198	-0.277848741	1.01E-05	0.394501658	1.50E-10
-0.259974632	3.80E-05	-0.436865105	7.70E-13	0.530394444	3.54E-19
0.160630327	0.011809883	-0.258751775	4.14E-05	0.436284967	8.32E-13
-0.440798854	4.54E-13	-0.7144751	1.45E-39	0.405072469	4.32E-11
-0.120220042	0.06025294	-0.470376055	6.84E-15	0.511082986	1.05E-17
-0.101520188	0.112963268	-0.436119885	8.51E-13	0.350019702	1.80E-08
0.008550181	0.894075136	-0.383862039	5.05E-10	0.242160871	0.000129102
-0.397311027	1.08E-10	-0.632790893	8.18E-29	0.421824374	5.45E-12
-0.210598955	0.000910502	-0.552755843	5.28E-21	0.407720289	3.14E-11
-0.221674751	0.000472989	-0.610119599	2.25E-26	0.497211364	1.05E-16
-0.051143146	0.425483167	-0.489040867	3.90E-16	0.276737503	1.10E-05
0.030527317	0.634433984	-0.424841162	3.71E-12	0.31246586	5.99E-07
-0.209155838	0.000989245	-0.503354286	3.84E-17	0.620763656	1.70E-27
-0.235309513	0.000201871	-0.549727667	9.50E-21	0.528852921	4.68E-19
-0.272853584	1.48E-05	-0.495787156	1.33E-16	0.581913263	1.33E-23

-0.1194973	0.061823372	-0.397330148	1.08E-10	0.67814216	2.36E-34
0.082263115	0.199417661	-0.231478662	0.000257741	0.466143295	1.28E-14
-0.135485253	0.034038327	-0.333713873	8.75E-08	0.365522321	3.69E-09
-0.030075502	0.639459534	-0.203199763	0.001385089	0.539096526	7.15E-20
-0.100792368	0.115581862	-0.450241286	1.24E-13	0.471959476	5.40E-15
-0.182637426	0.004127202	-0.450162213	1.26E-13	0.302987939	1.35E-06
-0.308862384	8.19E-07	-0.650306682	7.68E-31	0.506589652	2.24E-17
-0.294580906	2.71E-06	-0.611899645	1.47E-26	0.173595944	0.006449196
-0.158141377	0.013202503	-0.451036742	1.11E-13	0.415560752	1.20E-11
-0.424230155	4.01E-12	-0.707426915	1.72E-38	0.38367905	5.16E-10
-0.208382938	0.00103395	-0.651514829	5.50E-31	0.528889993	4.64E-19
0.128948167	0.043751666	-0.319869967	3.12E-07	0.355220085	1.07E-08
-0.278316616	9.79E-06	-0.717175487	5.50E-40	0.594220879	8.88E-25
-0.091242407	0.154493521	-0.331466313	1.08E-07	0.364583213	4.08E-09
-0.227345163	0.000333985	-0.562042034	8.37E-22	0.23870498	0.000162018
-0.272163175	1.56E-05	0.221483074	0.000478513	-0.324038449	2.14E-07
-0.247867276	8.81E-05	-0.668813623	3.91E-33	0.457105407	4.72E-14
0.128459165	0.044563527	-0.040098185	0.532182811	0.253134355	6.14E-05
-0.027963242	0.663169959	-0.29137257	3.51E-06	0.231609317	0.000255619
0.295862101	2.44E-06	-0.017301742	0.787583173	0.248324264	8.54E-05
-0.118430826	0.064202047	-0.298578031	1.95E-06	0.327539756	1.55E-07
0.048766788	0.447332568	-0.178558742	0.005060523	0.319029658	3.36E-07
-0.227205899	0.000336886	-0.573795158	7.49E-23	0.516771902	3.95E-18
0.01181415	0.85402803	-0.308819995	8.22E-07	0.417833944	9.02E-12
-0.23722519	0.000178385	-0.65214316	4.62E-31	0.378355404	9.30E-10
-0.298114185	2.03E-06	-0.308351622	8.56E-07	0.184464889	0.00376183
-0.167132528	0.008764112	-0.56552805	4.13E-22	0.450342041	1.23E-13
-0.31007949	7.37E-07	-0.620230741	1.94E-27	0.281940366	7.40E-06
-0.089698691	0.161618344	-0.495947948	1.29E-16	0.480595399	1.46E-15
-0.17819091	0.005153375	-0.613253794	1.06E-26	0.253584614	5.95E-05
-0.275785335	1.19E-05	-0.392335249	1.93E-10	0.201750317	0.001501217
-0.354905799	1.10E-08	-0.613010118	1.13E-26	0.3277793	1.52E-07
-0.346510265	2.55E-08	-0.50267929	4.29E-17	0.316334597	4.27E-07
-0.115714718	0.070600684	-0.357034768	8.90E-09	0.404077824	4.87E-11
-0.143948546	0.024235394	-0.413353786	1.58E-11	0.495081379	1.49E-16
0.205491356	0.001218184	-0.122291496	0.055933123	0.433550019	1.20E-12
-0.254488843	5.59E-05	-0.571981703	1.09E-22	0.308627582	8.36E-07
-0.11090268	0.083207373	-0.510200261	1.22E-17	0.441005226	4.42E-13
-0.036589474	0.56869271	-0.346878413	2.46E-08	0.149547168	0.019180827
-0.282184668	7.26E-06	-0.616692059	4.62E-27	0.343650187	3.38E-08

0.085494702	0.182268276	-0.327438999	1.57E-07	0.184547576	0.003746012
-0.292668515	3.16E-06	-0.486372277	5.94E-16	0.491191949	2.77E-16
-0.166437443	0.009052496	-0.499547827	7.19E-17	0.307657454	9.08E-07
-0.11994342	0.06085008	-0.398631022	9.28E-11	0.360995507	5.92E-09
-0.238324425	0.000166087	-0.599121185	2.92E-25	0.437760673	6.83E-13
-0.032696483	0.610542073	-0.343848214	3.32E-08	0.385407182	4.25E-10
-0.112187583	0.079675873	-0.537902072	8.93E-20	0.297562691	2.12E-06
-0.172416338	0.006825711	-0.555039008	3.37E-21	0.197463711	0.001898772
-0.134138159	0.035874325	-0.490738246	2.98E-16	0.329122163	1.34E-07
-0.182710291	0.004112042	-0.555582719	3.03E-21	0.590120233	2.22E-24
-0.457053663	4.76E-14	-0.712956712	2.48E-39	0.373681605	1.55E-09
-0.086361224	0.177860271	-0.384914895	4.49E-10	0.320366433	2.98E-07
0.076759331	0.231269091	-0.096975766	0.130097367	0.259853982	3.83E-05
-0.304460203	1.19E-06	-0.698599738	3.42E-37	0.412597065	1.73E-11
-0.236273843	0.000189709	-0.419258259	7.55E-12	0.206572201	0.001146053
-0.238571047	0.000163439	-0.650988026	6.36E-31	0.437276657	7.29E-13
-0.18019647	0.004665093	-0.341284735	4.25E-08	0.5533128	4.73E-21
-0.241901879	0.000131333	-0.640101998	1.21E-29	0.307594324	9.13E-07
-0.05705611	0.373881198	-0.363517016	4.56E-09	0.193095622	0.002400674
-0.157479861	0.013596094	-0.425263391	3.52E-12	0.370427139	2.20E-09
-0.263473617	2.95E-05	-0.579548525	2.22E-23	0.115106681	0.072102084
-0.243407237	0.000118852	-0.574727745	6.15E-23	0.297611775	2.11E-06
-0.239283608	0.000156009	-0.4732539	4.45E-15	0.124980304	0.050711324
-0.064853793	0.312018454	-0.240756616	0.000141639	0.207426035	0.00109187
0.200193558	0.001635819	-0.316192618	4.32E-07	0.578342553	2.86E-23
-0.299182241	1.85E-06	-0.77365853	4.58E-50	0.54662065	1.73E-20
-0.210621189	0.000909335	-0.627999037	2.79E-28	0.40821924	2.95E-11
-0.188903101	0.002993039	-0.626875549	3.70E-28	0.283813863	6.40E-06
-0.130994356	0.040487893	-0.686800158	1.59E-35	0.465551804	1.39E-14
-0.011955818	0.852297508	-0.321774213	2.63E-07	0.338207334	5.72E-08
-0.338767475	5.42E-08	-0.637285068	2.54E-29	0.241740417	0.000132742
0.036568696	0.568912414	-0.381983306	6.23E-10	0.434512537	1.05E-12
-0.351405763	1.57E-08	-0.804039643	8.61E-57	0.472829989	4.74E-15
-0.112139352	0.079806212	-0.52086678	1.94E-18	0.29819678	2.01E-06
0.071529979	0.264711295	-0.255259317	5.30E-05	0.301702111	1.50E-06
-0.207199636	0.001106003	-0.59789939	3.86E-25	0.553498939	4.56E-21
-0.067587963	0.292013838	-0.312075963	6.20E-07	0.294660594	2.69E-06
-0.198865704	0.001759256	-0.424528553	3.86E-12	0.323475928	2.25E-07
0.011647032	0.856070325	-0.294515184	2.72E-06	0.229647136	0.000289267
-0.184269649	0.003799421	-0.526283277	7.42E-19	0.442094357	3.81E-13

-0.029971313	0.64062078	-0.165756751	0.009343037	0.323869519	2.17E-07
-0.400720708	7.26E-11	-0.693326666	1.95E-36	0.272481219	1.52E-05
-0.143629601	0.02455505	-0.574817897	6.04E-23	0.23859391	0.000163196
0.079891471	0.212728813	-0.335315283	7.52E-08	0.424520778	3.87E-12
-0.418887277	7.91E-12	-0.344203201	3.20E-08	0.142337417	0.025887534
0.048071093	0.453847088	-0.136190123	0.033109872	0.188777036	0.003012745
-0.358341367	7.78E-09	-0.664251621	1.49E-32	0.308650053	8.34E-07
0.111050027	0.08279611	-0.18734412	0.003245144	0.418752879	8.04E-12
-0.099371995	0.120828525	-0.540960031	5.04E-20	0.312469371	5.99E-07
-0.109946596	0.085916045	-0.553220316	4.82E-21	0.498179162	8.99E-17
-0.14881663	0.019783557	-0.406360757	3.70E-11	0.392377484	1.92E-10
-0.005071084	0.937056799	-0.293720794	2.90E-06	0.314323146	5.10E-07
0.004069041	0.949475765	-0.163654761	0.0102931	0.287999929	4.60E-06
-0.425014473	3.63E-12	-0.724975386	3.16E-41	0.458492584	3.87E-14
-0.476358259	2.78E-15	-0.745562574	1.02E-44	0.456316449	5.29E-14
-0.057391784	0.371072105	-0.36293345	4.84E-09	0.479011723	1.86E-15
-0.006464478	0.919813479	-0.344873007	3.00E-08	0.284345286	6.14E-06
-0.436332013	8.27E-13	-0.850727423	7.92E-70	0.442408193	3.65E-13
-0.110617555	0.084007867	-0.415158241	1.26E-11	0.231187398	0.00026253
-0.17846791	0.005083311	-0.504864406	2.99E-17	0.3632438	4.69E-09
-0.141929663	0.0263207	-0.139097652	0.029504411	0.283071314	6.78E-06
-0.169953019	0.007675883	-0.331843881	1.04E-07	0.60364957	1.03E-25
-0.229798606	0.00028653	-0.292254705	3.27E-06	0.435309375	9.47E-13
-0.225742807	0.000368819	-0.180626875	0.004565895	-0.062554878	0.329517277
0.061274859	0.339529083	-0.20928686	0.000981846	0.387288533	3.44E-10
0.128023415	0.045297584	-0.181864591	0.004291122	0.1101008	0.085474446
-0.100432598	0.11689366	-0.259713649	3.87E-05	0.17187273	0.00700574
-0.218069706	0.000587467	-0.272786671	1.49E-05	0.370210835	2.25E-09
0.244243929	0.000112405	0.257055835	4.67E-05	-0.097353019	0.128602796
-0.048309057	0.451612818	-0.177786336	0.005257267	0.127835418	0.045617393
-0.039031204	0.543159371	-0.238544613	0.000163721	0.300460058	1.67E-06
-0.129023335	0.043627976	-0.206906106	0.001124577	0.34915426	1.97E-08
-0.064293124	0.316228967	-0.188575868	0.003044434	0.588538686	3.15E-24
-0.066693366	0.298462698	-0.178212661	0.005147842	0.504809917	3.02E-17
0.106984515	0.094759755	-0.079324574	0.21600291	0.337965734	5.85E-08
0.359561035	6.87E-09	-0.156778156	0.01402482	0.445441359	2.41E-13
-0.048584732	0.449032225	-0.232370367	0.000243579	0.411300466	2.03E-11
-0.139345106	0.029213679	-0.281397487	7.72E-06	0.45779821	4.28E-14
-0.057486489	0.370281934	0.07403443	0.248303078	0.047058549	0.463422913
-0.220015363	0.000522838	-0.265429191	2.56E-05	0.453854548	7.49E-14

-0.28873981	4.34E-06	-0.150773951	0.018204525	0.036068544	0.574212996
-0.182758799	0.004101978	-0.193604386	0.002336567	0.420471309	6.47E-12
0.004569004	0.943277695	-0.277590706	1.03E-05	0.663878317	1.66E-32
-0.233845582	0.000221732	-0.218257141	0.000580933	0.545025718	2.34E-20
-0.212904053	0.000796614	-0.289633375	4.04E-06	0.585688955	5.88E-24
-0.055720941	0.385183477	-0.211177698	0.000880578	0.584248612	8.05E-24
0.372090017	1.84E-09	0.111000283	0.082934768	0.114612245	0.073342045
-0.096600609	0.131596883	-0.084955193	0.185053136	0.207384341	0.00109446
-0.169593975	0.007807375	-0.29763013	2.11E-06	0.492969751	2.09E-16
-0.090518121	0.157806266	-0.284570138	6.03E-06	0.251882814	6.69E-05
-0.072132063	0.260700458	-0.230241812	0.000278657	0.488578267	4.19E-16
-0.055805871	0.384458419	-0.315225068	4.71E-07	0.564009492	5.63E-22
0.015597753	0.808075376	-0.044129716	0.49174133	0.327330163	1.58E-07
-0.299961981	1.74E-06	-0.164347635	0.009970895	-0.247727646	8.89E-05
-0.088685279	0.166427911	-0.059520215	0.353564751	0.024997472	0.697030431
0.064688813	0.313253595	-0.063681687	0.320862779	0.474196226	3.86E-15
-0.220496061	0.000507919	-0.204800813	0.001266424	0.144177703	0.024007946
-0.238655928	0.000162537	-0.268997697	1.97E-05	0.582829715	1.09E-23
-0.074340738	0.246345893	0.149388811	0.019310108	-0.535572283	1.37E-19
-0.165492591	0.009457997	-0.268770179	2.01E-05	0.549675543	9.60E-21
-1.28E-05	0.999840535	-0.1985864	0.00178628	0.533157211	2.14E-19
-0.157295929	0.013707345	-0.302309205	1.43E-06	0.647414072	1.70E-30
-0.123268599	0.053986275	-0.312827739	5.81E-07	0.568635384	2.19E-22
-0.154438913	0.015540889	-0.243508104	0.000118057	0.37711338	1.07E-09
-0.136426145	0.032803837	-0.19989688	0.00166269	0.393978731	1.60E-10
0.195054281	0.002162325	0.289364387	4.12E-06	-0.2765549	1.12E-05
-0.191411616	0.002624447	-0.246646269	9.57E-05	0.480408144	1.50E-15
-0.299212572	1.85E-06	-0.293679703	2.91E-06	0.41683322	1.02E-11
-0.178498688	0.005075579	-0.230564991	0.000273044	0.494056575	1.75E-16
-0.185257742	0.003612605	-0.238200406	0.000167434	0.149000767	0.019630106
0.262074448	3.27E-05	0.082605193	0.197548749	-0.451512445	1.04E-13
-0.198781506	0.001767363	-0.330142138	1.22E-07	0.570439284	1.51E-22
-0.131347239	0.039946302	0.104658351	0.102200958	-0.454637818	6.71E-14
0.249587685	7.84E-05	0.176809734	0.005515839	-0.400310849	7.62E-11
0.109764193	0.086440768	0.201791097	0.001497832	-0.022230307	0.729175389
-0.279190984	9.16E-06	-0.207824903	0.001067374	0.173680836	0.006422829
0.069009928	0.281956167	0.252253565	6.53E-05	-0.356424742	9.47E-09
-0.040398005	0.529118628	-0.268433998	2.06E-05	0.574789333	6.08E-23
-0.082241534	0.199535998	-0.269531823	1.90E-05	0.440047068	5.03E-13
-0.054794861	0.393143337	-0.245053877	0.000106477	0.343026315	3.59E-08

0.014006968	0.827325321	-0.264547237	2.73E-05	0.37257563	1.74E-09
-0.335798329	7.19E-08	-0.285469537	5.62E-06	0.242470232	0.000126483
0.261040454	3.52E-05	0.297308411	2.17E-06	0.052393831	0.414237019
-0.186698115	0.003355131	-0.310289971	7.24E-07	0.521651374	1.69E-18
-0.039322639	0.540150143	-0.183086746	0.004034517	0.668901715	3.81E-33
0.020031416	0.755064013	0.15499833	0.015165852	-0.054496687	0.395727143
-0.197150095	0.001931332	-0.175404527	0.005907915	0.242886458	0.000123038
0.223715182	0.000417738	0.293910854	2.86E-06	-0.259363695	3.97E-05
0.252829616	6.27E-05	-0.033396295	0.602919619	0.30717039	9.47E-07
0.359992085	6.57E-09	0.1673976	0.008656303	-0.32905454	1.35E-07
0.903236415	2.98E-91	0.71994271	2.02E-40	-0.115570757	0.070953837
0.899305663	2.94E-89	0.693049779	2.13E-36	-0.251250032	6.99E-05
0.808678263	6.36E-58	0.490068415	3.31E-16	-0.261670633	3.36E-05
0.855311899	2.41E-71	0.737350211	2.76E-43	-0.239311052	0.000155729
0.889080953	1.96E-84	0.639500045	1.42E-29	-0.211316862	0.000873518
0.861288618	2.10E-73	0.70649175	2.37E-38	-0.133108439	0.037333751
0.877687233	1.37E-79	0.710524975	5.84E-39	-0.157843074	0.013378732
0.428973765	2.18E-12	0.191973265	0.002547791	-0.300496497	1.66E-06
0.711771517	3.77E-39	0.425197644	3.55E-12	-0.040067014	0.532501887
0.905785231	1.37E-92	0.840192688	1.59E-66	-0.30162152	1.51E-06
0.666475565	7.79E-33	0.437876327	6.73E-13	-0.458821475	3.70E-14
0.835638869	3.59E-65	0.736664263	3.61E-43	-0.223850076	0.000414305
0.941494566	9.07E-117	0.578498638	2.77E-23	-0.157104889	0.01382374
0.441874503	3.93E-13	0.269513517	1.90E-05	-0.182775585	0.004098501
-0.466936582	1.14E-14	-0.273955458	1.36E-05	-0.16238055	0.010909754
0.863675171	2.97E-74	0.658038625	8.85E-32	-0.123363912	0.053799413
0.925248996	2.83E-104	0.605442266	6.77E-26	-0.148831216	0.019771363
0.586139728	5.33E-24	0.352390136	1.42E-08	-0.342886582	3.64E-08
0.792420334	4.36E-54	0.619717853	2.21E-27	-0.256149216	4.98E-05
0.777313535	8.09E-51	0.441289308	4.25E-13	-0.256837911	4.74E-05
0.769026524	3.93E-49	0.612480276	1.28E-26	-0.350259307	1.76E-08
0.832760559	2.46E-64	0.543804108	2.95E-20	-0.164701369	0.009809868
0.385917115	4.01E-10	-0.021170242	0.741619829	0.438353405	6.31E-13
0.907984015	8.90E-94	0.617174593	4.11E-27	-0.088178985	0.168870434
0.889230214	1.68E-84	0.560263535	1.20E-21	-0.042663253	0.506259567
0.74730426	4.98E-45	0.474107784	3.91E-15	-0.326599803	1.69E-07
0.778782729	4.00E-51	0.279579784	8.89E-06	-0.06296387	0.326358776
0.882505556	1.41E-81	0.570356988	1.53E-22	-0.072407821	0.258877486
0.775071167	2.35E-50	0.492821766	2.14E-16	-0.131713535	0.039390599
0.926892935	2.10E-105	0.822398381	1.86E-61	-0.267874852	2.14E-05

0.872534363	1.49E-77	0.512217546	8.65E-18	-0.151091901	0.017958661
0.827707418	6.58E-63	0.63999667	1.24E-29	-0.215733365	0.000674815
0.832979737	2.13E-64	0.639505165	1.42E-29	-0.027366261	0.669933721
0.850221347	1.16E-69	0.659392705	6.02E-32	-0.072892306	0.255695977
0.593612379	1.02E-24	0.266719872	2.33E-05	0.096247729	0.133019475
0.870574964	8.39E-77	0.790182185	1.38E-53	-0.227087557	0.00033937
0.943941044	5.87E-119	0.776981569	9.48E-51	-0.306295428	1.02E-06
0.776696344	1.09E-50	0.368577808	2.67E-09	-0.205669288	0.00120603
0.834631761	7.07E-65	0.72197502	9.59E-41	-0.220095795	0.000520314
0.814793239	1.84E-59	0.545213647	2.26E-20	-0.115484911	0.071165114
0.800468445	6.10E-56	0.680694925	1.08E-34	-0.210528329	0.000914217
0.868491628	5.11E-76	0.662914096	2.19E-32	-0.304871964	1.15E-06
0.801731706	3.06E-56	0.476451016	2.74E-15	-0.274850188	1.27E-05
0.848803898	3.32E-69	0.568881722	2.08E-22	-0.171741779	0.007049736
0.743444398	2.41E-44	0.588855718	2.93E-24	-0.318557757	3.51E-07
0.737084117	3.06E-43	0.362501655	5.06E-09	-0.04010098	0.5321542
0.827538274	7.33E-63	0.561204752	9.91E-22	-0.220904366	0.000495557
0.810198391	2.67E-58	0.500067228	6.60E-17	-0.066880706	0.297104457
0.860949223	2.76E-73	0.619849937	2.13E-27	-0.189114875	0.002960198
0.763389748	5.02E-48	0.510783185	1.10E-17	-0.200993179	0.001565361
0.838177325	6.40E-66	0.613004431	1.13E-26	-0.069562397	0.278112254
0.789553385	1.91E-53	0.640382354	1.12E-29	-0.227379899	0.000333264
0.745568382	1.02E-44	0.382149682	6.12E-10	0.085987103	0.179753657
0.838577084	4.86E-66	0.60802507	3.69E-26	-0.167165813	0.008750509
0.883439966	5.68E-82	0.721605169	1.10E-40	-0.204986213	0.001253303
0.860736446	3.28E-73	0.595177767	7.16E-25	-0.268060061	2.11E-05
0.811154031	1.54E-58	0.476676468	2.65E-15	0.005658331	0.929785541
-0.426753131	2.90E-12	-0.237415171	0.0001762	0.226159854	0.000359441
0.79200274	5.41E-54	0.49816217	9.01E-17	-0.106330109	0.096808142
0.852304823	2.41E-70	0.63539901	4.16E-29	-0.044518663	0.487928287
0.829664303	1.87E-63	0.759116736	3.31E-47	-0.341078423	4.34E-08
0.757888275	5.65E-47	0.452364692	9.24E-14	-0.065433892	0.307700863
0.46038801	2.95E-14	0.177887903	0.00523101	-0.219830727	0.000528676
0.778343743	4.94E-51	0.38305519	5.53E-10	0.102934591	0.108007534
0.74276945	3.17E-44	0.505567167	2.66E-17	-0.170005003	0.007657009
-0.48116268	1.33E-15	-0.220946045	0.000494311	0.053915317	0.400794181
0.642225427	6.87E-30	0.862724845	6.50E-74	-0.284665821	5.98E-06
0.491825433	2.50E-16	0.71128238	4.48E-39	-0.407178193	3.35E-11
0.377487432	1.02E-09	0.527388648	6.08E-19	-0.215707897	0.00067583
0.467911709	9.85E-15	0.664774247	1.28E-32	-0.083063857	0.195062922

0.693895501	1.62E-36	0.798893076	1.43E-55	-0.435683995	9.01E-13
0.497253173	1.05E-16	0.832459463	3.00E-64	-0.276634741	1.11E-05
-0.538261027	8.35E-20	-0.639420194	1.45E-29	0.404959983	4.38E-11
0.510901621	1.08E-17	0.719484178	2.39E-40	-0.1944673	0.002231388
0.543633157	3.05E-20	0.826703649	1.25E-62	-0.190509778	0.002751925
-0.211384542	0.000870104	-0.370646442	2.14E-09	0.416182638	1.11E-11
0.444449821	2.76E-13	0.424412099	3.92E-12	-0.217608557	0.000603832
0.646293314	2.30E-30	0.751455152	8.83E-46	-0.345962991	2.70E-08
0.290809501	3.67E-06	0.769615507	3.00E-49	-0.430150404	1.87E-12
0.426881485	2.85E-12	0.676006746	4.53E-34	-0.127619404	0.045987197
0.575746059	4.97E-23	0.707832553	1.49E-38	-0.298475287	1.97E-06
0.413751521	1.50E-11	0.541051229	4.96E-20	-0.176508067	0.005597985
0.725080222	3.04E-41	0.693960301	1.58E-36	-0.213566153	0.000766419
-0.313573959	5.44E-07	-0.489443132	3.66E-16	0.692912729	2.23E-36
0.583888606	8.70E-24	0.667547928	5.68E-33	-0.349496622	1.90E-08
0.570473671	1.50E-22	0.804526341	6.57E-57	-0.382337006	5.99E-10
-0.452515117	9.04E-14	-0.585173871	6.58E-24	0.292484812	3.21E-06
-0.339560917	5.02E-08	-0.351479128	1.56E-08	0.236120965	0.000191591
0.746160916	7.97E-45	0.6646007	1.35E-32	-0.349656292	1.87E-08
-0.456228395	5.35E-14	-0.621893805	1.29E-27	0.277835321	1.02E-05
-0.192542981	0.00247212	-0.555290567	3.21E-21	0.714437524	1.47E-39
-0.288788695	4.32E-06	-0.412681203	1.71E-11	0.606255022	5.60E-26
0.387513946	3.35E-10	0.62810022	2.72E-28	-0.326028551	1.79E-07
0.615616769	6.00E-27	0.497709459	9.70E-17	-0.120507871	0.059636742
0.375416945	1.28E-09	0.670668417	2.26E-33	-0.139094791	0.029507786
-0.467997066	9.73E-15	-0.593692485	1.00E-24	0.348063565	2.19E-08
0.573629472	7.75E-23	0.882821939	1.04E-81	-0.346324187	2.60E-08
0.651487308	5.54E-31	0.677618396	2.77E-34	-0.287609645	4.74E-06
-0.309692029	7.62E-07	-0.609424396	2.65E-26	0.443990829	2.94E-13
0.451554668	1.03E-13	0.549047578	1.08E-20	-0.251983215	6.65E-05
0.564160642	5.46E-22	0.864669923	1.30E-74	-0.324344861	2.08E-07
0.795257665	9.89E-55	0.808396289	7.47E-58	-0.269217874	1.94E-05
-0.265570531	2.54E-05	-0.39119661	2.20E-10	0.201335244	0.001536077
0.336112154	6.98E-08	0.710763931	5.37E-39	-0.1314576	0.039778184
-0.289195559	4.18E-06	-0.608678129	3.16E-26	0.361508274	5.61E-09
0.497062557	1.08E-16	0.581921338	1.33E-23	-0.245632497	0.000102423
0.508906448	1.52E-17	0.683119512	5.06E-35	-0.22566392	0.000370618
0.492391663	2.29E-16	0.86722355	1.51E-75	-0.348556433	2.09E-08
0.342653172	3.72E-08	0.517969284	3.21E-18	-0.142825193	0.025377418
0.711603764	4.00E-39	0.701480324	1.30E-37	-0.111116577	0.082610896

-0.403233971	5.38E-11	-0.431039325	1.66E-12	0.388719743	2.92E-10
-0.270916058	1.71E-05	-0.466334533	1.24E-14	0.542592798	3.71E-20
-0.207999484	0.001056812	-0.492266169	2.33E-16	0.379456345	8.24E-10
0.641317115	8.76E-30	0.797976209	2.34E-55	-0.136529276	0.032670872
-0.329564549	1.29E-07	-0.413336872	1.58E-11	-0.216749096	0.000635462
-0.034931557	0.586348397	-0.411118827	2.07E-11	0.259064013	4.05E-05
-0.435953358	8.70E-13	-0.680558106	1.12E-34	0.344184913	3.21E-08
0.654003404	2.76E-31	0.892801983	3.92E-86	-0.323217122	2.31E-07
0.13702428	0.032039035	-0.283317141	6.65E-06	0.237777077	0.000172108
-0.407497124	3.22E-11	-0.575065719	5.73E-23	0.673286639	1.03E-33
0.52936131	4.27E-19	0.655935166	1.60E-31	-0.368749788	2.63E-09
0.358826358	7.40E-09	0.748311367	3.28E-45	-0.242407895	0.000127007
0.585391983	6.27E-24	0.628356032	2.55E-28	-0.288567544	4.40E-06
-0.25902879	4.06E-05	-0.481226938	1.32E-15	0.576382349	4.34E-23
-0.204927493	0.001257445	-0.468774396	8.67E-15	0.479316918	1.77E-15
-0.591187228	1.75E-24	-0.669075433	3.62E-33	0.342690649	3.71E-08
-0.351513653	1.55E-08	-0.529687834	4.02E-19	0.563652981	6.05E-22
0.425561762	3.38E-12	0.770898426	1.66E-49	-0.335535651	7.37E-08
-0.395053789	1.41E-10	-0.413244764	1.60E-11	0.282656691	7.00E-06
-0.486692098	5.65E-16	-0.549986204	9.04E-21	0.070739077	0.270043886
-0.337796017	5.94E-08	-0.526507891	7.12E-19	0.192249635	0.002510824
0.50381087	3.56E-17	0.492677924	2.19E-16	-0.210303704	0.000926126
0.52558364	8.40E-19	0.793305631	2.75E-54	-0.384880318	4.51E-10
-0.237462408	0.000175661	-0.475560219	3.14E-15	0.484963385	7.40E-16
0.812269923	8.06E-59	0.806622793	2.04E-57	-0.266331283	2.40E-05
-0.267417976	2.22E-05	-0.558735104	1.62E-21	0.166740325	0.008925813
0.704638867	4.47E-38	0.648378487	1.30E-30	-0.101769569	0.112076796
0.5492151	1.05E-20	0.829681171	1.85E-63	-0.389693721	2.61E-10
0.492893377	2.11E-16	0.766098357	1.49E-48	-0.169673179	0.007778197
0.368113868	2.81E-09	0.690545745	4.79E-36	-0.233984926	0.000219766
0.539223605	6.98E-20	0.61208198	1.41E-26	-0.298927578	1.89E-06
0.468542451	8.97E-15	0.684542725	3.24E-35	-0.486053973	6.24E-16
-0.219345224	0.000544315	-0.612641883	1.23E-26	0.26902761	1.97E-05
-0.446412062	2.11E-13	-0.541734003	4.36E-20	-0.078528942	0.22065865
0.651126075	6.13E-31	0.666978252	6.72E-33	-0.140969082	0.027365819
-0.075466377	0.239245911	-0.544092665	2.79E-20	0.518454209	2.95E-18
0.651720858	5.20E-31	0.790269282	1.32E-53	-0.497251593	1.05E-16
0.712915916	2.52E-39	0.702577744	9.01E-38	-0.171550074	0.007114588
0.360220591	6.41E-09	0.671587461	1.72E-33	-0.341925131	4.00E-08
0.040093923	0.532226432	-0.277527854	1.04E-05	0.21718232	0.000619331

0.405549778	4.08E-11	0.536754312	1.10E-19	-0.189202493	0.002946706
0.520823946	1.95E-18	0.824297957	5.69E-62	-0.49364172	1.87E-16
-0.477888532	2.20E-15	-0.575102761	5.69E-23	0.154962794	0.015189437
-0.532261051	2.52E-19	-0.451646037	1.02E-13	0.416423012	1.08E-11
0.637601982	2.34E-29	0.823088547	1.21E-61	-0.393114473	1.77E-10
0.74255167	3.46E-44	0.894875555	4.16E-87	-0.403582799	5.16E-11
0.665654822	9.90E-33	0.884755326	1.56E-82	-0.345537554	2.81E-08
0.582372287	1.21E-23	0.689942215	5.82E-36	-0.393999649	1.59E-10
0.502142697	4.69E-17	0.721491146	1.15E-40	-0.44873572	1.53E-13
0.42784602	2.52E-12	0.793234621	2.86E-54	-0.494595214	1.61E-16
0.700439536	1.85E-37	0.558708849	1.63E-21	-0.457864543	4.24E-14
0.63790344	2.16E-29	0.808806144	5.92E-58	-0.168120366	0.008368305
-0.384288395	4.82E-10	-0.689634492	6.42E-36	0.430561528	1.77E-12
0.838522942	5.05E-66	0.903761621	1.59E-91	-0.41476	1.32E-11
0.72148591	1.15E-40	0.815590236	1.15E-59	-0.368376028	2.73E-09
0.682370048	6.39E-35	0.923005261	8.95E-103	-0.4199914	6.88E-12
0.800165696	7.18E-56	0.843697104	1.35E-67	-0.470654804	6.56E-15
0.610147761	2.23E-26	0.894041863	1.03E-86	-0.470531806	6.68E-15
0.590255716	2.15E-24	0.872188315	2.02E-77	-0.328115963	1.47E-07
0.797793454	2.58E-55	0.853696452	8.35E-71	-0.327920578	1.50E-07
0.677926965	2.52E-34	0.819084435	1.41E-60	-0.261858454	3.32E-05
0.485334306	6.98E-16	0.80413811	8.15E-57	-0.216759996	0.000635051
0.3245684	2.04E-07	0.711794385	3.74E-39	-0.162540067	0.01083081
0.569957357	1.66E-22	0.806805256	1.84E-57	-0.383296261	5.38E-10
0.586091529	5.38E-24	0.781211041	1.23E-51	-0.153294897	0.016333174
-0.408867923	2.73E-11	-0.401941998	6.28E-11	0.53066629	3.37E-19
-0.194625453	0.002212585	-0.494798041	1.56E-16	0.464208246	1.70E-14
0.618908764	2.69E-27	0.862536722	7.58E-74	-0.377482328	1.02E-09
-0.076458976	0.233105161	-0.438653024	6.06E-13	0.649382719	9.90E-31
-0.31008383	7.37E-07	-0.512240585	8.62E-18	0.541556428	4.51E-20
0.106581529	0.096017037	0.342743172	3.69E-08	-0.420773694	6.23E-12
0.001082835	0.986546411	-0.357729383	8.29E-09	0.499199344	7.61E-17
0.772533691	7.75E-50	0.872990764	9.91E-78	-0.333972351	8.54E-08
-0.289535355	4.07E-06	-0.58821257	3.38E-24	0.708893079	1.03E-38
-0.349089198	1.98E-08	-0.682856578	5.49E-35	0.3129529	5.75E-07
-0.506407824	2.31E-17	-0.628478768	2.47E-28	0.33077272	1.15E-07
-0.275693749	1.20E-05	-0.612769041	1.19E-26	0.22098724	0.000493082
0.349094719	1.98E-08	0.598517305	3.35E-25	-0.160233522	0.012022836
0.505114206	2.87E-17	0.711729972	3.83E-39	-0.277180959	1.07E-05
-0.442150443	3.78E-13	-0.685272164	2.57E-35	0.497767698	9.61E-17

0.128650616	0.044244181	0.503115383	4.00E-17	-0.49370751	1.85E-16
0.627698364	3.01E-28	0.83250707	2.90E-64	-0.355152348	1.08E-08
0.640763477	1.01E-29	0.927715295	5.60E-106	-0.454399052	6.94E-14
0.632653747	8.48E-29	0.920705719	2.77E-101	-0.460195528	3.03E-14
-0.3673913	3.03E-09	-0.769929575	2.59E-49	0.609075881	2.88E-26
0.544537311	2.57E-20	0.809027114	5.22E-58	-0.271249368	1.67E-05
0.307623952	9.11E-07	0.60880387	3.07E-26	-0.257155508	4.64E-05
0.429675976	1.99E-12	0.794403759	1.55E-54	-0.360840426	6.02E-09
0.591662984	1.57E-24	0.801907753	2.78E-56	-0.16718086	0.008744366
0.575739502	4.97E-23	0.712888823	2.54E-39	-0.296057572	2.40E-06
-0.374160851	1.47E-09	-0.495342098	1.42E-16	0.456096057	5.45E-14
-0.473779817	4.11E-15	-0.424925641	3.67E-12	0.143281985	0.024907568
0.436356234	8.24E-13	0.763513149	4.76E-48	-0.268613116	2.03E-05
0.699684472	2.38E-37	0.638072363	2.07E-29	-0.506744903	2.18E-17
0.785689739	1.34E-52	0.652147645	4.62E-31	-0.392016724	2.00E-10
-0.37168014	1.92E-09	-0.693190428	2.03E-36	0.363838608	4.41E-09
0.523254251	1.27E-18	0.901235195	3.17E-90	-0.334646663	8.01E-08
0.404624122	4.56E-11	0.45576391	5.72E-14	-0.269329973	1.93E-05
0.417719352	9.15E-12	0.799030118	1.33E-55	-0.459595121	3.31E-14
0.436872463	7.69E-13	0.785323349	1.61E-52	-0.235266154	0.000202435
0.64551034	2.84E-30	0.706433062	2.42E-38	-0.03465612	0.5893059
0.294494831	2.73E-06	0.672324596	1.38E-33	-0.200078251	0.001646215
0.759090398	3.35E-47	0.848200242	5.18E-69	-0.311343948	6.61E-07
0.38706825	3.52E-10	0.526256068	7.45E-19	-0.309983766	7.43E-07
-0.203167049	0.001387616	-0.520502826	2.06E-18	0.292375146	3.24E-06
0.228664859	0.000307619	0.283233797	6.69E-06	-0.103262519	0.106883345
-0.326212946	1.76E-07	-0.513389308	7.08E-18	0.282796536	6.93E-06
-0.262591021	3.15E-05	-0.568181724	2.40E-22	0.567886242	2.55E-22
0.66487994	1.24E-32	0.689768957	6.15E-36	-0.381178438	6.81E-10
-0.374200757	1.46E-09	-0.697084168	5.66E-37	0.441859126	3.93E-13
-0.222038619	0.000462665	-0.5454003	2.18E-20	0.599003934	3.00E-25
0.077113383	0.229117907	-0.476601735	2.68E-15	0.407634143	3.17E-11
0.653417177	3.24E-31	0.844695247	6.61E-68	-0.306931282	9.66E-07
0.531039604	3.15E-19	0.548908866	1.11E-20	-0.087011754	0.174603216
0.597230492	4.50E-25	0.718061817	4.00E-40	-0.216962716	0.00062746
-0.333274667	9.12E-08	-0.319819344	3.13E-07	0.51037335	1.18E-17
0.427756896	2.55E-12	0.731718379	2.46E-42	-0.576960631	3.84E-23
0.600094805	2.34E-25	0.850685106	8.18E-70	-0.247944031	8.76E-05
-0.095085797	0.137787358	-0.337056746	6.38E-08	0.408647291	2.80E-11
0.597145142	4.59E-25	0.663418869	1.90E-32	-0.243035317	0.000121828

0.852920879	1.51E-70	0.780967258	1.39E-51	-0.184127014	0.003827098
0.596498537	5.31E-25	0.799477355	1.04E-55	-0.355544752	1.04E-08
0.347284611	2.37E-08	0.549150299	1.06E-20	-0.344047493	3.25E-08
-0.051454229	0.422669482	-0.456585288	5.09E-14	0.341452427	4.18E-08
0.622022755	1.25E-27	0.909089914	2.20E-94	-0.339527301	5.04E-08
0.322745534	2.41E-07	0.663670152	1.76E-32	-0.314303245	5.11E-07
0.053090785	0.408046588	0.383521347	5.25E-10	-0.442184868	3.76E-13
-0.494514908	1.63E-16	-0.791179384	8.28E-54	0.280892015	8.03E-06
0.54600488	1.94E-20	0.845737491	3.12E-68	-0.389420204	2.70E-10
0.053377895	0.405512457	-0.286300799	5.26E-06	0.2939889	2.84E-06
0.447743658	1.76E-13	0.612487375	1.28E-26	-0.286581501	5.15E-06
0.208969762	0.000999842	0.579849925	2.08E-23	-0.432117384	1.44E-12
0.701704598	1.21E-37	0.778618353	4.32E-51	-0.382047838	6.18E-10
0.843262664	1.84E-67	0.890281621	5.63E-85	-0.351150732	1.61E-08
0.772456372	8.03E-50	0.757232278	7.51E-47	-0.28919994	4.18E-06
0.385659912	4.13E-10	0.55328988	4.75E-21	-0.231505867	0.000257298
-0.411365339	2.01E-11	-0.739515763	1.17E-43	0.434056146	1.12E-12
-0.177868545	0.005236005	-0.576855599	3.93E-23	0.39344312	1.70E-10
0.588030629	3.52E-24	0.566365638	3.48E-22	-0.308506204	8.44E-07
-0.464789153	1.56E-14	-0.676285872	4.16E-34	0.36640862	3.36E-09
-0.1480208	0.020458794	-0.358450618	7.70E-09	0.372331396	1.79E-09
-0.319062111	3.35E-07	-0.512203308	8.67E-18	0.526171984	7.56E-19
-0.542343997	3.89E-20	-0.592095891	1.43E-24	0.375879692	1.22E-09
0.659684713	5.54E-32	0.829934623	1.57E-63	-0.291667017	3.43E-06
-0.302480688	1.41E-06	-0.623924157	7.77E-28	0.363207451	4.71E-09
-0.256969437	4.70E-05	-0.519673919	2.39E-18	0.45773485	4.32E-14
-0.10816614	0.091148647	-0.567478084	2.77E-22	0.354826398	1.11E-08
0.662638504	2.38E-32	0.709814441	7.49E-39	-0.342928731	3.63E-08
-0.094696942	0.139411895	-0.410390541	2.27E-11	0.456187019	5.38E-14
-0.078206728	0.222564329	-0.476612669	2.68E-15	0.346056085	2.67E-08
0.495724922	1.34E-16	0.808245031	8.14E-58	-0.251102248	7.06E-05
0.269447946	1.91E-05	0.564378627	5.22E-22	-0.161936274	0.011132309
0.378839924	8.82E-10	0.442090865	3.81E-13	-0.029884133	0.641593126
-0.443844886	3.00E-13	-0.815669014	1.10E-59	0.523482082	1.22E-18
-0.230459646	0.000274862	-0.361456993	5.64E-09	0.624572776	6.61E-28
0.369402676	2.45E-09	0.469726423	7.53E-15	-0.083718531	0.191554377
-0.273830051	1.38E-05	-0.452764946	8.73E-14	0.311960872	6.26E-07
-0.145925333	0.022333075	-0.337382665	6.18E-08	0.654583162	2.34E-31
0.49559323	1.37E-16	0.709150498	9.43E-39	-0.232132337	0.000247287
-0.191737879	0.002579666	-0.514435542	5.92E-18	0.400540632	7.41E-11

-0.249294582	7.99E-05	-0.576007021	4.70E-23	0.188270573	0.003093104
0.391211766	2.20E-10	0.666148851	8.57E-33	-0.444969465	2.58E-13
0.659159564	6.44E-32	0.780698852	1.58E-51	-0.222476603	0.000450514
0.714285881	1.55E-39	0.751139348	1.01E-45	-0.26408664	2.83E-05
0.472270291	5.15E-15	0.688721683	8.60E-36	-0.364543434	4.09E-09
0.666499935	7.73E-33	0.778678413	4.20E-51	-0.27735427	1.05E-05
0.836426108	2.11E-65	0.813635992	3.63E-59	-0.210947268	0.000892382
-0.291675035	3.43E-06	-0.553087792	4.95E-21	0.436565465	8.02E-13
0.433719553	1.17E-12	0.668456892	4.35E-33	-0.355430115	1.05E-08
0.181110124	0.004456783	0.506375989	2.32E-17	-0.516011877	4.51E-18
-0.33975461	4.93E-08	-0.651287175	5.86E-31	0.376756425	1.11E-09
0.501242331	5.44E-17	0.48536454	6.95E-16	-0.231387015	0.000259239
-0.310562361	7.07E-07	-0.516834039	3.91E-18	0.532755466	2.30E-19
0.703595337	6.38E-38	0.707250833	1.82E-38	-0.129612214	0.042669092
-0.334912076	7.82E-08	-0.492716799	2.17E-16	0.043585607	0.497102048
-0.348009265	2.20E-08	-0.652706785	3.95E-31	0.649670848	9.15E-31
-0.271293398	1.66E-05	-0.539534604	6.59E-20	0.172414654	0.006826263
0.012025518	0.851446363	-0.428234041	2.40E-12	0.360991214	5.92E-09
-0.380669642	7.21E-10	-0.51814956	3.11E-18	0.491213606	2.76E-16
-0.32858066	1.41E-07	-0.462874954	2.06E-14	0.447276931	1.87E-13
-0.613384875	1.03E-26	-0.80364823	1.07E-56	0.377396132	1.03E-09
0.394327867	1.53E-10	0.590224106	2.17E-24	-0.297846316	2.07E-06
0.614939404	7.08E-27	0.686285971	1.87E-35	-0.243074056	0.000121515
-0.336207439	6.91E-08	-0.332762218	9.57E-08	0.370325984	2.22E-09
-0.376369315	1.16E-09	-0.690311977	5.16E-36	0.472894822	4.69E-15
0.488951344	3.95E-16	0.895232812	2.82E-87	-0.462808997	2.08E-14
0.688499456	9.24E-36	0.82780731	6.17E-63	-0.182634742	0.004127762
0.667294029	6.12E-33	0.707765625	1.53E-38	-0.172557732	0.006779568
0.460945347	2.72E-14	0.650382772	7.52E-31	0.022390861	0.727296679
0.703957645	5.64E-38	0.676267868	4.19E-34	-0.410999567	2.10E-11
0.482579903	1.07E-15	0.616098147	5.34E-27	-0.239078686	0.000158113
-0.360642439	6.14E-09	-0.63055094	1.45E-28	0.631363247	1.18E-28
-0.315050951	4.78E-07	-0.520627982	2.02E-18	0.517138183	3.71E-18
0.333268735	9.12E-08	0.365263143	3.79E-09	-0.011951812	0.852346436
-0.383186848	5.45E-10	-0.511160054	1.04E-17	0.164186006	0.010045247
-0.418030018	8.80E-12	-0.517369314	3.56E-18	0.194862568	0.002184665
0.278678174	9.52E-06	0.520566859	2.04E-18	-0.368315655	2.75E-09
0.728014196	1.01E-41	0.853668654	8.53E-71	-0.483870414	8.77E-16
0.373545591	1.57E-09	0.630139613	1.62E-28	-0.398031735	9.96E-11
0.280437966	8.32E-06	0.623263121	9.17E-28	-0.148238723	0.020271935

0.369745576	2.36E-09	0.624696991	6.41E-28	-0.31082352	6.91E-07
-0.311187303	6.70E-07	-0.60771027	3.98E-26	0.252285427	6.51E-05
-0.453376024	8.01E-14	-0.795776187	7.52E-55	0.460581344	2.87E-14
-0.389732715	2.60E-10	-0.611039614	1.81E-26	0.713856316	1.80E-39
-0.076982255	0.229912971	-0.495893562	1.30E-16	0.296452495	2.32E-06
0.500290019	6.37E-17	0.601891857	1.55E-25	-0.386228751	3.87E-10
0.506420701	2.30E-17	0.768992266	3.99E-49	-0.291480896	3.48E-06
0.122203385	0.056111503	0.407765827	3.12E-11	-0.395658841	1.31E-10
0.577390983	3.51E-23	0.915659804	3.64E-98	-0.431177328	1.63E-12
-0.464082713	1.73E-14	-0.524977858	9.36E-19	0.095944439	0.134251581
0.280812851	8.08E-06	0.748534574	2.99E-45	-0.261801002	3.33E-05
-0.405603166	4.05E-11	-0.577210202	3.64E-23	0.355467262	1.04E-08
0.685364426	2.50E-35	0.873517782	6.19E-78	-0.422377631	5.08E-12
0.645915119	2.55E-30	0.517753075	3.33E-18	-0.226878276	0.000343805
-0.196521489	0.001998132	-0.428568109	2.29E-12	0.326175465	1.76E-07
-0.448592738	1.56E-13	-0.490828571	2.94E-16	0.037286519	0.561345821
0.340084901	4.77E-08	0.572609875	9.59E-23	-0.266442026	2.38E-05
0.571738693	1.15E-22	0.565656108	4.03E-22	-0.257334027	4.58E-05
0.356253736	9.63E-09	0.559052656	1.52E-21	-0.128450635	0.044577801
0.541050299	4.96E-20	0.825348608	2.94E-62	-0.381199535	6.80E-10
0.362097341	5.28E-09	0.770576968	1.92E-49	-0.213085745	0.000788221
0.545947912	1.96E-20	0.752096269	6.74E-46	-0.191689357	0.002586282
0.577091112	3.74E-23	0.723668593	5.13E-41	-0.228080403	0.000319047
0.604208049	9.03E-26	0.829717422	1.80E-63	-0.324888285	1.98E-07
0.540902858	5.10E-20	0.733430362	1.27E-42	-0.347165842	2.39E-08
0.488946356	3.96E-16	0.622188988	1.20E-27	-0.283488724	6.56E-06
0.674811428	6.51E-34	0.849174616	2.52E-69	-0.398750817	9.15E-11
-0.283605917	6.50E-06	-0.338133908	5.76E-08	0.500547498	6.10E-17
-0.325359748	1.90E-07	-0.422472638	5.02E-12	0.194104534	0.002275068
0.856218976	1.19E-71	0.737146838	2.99E-43	-0.161076955	0.011574173
-0.574410441	6.58E-23	-0.434914682	9.98E-13	-0.005804163	0.927980739
0.578981809	2.50E-23	0.747188715	5.22E-45	-0.293578127	2.94E-06
-0.363054283	4.78E-09	-0.338721265	5.44E-08	0.146671075	0.021649707
0.751637023	8.18E-46	0.759678181	2.59E-47	-0.202948931	0.001404577
0.250581747	7.32E-05	0.474687929	3.58E-15	-0.397103169	1.11E-10
0.463268298	1.95E-14	0.478557841	1.99E-15	-0.167895206	0.008457097
-0.202096455	0.001472702	-0.419365492	7.44E-12	0.456152603	5.41E-14
-0.242961931	0.000122423	-0.506824849	2.15E-17	0.492172781	2.37E-16
-0.326928944	1.64E-07	-0.536450504	1.17E-19	0.316826747	4.09E-07
0.626215892	4.37E-28	0.896602485	6.21E-88	-0.529953551	3.83E-19

-0.107195908	0.09410549	-0.477025652	2.51E-15	0.534821496	1.58E-19
-0.19467587	0.002206621	-0.441303504	4.24E-13	0.438326515	6.33E-13
-0.325050483	1.95E-07	-0.621479622	1.43E-27	0.387846456	3.23E-10
0.488517772	4.23E-16	0.654165853	2.63E-31	-0.322921006	2.37E-07
-0.269515268	1.90E-05	-0.638645991	1.78E-29	0.239294976	0.000155893
0.288059219	4.58E-06	0.629965905	1.69E-28	-0.191994156	0.00254498
-0.285179664	5.75E-06	-0.651300672	5.84E-31	0.396766128	1.16E-10
-0.505189032	2.83E-17	-0.759955714	2.29E-47	0.302612885	1.39E-06
-0.579897459	2.06E-23	-0.823061889	1.23E-61	0.455170766	6.22E-14
-0.319698424	3.17E-07	-0.677963027	2.49E-34	0.228710456	0.000306744
-0.243192936	0.000120558	-0.534115843	1.80E-19	0.357394893	8.57E-09
-0.237558852	0.000174565	-0.561210152	9.90E-22	0.464834167	1.55E-14
-0.196807494	0.001967482	-0.6325572	8.69E-29	0.324841389	1.99E-07
0.615710293	5.87E-27	0.665778394	9.55E-33	-0.229847818	0.000285645
-0.169165694	0.007966845	-0.499946713	6.73E-17	0.553885937	4.23E-21
0.49654456	1.17E-16	0.753837234	3.22E-46	-0.271808535	1.60E-05
0.58811436	3.45E-24	0.452034528	9.67E-14	-0.190355501	0.002774287
0.28182511	7.47E-06	0.680863924	1.02E-34	-0.556602911	2.48E-21
-0.307640219	9.09E-07	-0.472681364	4.84E-15	0.358785365	7.44E-09
-0.382255205	6.04E-10	-0.505738684	2.58E-17	0.51779065	3.31E-18
0.777469708	7.51E-51	0.866083673	3.97E-75	-0.332814715	9.52E-08
0.545151757	2.28E-20	0.619933646	2.09E-27	-0.030878442	0.630539929
0.357377478	8.59E-09	0.738114729	2.04E-43	-0.271320345	1.66E-05
0.6894812	6.74E-36	0.913834918	4.38E-97	-0.318883371	3.40E-07
-0.274443943	1.31E-05	-0.603277044	1.12E-25	0.699641049	2.42E-37
0.5663861	3.47E-22	0.699362567	2.66E-37	-0.368584296	2.67E-09
0.283617899	6.50E-06	0.617740754	3.58E-27	-0.199447564	0.001704155
0.533191784	2.13E-19	0.592651686	1.26E-24	-0.200875506	0.001575553
0.149097013	0.019550311	0.53906256	7.19E-20	-0.357358115	8.61E-09
-0.250542604	7.34E-05	-0.577393975	3.50E-23	0.485786859	6.51E-16
-0.395737934	1.30E-10	-0.681896865	7.41E-35	0.419514928	7.31E-12
-0.442971237	3.38E-13	-0.592104695	1.43E-24	0.104438464	0.102927623
-0.385569703	4.17E-10	-0.633330767	7.12E-29	0.567132589	2.98E-22
0.523136749	1.30E-18	0.871603137	3.40E-77	-0.405412561	4.15E-11
0.502695504	4.28E-17	0.781842971	9.03E-52	-0.31578281	4.48E-07
0.659104973	6.54E-32	0.664781364	1.28E-32	-0.071295127	0.266287182
0.531062968	3.13E-19	0.66147305	3.32E-32	-0.434707814	1.03E-12
-0.376427829	1.15E-09	-0.713363577	2.15E-39	0.548310003	1.25E-20
0.075586579	0.238496299	0.386426945	3.79E-10	-0.069056747	0.281629028
0.425105072	3.59E-12	0.432478087	1.38E-12	-0.335237836	7.58E-08

-0.244030972	0.000114014	-0.57400842	7.16E-23	0.249573686	7.84E-05
-0.021446252	0.738373039	-0.352677726	1.38E-08	0.188923768	0.002989819
0.386963893	3.56E-10	0.77507535	2.35E-50	-0.368749505	2.63E-09
-0.189457722	0.002907723	-0.675275295	5.66E-34	0.34986896	1.83E-08
0.266446083	2.38E-05	0.698951284	3.05E-37	-0.326915636	1.65E-07
0.703706662	6.14E-38	0.68089941	1.01E-34	-0.34981497	1.84E-08
0.608388451	3.39E-26	0.716118469	8.05E-40	-0.212106539	0.000834439
0.559384168	1.43E-21	0.801310537	3.86E-56	-0.158525437	0.012978617
0.814612689	2.05E-59	0.738791029	1.56E-43	-0.245768916	0.000101489
0.252285948	6.51E-05	0.50230867	4.57E-17	-0.125926972	0.048972264
-0.10692039	0.094958938	-0.569880544	1.69E-22	0.151592141	0.017577687
0.341679455	4.09E-08	0.589850049	2.36E-24	-0.341770734	4.06E-08
0.390576251	2.36E-10	0.734835696	7.38E-43	-0.225693119	0.000369951
0.550571131	8.07E-21	0.702257316	1.00E-37	-0.199550382	0.001694584
0.417667965	9.21E-12	0.564373772	5.23E-22	-0.077553446	0.226463918
0.578772481	2.61E-23	0.884604746	1.81E-82	-0.311363703	6.60E-07
0.499512313	7.23E-17	0.794571602	1.42E-54	-0.396672318	1.17E-10
-0.343095913	3.57E-08	-0.717968552	4.13E-40	0.492375511	2.29E-16
0.281950478	7.40E-06	0.731308635	2.88E-42	-0.279857538	8.70E-06
-0.251613276	6.82E-05	-0.602435062	1.36E-25	0.471504087	5.78E-15
0.743619172	2.25E-44	0.723575692	5.31E-41	-0.112069616	0.079994968
0.475592966	3.12E-15	0.722103423	9.15E-41	-0.154869946	0.015251213
-0.356807673	9.10E-09	-0.577694582	3.29E-23	0.150367196	0.018523326
0.511610937	9.59E-18	0.717821711	4.36E-40	-0.163769506	0.010239112
0.448242646	1.64E-13	0.679044748	1.79E-34	-0.138928637	0.029704405
-0.217205488	0.00061848	-0.413873583	1.48E-11	0.771568605	1.21E-49
-0.248566021	8.40E-05	-0.33440812	8.20E-08	0.461320427	2.58E-14
0.634592647	5.13E-29	0.786080363	1.11E-52	-0.334272911	8.30E-08
-0.356834896	9.08E-09	-0.547941562	1.34E-20	0.463446166	1.90E-14
-0.299269068	1.84E-06	-0.366049939	3.49E-09	0.408530471	2.84E-11
0.530074955	3.75E-19	0.903376123	2.53E-91	-0.373893926	1.51E-09
0.620199176	1.96E-27	0.832756544	2.46E-64	-0.302209406	1.44E-06
0.108146126	0.091208883	0.542752087	3.60E-20	-0.196243613	0.002028328
0.738368417	1.84E-43	0.845355871	4.11E-68	-0.239876991	0.000150063
-0.416770803	1.03E-11	-0.696699679	6.43E-37	0.296461906	2.32E-06
-0.181487322	0.004373254	-0.408503378	2.85E-11	0.254934153	5.42E-05
-0.568713737	2.15E-22	-0.702269224	1.00E-37	0.287699357	4.71E-06
0.689224084	7.32E-36	0.785759844	1.30E-52	-0.258343977	4.26E-05
-0.323400717	2.27E-07	-0.510838419	1.09E-17	0.285712341	5.51E-06
0.552718968	5.32E-21	0.673369921	1.01E-33	-0.121227421	0.058118995

-0.240323224	0.000145733	-0.528129431	5.33E-19	0.541684468	4.40E-20
0.653870179	2.86E-31	0.556849245	2.36E-21	-0.084418669	0.187853482
0.523879916	1.14E-18	0.503567898	3.71E-17	-0.153344313	0.016298235
-0.370242029	2.24E-09	-0.622308135	1.16E-27	0.267222308	2.25E-05
-0.283373236	6.62E-06	-0.661739277	3.08E-32	0.586814941	4.60E-24
0.495679796	1.35E-16	0.640750872	1.02E-29	-0.465590053	1.39E-14
0.563905298	5.75E-22	0.721509365	1.14E-40	-0.199470698	0.001701997
0.3056442	1.08E-06	0.772070824	9.62E-50	-0.37466958	1.39E-09
0.302991711	1.35E-06	0.561302456	9.72E-22	-0.453417689	7.96E-14
0.654878773	2.16E-31	0.866078492	3.99E-75	-0.401720499	6.44E-11
0.560397639	1.16E-21	0.80184577	2.88E-56	-0.179372371	0.004860457
0.369556083	2.41E-09	0.611047528	1.80E-26	-0.319705472	3.16E-07
0.615054256	6.88E-27	0.820804147	4.95E-61	-0.282963042	6.84E-06
0.804832969	5.54E-57	0.777842563	6.28E-51	-0.27437663	1.32E-05
-0.44906434	1.46E-13	-0.580790207	1.70E-23	0.4689324	8.47E-15
-0.022112548	0.73055437	-0.389739136	2.60E-10	0.337313352	6.22E-08
0.562679075	7.36E-22	0.66029647	4.65E-32	-0.478923841	1.88E-15
0.460840647	2.76E-14	0.87108118	5.38E-77	-0.331951685	1.03E-07
0.676756352	3.61E-34	0.820421094	6.26E-61	-0.395477225	1.34E-10
-0.379635408	8.08E-10	-0.741444799	5.41E-44	0.402956938	5.56E-11
-0.163186759	0.010515912	-0.343891479	3.30E-08	0.690038188	5.64E-36
-0.191378619	0.002629015	-0.403794642	5.03E-11	0.563506957	6.23E-22
-0.176266541	0.005664543	-0.597891873	3.87E-25	0.491484734	2.64E-16
0.897165047	3.31E-88	0.844656673	6.79E-68	-0.261742095	3.35E-05
-0.340283113	4.68E-08	-0.641989255	7.32E-30	0.087318063	0.17308501
-0.251962708	6.66E-05	-0.42378142	4.25E-12	0.083796007	0.191142239
0.505514304	2.68E-17	0.696083798	7.88E-37	-0.187615623	0.003199901
0.641723204	7.86E-30	0.656012735	1.57E-31	-0.292222385	3.28E-06
-0.297779081	2.08E-06	-0.4977879	9.58E-17	0.357972495	8.08E-09
0.183626691	0.003925624	0.631258064	1.21E-28	-0.282231012	7.24E-06
-0.402110891	6.15E-11	-0.633685075	6.49E-29	0.454280698	7.05E-14
0.52148401	1.74E-18	0.861168896	2.31E-73	-0.309710005	7.61E-07
0.463717078	1.82E-14	0.60291876	1.22E-25	-0.405664751	4.02E-11
0.02438021	0.704156592	0.387295729	3.43E-10	-0.204071037	0.001319305
0.637699625	2.28E-29	0.91341227	7.73E-97	-0.34035355	4.65E-08
0.749809757	1.76E-45	0.695573255	9.32E-37	-0.19663305	0.001986125
0.623868258	7.88E-28	0.74856691	2.95E-45	-0.307017885	9.59E-07
0.715727479	9.26E-40	0.783270771	4.47E-52	-0.246556341	9.62E-05
0.823487447	9.44E-62	0.756830579	8.93E-47	-0.208534526	0.001025038
-0.331655637	1.06E-07	-0.620716454	1.72E-27	0.509322541	1.41E-17

-0.202199603	0.001464301	-0.541253881	4.77E-20	0.567608551	2.70E-22
0.345102053	2.93E-08	0.387429085	3.38E-10	0.020822324	0.745719008
0.567825726	2.58E-22	0.790536068	1.15E-53	-0.331372622	1.09E-07
0.352696246	1.38E-08	0.531949289	2.67E-19	-0.242888092	0.000123025
0.753427748	3.84E-46	0.781239497	1.21E-51	-0.270955363	1.71E-05
0.636792694	2.89E-29	0.739160228	1.35E-43	-0.291277534	3.54E-06
0.817147703	4.54E-60	0.693273541	1.98E-36	-0.368533525	2.69E-09
0.565601641	4.07E-22	0.48149159	1.27E-15	-0.147420541	0.020981227
0.528730987	4.78E-19	0.772339512	8.48E-50	-0.401566265	6.56E-11
-0.28708069	4.95E-06	-0.586132944	5.34E-24	0.438041473	6.58E-13
0.615654913	5.95E-27	0.690048201	5.62E-36	-0.134413843	0.035491903
0.219580619	0.000536679	0.338828583	5.39E-08	-0.277087418	1.08E-05
-0.317759998	3.76E-07	-0.527897197	5.55E-19	0.544130136	2.77E-20
-0.548761069	1.14E-20	-0.59076919	1.92E-24	0.340739419	4.48E-08
0.744810002	1.39E-44	0.67864685	2.02E-34	-0.167158303	0.008753576
-0.182884458	0.00407601	-0.442370785	3.67E-13	0.400309639	7.62E-11
-0.214695502	0.000717342	-0.589420896	2.59E-24	0.381147458	6.83E-10
0.720354922	1.74E-40	0.79627141	5.79E-55	-0.229925224	0.00028426
-0.278329503	9.78E-06	-0.558721533	1.63E-21	0.318942085	3.39E-07
0.621783462	1.32E-27	0.593103013	1.14E-24	-0.251868949	6.70E-05
0.76522092	2.21E-48	0.899468236	2.44E-89	-0.237056184	0.000180349
-0.358418405	7.72E-09	-0.698362356	3.71E-37	0.338675452	5.46E-08
0.825567411	2.56E-62	0.810148719	2.75E-58	-0.373341519	1.61E-09
-0.036551836	0.569090714	-0.353103026	1.33E-08	0.485568687	6.73E-16
-0.395452964	1.35E-10	-0.667780348	5.31E-33	0.529320759	4.30E-19
-0.295214315	2.57E-06	-0.51237415	8.42E-18	0.327587318	1.55E-07
0.780098727	2.11E-51	0.872994154	9.88E-78	-0.382699937	5.75E-10
0.264318673	2.78E-05	0.638741003	1.73E-29	-0.414871448	1.31E-11
0.485915974	6.38E-16	0.55409468	4.06E-21	-0.424611926	3.82E-12
-0.35832835	7.79E-09	-0.692571957	2.49E-36	0.456315357	5.29E-14
-0.398085964	9.90E-11	-0.571895993	1.11E-22	0.179921962	0.00472937
-0.476543994	2.70E-15	-0.677012007	3.34E-34	0.232094616	0.000247879
0.752889889	4.82E-46	0.703763044	6.02E-38	-0.234190364	0.000216897
0.170172161	0.007596599	0.525492246	8.54E-19	-0.21046133	0.000917755
0.680457884	1.16E-34	0.701289381	1.39E-37	-0.233837285	0.00022185
0.382940809	5.60E-10	0.665805553	9.47E-33	-0.344630733	3.07E-08
-0.354315888	1.17E-08	-0.56906824	2.00E-22	0.416637306	1.05E-11
-0.269205558	1.94E-05	-0.379768696	7.96E-10	0.360613277	6.16E-09
0.494806507	1.55E-16	0.721738644	1.05E-40	-0.336994724	6.42E-08
-0.385681268	4.12E-10	-0.356676923	9.23E-09	0.445245785	2.48E-13

-0.442376221	3.67E-13	-0.483951982	8.66E-16	0.321158885	2.78E-07
-0.544853167	2.42E-20	-0.861305323	2.07E-73	0.446510729	2.08E-13
-0.223281675	0.000428949	-0.471099962	6.14E-15	0.292834395	3.12E-06
-0.455320799	6.09E-14	-0.608672275	3.17E-26	0.450357179	1.22E-13
-0.468857448	8.57E-15	-0.810899205	1.78E-58	0.356593086	9.31E-09
-0.140817145	0.027534344	-0.358832139	7.40E-09	0.36354477	4.54E-09
0.546652311	1.72E-20	0.49633451	1.21E-16	-0.297312211	2.16E-06
0.336776681	6.55E-08	0.410081104	2.35E-11	-0.176427497	0.00562011
0.288954363	4.26E-06	0.721791782	1.03E-40	-0.487669263	4.84E-16
0.386887432	3.60E-10	0.62964276	1.83E-28	-0.318281212	3.59E-07
-0.334183436	8.37E-08	-0.685618002	2.31E-35	0.510263853	1.21E-17
0.70604334	2.76E-38	0.736269753	4.21E-43	-0.339323479	5.14E-08
-0.350649442	1.69E-08	-0.678081347	2.41E-34	0.446449927	2.10E-13
-0.115416278	0.071334394	-0.576904515	3.89E-23	0.361423617	5.66E-09
0.463239314	1.95E-14	0.718914172	2.94E-40	-0.286038141	5.37E-06
0.616444868	4.91E-27	0.898371855	8.52E-89	-0.407844999	3.09E-11
0.226331917	0.000355637	0.63160569	1.11E-28	-0.226574764	0.000350332
0.735399324	5.92E-43	0.881465468	3.86E-81	-0.375405685	1.28E-09
0.540362893	5.64E-20	0.905663046	1.59E-92	-0.45357054	7.80E-14
0.631737114	1.07E-28	0.797535344	2.96E-55	-0.226551471	0.000350837
0.777811453	6.37E-51	0.878161694	8.83E-80	-0.382868364	5.64E-10
0.726217528	1.99E-41	0.882702737	1.17E-81	-0.401268449	6.80E-11
0.640281339	1.15E-29	0.808734391	6.16E-58	-0.403248705	5.37E-11
-0.269733932	1.87E-05	-0.532712451	2.32E-19	0.310503078	7.11E-07
-0.167171686	0.008748111	-0.47733069	2.40E-15	0.387457986	3.37E-10
0.267884213	2.14E-05	0.437782242	6.81E-13	-0.175527883	0.005872523
-0.137770169	0.031106633	-0.322888397	2.38E-07	0.510424439	1.17E-17
-0.238196313	0.000167478	-0.463702895	1.83E-14	0.48944638	3.66E-16
-0.288312908	4.49E-06	-0.353741027	1.24E-08	0.341697005	4.09E-08
-0.325504868	1.87E-07	-0.723627497	5.21E-41	0.369373164	2.46E-09
-0.153294112	0.016333729	-0.42012868	6.76E-12	0.511051834	1.05E-17
-0.405009166	4.35E-11	-0.307337221	9.33E-07	0.124930019	0.050805117
0.530258259	3.63E-19	0.754942261	2.01E-46	-0.368577694	2.67E-09
-0.574033537	7.12E-23	-0.558366061	1.75E-21	0.112174266	0.079711844
-0.189559091	0.002892371	-0.39168656	2.08E-10	0.68051121	1.14E-34
-0.368938929	2.57E-09	-0.679755007	1.44E-34	0.507989849	1.77E-17
0.501849272	4.93E-17	0.820934456	4.57E-61	-0.356111972	9.77E-09
-0.177269767	0.005392649	-0.344446985	3.13E-08	0.393764314	1.64E-10
-0.443498201	3.15E-13	-0.700507834	1.81E-37	0.400858311	7.14E-11
-0.457994929	4.16E-14	-0.674297915	7.61E-34	0.480357014	1.51E-15

-0.363627886	4.50E-09	-0.60137554	1.74E-25	0.473443413	4.32E-15
0.267470403	2.21E-05	0.624832275	6.19E-28	-0.325479157	1.88E-07
0.650824804	6.66E-31	0.706833459	2.10E-38	-0.271374456	1.65E-05
0.135116951	0.034532189	0.582562102	1.16E-23	-0.430484824	1.79E-12
0.737894292	2.22E-43	0.906569958	5.20E-93	-0.291412998	3.50E-06
0.396498112	1.19E-10	0.675100314	5.97E-34	-0.135317389	0.03426267
0.714070704	1.67E-39	0.592912122	1.19E-24	-0.093767487	0.143354254
0.705655942	3.16E-38	0.828565391	3.79E-63	-0.387492804	3.36E-10
0.458163937	4.06E-14	0.600645569	2.06E-25	-0.148432652	0.020106897
-0.342115049	3.92E-08	-0.523327196	1.25E-18	0.525645751	8.31E-19
-0.30006788	1.72E-06	-0.573571235	7.85E-23	0.208945923	0.001001207
0.436574962	8.01E-13	0.60203362	1.50E-25	-0.152447583	0.016942499
0.499876199	6.81E-17	0.706668945	2.23E-38	-0.295128605	2.59E-06
-0.359426202	6.96E-09	-0.573968341	7.22E-23	0.545981381	1.95E-20
0.692247709	2.76E-36	0.854978994	3.11E-71	-0.238918151	0.000159779
0.780514731	1.73E-51	0.709083025	9.66E-39	-0.454840235	6.52E-14
0.345199791	2.91E-08	0.582661734	1.13E-23	-0.16551646	0.009447558
-0.244861105	0.000107861	-0.651771149	5.13E-31	0.477485284	2.34E-15
0.29157515	3.45E-06	0.587905684	3.62E-24	-0.221602799	0.000475056
0.746660467	6.49E-45	0.947413429	3.07E-122	-0.414509662	1.37E-11
0.641927533	7.44E-30	0.811792879	1.06E-58	-0.382439842	5.92E-10
0.437478652	7.10E-13	0.653950956	2.80E-31	-0.237715155	0.000172802
0.384605642	4.65E-10	0.588535781	3.15E-24	-0.264230955	2.80E-05
0.369533339	2.42E-09	0.374031006	1.49E-09	-0.445587593	2.37E-13
0.412344441	1.78E-11	0.574156772	6.94E-23	-0.207748099	0.001072051
0.609928402	2.35E-26	0.797425892	3.13E-55	-0.217337851	0.000613634
-0.338225655	5.71E-08	-0.563483999	6.26E-22	0.647631738	1.60E-30
0.161245116	0.011486507	0.327197069	1.60E-07	-0.239855582	0.000150274
0.46590931	1.32E-14	0.652756047	3.90E-31	-0.161148967	0.01153656
0.419170367	7.63E-12	0.710041668	6.91E-39	-0.426477052	3.01E-12
-0.57455434	6.38E-23	-0.604822136	7.83E-26	0.178300859	0.005125461
-0.350894624	1.65E-08	-0.513300865	7.19E-18	0.284468379	6.08E-06
0.194680798	0.002206039	0.402336815	5.99E-11	-0.191307883	0.002638832
0.701959679	1.11E-37	0.510548228	1.15E-17	-0.192477091	0.002480765
0.731654368	2.53E-42	0.744173783	1.80E-44	-0.315940741	4.42E-07
0.420351175	6.57E-12	0.747148413	5.31E-45	-0.338108441	5.77E-08
0.705295874	3.57E-38	0.83250099	2.92E-64	-0.355125465	1.08E-08
-0.56773789	2.63E-22	-0.715519888	9.97E-40	0.064660684	0.313464503
0.479275405	1.78E-15	0.615859481	5.66E-27	-0.210379261	0.000922105
0.761375335	1.23E-47	0.627801386	2.93E-28	-0.068785335	0.283529005

-0.377120512	1.06E-09	-0.743837573	2.06E-44	0.472245652	5.17E-15
-0.366901927	3.19E-09	-0.59642592	5.40E-25	0.492868436	2.12E-16
-0.142050121	0.026192092	-0.431890984	1.49E-12	0.171229794	0.007224124
0.603912994	9.68E-26	0.539146752	7.08E-20	-0.233297001	0.000229634
-0.287423877	4.81E-06	-0.336581257	6.67E-08	0.301589788	1.52E-06
-0.297246296	2.18E-06	-0.512470478	8.28E-18	0.345660825	2.78E-08
0.494968638	1.51E-16	0.498610766	8.38E-17	-0.294772233	2.67E-06
0.454227895	7.11E-14	0.606407832	5.40E-26	-0.256431322	4.88E-05
-0.111656501	0.081120564	-0.435179671	9.64E-13	0.321145455	2.78E-07
0.545682549	2.07E-20	0.438729427	6.00E-13	-0.215354778	0.00069005
-0.058176874	0.364553075	-0.394367445	1.53E-10	0.570353382	1.53E-22
-0.357297153	8.66E-09	-0.601929211	1.53E-25	0.544219671	2.73E-20
0.287659596	4.72E-06	0.367172547	3.10E-09	-0.196828936	0.001965202
0.578590114	2.72E-23	0.850571235	8.91E-70	-0.379475307	8.22E-10
-0.309569812	7.70E-07	-0.593866682	9.62E-25	0.083442467	0.193028201
-0.131751416	0.039333504	-0.353946888	1.22E-08	0.52663953	6.96E-19
0.409835364	2.43E-11	0.648544898	1.25E-30	-0.257574409	4.50E-05
-0.23589352	0.000194422	-0.553776798	4.32E-21	0.473183232	4.49E-15
0.036020466	0.574723743	-0.344721124	3.04E-08	0.18785803	0.003159991
-0.248119778	8.66E-05	-0.618793227	2.77E-27	0.476426306	2.75E-15
-0.387394362	3.39E-10	-0.64680283	2.00E-30	0.534539499	1.66E-19
-0.41465231	1.34E-11	-0.482640977	1.06E-15	-0.134836619	0.034912157
0.47553327	3.15E-15	0.81029968	2.52E-58	-0.281459447	7.68E-06
0.722030008	9.40E-41	0.742601623	3.39E-44	-0.364534446	4.10E-09
-0.10661336	0.095917246	-0.437219006	7.35E-13	0.438081484	6.55E-13
-0.1199376	0.060862694	-0.334039101	8.49E-08	0.410132374	2.34E-11
-0.436950371	7.61E-13	-0.653342576	3.31E-31	0.548774229	1.14E-20
0.536681147	1.12E-19	0.808326208	7.77E-58	-0.240743281	0.000141763
-0.360767154	6.06E-09	-0.602658945	1.29E-25	0.533846609	1.89E-19
-0.078156185	0.222864312	-0.371997029	1.86E-09	0.217790207	0.000597336
0.617312326	3.98E-27	0.842438598	3.29E-67	-0.223091722	0.000433949
0.580870619	1.67E-23	0.66028555	4.67E-32	-0.263245032	3.00E-05
0.329663859	1.28E-07	0.741966291	4.38E-44	-0.213767904	0.000757431
0.529085022	4.48E-19	0.766451647	1.27E-48	-0.326425482	1.72E-07
-0.422481908	5.02E-12	-0.61355983	9.87E-27	0.317618943	3.81E-07
0.115960595	0.070000831	0.604230861	8.99E-26	-0.45054731	1.19E-13
0.392434937	1.91E-10	0.458778102	3.72E-14	-0.479749818	1.66E-15
0.621644462	1.37E-27	0.718300899	3.67E-40	-0.354732978	1.12E-08
0.690352897	5.10E-36	0.959047888	4.00E-135	-0.408638662	2.81E-11
0.583690474	9.08E-24	0.48312983	9.84E-16	-0.144310932	0.023876557

0.250428102	7.40E-05	0.53269376	2.33E-19	-0.24617398	9.88E-05
0.376031727	1.20E-09	0.529441864	4.20E-19	-0.239950588	0.000149341
0.848314247	4.76E-69	0.801984513	2.67E-56	-0.30401637	1.24E-06
-0.160018912	0.012139415	-0.407979069	3.04E-11	0.203958349	0.001327649
0.568044172	2.47E-22	0.528944377	4.60E-19	-0.262227001	3.23E-05
-0.156595359	0.014138434	-0.358368858	7.76E-09	0.318878272	3.41E-07
0.274807579	1.28E-05	0.549143881	1.06E-20	-0.105341193	0.099970198
-0.247462228	9.05E-05	-0.596625265	5.16E-25	0.478997754	1.86E-15
-0.480035635	1.59E-15	-0.643698945	4.63E-30	0.469313274	8.01E-15
0.653231059	3.42E-31	0.723819353	4.86E-41	-0.494116834	1.74E-16
0.555464098	3.10E-21	0.528227168	5.23E-19	-0.057375662	0.371206725
-0.509851678	1.29E-17	-0.483333629	9.54E-16	0.184731188	0.0037111
-0.290843322	3.66E-06	-0.568261875	2.36E-22	0.430651822	1.75E-12
-0.360142815	6.47E-09	-0.579942761	2.04E-23	0.586139206	5.33E-24
-0.154246648	0.015671644	-0.381009613	6.94E-10	0.369265581	2.49E-09
-0.407001803	3.42E-11	-0.609369132	2.69E-26	0.482830793	1.03E-15
0.629739528	1.79E-28	0.699012323	2.98E-37	-0.317375719	3.89E-07
0.327630364	1.54E-07	0.537762831	9.16E-20	-0.142553336	0.025660649
-0.166033136	0.009224084	-0.389461267	2.68E-10	0.467442685	1.06E-14
-0.314652079	4.95E-07	-0.395622746	1.32E-10	0.580273937	1.90E-23
-0.216763432	0.000634922	-0.62459337	6.57E-28	0.413399392	1.57E-11
0.399591485	8.29E-11	0.611826348	1.50E-26	-0.191976447	0.002547363
-0.246033779	9.97E-05	-0.576493357	4.24E-23	0.688137856	1.04E-35
-0.370387831	2.20E-09	-0.645668292	2.72E-30	0.482951498	1.01E-15
-0.370369948	2.21E-09	-0.634051485	5.91E-29	0.521291638	1.80E-18
-0.419992492	6.88E-12	-0.580027912	2.00E-23	-0.055019044	0.391207407
0.28148987	7.67E-06	0.488360379	4.34E-16	-0.354747688	1.12E-08
-0.450181027	1.25E-13	-0.596884255	4.87E-25	0.33531967	7.52E-08
-0.227580168	0.000329141	-0.451288109	1.07E-13	0.496030273	1.27E-16
0.728891177	7.25E-42	0.729057942	6.80E-42	-0.204213147	0.001308852
-0.101100607	0.114467127	-0.527555307	5.90E-19	0.452948413	8.51E-14
-0.268198858	2.09E-05	-0.370979286	2.07E-09	0.699678949	2.39E-37
-0.375862932	1.22E-09	-0.664509415	1.38E-32	0.595927399	6.04E-25
-0.207229677	0.001104118	-0.604918232	7.66E-26	0.520704082	1.99E-18
0.629822052	1.75E-28	0.726111207	2.07E-41	-0.319263993	3.29E-07
0.557130476	2.23E-21	0.578211133	2.95E-23	-0.159198806	0.012594128
0.420132883	6.76E-12	0.679439887	1.58E-34	-0.292320334	3.25E-06
0.722340937	8.38E-41	0.806380238	2.33E-57	-0.286575783	5.15E-06
-0.108624939	0.089776519	-0.590087524	2.23E-24	0.526976024	6.55E-19
-0.443150133	3.30E-13	-0.550960408	7.48E-21	0.178945087	0.004964618

0.626665643	3.90E-28	0.80380103	9.82E-57	-0.284413872	6.10E-06
0.006513821	0.919203505	-0.461264143	2.60E-14	0.144041859	0.024142553
0.460718116	2.81E-14	0.775707596	1.74E-50	-0.21059109	0.000910915
-0.371218571	2.02E-09	-0.649445191	9.73E-31	0.397885075	1.01E-10
0.510444157	1.17E-17	0.837179005	1.27E-65	-0.30307905	1.34E-06
0.647414488	1.70E-30	0.842834383	2.49E-67	-0.250578277	7.32E-05
-0.325072969	1.95E-07	-0.546259073	1.85E-20	0.470025535	7.20E-15
-0.250608203	7.31E-05	-0.512956734	7.62E-18	0.221714309	0.000471856
0.525554633	8.45E-19	0.908289099	6.06E-94	-0.307496287	9.21E-07
-0.575555739	5.17E-23	-0.706784151	2.14E-38	0.50479104	3.02E-17
-0.368334564	2.74E-09	-0.549897626	9.19E-21	0.57899411	2.49E-23
-0.170695494	0.007410206	-0.396770696	1.15E-10	0.389444598	2.69E-10
-0.483652911	9.07E-16	-0.803133108	1.42E-56	0.641288677	8.82E-30
0.361700078	5.50E-09	0.474436654	3.72E-15	-0.260776833	3.59E-05
-0.426665327	2.94E-12	-0.563566153	6.16E-22	0.49918252	7.63E-17
-0.442736675	3.49E-13	-0.820508033	5.94E-61	0.585324111	6.37E-24
0.568399321	2.30E-22	0.814125862	2.72E-59	-0.180651143	0.004560358
-0.409377359	2.57E-11	-0.759156382	3.25E-47	0.483208016	9.72E-16
-0.274154942	1.34E-05	-0.66074143	4.10E-32	0.590900002	1.87E-24
-0.270643514	1.75E-05	-0.439300035	5.56E-13	0.361257496	5.76E-09
0.45722637	4.64E-14	0.862163282	1.03E-73	-0.326321061	1.74E-07
-0.311232996	6.67E-07	-0.607237305	4.45E-26	0.376748616	1.11E-09
-0.508546479	1.61E-17	-0.599069427	2.96E-25	0.487909974	4.66E-16
0.143267621	0.024922229	0.396612874	1.18E-10	-0.478590994	1.98E-15
0.68554342	2.36E-35	0.849779252	1.61E-69	-0.486392543	5.92E-16
0.622209628	1.19E-27	0.622853625	1.01E-27	-0.331154074	1.11E-07
0.534708105	1.61E-19	0.748782166	2.70E-45	-0.456435768	5.20E-14
-0.213066075	0.000789125	-0.379175957	8.50E-10	0.612491536	1.28E-26
0.459799658	3.21E-14	0.763093934	5.73E-48	-0.325538057	1.87E-07
-0.016354969	0.798952016	-0.347003835	2.43E-08	0.561603542	9.15E-22
0.291939448	3.35E-06	0.752371032	6.00E-46	-0.386851245	3.61E-10
-0.178807163	0.004998667	-0.612521447	1.27E-26	0.433752409	1.16E-12
0.675695934	4.98E-34	0.845938716	2.70E-68	-0.414042035	1.45E-11
-0.523886645	1.14E-18	-0.49202538	2.43E-16	0.101076509	0.114553975
0.416199767	1.11E-11	0.725796023	2.33E-41	-0.522940702	1.34E-18
-0.4204997	6.45E-12	-0.743969509	1.95E-44	0.414302761	1.40E-11
0.749086873	2.38E-45	0.688062292	1.06E-35	-0.259189031	4.02E-05
-0.361848844	5.42E-09	-0.582898332	1.08E-23	0.510920267	1.08E-17
-0.456568309	5.10E-14	-0.391811716	2.05E-10	0.256334514	4.91E-05
0.779758344	2.49E-51	0.817418531	3.86E-60	-0.379276152	8.41E-10

0.558367317	1.75E-21	0.669190991	3.50E-33	-0.300184459	1.71E-06
0.663048644	2.11E-32	0.661055672	3.75E-32	-0.086779126	0.175762812
0.665415158	1.06E-32	0.838137934	6.57E-66	-0.559100898	1.51E-21
-0.404089113	4.86E-11	-0.478562825	1.99E-15	0.236165466	0.000191041
-0.33470729	7.97E-08	-0.629597304	1.86E-28	0.430315782	1.83E-12
-0.303967179	1.24E-06	-0.320363475	2.98E-07	0.346722151	2.50E-08
-0.227607255	0.000328587	-0.472075502	5.30E-15	0.548036147	1.32E-20
0.621897954	1.29E-27	0.637940039	2.14E-29	-0.071654634	0.263877443
-0.33506504	7.70E-08	-0.59008959	2.23E-24	0.459783652	3.22E-14
0.4666038	1.19E-14	0.718518744	3.39E-40	-0.0693692	0.279452404
0.212421563	0.000819305	0.497479908	1.01E-16	-0.347286201	2.37E-08
0.504422114	3.22E-17	0.705149859	3.75E-38	-0.214770772	0.000714176
-0.347305444	2.36E-08	-0.764115139	3.63E-48	0.355913422	9.97E-09
-0.220087683	0.000520568	-0.315871984	4.45E-07	0.482194199	1.14E-15
-0.521246502	1.81E-18	-0.731817366	2.37E-42	0.352479745	1.41E-08
-0.398192609	9.78E-11	-0.613894795	9.11E-27	0.573963891	7.23E-23
0.244277984	0.000112149	0.619806001	2.16E-27	-0.308811892	8.22E-07
0.662927427	2.19E-32	0.680614266	1.10E-34	-0.207346943	0.001096788
0.530537147	3.45E-19	0.540730498	5.27E-20	0.004915103	0.938989057
-0.153237809	0.016373618	-0.446261593	2.16E-13	0.206297749	0.001163988
-0.359422891	6.96E-09	-0.396711331	1.16E-10	0.363769299	4.44E-09
-0.495079741	1.49E-16	-0.607375507	4.30E-26	0.293307351	3.00E-06
-0.180395248	0.004619041	-0.417875916	8.98E-12	0.619549623	2.30E-27
0.418250428	8.56E-12	0.715564051	9.82E-40	-0.162231223	0.010984115
-0.55256946	5.47E-21	-0.49254133	2.23E-16	0.253150179	6.13E-05
-0.442946245	3.39E-13	-0.377900868	9.78E-10	0.258164056	4.32E-05
-0.40567422	4.02E-11	-0.646607931	2.11E-30	0.596418287	5.41E-25
-0.21212908	0.000833348	-0.371691999	1.92E-09	0.369257906	2.49E-09
-0.307640875	9.09E-07	-0.574028199	7.13E-23	0.068222707	0.287495019
-0.421149236	5.94E-12	-0.683887934	3.98E-35	0.093870518	0.142913097
-0.331694385	1.06E-07	-0.509942576	1.27E-17	0.175347583	0.005924316
0.71421073	1.59E-39	0.698420288	3.64E-37	-0.328602965	1.41E-07
0.411593267	1.96E-11	0.775800351	1.66E-50	-0.199528032	0.00169666
0.550021607	8.98E-21	0.800050012	7.65E-56	-0.371925861	1.87E-09
0.588269293	3.34E-24	0.732377534	1.91E-42	-0.292604624	3.18E-06
-0.195433447	0.002118755	-0.306022663	1.04E-06	0.347399911	2.34E-08
-0.478451528	2.02E-15	-0.793228632	2.86E-54	0.545951656	1.96E-20
0.830981878	7.91E-64	0.821900044	2.53E-61	-0.183970582	0.003857661
0.431656198	1.53E-12	0.430726714	1.73E-12	0.094341288	0.140910518
-0.450978421	1.12E-13	-0.773627362	4.64E-50	0.516443287	4.18E-18

0.637180679	2.61E-29	0.875915001	7.05E-79	-0.213761349	0.000757721
0.222482048	0.000450365	0.46776435	1.01E-14	-0.22712984	0.000338481
0.505750422	2.58E-17	0.800058358	7.61E-56	-0.452293421	9.33E-14
-0.421203679	5.90E-12	-0.719172135	2.67E-40	0.562179721	8.14E-22
0.669354946	3.33E-33	0.753961395	3.06E-46	-0.36040855	6.29E-09
-0.137188142	0.031832183	-0.445885314	2.27E-13	0.327735752	1.53E-07
0.580858367	1.67E-23	0.694361219	1.39E-36	-0.259110753	4.04E-05
-0.418695823	8.10E-12	-0.533232638	2.11E-19	0.252426925	6.45E-05
-0.303073699	1.34E-06	-0.564120196	5.50E-22	0.249572886	7.84E-05
-0.431325038	1.60E-12	-0.679151928	1.73E-34	0.266802136	2.32E-05
0.756085745	1.23E-46	0.865127481	8.87E-75	-0.331241413	1.10E-07
-0.188350947	0.003080223	-0.490371321	3.16E-16	0.243946919	0.000114655
-0.210162963	0.000933661	-0.460653521	2.84E-14	0.385312224	4.29E-10
0.09561879	0.135584263	0.470484625	6.73E-15	-0.341262435	4.26E-08
0.603179407	1.15E-25	0.789054599	2.46E-53	-0.312826689	5.81E-07
0.760164927	2.09E-47	0.897778694	1.66E-88	-0.483422714	9.41E-16
0.503312169	3.87E-17	0.799158521	1.24E-55	-0.290056573	3.90E-06
0.57256598	9.68E-23	0.582657474	1.14E-23	-0.062428095	0.330500355
0.55996438	1.27E-21	0.902871324	4.61E-91	-0.367991944	2.85E-09
0.322890022	2.38E-07	0.443506949	3.14E-13	0.033964891	0.596757922
0.467107649	1.11E-14	0.722067388	9.27E-41	-0.273466809	1.41E-05
0.198520518	0.001792709	0.466374812	1.24E-14	-0.138357226	0.030389149
0.57082899	1.39E-22	0.575670536	5.05E-23	-0.199253093	0.001722393
0.512498112	8.25E-18	0.890320401	5.41E-85	-0.325103195	1.94E-07
0.612064237	1.41E-26	0.870163382	1.20E-76	-0.282837263	6.90E-06
-0.345658987	2.78E-08	-0.654811877	2.20E-31	0.294875255	2.64E-06
-0.221234712	0.00048576	-0.553775348	4.32E-21	0.424204702	4.03E-12
-0.463345676	1.92E-14	-0.713264875	2.23E-39	0.386169158	3.90E-10
0.281018839	7.95E-06	0.652975111	3.67E-31	-0.181605581	0.004347358
0.748472879	3.07E-45	0.758491612	4.35E-47	-0.295011815	2.61E-06
0.719796478	2.13E-40	0.800856687	4.94E-56	-0.310032222	7.40E-07
0.465001525	1.51E-14	0.792380969	4.45E-54	-0.258869039	4.11E-05
0.508091879	1.74E-17	0.880469171	1.00E-80	-0.431534671	1.56E-12
-0.413257707	1.59E-11	-0.736775004	3.46E-43	0.524652236	9.92E-19
0.608744639	3.11E-26	0.83248497	2.95E-64	-0.370389199	2.20E-09
0.616495723	4.85E-27	0.802441378	2.08E-56	-0.3775276	1.02E-09
0.773017608	6.18E-50	0.747049434	5.53E-45	-0.279960945	8.63E-06
-0.459409009	3.40E-14	-0.727794876	1.10E-41	0.583460517	9.55E-24
-0.543202345	3.31E-20	-0.767471119	7.99E-49	0.387248247	3.45E-10
0.551769845	6.40E-21	0.805009931	5.02E-57	-0.393592331	1.67E-10

-0.158990019	0.012712258	-0.426156699	3.13E-12	0.328887599	1.37E-07
0.584032281	8.43E-24	0.606435246	5.37E-26	0.001078158	0.986604514
0.581067553	1.60E-23	0.668374648	4.46E-33	-0.160497215	0.011880946
0.553559417	4.51E-21	0.658071929	8.77E-32	-0.374703132	1.39E-09
-0.050742352	0.42912424	-0.5274043	6.07E-19	0.323707174	2.21E-07
0.713463878	2.07E-39	0.617552571	3.75E-27	-0.096157365	0.133385663
0.281564304	7.62E-06	0.51766813	3.38E-18	-0.278843153	9.40E-06
0.583572532	9.32E-24	0.797136074	3.66E-55	-0.373147438	1.64E-09
-0.242705376	0.000124526	-0.438776761	5.96E-13	0.352278681	1.44E-08
-0.410666155	2.19E-11	-0.62104868	1.59E-27	0.539782232	6.29E-20
0.552184814	5.90E-21	0.783423175	4.15E-52	-0.369063042	2.54E-09
-0.367560887	2.98E-09	-0.743953813	1.96E-44	0.423893228	4.19E-12
0.629182051	2.06E-28	0.759523794	2.77E-47	-0.270718276	1.74E-05
-0.257007586	4.68E-05	-0.563784828	5.89E-22	0.355357878	1.06E-08
-0.263098516	3.04E-05	-0.415595183	1.19E-11	0.351662787	1.53E-08
-0.352936537	1.35E-08	-0.660090861	4.94E-32	0.384611515	4.64E-10
0.722797762	7.08E-41	0.669514932	3.18E-33	-0.25239779	6.46E-05
-0.460260371	3.01E-14	-0.725276281	2.82E-41	0.267608213	2.19E-05
-0.155089493	0.015105492	-0.403785572	5.04E-11	0.288694725	4.35E-06
-0.331128615	1.11E-07	-0.625460863	5.29E-28	0.616261379	5.14E-27
-0.387689176	3.28E-10	-0.764388969	3.21E-48	0.384699321	4.60E-10
0.371126906	2.04E-09	0.646775305	2.02E-30	-0.100321999	0.117299255
0.536909828	1.07E-19	0.694065638	1.53E-36	-0.187429327	0.003230884
0.522950561	1.34E-18	0.504355191	3.25E-17	-0.004523265	0.943844581
0.607755326	3.93E-26	0.940296779	9.88E-116	-0.39250269	1.89E-10
0.462575005	2.15E-14	0.85440855	4.83E-71	-0.256390228	4.89E-05
-0.518056872	3.16E-18	-0.692732558	2.36E-36	0.483201079	9.73E-16
0.619957702	2.08E-27	0.693554971	1.81E-36	-0.284792491	5.92E-06
-0.195184119	0.002147315	-0.499479033	7.27E-17	0.333755784	8.72E-08
-0.347605012	2.29E-08	-0.42256817	4.96E-12	0.402007094	6.23E-11
0.502624484	4.33E-17	0.562208958	8.10E-22	-0.245061159	0.000106425
0.453190753	8.22E-14	0.711391237	4.31E-39	-0.354213156	1.18E-08
0.636559045	3.07E-29	0.616551415	4.79E-27	-0.1582868	0.013117333
0.464493456	1.63E-14	0.543169545	3.33E-20	-0.273003193	1.46E-05
0.437628411	6.96E-13	0.66206906	2.80E-32	0.000231337	0.997125647
0.541319785	4.72E-20	0.578919267	2.53E-23	-0.119100898	0.062698905
0.558483418	1.71E-21	0.493427965	1.94E-16	-0.112049893	0.080048417
0.427853378	2.52E-12	0.567152759	2.96E-22	-0.192057266	0.002536503
0.579272503	2.35E-23	0.605122791	7.30E-26	-0.247201543	9.21E-05
0.561539786	9.26E-22	0.874644378	2.24E-78	-0.38173212	6.41E-10

-0.28726024	4.88E-06	-0.563707759	5.98E-22	0.477016866	2.52E-15
-0.198648898	0.0017802	-0.378338843	9.32E-10	0.556836317	2.37E-21
-0.261005891	3.53E-05	-0.367812229	2.90E-09	0.498028619	9.21E-17
0.347282553	2.37E-08	0.589570578	2.51E-24	-0.216390077	0.000649123
-0.462165456	2.28E-14	-0.609937467	2.35E-26	0.292679669	3.16E-06
-0.367524539	2.99E-09	-0.660832727	3.99E-32	0.575569163	5.16E-23
0.130630766	0.041052375	-0.413216715	1.60E-11	0.30088858	1.61E-06
-0.264528339	2.74E-05	-0.546930428	1.63E-20	0.38397761	4.99E-10
-0.195846101	0.002072247	-0.476868882	2.57E-15	0.349209383	1.96E-08
-0.001014922	0.987390112	-0.351680703	1.53E-08	0.34630591	2.61E-08
-0.11068403	0.083820686	-0.378534432	9.12E-10	0.313135898	5.65E-07
-0.176206043	0.005681326	-0.471375355	5.89E-15	0.462784136	2.09E-14
-0.218639187	0.000567824	-0.458793522	3.71E-14	0.388152311	3.12E-10
-0.35016033	1.78E-08	-0.669786739	2.93E-33	0.467707275	1.02E-14
0.705502581	3.33E-38	0.805179352	4.57E-57	-0.286584182	5.14E-06
-0.304357674	1.20E-06	-0.658884023	6.96E-32	0.412396395	1.77E-11
0.86112372	2.40E-73	0.762480064	7.53E-48	-0.268423471	2.06E-05
-0.145391512	0.022833621	-0.298557155	1.95E-06	0.33713486	6.33E-08
-0.338532891	5.54E-08	-0.452248748	9.39E-14	0.592957522	1.18E-24
-0.453541877	7.83E-14	-0.740822569	6.94E-44	0.337055881	6.38E-08
-0.438961477	5.82E-13	-0.622387275	1.14E-27	0.369228613	2.50E-09
0.723097777	6.34E-41	0.880632749	8.57E-81	-0.306410812	1.01E-06
-0.447177614	1.90E-13	-0.760139948	2.12E-47	0.51366089	6.76E-18
0.708584371	1.15E-38	0.914029264	3.37E-97	-0.381359339	6.68E-10
0.637098389	2.67E-29	0.913365519	8.23E-97	-0.374807746	1.37E-09
0.650671966	6.94E-31	0.866822925	2.13E-75	-0.488147355	4.49E-16
0.761280819	1.28E-47	0.924719764	6.45E-104	-0.418999667	7.79E-12
0.426363774	3.05E-12	0.661285298	3.51E-32	-0.220384062	0.000511359
-0.011032302	0.863590866	-0.380774147	7.12E-10	0.400699988	7.27E-11
0.638516561	1.84E-29	0.773426828	5.10E-50	-0.239051198	0.000158397
0.488986004	3.93E-16	0.819327142	1.22E-60	-0.268248064	2.09E-05
-0.301422681	1.54E-06	-0.535773164	1.32E-19	0.272460599	1.53E-05
0.76676153	1.10E-48	0.709854456	7.38E-39	-0.054648809	0.394407668
-0.230629555	0.000271936	-0.413989611	1.46E-11	0.32680492	1.66E-07
0.390128775	2.49E-10	0.758168511	5.00E-47	-0.13562726	0.033849516
0.442541925	3.59E-13	0.824761653	4.26E-62	-0.316871137	4.07E-07
-0.396596675	1.18E-10	-0.655608834	1.76E-31	0.168311552	0.008293563
0.734650183	7.93E-43	0.801000727	4.57E-56	-0.35018252	1.78E-08
0.711515392	4.13E-39	0.919868419	9.42E-101	-0.34293332	3.62E-08
0.717584177	4.75E-40	0.86725418	1.47E-75	-0.298770852	1.92E-06

0.801821382	2.92E-56	0.740807676	6.98E-44	-0.166855516	0.008878049
0.587516776	3.94E-24	0.843608642	1.44E-67	-0.480987124	1.37E-15
0.571781103	1.14E-22	0.826782284	1.19E-62	-0.442770625	3.48E-13
0.377113972	1.07E-09	0.549036644	1.09E-20	-0.257180996	4.63E-05
0.495140552	1.47E-16	0.653348058	3.31E-31	-0.098660258	0.12352632
0.543553962	3.09E-20	0.755510025	1.58E-46	-0.238180466	0.000167651
0.4554501	5.98E-14	0.377478707	1.02E-09	-0.116139477	0.069567039
0.493915629	1.79E-16	0.606229894	5.63E-26	-0.354920025	1.10E-08
0.601849231	1.56E-25	0.851665004	3.91E-70	-0.383100002	5.50E-10
0.639820463	1.30E-29	0.775616766	1.82E-50	-0.310295522	7.24E-07
0.460488293	2.91E-14	0.750223481	1.48E-45	-0.316528141	4.20E-07
0.664747678	1.29E-32	0.669059096	3.64E-33	-0.180959784	0.004490474
0.824567287	4.81E-62	0.807472309	1.26E-57	-0.126331531	0.0482444
0.592192989	1.40E-24	0.897537146	2.18E-88	-0.370780594	2.11E-09
-0.604521283	8.40E-26	-0.838406897	5.46E-66	0.503066718	4.03E-17
0.572174973	1.05E-22	0.643136591	5.39E-30	-0.400509661	7.44E-11
0.648976203	1.11E-30	0.620255311	1.93E-27	-0.396848954	1.14E-10
0.573206034	8.47E-23	0.667137888	6.41E-33	-0.335455841	7.42E-08
0.731122943	3.10E-42	0.889787646	9.42E-85	-0.338849135	5.37E-08
-0.237031772	0.000180635	-0.605368547	6.89E-26	0.461871314	2.38E-14
0.826227027	1.69E-62	0.890744117	3.47E-85	-0.237720256	0.000172744
0.71334466	2.16E-39	0.915861601	2.76E-98	-0.273234922	1.44E-05
-0.457596386	4.40E-14	-0.675716726	4.95E-34	0.313371096	5.54E-07
-0.40315022	5.44E-11	-0.431203381	1.63E-12	0.117713115	0.065844722
0.643142225	5.38E-30	0.757919283	5.58E-47	-0.319850035	3.12E-07
0.048142383	0.45317709	-0.344485571	3.12E-08	0.088022787	0.169629351
0.515445665	4.97E-18	0.718484698	3.43E-40	-0.481947993	1.18E-15
-0.272672742	1.50E-05	-0.590001523	2.28E-24	0.189452615	0.002908499
-0.619490212	2.33E-27	-0.719015257	2.83E-40	0.49798175	9.28E-17
-0.313129142	5.66E-07	-0.535469848	1.40E-19	0.270889283	1.72E-05
-0.365184652	3.83E-09	-0.645937482	2.53E-30	0.511009364	1.06E-17
-0.466669464	1.18E-14	-0.761919563	9.65E-48	0.533353314	2.07E-19
-0.301369592	1.55E-06	-0.563531395	6.20E-22	0.605556499	6.59E-26
0.660042402	5.01E-32	0.751746804	7.81E-46	-0.356773543	9.14E-09
0.566525453	3.37E-22	0.734515368	8.36E-43	-0.142286645	0.025941135
-0.21973975	0.000531574	-0.563702462	5.99E-22	0.487683676	4.83E-16
-0.239138731	0.000157493	-0.554798482	3.54E-21	0.472478532	4.99E-15
0.61748562	3.81E-27	0.871677631	3.18E-77	-0.375039515	1.34E-09
0.59316474	1.13E-24	0.700383155	1.89E-37	-0.297705816	2.10E-06
0.770583255	1.92E-49	0.775617739	1.82E-50	-0.288354146	4.47E-06

0.70392226	5.71E-38	0.823005549	1.27E-61	-0.349491228	1.90E-08
-0.249916874	7.66E-05	-0.57251287	9.79E-23	0.10403822	0.10426079
-0.180992994	0.004483012	-0.593607461	1.02E-24	0.300428332	1.67E-06
0.408795175	2.75E-11	0.506969215	2.10E-17	0.049240051	0.442931284
0.394942578	1.43E-10	0.751659757	8.10E-46	-0.465956789	1.31E-14
0.51401438	6.36E-18	0.621644714	1.37E-27	-0.31182689	6.34E-07
0.563956407	5.69E-22	0.668542672	4.24E-33	-0.104874917	0.101489241
-0.305584329	1.08E-06	-0.477384017	2.38E-15	0.308799101	8.23E-07
-0.223075172	0.000434387	-0.29791217	2.06E-06	0.521476328	1.74E-18
-0.414206842	1.42E-11	-0.683352676	4.70E-35	0.39596943	1.27E-10
0.211126749	0.000883175	0.558450473	1.72E-21	-0.316192078	4.32E-07
-0.336576495	6.68E-08	-0.55639752	2.58E-21	0.348921992	2.01E-08
0.803683668	1.05E-56	0.875470315	1.06E-78	-0.391194621	2.20E-10
0.668348109	4.49E-33	0.614916705	7.11E-27	-0.36121105	5.79E-09
0.645241094	3.06E-30	0.671296134	1.87E-33	-0.343255271	3.51E-08
0.432644059	1.35E-12	0.4861609	6.14E-16	-0.078613929	0.220157955
-0.200109404	0.001643401	-0.666731629	7.22E-33	0.290220189	3.85E-06
0.730667515	3.69E-42	0.659904452	5.21E-32	-0.181464465	0.004378275
0.425257181	3.52E-12	0.718267749	3.71E-40	-0.65736823	1.07E-31
0.32759482	1.55E-07	0.768266643	5.56E-49	-0.445269893	2.47E-13
0.09943008	0.120610396	0.530837305	3.27E-19	-0.512804318	7.82E-18
0.789999368	1.52E-53	0.943565737	1.29E-118	-0.423967672	4.15E-12
-0.107693699	0.092579033	-0.403036111	5.51E-11	0.222470768	0.000450674
0.236428316	0.000187826	0.45544112	5.98E-14	-0.331766721	1.05E-07
0.717693864	4.57E-40	0.749895974	1.70E-45	-0.167050586	0.008797679
0.773048983	6.09E-50	0.781719306	9.59E-52	-0.459585199	3.31E-14
0.668396353	4.43E-33	0.612434641	1.29E-26	-0.17320442	0.006572054
0.654431824	2.44E-31	0.89206249	8.63E-86	-0.351141529	1.61E-08
0.463448298	1.89E-14	0.838413644	5.44E-66	-0.381129017	6.85E-10
0.350398997	1.74E-08	0.496425772	1.20E-16	0.016860416	0.792877181
-0.43996168	5.08E-13	-0.707416511	1.72E-38	0.589797516	2.38E-24
-0.464098842	1.72E-14	-0.628951881	2.19E-28	0.364613914	4.06E-09
0.617110064	4.18E-27	0.786485311	9.02E-53	-0.106735759	0.095534294
0.356257079	9.63E-09	0.43645238	8.14E-13	-0.002320188	0.971177899
0.486515664	5.80E-16	0.604458987	8.52E-26	-0.332326466	9.97E-08
0.317061395	4.00E-07	0.576355775	4.37E-23	-0.251060823	7.08E-05
0.782738363	5.81E-52	0.753369423	3.93E-46	-0.375313154	1.30E-09
0.336361884	6.81E-08	0.733420668	1.28E-42	-0.302491267	1.41E-06
0.267852008	2.15E-05	0.295999492	2.41E-06	-0.249751751	7.75E-05
-0.289229292	4.17E-06	-0.605841696	6.17E-26	0.160974866	0.011627682

0.699754468	2.33E-37	0.880139851	1.37E-80	-0.309531932	7.73E-07
0.75344423	3.81E-46	0.845332279	4.18E-68	-0.37101619	2.06E-09
0.359567298	6.86E-09	0.658060697	8.80E-32	-0.124312333	0.051969097
0.591471926	1.64E-24	0.817956693	2.79E-60	-0.300051557	1.73E-06
0.531845249	2.72E-19	0.561356265	9.61E-22	-0.246852747	9.43E-05
0.505477968	2.70E-17	0.726117031	2.06E-41	-0.169263716	0.007930093
0.569900185	1.68E-22	0.670111153	2.66E-33	-0.050125797	0.434760428
-0.292398925	3.23E-06	-0.42794937	2.49E-12	0.310158013	7.32E-07
0.509068071	1.48E-17	0.800245527	6.88E-56	-0.215141095	0.000698789
0.557976068	1.89E-21	0.872496518	1.54E-77	-0.325539065	1.87E-07
0.758419468	4.49E-47	0.68581121	2.17E-35	-0.446798237	2.00E-13
-0.262868823	3.09E-05	-0.625382209	5.39E-28	0.423439991	4.44E-12
0.292820634	3.12E-06	0.715662299	9.48E-40	-0.127581083	0.046053061
0.747420843	4.75E-45	0.93089956	2.86E-108	-0.451292445	1.07E-13
0.677766692	2.65E-34	0.819025301	1.46E-60	-0.216361647	0.000650216
0.422481984	5.02E-12	0.469398378	7.91E-15	-0.167716316	0.008528238
-0.445150781	2.51E-13	-0.741859728	4.58E-44	0.365490013	3.71E-09
-0.303872379	1.25E-06	-0.541560695	4.51E-20	0.541771084	4.33E-20
-0.355244606	1.07E-08	-0.541235597	4.79E-20	0.522682827	1.41E-18
-0.191377085	0.002629227	-0.486425563	5.89E-16	0.674335905	7.52E-34
-0.186729311	0.003349743	-0.496330398	1.21E-16	0.525818652	8.06E-19
-0.613001193	1.13E-26	-0.474500662	3.68E-15	0.244948389	0.000107232
-0.237994714	0.00016969	-0.546171756	1.88E-20	0.340141013	4.75E-08
-0.029049939	0.650927831	-0.463262959	1.95E-14	0.152834293	0.016661988
0.236683181	0.000184757	0.53799233	8.78E-20	-0.17410004	0.006294039
0.601854719	1.56E-25	0.844673928	6.71E-68	-0.381988399	6.23E-10
0.732921216	1.55E-42	0.924043364	1.83E-103	-0.398312699	9.64E-11
0.628906249	2.21E-28	0.565985501	3.76E-22	-0.416982775	1.00E-11
0.745963142	8.65E-45	0.818858373	1.62E-60	-0.436600438	7.98E-13
0.509891346	1.28E-17	0.745593817	1.01E-44	-0.347440701	2.33E-08
-0.029104064	0.650320496	-0.282650866	7.01E-06	0.191963803	0.002549066
0.347255973	2.37E-08	0.642468457	6.44E-30	-0.457460956	4.49E-14
0.602736121	1.27E-25	0.700838052	1.62E-37	-0.107697036	0.092568866
-0.234213558	0.000216575	-0.529597717	4.09E-19	0.425443283	3.44E-12
-0.406630534	3.58E-11	-0.750676768	1.22E-45	0.319130318	3.33E-07
-0.28857539	4.39E-06	-0.754461935	2.47E-46	0.569716288	1.75E-22
0.569614319	1.79E-22	0.743048193	2.83E-44	-0.347203631	2.39E-08
0.48145157	1.28E-15	0.774904475	2.54E-50	-0.294171305	2.80E-06
0.592578303	1.28E-24	0.590928334	1.85E-24	-0.068271179	0.287151879
-0.253840344	5.85E-05	-0.395651931	1.32E-10	0.364985955	3.91E-09

-0.458634748	3.80E-14	-0.584238955	8.06E-24	0.555182864	3.28E-21
-0.175305552	0.005936449	-0.44250355	3.60E-13	0.380380997	7.44E-10
0.499524911	7.22E-17	0.773735492	4.41E-50	-0.332667086	9.65E-08
0.608155306	3.58E-26	0.897594143	2.05E-88	-0.432684446	1.34E-12
0.543785849	2.96E-20	0.637298963	2.53E-29	-0.228278903	0.000315122
0.619712463	2.21E-27	0.878928121	4.31E-80	-0.345464125	2.83E-08
0.360803694	6.04E-09	0.804176167	7.98E-57	-0.320397479	2.97E-07
-0.478536336	2.00E-15	-0.757100142	7.95E-47	0.39696168	1.13E-10
-0.441456517	4.16E-13	-0.666079534	8.74E-33	0.246041679	9.96E-05
-0.315546469	4.58E-07	-0.700210571	2.00E-37	0.336533776	6.70E-08
-0.218616481	0.000568595	-0.431065717	1.66E-12	0.405585502	4.06E-11
-0.410192198	2.32E-11	-0.536730897	1.11E-19	0.155391313	0.014907152
0.606639908	5.12E-26	0.689354228	7.02E-36	-0.081699237	0.202526321
-0.241925374	0.000131129	-0.40781675	3.10E-11	0.409147409	2.64E-11
0.068656203	0.284435998	-0.365605204	3.66E-09	0.111141932	0.082540418
0.638201094	2.00E-29	0.788986074	2.55E-53	-0.106936286	0.09490953
-0.171833597	0.007018862	-0.353936646	1.22E-08	0.541599959	4.47E-20
0.20116999	0.001550161	0.468969858	8.42E-15	-0.270958941	1.71E-05
0.170976554	0.007311795	0.637139966	2.64E-29	-0.532693551	2.33E-19
0.553939948	4.19E-21	0.708589227	1.15E-38	-0.356189147	9.70E-09
0.564973446	4.63E-22	0.6289221	2.20E-28	-0.28031942	8.39E-06
-0.394864428	1.44E-10	-0.604745955	7.97E-26	0.349347604	1.93E-08
-0.390710375	2.33E-10	-0.548095936	1.30E-20	0.476396527	2.77E-15
-0.458512354	3.86E-14	-0.629700059	1.81E-28	0.467842381	9.95E-15
-0.264758413	2.69E-05	-0.496100782	1.26E-16	0.282726328	6.96E-06
-0.275203618	1.24E-05	-0.55060651	8.02E-21	0.74806051	3.64E-45
-0.51028449	1.20E-17	-0.705149169	3.75E-38	0.268022709	2.12E-05
-0.170609875	0.007440419	-0.414720889	1.33E-11	0.57335705	8.21E-23
0.55453608	3.72E-21	0.787532098	5.33E-53	-0.587653809	3.82E-24
-0.259818236	3.84E-05	-0.456728553	4.98E-14	0.571809765	1.13E-22
-0.371348292	1.99E-09	-0.629202044	2.05E-28	0.224196511	0.000405608
-0.211285251	0.000875117	-0.474135787	3.89E-15	0.111317694	0.082053203
-0.238829695	0.000160705	-0.637919732	2.15E-29	0.564897621	4.70E-22
0.359198018	7.13E-09	0.578607558	2.71E-23	-0.582167521	1.26E-23
-0.215793802	0.000672412	-0.624456134	6.80E-28	0.310552023	7.08E-07
0.001326166	0.983523547	-0.403742846	5.06E-11	0.262591394	3.15E-05
-0.263157811	3.02E-05	-0.472529521	4.96E-15	0.396905531	1.14E-10
0.570461318	1.50E-22	0.757824591	5.81E-47	-0.134717794	0.035074282
0.520921429	1.92E-18	0.865077008	9.25E-75	-0.241244787	0.000137157
0.765782812	1.72E-48	0.897749063	1.72E-88	-0.321471172	2.70E-07

-0.354362488	1.17E-08	-0.548219173	1.27E-20	0.295335301	2.55E-06
-0.204083826	0.001318362	-0.456629396	5.06E-14	0.502227298	4.63E-17
0.207950664	0.001059756	0.423418883	4.45E-12	-0.137602101	0.031314682
-0.39256733	1.88E-10	-0.605236013	7.11E-26	0.342017278	3.96E-08
0.525886807	7.96E-19	0.614495805	7.88E-27	-0.253697179	5.90E-05
-0.495885516	1.30E-16	-0.775200963	2.21E-50	0.276358328	1.14E-05
0.20833911	0.00103654	0.621001574	1.61E-27	-0.16808902	0.008380617
0.210715776	0.000904387	0.489401319	3.68E-16	-0.43468067	1.03E-12
-0.323122644	2.33E-07	-0.679488705	1.56E-34	0.510595325	1.14E-17
0.358830686	7.40E-09	0.579556384	2.21E-23	-0.153739455	0.016021201
0.450730435	1.16E-13	0.689872806	5.95E-36	-0.363859567	4.40E-09
0.628538488	2.43E-28	0.651465685	5.58E-31	-0.229002471	0.000301193
0.060263152	0.34757797	-0.38905098	2.81E-10	0.368330423	2.75E-09
0.441962379	3.88E-13	0.77191862	1.03E-49	-0.318153502	3.63E-07
0.793851332	2.07E-54	0.769770161	2.79E-49	-0.280648995	8.18E-06
-0.285188159	5.74E-06	-0.411245611	2.04E-11	0.411203055	2.05E-11
0.328847108	1.38E-07	0.689828245	6.03E-36	-0.352595455	1.39E-08
0.259366226	3.97E-05	0.656599455	1.33E-31	-0.406953765	3.44E-11
0.688748549	8.53E-36	0.926949404	1.92E-105	-0.403994831	4.91E-11
0.409239045	2.61E-11	0.65397038	2.78E-31	-0.330333975	1.20E-07
0.62282141	1.02E-27	0.752909876	4.78E-46	-0.14919387	0.019470294
0.662980271	2.15E-32	0.572454265	9.91E-23	-0.064327537	0.315969468
0.554248067	3.94E-21	0.546070432	1.92E-20	-0.305465274	1.09E-06
0.246640664	9.57E-05	0.504652637	3.10E-17	0.06875889	0.283714588
0.457502689	4.46E-14	0.666443289	7.86E-33	-0.21677986	0.000634304
0.748969535	2.50E-45	0.884878315	1.38E-82	-0.33440165	8.20E-08
-0.24236462	0.000127371	-0.593010695	1.16E-24	0.307368487	9.31E-07
0.479804405	1.64E-15	0.777544816	7.24E-51	-0.305752736	1.07E-06
0.654300934	2.54E-31	0.919955436	8.30E-101	-0.344642065	3.07E-08
0.594076994	9.17E-25	0.830617916	1.00E-63	-0.387014759	3.54E-10
-0.364008119	4.33E-09	-0.632683619	8.41E-29	0.566148008	3.64E-22
0.392966237	1.80E-10	0.595160683	7.19E-25	-0.216317853	0.000651903
0.736707126	3.55E-43	0.563701294	5.99E-22	-0.345941916	2.70E-08
0.751972739	7.10E-46	0.759180799	3.22E-47	-0.219597283	0.000536143
0.393097503	1.77E-10	0.779078647	3.46E-51	-0.350407916	1.74E-08
0.730176791	4.44E-42	0.713120531	2.34E-39	-0.196547935	0.00199528
0.546321345	1.83E-20	0.822592389	1.65E-61	-0.143545379	0.024640063
0.378850831	8.81E-10	0.685956422	2.07E-35	-0.114684732	0.073159184
0.644224405	4.02E-30	0.70489738	4.09E-38	-0.12465495	0.051320755
0.581711091	1.39E-23	0.61114258	1.76E-26	-0.293450211	2.97E-06

0.479754718	1.66E-15	0.734696827	7.79E-43	-0.116925471	0.067686914
0.612017248	1.43E-26	0.767291229	8.67E-49	-0.303948367	1.24E-06
0.551826064	6.33E-21	0.71937879	2.48E-40	-0.16109567	0.011564388
0.499151773	7.67E-17	0.805986399	2.91E-57	-0.246227624	9.84E-05
0.526274053	7.43E-19	0.667539421	5.70E-33	-0.104586601	0.102437625
0.411444793	1.99E-11	0.789351819	2.11E-53	-0.262041143	3.28E-05
-0.511454668	9.85E-18	-0.875394978	1.13E-78	0.539390932	6.77E-20
0.629883079	1.73E-28	0.680648343	1.09E-34	-0.119592018	0.061615663
-0.295755127	2.46E-06	-0.664842499	1.25E-32	0.225770461	0.00036819
-0.40939542	2.56E-11	-0.789187722	2.30E-53	0.241252433	0.000137087
0.644189891	4.06E-30	0.730654239	3.70E-42	-0.427811267	2.53E-12
0.595701021	6.36E-25	0.639442858	1.44E-29	-0.292551088	3.19E-06
0.515143534	5.24E-18	0.609818731	2.41E-26	-0.279294759	9.08E-06
0.853042417	1.38E-70	0.709050726	9.77E-39	-0.08069082	0.208172931
0.31905594	3.35E-07	0.583680368	9.10E-24	-0.472889432	4.70E-15
0.511060789	1.05E-17	0.497084294	1.07E-16	-0.116882432	0.067788779
0.280960095	7.99E-06	0.670725285	2.22E-33	-0.279767238	8.76E-06
-0.416272633	1.10E-11	-0.480720862	1.43E-15	0.275101526	1.25E-05
-0.225177527	0.000381893	-0.424868194	3.70E-12	0.340869189	4.43E-08
-0.193029052	0.00240918	-0.473774543	4.11E-15	0.461865513	2.38E-14
0.496933055	1.10E-16	0.713444573	2.09E-39	-0.164132706	0.010069874
0.810716588	1.98E-58	0.79535869	9.38E-55	-0.232519862	0.000241277
0.636970289	2.76E-29	0.805202503	4.51E-57	-0.388357526	3.04E-10
0.49082505	2.94E-16	0.616849109	4.45E-27	-0.35565398	1.02E-08
0.751784557	7.69E-46	0.800237163	6.91E-56	-0.239136087	0.000157521
0.606679032	5.07E-26	0.818611175	1.88E-60	-0.232418281	0.000242839
0.57411533	7.00E-23	0.873518534	6.18E-78	-0.366009591	3.51E-09
0.673527088	9.59E-34	0.621352327	1.47E-27	-0.320442599	2.96E-07
0.335474798	7.41E-08	0.625681163	5.00E-28	-0.168087681	0.008381143
0.538445734	8.07E-20	0.799315011	1.14E-55	-0.248052245	8.70E-05
-0.515852278	4.63E-18	-0.722408212	8.18E-41	0.371599271	1.94E-09
0.481746613	1.22E-15	0.634969014	4.65E-29	-0.084340264	0.188265301
-0.293536646	2.95E-06	-0.639111866	1.57E-29	0.388793444	2.90E-10
0.75713862	7.82E-47	0.831698614	4.95E-64	-0.389355157	2.72E-10
0.434394458	1.07E-12	0.769928905	2.59E-49	-0.358478831	7.67E-09
0.711459161	4.21E-39	0.75824813	4.83E-47	-0.206586089	0.001145153
-0.242841676	0.000123405	-0.596955933	4.79E-25	0.209207487	0.000986322
0.640765144	1.01E-29	0.775782998	1.68E-50	-0.188018631	0.003133798
-0.361446829	5.65E-09	-0.701624066	1.24E-37	0.476236426	2.83E-15
0.028486673	0.657261855	0.572119673	1.06E-22	-0.295897598	2.43E-06

-0.427636618	2.59E-12	-0.591506182	1.63E-24	0.163902194	0.010176995
0.471234388	6.02E-15	0.330563829	1.17E-07	0.103669167	0.105502109
0.447652506	1.78E-13	0.502187381	4.66E-17	-0.502037395	4.77E-17
0.293218112	3.02E-06	0.567392224	2.82E-22	-0.333315894	9.08E-08
0.511406572	9.93E-18	0.790798133	1.01E-53	-0.355914184	9.97E-09
-0.321730247	2.64E-07	-0.650226115	7.85E-31	0.621819684	1.31E-27
-0.322450415	2.47E-07	-0.546668775	1.71E-20	0.512165658	8.73E-18
-0.156781451	0.01402278	-0.512554721	8.17E-18	0.381575472	6.52E-10
0.424378292	3.94E-12	0.724803585	3.37E-41	-0.348813245	2.03E-08
-0.412618067	1.72E-11	-0.645185918	3.10E-30	0.462125821	2.30E-14
-0.251133482	7.05E-05	-0.427525087	2.63E-12	0.506001578	2.47E-17
0.551629582	6.57E-21	0.572996277	8.85E-23	-0.270613824	1.75E-05
-0.199493439	0.001699879	-0.540821035	5.18E-20	0.549829828	9.32E-21
0.752983745	4.63E-46	0.907923404	9.61E-94	-0.447196974	1.90E-13
-0.04916508	0.44362686	-0.374655602	1.39E-09	0.275533959	1.21E-05
0.829139389	2.62E-63	0.847741339	7.25E-69	-0.323125891	2.33E-07
0.802665365	1.84E-56	0.862401346	8.47E-74	-0.277623292	1.03E-05
-0.528795755	4.72E-19	-0.551180441	7.17E-21	0.414654849	1.34E-11
0.655833595	1.65E-31	0.884830865	1.44E-82	-0.244858857	0.000107877
-0.256828499	4.74E-05	-0.438001192	6.62E-13	0.55693153	2.32E-21
0.689879852	5.93E-36	0.613540135	9.92E-27	-0.2110568	0.000886753
-0.285047647	5.81E-06	-0.529340371	4.28E-19	0.383525208	5.25E-10
-0.491084516	2.82E-16	-0.661620154	3.18E-32	0.447754192	1.76E-13
-0.070015987	0.274982926	-0.498547113	8.47E-17	0.429285911	2.09E-12
-0.266490092	2.37E-05	-0.392627972	1.87E-10	0.472420572	5.04E-15
0.506347152	2.33E-17	0.421007899	6.05E-12	-0.111634151	0.081181822
0.802618041	1.89E-56	0.934600101	4.48E-111	-0.327063556	1.62E-07
0.617196453	4.09E-27	0.865929047	4.52E-75	-0.342969368	3.61E-08
-0.395776843	1.30E-10	-0.756717187	9.38E-47	0.461199563	2.63E-14
-0.402810972	5.66E-11	-0.701237083	1.42E-37	0.527116008	6.39E-19
-0.526112976	7.64E-19	-0.778020293	5.77E-51	0.391981211	2.01E-10
0.825287532	3.06E-62	0.786483547	9.03E-53	-0.107500868	0.093167979
0.192851798	0.002431961	0.380027657	7.74E-10	-0.388876993	2.87E-10
-0.262255884	3.23E-05	-0.346247766	2.62E-08	0.352441475	1.42E-08
0.367274502	3.07E-09	0.555105782	3.33E-21	-0.188406204	0.003071395
0.344007855	3.26E-08	0.624455482	6.80E-28	-0.354234292	1.18E-08
-0.185830534	0.00350815	-0.579546521	2.22E-23	0.322827768	2.39E-07
-0.251836277	6.72E-05	-0.483924485	8.70E-16	0.244032232	0.000114004
-0.293896412	2.86E-06	-0.465492525	1.41E-14	0.627274603	3.35E-28
0.767253489	8.82E-49	0.87989774	1.72E-80	-0.442901519	3.41E-13

0.529659573	4.04E-19	0.795004098	1.13E-54	-0.408503775	2.85E-11
0.655344299	1.89E-31	0.754414141	2.52E-46	-0.593040196	1.16E-24
-0.290693857	3.71E-06	-0.644461304	3.77E-30	0.234778598	0.000208874
0.393607025	1.67E-10	0.584853712	7.05E-24	-0.127082112	0.046917905
0.605421978	6.81E-26	0.670774195	2.19E-33	-0.123756171	0.053036026
0.366497211	3.33E-09	0.754725223	2.21E-46	-0.365082381	3.87E-09
-0.281404513	7.72E-06	-0.63161916	1.11E-28	0.404160899	4.82E-11
0.688827988	8.31E-36	0.62534161	5.45E-28	-0.355892342	9.99E-09
-0.502227848	4.63E-17	-0.601439457	1.72E-25	0.219938139	0.000525272
0.598975702	3.02E-25	0.81444919	2.25E-59	-0.320832716	2.86E-07
0.547333845	1.51E-20	0.712918712	2.52E-39	-0.180830107	0.004519718
0.720405025	1.71E-40	0.828455623	4.07E-63	-0.223877943	0.000413599
0.649640223	9.22E-31	0.926287958	5.51E-105	-0.396002893	1.26E-10
0.849440667	2.07E-69	0.82843996	4.11E-63	-0.412711279	1.71E-11
0.633637212	6.57E-29	0.897106225	3.54E-88	-0.385868419	4.03E-10
0.408017045	3.03E-11	0.713101748	2.36E-39	-0.453655197	7.70E-14
0.514420727	5.93E-18	0.871138018	5.12E-77	-0.363375726	4.62E-09
0.673750524	8.97E-34	0.799417121	1.08E-55	-0.26464803	2.71E-05
0.696703813	6.42E-37	0.766245923	1.39E-48	-0.27670474	1.11E-05
-0.29167491	3.43E-06	-0.4745931	3.63E-15	0.550877226	7.61E-21
-0.385238419	4.33E-10	-0.508335093	1.67E-17	0.24064783	0.000142656
-0.614383211	8.09E-27	-0.72684937	1.57E-41	0.336430977	6.77E-08
-0.076930092	0.230229788	-0.468667112	8.81E-15	0.484196282	8.34E-16
-0.170147174	0.007605602	-0.405638042	4.03E-11	0.481666174	1.23E-15
0.837174102	1.27E-65	0.722775155	7.14E-41	-0.076581483	0.232355039
0.811061597	1.62E-58	0.727087197	1.43E-41	-0.304893916	1.15E-06
0.629965767	1.69E-28	0.849669182	1.75E-69	-0.430194638	1.86E-12
0.345911415	2.71E-08	0.50086447	5.79E-17	-0.339559105	5.02E-08
0.776326932	1.30E-50	0.928545674	1.45E-106	-0.442557858	3.58E-13
0.590046665	2.25E-24	0.839561877	2.46E-66	-0.432983549	1.29E-12
0.767462452	8.02E-49	0.761307836	1.27E-47	-0.219240807	0.000547734
0.257548476	4.51E-05	0.578403754	2.83E-23	-0.11984485	0.061064035
0.54428833	2.69E-20	0.860372329	4.40E-73	-0.326047091	1.78E-07
0.734738802	7.66E-43	0.639086068	1.58E-29	-0.274257943	1.33E-05
-0.082965759	0.195592659	-0.478568835	1.99E-15	0.30647594	1.00E-06
0.081788331	0.202032824	0.477290913	2.41E-15	-0.486965197	5.41E-16
0.445886937	2.27E-13	0.643209425	5.28E-30	-0.240561269	0.000143471
0.682457215	6.22E-35	0.761173311	1.34E-47	-0.340944352	4.39E-08
0.362352177	5.14E-09	0.623586586	8.46E-28	-0.271719773	1.61E-05
0.617452372	3.84E-27	0.839883644	1.97E-66	-0.444666853	2.68E-13

-0.331743587	1.05E-07	-0.597009595	4.73E-25	0.422103041	5.26E-12
0.341881877	4.01E-08	0.622677825	1.06E-27	-0.369964995	2.31E-09
0.393474771	1.69E-10	0.75982135	2.43E-47	-0.246799272	9.47E-05
-0.160918056	0.011657553	-0.346960501	2.44E-08	0.571219051	1.28E-22
-0.434753442	1.02E-12	-0.851947926	3.16E-70	0.495375307	1.42E-16
0.756336526	1.11E-46	0.899601423	2.10E-89	-0.233232746	0.000230576
-0.206170425	0.001172395	-0.469608818	7.66E-15	0.456075956	5.47E-14
0.746459692	7.05E-45	0.653110673	3.53E-31	-0.507360512	1.97E-17
0.675869997	4.72E-34	0.797457039	3.08E-55	-0.194088887	0.002276969
0.698574577	3.45E-37	0.875821915	7.67E-79	-0.4131894	1.61E-11
-0.285669181	5.53E-06	-0.592972186	1.18E-24	0.202953894	0.001404189
0.422224203	5.18E-12	0.294987046	2.62E-06	-0.238213619	0.00016729
-0.187847458	0.003161722	-0.357206585	8.74E-09	0.488689356	4.12E-16
-0.285190595	5.74E-06	-0.668404055	4.42E-33	0.347957912	2.21E-08
0.587524493	3.93E-24	0.641136033	9.19E-30	0.043028935	0.502618402
0.617530373	3.77E-27	0.671763619	1.63E-33	-0.219659867	0.000534131
-0.06820326	0.287632764	-0.369614171	2.39E-09	0.615457058	6.24E-27
0.586086418	5.39E-24	0.827738577	6.45E-63	-0.391402254	2.15E-10
-0.348782146	2.04E-08	-0.65772497	9.68E-32	0.614168394	8.52E-27
0.355836814	1.01E-08	0.744639924	1.48E-44	-0.417720762	9.15E-12
-0.426217807	3.11E-12	-0.648729599	1.18E-30	0.408006844	3.03E-11
0.112965504	0.077597305	0.468978796	8.41E-15	-0.288260368	4.50E-06
0.389495701	2.67E-10	0.584323319	7.92E-24	-0.216436724	0.000647332
0.672425981	1.34E-33	0.808436823	7.30E-58	-0.308492113	8.45E-07
0.717466854	4.96E-40	0.657617759	9.97E-32	-0.387176971	3.48E-10
-0.146855478	0.021483544	-0.517536387	3.46E-18	0.352389908	1.42E-08
0.484927125	7.44E-16	0.667163237	6.36E-33	-0.302712933	1.38E-06
-0.276972221	1.08E-05	-0.561128319	1.01E-21	0.182931084	0.004066412
0.538215785	8.42E-20	0.874944971	1.71E-78	-0.347915233	2.22E-08
0.58940621	2.60E-24	0.83020197	1.32E-63	-0.427171013	2.75E-12
0.375404729	1.28E-09	0.684407044	3.38E-35	-0.193200507	0.002387328
-0.318670733	3.47E-07	-0.503502472	3.75E-17	0.140855561	0.02749165
-0.273173701	1.45E-05	-0.627888329	2.87E-28	0.276081153	1.16E-05
-0.390475156	2.39E-10	-0.643023451	5.55E-30	0.450503912	1.20E-13
0.595947344	6.02E-25	0.568343202	2.32E-22	-0.271519288	1.64E-05
0.384870349	4.51E-10	0.583305011	9.87E-24	-0.206236424	0.00116803
-0.398762883	9.14E-11	-0.710264076	6.40E-39	0.639620391	1.37E-29
-0.185348642	0.003595843	-0.435064872	9.79E-13	0.161204643	0.011507553
-0.249714704	7.77E-05	-0.492242048	2.34E-16	0.463602846	1.85E-14
0.215350485	0.000690225	0.611417289	1.65E-26	-0.476379171	2.77E-15

-0.35646302	9.43E-09	-0.745274071	1.15E-44	0.462928441	2.04E-14
0.454715079	6.63E-14	0.823413177	9.89E-62	-0.324599732	2.03E-07
-0.233321704	0.000229272	-0.354601542	1.14E-08	0.18298549	0.004055238
0.515432968	4.98E-18	0.587714775	3.77E-24	-0.233493005	0.000226781
-0.277577108	1.04E-05	-0.641359005	8.66E-30	0.617040912	4.25E-27
-0.224361252	0.000401532	-0.61961959	2.26E-27	0.218039424	0.000588529
-0.222526547	0.000449148	-0.325701814	1.84E-07	0.176854749	0.005503674
0.062698459	0.328406213	0.376160837	1.18E-09	-0.572953287	8.93E-23
-0.404145683	4.83E-11	-0.710738998	5.42E-39	0.336622846	6.65E-08
0.83206478	3.89E-64	0.866760217	2.24E-75	-0.28397756	6.32E-06
-0.406107382	3.81E-11	-0.633708327	6.45E-29	0.423719101	4.29E-12
-0.451530314	1.04E-13	-0.649522765	9.53E-31	0.497977447	9.29E-17
0.332671996	9.65E-08	0.701030485	1.52E-37	-0.261790247	3.33E-05
0.627878387	2.87E-28	0.660045741	5.00E-32	-0.284652231	5.99E-06
0.625802327	4.85E-28	0.912340778	3.22E-96	-0.419708049	7.13E-12
0.585706465	5.86E-24	0.832004786	4.05E-64	-0.325526894	1.87E-07
-0.333153084	9.22E-08	-0.531178779	3.07E-19	0.378708443	8.95E-10
-0.205002857	0.001252131	-0.37171532	1.91E-09	0.365699822	3.62E-09
-0.421382973	5.77E-12	-0.654735943	2.25E-31	0.504954312	2.94E-17
-0.218177019	0.000583718	-0.562111541	8.26E-22	0.280969903	7.98E-06
-0.338694678	5.45E-08	-0.645001838	3.26E-30	0.539681905	6.41E-20
-0.307752703	9.01E-07	-0.587533533	3.93E-24	0.197361145	0.001909365
-0.4268485	2.87E-12	-0.753356664	3.95E-46	0.364903733	3.94E-09
0.349829774	1.84E-08	0.531255404	3.03E-19	-0.266614338	2.35E-05
0.759895867	2.35E-47	0.843257497	1.84E-67	-0.322899234	2.37E-07
0.357574757	8.42E-09	0.737766136	2.34E-43	-0.309400701	7.82E-07
0.783875374	3.32E-52	0.78887881	2.69E-53	-0.394642429	1.48E-10
0.792157437	5.00E-54	0.829903517	1.60E-63	-0.426652619	2.94E-12
0.6229067	1.00E-27	0.867901667	8.48E-76	-0.279067338	9.24E-06
0.562736067	7.28E-22	0.646819589	1.99E-30	-0.332096863	1.02E-07
-0.251695524	6.78E-05	-0.402143783	6.13E-11	0.463397506	1.91E-14
0.720145374	1.88E-40	0.918848893	4.11E-100	-0.319974932	3.09E-07
-0.255980465	5.04E-05	-0.562484062	7.66E-22	0.141758689	0.026504174
0.358653128	7.54E-09	0.651433316	5.63E-31	-0.288765156	4.33E-06
0.501979459	4.82E-17	0.352882004	1.36E-08	0.039623813	0.537049088
-0.405794205	3.96E-11	-0.715940007	8.58E-40	0.32384482	2.18E-07
-0.406118477	3.81E-11	-0.650006207	8.34E-31	0.430552929	1.77E-12
-0.194724117	0.002200928	-0.371025939	2.06E-09	0.630201547	1.59E-28
-0.282798669	6.93E-06	-0.561588072	9.17E-22	0.577624587	3.34E-23
-0.343830561	3.32E-08	-0.375530691	1.27E-09	0.400536329	7.42E-11

0.533941177	1.85E-19	0.528220964	5.24E-19	0.015361747	0.810924275
-0.16765077	0.008554437	-0.401791613	6.39E-11	0.587500284	3.95E-24
-0.556234985	2.67E-21	-0.715047398	1.18E-39	0.287632902	4.73E-06
0.63466816	5.03E-29	0.775285719	2.12E-50	-0.328891356	1.37E-07
0.374824198	1.37E-09	0.396917689	1.13E-10	-0.240426477	0.000144747
-0.280803456	8.09E-06	-0.316501274	4.21E-07	0.264912541	2.66E-05
-0.302939297	1.36E-06	-0.638712306	1.75E-29	0.476404368	2.76E-15
-0.331789226	1.05E-07	-0.426599118	2.96E-12	0.65862514	7.50E-32
0.348597746	2.08E-08	0.641470151	8.41E-30	-0.080782875	0.207652819
0.428467964	2.32E-12	0.732576578	1.77E-42	-0.18378457	0.003894289
-0.261354751	3.44E-05	-0.628488515	2.46E-28	0.281044684	7.94E-06
-0.179957406	0.004721026	-0.429402564	2.06E-12	0.160384195	0.011941579
0.350821485	1.67E-08	0.622701164	1.05E-27	-0.424081204	4.09E-12
0.134728061	0.035060249	0.455279668	6.12E-14	-0.362421191	5.11E-09
0.664607633	1.34E-32	0.83383971	1.20E-64	-0.333880229	8.62E-08
0.832986515	2.12E-64	0.820666963	5.39E-61	-0.307201597	9.44E-07
0.844974783	5.41E-68	0.707113737	1.91E-38	-0.298379067	1.98E-06
0.417315403	9.63E-12	0.748976182	2.49E-45	-0.179475601	0.004835588
0.683540055	4.43E-35	0.805199184	4.52E-57	-0.177591983	0.00530784
-0.485698219	6.60E-16	-0.697648619	4.70E-37	0.144028287	0.024156038
-0.455684884	5.78E-14	-0.702940025	7.97E-38	0.269788874	1.86E-05
-0.437824057	6.78E-13	-0.621255955	1.51E-27	0.518312513	3.03E-18
0.725639908	2.47E-41	0.876503364	4.11E-79	-0.365411497	3.74E-09
-0.521383269	1.77E-18	-0.605946733	6.02E-26	0.211776538	0.00085057
0.556440457	2.56E-21	0.838856515	4.01E-66	-0.291950956	3.35E-06
0.665039581	1.18E-32	0.870686582	7.60E-77	-0.33268461	9.64E-08
-0.412333299	1.79E-11	-0.527843522	5.61E-19	0.45203239	9.68E-14
-0.351874902	1.50E-08	-0.514085305	6.28E-18	0.332893877	9.45E-08
-0.333611311	8.84E-08	-0.648696111	1.19E-30	0.472559892	4.93E-15
0.6271139	3.49E-28	0.873788006	4.85E-78	-0.524320469	1.05E-18
0.710167378	6.62E-39	0.714306087	1.54E-39	-0.460690967	2.83E-14
0.610160603	2.23E-26	0.56437641	5.22E-22	-0.271086877	1.69E-05
-0.277803172	1.02E-05	-0.449738492	1.33E-13	0.559366752	1.43E-21
0.473193929	4.49E-15	0.668720964	4.02E-33	-0.195313961	0.002132398
0.620084064	2.02E-27	0.796817797	4.33E-55	-0.310748583	6.96E-07
0.523742961	1.17E-18	0.772557243	7.66E-50	-0.268777524	2.01E-05
0.422891757	4.76E-12	0.785191527	1.72E-52	-0.209654557	0.000961351
0.54160737	4.47E-20	0.814044221	2.86E-59	-0.572822383	9.18E-23
0.500109509	6.56E-17	0.622784798	1.03E-27	-0.084835273	0.185676367
0.521903868	1.61E-18	0.426806647	2.88E-12	-0.376505524	1.14E-09

0.654784429	2.21E-31	0.861544093	1.71E-73	-0.498112023	9.09E-17
-0.3471844	2.39E-08	-0.526566648	7.05E-19	0.638083396	2.06E-29
-0.314464226	5.03E-07	-0.612348513	1.32E-26	0.561663598	9.04E-22
0.312455588	6.00E-07	-0.231561076	0.0002564	0.116934412	0.067665768
0.596261116	5.60E-25	0.879418026	2.71E-80	-0.413719219	1.51E-11
0.585513006	6.11E-24	0.58059946	1.77E-23	-0.599767969	2.52E-25
0.81523092	1.42E-59	0.831743241	4.80E-64	-0.336166293	6.94E-08
0.678141985	2.36E-34	0.695688353	8.98E-37	-0.191701363	0.002584644
0.671349883	1.84E-33	0.931968734	4.59E-109	-0.312262771	6.10E-07
0.740479383	7.96E-44	0.91668875	8.74E-99	-0.422443628	5.04E-12
0.697466993	4.99E-37	0.875155376	1.41E-78	-0.435688067	9.01E-13
-0.394811118	1.45E-10	-0.707751261	1.53E-38	0.496352682	1.21E-16
0.425800744	3.28E-12	0.645969697	2.51E-30	-0.352291244	1.44E-08
-0.176708158	0.005543377	-0.436167646	8.45E-13	0.319224923	3.30E-07
-0.400984216	7.03E-11	-0.509519478	1.37E-17	0.371583034	1.94E-09
0.077007065	0.229762396	-0.43699035	7.57E-13	0.256819014	4.75E-05
-0.271758503	1.61E-05	-0.581373463	1.50E-23	0.479234812	1.79E-15
-0.366710006	3.26E-09	-0.524033104	1.11E-18	0.391534232	2.12E-10
0.704267985	5.07E-38	0.894625906	5.47E-87	-0.411023877	2.10E-11
-0.280747745	8.12E-06	-0.554211108	3.97E-21	0.53321582	2.12E-19
0.463645935	1.84E-14	0.636555425	3.08E-29	-0.313162128	5.64E-07
-0.128289167	0.044848708	-0.501112105	5.56E-17	0.510987894	1.07E-17
-0.260492436	3.66E-05	-0.469074519	8.29E-15	0.194912245	0.002178856
0.562967509	6.95E-22	0.580413661	1.84E-23	0.023622081	0.712944439
-0.218899615	0.000559045	-0.4084971	2.86E-11	0.451151301	1.09E-13
-0.338633417	5.49E-08	-0.562043668	8.37E-22	0.53615386	1.23E-19
0.437390813	7.18E-13	0.683150563	5.01E-35	-0.309359492	7.85E-07
0.483295289	9.59E-16	0.721623737	1.09E-40	-0.290647439	3.72E-06
-0.341033666	4.36E-08	-0.630000278	1.67E-28	0.425083802	3.60E-12
-0.460356828	2.96E-14	-0.604973382	7.56E-26	0.362313074	5.16E-09
-0.370946316	2.08E-09	-0.5590642	1.52E-21	0.272764985	1.49E-05
0.547277743	1.52E-20	0.826121909	1.80E-62	-0.265863123	2.48E-05
0.663318216	1.95E-32	0.821676813	2.90E-61	-0.497659014	9.78E-17
0.495805048	1.32E-16	0.814305655	2.45E-59	-0.279529919	8.92E-06
-0.400773688	7.21E-11	-0.592714873	1.24E-24	0.170778782	0.007380921
-0.137447509	0.031507097	-0.385701002	4.11E-10	0.217053693	0.000624081
-0.442947759	3.39E-13	-0.575354651	5.39E-23	0.240553232	0.000143546
-0.434861963	1.01E-12	-0.501109753	5.56E-17	0.174807209	0.006082005
0.635052022	4.55E-29	0.818817592	1.66E-60	-0.26589038	2.48E-05
-0.489219943	3.79E-16	-0.781597161	1.02E-51	0.588639614	3.08E-24

0.547021958	1.60E-20	0.725065521	3.05E-41	-0.448442946	1.60E-13
0.086162761	0.178862826	0.560871386	1.06E-21	-0.389963552	2.53E-10
-0.278487098	9.66E-06	-0.447825321	1.74E-13	0.64829665	1.33E-30
-0.487601025	4.89E-16	-0.598403945	3.44E-25	0.492817706	2.14E-16
-0.316110733	4.35E-07	-0.620915315	1.64E-27	0.609253744	2.76E-26
-0.400369669	7.56E-11	-0.629315364	1.99E-28	0.46875599	8.69E-15
0.544348545	2.66E-20	0.614344582	8.17E-27	-0.105445623	0.099632467
0.345842731	2.73E-08	0.647172361	1.81E-30	-0.305421645	1.10E-06
0.558701775	1.63E-21	0.829308392	2.35E-63	-0.264815386	2.68E-05
0.425913684	3.23E-12	0.804921312	5.27E-57	-0.407886829	3.07E-11
0.61396077	8.96E-27	0.535655373	1.35E-19	-0.059295356	0.355389419
0.701708694	1.21E-37	0.755081234	1.90E-46	-0.231838713	0.000251933
-0.344931591	2.98E-08	-0.584809716	7.12E-24	0.581131739	1.58E-23
-0.294729615	2.67E-06	-0.656347698	1.43E-31	0.495203576	1.46E-16
-0.447186504	1.90E-13	-0.545571441	2.11E-20	0.601234874	1.80E-25
0.737467246	2.63E-43	0.629593578	1.86E-28	-0.288408898	4.45E-06
-0.3174688	3.86E-07	-0.484724028	7.68E-16	0.476217143	2.84E-15
0.430594405	1.76E-12	0.72164282	1.08E-40	-0.333569728	8.87E-08
0.57539678	5.35E-23	0.858438625	2.07E-72	-0.41575956	1.17E-11
-0.318713262	3.46E-07	-0.451788551	1.00E-13	0.434479474	1.06E-12
0.429969697	1.91E-12	0.695251906	1.04E-36	-0.085505244	0.182214172
0.585301387	6.40E-24	0.634153924	5.75E-29	-0.201108067	0.001555469
-0.141609723	0.026664924	-0.424256376	4.00E-12	0.491991308	2.44E-16
0.433207298	1.25E-12	0.677219702	3.13E-34	-0.199398853	0.001708707
0.804827222	5.56E-57	0.768870684	4.22E-49	-0.293492757	2.96E-06
0.617215477	4.07E-27	0.875588943	9.49E-79	-0.232333002	0.000244158
0.477538165	2.33E-15	0.630791271	1.37E-28	-0.179461385	0.004839006
0.700164106	2.03E-37	0.868732581	4.15E-76	-0.29574495	2.46E-06
0.335733133	7.23E-08	0.589649499	2.46E-24	-0.408081868	3.00E-11
-0.207480465	0.001088497	-0.406210766	3.77E-11	0.250337389	7.44E-05
0.321265394	2.75E-07	0.630638797	1.42E-28	-0.277865271	1.01E-05
-0.339127849	5.23E-08	-0.66339444	1.91E-32	0.37216438	1.82E-09
-0.180021126	0.004706059	-0.642168149	6.98E-30	0.30095745	1.60E-06
-0.09550365	0.136057885	-0.34518984	2.91E-08	0.463476045	1.89E-14
0.406857385	3.48E-11	0.420075821	6.81E-12	0.019872547	0.756945487
0.332319625	9.97E-08	0.302202245	1.44E-06	-0.510239983	1.21E-17
0.244757867	0.000108609	0.474871466	3.48E-15	-0.328961491	1.36E-07
0.36283334	4.89E-09	0.716308059	7.52E-40	-0.281941284	7.40E-06
-0.196463894	0.002004357	-0.388535935	2.98E-10	0.244274721	0.000112174
0.684220132	3.58E-35	0.634935041	4.70E-29	-0.111886062	0.080493522

-0.230634087	0.000271858	-0.64962696	9.26E-31	0.629984353	1.68E-28
-0.285888359	5.44E-06	-0.647304797	1.75E-30	0.647641622	1.59E-30
-0.439719803	5.25E-13	-0.653441241	3.22E-31	0.392221081	1.96E-10
0.675611294	5.11E-34	0.650480276	7.32E-31	-0.196914902	0.001956083
-0.091991753	0.151121698	-0.379826315	7.91E-10	0.314733548	4.92E-07
-0.484951493	7.41E-16	-0.680378001	1.19E-34	0.506068291	2.44E-17
-0.12130041	0.057966838	-0.474627361	3.62E-15	0.535427952	1.41E-19
-0.577355134	3.53E-23	-0.565097018	4.51E-22	-0.052290497	0.415159524
0.734483824	8.46E-43	0.911385439	1.13E-95	-0.434942085	9.95E-13
0.333448641	8.97E-08	0.702233695	1.01E-37	-0.179973496	0.004717243
-0.469579547	7.70E-15	-0.670100614	2.67E-33	0.407487917	3.23E-11
0.433674482	1.18E-12	0.716401985	7.27E-40	-0.270555103	1.76E-05
-0.446150073	2.19E-13	-0.704264507	5.08E-38	0.329996432	1.24E-07
0.402078814	6.18E-11	0.54703925	1.59E-20	-0.213812511	0.000755457
0.426283355	3.08E-12	0.579698845	2.15E-23	-0.390444232	2.40E-10
0.274733623	1.29E-05	0.658756591	7.22E-32	-0.414715576	1.33E-11
0.476809636	2.60E-15	0.891824995	1.11E-85	-0.379553737	8.15E-10
-0.248043972	8.70E-05	-0.502808284	4.20E-17	0.543242347	3.28E-20
-0.316424572	4.24E-07	-0.72578692	2.33E-41	0.595696101	6.37E-25
-0.362951187	4.83E-09	-0.602109075	1.47E-25	0.397625229	1.04E-10
-0.288329253	4.48E-06	-0.474299019	3.80E-15	0.632249956	9.41E-29
-0.464168817	1.71E-14	-0.761131127	1.37E-47	0.411473148	1.99E-11
0.734796854	7.49E-43	0.623395554	8.87E-28	-0.302408397	1.42E-06
-0.018847091	0.769123912	0.351249658	1.60E-08	-0.47698404	2.53E-15
0.701648566	1.23E-37	0.856013582	1.39E-71	-0.185616128	0.003546925
-0.477209849	2.44E-15	-0.667380356	5.97E-33	0.228061077	0.000319432
0.299236892	1.85E-06	0.487648814	4.86E-16	-0.258567292	4.20E-05
0.622537623	1.10E-27	0.751543025	8.51E-46	-0.367327348	3.05E-09
-0.234775069	0.000208921	-0.689938232	5.82E-36	0.589946213	2.31E-24
0.542654862	3.67E-20	0.906050694	9.86E-93	-0.349244436	1.95E-08
0.669432821	3.26E-33	0.826482368	1.44E-62	-0.247934573	8.77E-05
0.182134246	0.004233273	0.598752676	3.18E-25	-0.240792856	0.000141301
-0.164974906	0.009686931	-0.611384905	1.66E-26	0.521077095	1.87E-18
-0.472038751	5.33E-15	-0.784307601	2.68E-52	0.57408835	7.04E-23
-0.347882986	2.23E-08	-0.482068301	1.16E-15	0.277403308	1.05E-05
0.415753272	1.17E-11	0.443630889	3.09E-13	-0.244036033	0.000113975
0.666759749	7.16E-33	0.711238403	4.55E-39	-0.379219239	8.46E-10
0.414712754	1.33E-11	0.494852121	1.54E-16	-0.054755709	0.393482028
0.587314655	4.12E-24	0.802097114	2.51E-56	-0.399765946	8.12E-11
0.728720377	7.73E-42	0.731489698	2.69E-42	-0.301021115	1.59E-06

0.135121623	0.034525887	0.306362382	1.01E-06	-0.536271554	1.21E-19
0.624508653	6.71E-28	0.861384464	1.94E-73	-0.266425585	2.38E-05
0.332850195	9.49E-08	0.623342589	8.99E-28	-0.25314513	6.14E-05
0.437346307	7.22E-13	0.609867035	2.39E-26	-0.127714566	0.045823977
0.753452626	3.80E-46	0.84195329	4.64E-67	-0.338250548	5.69E-08
0.397180814	1.10E-10	0.638702324	1.75E-29	-0.153917467	0.015897747
0.531694708	2.79E-19	0.779792198	2.45E-51	-0.322343765	2.50E-07
0.472782346	4.77E-15	0.761214174	1.32E-47	-0.423255658	4.55E-12
0.617396939	3.89E-27	0.87576615	8.07E-79	-0.390298077	2.44E-10
0.456608447	5.07E-14	0.689965314	5.77E-36	-0.334775889	7.92E-08
0.380592684	7.27E-10	0.446275456	2.15E-13	-0.075688839	0.237859877
0.694427114	1.36E-36	0.715717871	9.29E-40	-0.263027118	3.05E-05
0.63046685	1.49E-28	0.873770773	4.93E-78	-0.321637915	2.66E-07
0.727269019	1.34E-41	0.7942162	1.71E-54	-0.222995211	0.00043651
0.690012355	5.69E-36	0.879769827	1.95E-80	-0.297186665	2.19E-06
0.405750273	3.98E-11	0.772383723	8.31E-50	-0.210095807	0.000937276
0.407452917	3.24E-11	0.71639177	7.30E-40	-0.157476052	0.01359839
-0.403883083	4.98E-11	-0.58751513	3.94E-24	0.29608759	2.39E-06
-0.499540193	7.20E-17	-0.487243213	5.18E-16	0.269703079	1.87E-05
-0.261652025	3.37E-05	-0.384384138	4.76E-10	0.334018021	8.50E-08
-0.032333879	0.614508245	-0.408754346	2.77E-11	0.159189552	0.012599343
0.465590945	1.39E-14	0.825259285	3.11E-62	-0.28298176	6.83E-06
0.362320972	5.16E-09	0.627631196	3.06E-28	-0.1110704	0.082739374
0.096170466	0.133332524	0.422736332	4.86E-12	-0.418327816	8.48E-12
-0.462396203	2.21E-14	-0.66910173	3.59E-33	0.539992102	6.05E-20
0.521940749	1.60E-18	0.666488917	7.76E-33	-0.395944547	1.27E-10
-0.362772879	4.92E-09	-0.667287484	6.14E-33	0.212596313	0.000811018
0.025812577	0.687660847	-0.447064805	1.93E-13	0.527257998	6.23E-19
0.103653096	0.105556428	0.525799295	8.09E-19	-0.254615349	5.54E-05
0.033847514	0.59802757	-0.402662874	5.76E-11	0.283003713	6.82E-06
0.353531537	1.27E-08	0.63004899	1.65E-28	-0.221382429	0.000481438
-0.230337006	0.000276993	-0.326540526	1.70E-07	0.455824203	5.67E-14
0.439089726	5.72E-13	0.78004076	2.17E-51	-0.286852689	5.04E-06
0.52516489	9.05E-19	0.567849243	2.57E-22	-0.386040111	3.96E-10
0.471056443	6.18E-15	0.66079873	4.03E-32	-0.377221009	1.05E-09
0.619234305	2.48E-27	0.672069981	1.49E-33	-0.181700217	0.004326734
0.371022437	2.06E-09	0.639178384	1.54E-29	-0.225746698	0.00036873
0.38395173	5.00E-10	0.736423563	3.97E-43	-0.132418173	0.038339933
0.731433413	2.75E-42	0.837765214	8.48E-66	-0.157886634	0.01335287
0.791568741	6.78E-54	0.8988278	5.08E-89	-0.349706972	1.86E-08

0.608575946	3.24E-26	0.763815098	4.16E-48	-0.275573814	1.21E-05
-0.273480762	1.41E-05	-0.560667949	1.10E-21	0.437777409	6.82E-13
0.615155407	6.72E-27	0.860847799	3.00E-73	-0.381054521	6.91E-10
-0.415700356	1.18E-11	-0.64404206	4.22E-30	0.326500143	1.71E-07
0.679087906	1.77E-34	0.83854528	4.97E-66	-0.333654604	8.80E-08
-0.455727571	5.75E-14	-0.650846857	6.62E-31	0.293247401	3.02E-06
0.605460731	6.74E-26	0.700345964	1.91E-37	-0.192882097	0.002428053
0.66108079	3.72E-32	0.91854888	6.32E-100	-0.284045219	6.28E-06
0.675154389	5.87E-34	0.750590027	1.27E-45	-0.43779828	6.80E-13
0.356510283	9.38E-09	0.649505918	9.57E-31	-0.251947335	6.66E-05
0.706591019	2.29E-38	0.859851954	6.69E-73	-0.251888653	6.69E-05
-0.600899716	1.94E-25	-0.784278636	2.72E-52	0.413446963	1.56E-11
-0.293762917	2.89E-06	-0.309977567	7.44E-07	0.694268258	1.43E-36
-0.237825583	0.000171566	-0.544114252	2.78E-20	0.163637767	0.010301117
0.673594394	9.40E-34	0.855678288	1.81E-71	-0.284621791	6.00E-06
0.550758824	7.78E-21	0.685016206	2.79E-35	-0.006115339	0.924130901
0.712752408	2.67E-39	0.838532728	5.01E-66	-0.155128544	0.0150797
0.391624912	2.10E-10	0.786559916	8.69E-53	-0.393887637	1.61E-10
0.767863191	6.68E-49	0.899679644	1.92E-89	-0.372621932	1.73E-09
0.634521301	5.23E-29	0.905228952	2.70E-92	-0.350817526	1.67E-08
0.781598641	1.02E-51	0.878786369	4.92E-80	-0.258888793	4.10E-05
0.557580272	2.04E-21	0.819778376	9.27E-61	-0.27170515	1.61E-05
-0.57029513	1.55E-22	-0.842619235	2.90E-67	0.466831111	1.16E-14
-0.509837411	1.30E-17	-0.420139397	6.75E-12	0.195746177	0.002083423
0.295412087	2.53E-06	0.692215672	2.79E-36	-0.112362802	0.079203814
-0.31555276	4.57E-07	-0.521534342	1.72E-18	0.534381663	1.71E-19
0.292653808	3.17E-06	0.768323599	5.42E-49	-0.454178118	7.16E-14
0.460998598	2.70E-14	0.628927703	2.20E-28	-0.310132091	7.34E-07
-0.219273157	0.000546673	-0.315578008	4.56E-07	0.121316992	0.057932316
0.415526016	1.20E-11	0.723459684	5.55E-41	-0.253558602	5.96E-05
0.745157955	1.20E-44	0.688668238	8.75E-36	-0.158256373	0.013135114
0.475691036	3.08E-15	0.737096632	3.04E-43	-0.157243193	0.013739389
0.611340525	1.68E-26	0.707792904	1.51E-38	-0.258933565	4.09E-05
-0.282062471	7.33E-06	-0.423308199	4.52E-12	0.668402584	4.42E-33
-0.268345075	2.07E-05	-0.403417887	5.27E-11	0.668071288	4.87E-33
-0.070801336	0.269621475	0.406059597	3.83E-11	-0.306466756	1.01E-06
-0.714882566	1.25E-39	-0.6734898	9.70E-34	0.196402505	0.002011011
0.649422241	9.79E-31	0.465887051	1.33E-14	-0.212065398	0.000836435
-0.309014924	8.08E-07	-0.48808946	4.53E-16	0.009092806	0.887395971
-0.042038367	0.512513436	-0.313396974	5.53E-07	0.304869418	1.15E-06

0.57610858	4.60E-23	0.73251687	1.81E-42	-0.398380017	9.56E-11
-0.239372049	0.000155109	-0.451969343	9.76E-14	0.43429309	1.08E-12
0.547529372	1.45E-20	0.503392537	3.82E-17	-0.292961869	3.09E-06
0.698357402	3.71E-37	0.920555899	3.45E-101	-0.409570669	2.51E-11
-0.262505779	3.17E-05	-0.424598278	3.83E-12	0.363730818	4.46E-09
0.841029963	8.87E-67	0.834155534	9.72E-65	-0.31306205	5.69E-07
0.510176284	1.22E-17	0.834784057	6.39E-65	-0.268720086	2.01E-05
-0.439503865	5.41E-13	-0.65456161	2.36E-31	0.504135018	3.37E-17
-0.287442356	4.81E-06	-0.53773564	9.21E-20	0.414286468	1.40E-11
-0.439370697	5.51E-13	-0.810250359	2.59E-58	0.39536817	1.36E-10
0.124538216	0.051540893	0.59622088	5.65E-25	-0.305869435	1.06E-06
0.560277751	1.19E-21	0.656862281	1.23E-31	-0.192936178	0.002421092
0.373181803	1.63E-09	0.685794148	2.18E-35	-0.428338785	2.36E-12
0.527894847	5.56E-19	0.635254496	4.32E-29	-0.428186665	2.41E-12
-0.168808024	0.008102237	-0.644638219	3.60E-30	0.348274365	2.15E-08
-0.153911302	0.015902009	-0.433124639	1.26E-12	0.060124645	0.34868921
-0.19842255	0.001802309	-0.497955293	9.32E-17	0.075271764	0.240463066
0.292041514	3.33E-06	-0.068063018	0.288627421	0.115236653	0.071778986
-0.132632568	0.038024991	-0.379641916	8.07E-10	0.148566661	0.019993534
-0.384250839	4.84E-10	-0.682446322	6.24E-35	0.206701479	0.001137694
-0.106140826	0.097407141	-0.481714944	1.23E-15	0.065869843	0.30448213
-0.114776284	0.072928757	-0.398333402	9.61E-11	0.14007089	0.028375049
0.321140123	2.78E-07	0.37808673	9.58E-10	0.075378299	0.239796234
-0.306358925	1.01E-06	-0.666606974	7.49E-33	0.226953807	0.000342198
-0.090640217	0.157244105	-0.361746854	5.48E-09	0.221485299	0.000478449
-0.12404311	0.052483319	-0.303186568	1.33E-06	0.179886996	0.004737614
-0.143096577	0.025097367	-0.431227238	1.62E-12	0.148689208	0.019890352
-0.306423721	1.01E-06	-0.609430311	2.65E-26	0.256770177	4.76E-05
-0.126396713	0.048127976	-0.353412927	1.28E-08	0.120229276	0.06023309
0.005543749	0.931203852	-0.475761606	3.05E-15	0.169241766	0.007938309
-0.126512862	0.0479211	-0.406458292	3.65E-11	0.210841429	0.000897853
-0.310104769	7.36E-07	-0.710294059	6.33E-39	0.257356093	4.57E-05
-0.084843942	0.185631264	-0.442024995	3.85E-13	0.15505714	0.015126889
-0.27731752	1.06E-05	-0.522080361	1.56E-18	0.190027282	0.002822411
-0.175840361	0.00578372	-0.676960501	3.39E-34	0.196532723	0.00199692
-0.130620751	0.041068018	-0.6275276	3.14E-28	0.317691649	3.79E-07
-0.297261905	2.17E-06	-0.394151377	1.57E-10	0.224251589	0.000404241
-0.456214076	5.36E-14	-0.798117025	2.17E-55	0.226332068	0.000355634
-0.171782235	0.007036117	-0.423950261	4.16E-12	0.079366816	0.215757707
-0.10233047	0.110102892	-0.476272162	2.82E-15	0.138796057	0.029862095

-0.11891396	0.063115315	-0.500196949	6.46E-17	0.157847542	0.013376077
-0.257292886	4.59E-05	-0.625648626	5.04E-28	0.182849465	0.004083226
-0.300709612	1.63E-06	-0.725904786	2.23E-41	0.195635315	0.002095886
-0.297952821	2.05E-06	-0.660449161	4.46E-32	0.319271712	3.29E-07
-0.284838655	5.90E-06	-0.660673587	4.18E-32	0.167834766	0.008481073
-0.221829114	0.000468583	-0.595179141	7.16E-25	0.271648364	1.62E-05
-0.119110065	0.062678543	-0.587216115	4.21E-24	0.123068514	0.054380294
-0.219403981	0.0005424	-0.558982561	1.55E-21	0.089648559	0.161853786
-0.165038882	0.009658376	-0.493620728	1.88E-16	0.189114057	0.002960324
-0.221068191	0.000490676	-0.564243535	5.37E-22	0.106377475	0.096658709
-0.124269981	0.052049712	-0.482374832	1.11E-15	-0.086538886	0.17696633
-0.296307135	2.35E-06	-0.692198719	2.81E-36	0.311043391	6.78E-07
-0.312022362	6.23E-07	-0.471717434	5.60E-15	0.11367236	0.07574689
-0.284764983	5.94E-06	-0.583225312	1.00E-23	0.055772853	0.384740201
0.085152517	0.184030974	-0.182966852	0.004059063	0.261013453	3.53E-05
-0.015310627	0.811541701	-0.422020461	5.32E-12	0.058408341	0.362644735
-0.103650019	0.105566831	-0.27886074	9.39E-06	0.055068007	0.39078535
-0.090360623	0.158533661	-0.257504404	4.52E-05	0.27793109	1.01E-05
-0.007811558	0.903178767	-0.294803013	2.66E-06	0.183529977	0.003944932
0.433613772	1.19E-12	0.639133796	1.56E-29	-0.109823639	0.086269475
-0.123109056	0.054300265	-0.222435977	0.000451629	0.111267339	0.082192551
-0.267868542	2.14E-05	-0.578975535	2.50E-23	0.083890613	0.190639856
-0.158208698	0.013163015	-0.494916525	1.53E-16	0.134864616	0.034874051
-0.227396371	0.000332923	-0.534536578	1.66E-19	0.250645069	7.29E-05
-0.016175859	0.801107624	0.123613533	0.05331257	-0.002316279	0.97122644
-0.226696706	0.000347696	-0.478392568	2.04E-15	0.183495857	0.003951764
-0.256357451	4.90E-05	-0.673408605	9.94E-34	0.365228407	3.81E-09
-0.247139449	9.25E-05	-0.61708233	4.21E-27	0.340889049	4.42E-08
-0.141206086	0.027104704	-0.366141442	3.46E-09	0.093731664	0.143507882
-0.414460812	1.37E-11	-0.749194382	2.28E-45	0.390725893	2.32E-10
-0.241239067	0.000137208	-0.611976625	1.44E-26	0.308520676	8.43E-07
0.118987285	0.062951713	-0.374595779	1.40E-09	0.238921825	0.000159741
-0.068491601	0.285594945	-0.477041661	2.51E-15	0.278732642	9.48E-06
-0.389498756	2.67E-10	-0.479157194	1.82E-15	0.080923852	0.206858101
0.124244888	0.052097525	0.600099451	2.34E-25	-0.204249216	0.001306211
-0.067330139	0.29386279	-0.595317603	6.94E-25	0.159528975	0.012409292
0.134133924	0.035880226	-0.316934216	4.05E-07	0.325095144	1.94E-07
-0.173222107	0.006566459	-0.253300563	6.07E-05	0.022110946	0.730573144
-0.296623401	2.29E-06	-0.625689883	4.99E-28	0.151317319	0.017786105
-0.335889343	7.13E-08	-0.738869326	1.51E-43	0.319101838	3.34E-07

-0.357388426	8.58E-09	-0.691188364	3.89E-36	0.288449258	4.44E-06
-0.155883028	0.014588905	-0.225483843	0.000374756	0.289544545	4.07E-06
-0.319006754	3.37E-07	-0.55812965	1.83E-21	0.167189338	0.008740906
0.001576766	0.980410633	-0.350648399	1.70E-08	0.259764315	3.85E-05
0.084702389	0.18636878	-0.377950152	9.72E-10	0.12274953	0.055013387
-0.265165986	2.61E-05	-0.692325192	2.69E-36	0.221225518	0.00048603
-0.327790375	1.52E-07	-0.550197489	8.68E-21	-0.019648101	0.759606027
-0.219940097	0.00052521	-0.668268734	4.60E-33	0.471133287	6.11E-15
-0.077080491	0.229317158	-0.567852545	2.57E-22	0.366298285	3.40E-09
-0.179741039	0.004772168	-0.440010639	5.05E-13	0.150510326	0.018410596
-0.20368631	0.001347992	-0.547785491	1.38E-20	0.19569288	0.002089406
-0.149353006	0.019339444	-0.417282394	9.67E-12	0.343687558	3.37E-08
-0.23634937	0.000188786	-0.547475994	1.47E-20	0.079667662	0.214017135
-0.004823285	0.940126634	-0.088000778	0.16973649	-0.006676967	0.917187009
-0.325802792	1.82E-07	-0.731124196	3.09E-42	0.340318665	4.67E-08
-0.387388409	3.40E-10	-0.735704732	5.26E-43	0.273244629	1.44E-05
-0.194521202	0.002224963	-0.359797347	6.70E-09	-0.000485272	0.993970558
-0.249125436	8.09E-05	-0.560440614	1.15E-21	0.087567166	0.171857591
-0.249668222	7.79E-05	-0.595436116	6.75E-25	0.264622381	2.72E-05
-0.146812173	0.021522466	-0.391724004	2.07E-10	0.026426743	0.680632294
-0.134789308	0.034976632	-0.358661408	7.53E-09	0.122655865	0.055200441
-0.23082166	0.000268662	-0.531721478	2.78E-19	0.286064358	5.36E-06
-0.300954964	1.60E-06	-0.571038351	1.33E-22	0.082807184	0.196451192
-0.269388511	1.92E-05	-0.704980968	3.98E-38	0.331865999	1.04E-07
-0.252887277	6.25E-05	-0.64301392	5.57E-30	0.207040726	0.001116023
-0.093952748	0.142561744	-0.239061649	0.000158289	0.067830656	0.290280508
0.015742535	0.806328914	-0.423047676	4.67E-12	0.133956894	0.036127668
-0.229556734	0.000290913	-0.509548331	1.36E-17	0.111021692	0.082875069
-0.280569472	8.23E-06	-0.460759397	2.80E-14	0.106865792	0.095128791
-0.195281222	0.00213615	-0.628538963	2.43E-28	0.24265121	0.000124974
-0.21962969	0.0005351	-0.609229177	2.78E-26	0.358701428	7.50E-09
-0.223877342	0.000413614	-0.504742853	3.05E-17	0.227325677	0.000334389
-0.266273884	2.41E-05	-0.586406754	5.03E-24	0.182714951	0.004111075
-0.154309939	0.015628495	-0.511893039	9.14E-18	0.268408654	2.06E-05
-0.227038668	0.000340401	-0.578410385	2.82E-23	0.079760224	0.213483639
0.204750585	0.001270001	-0.153592773	0.016123556	0.277370219	1.05E-05
0.148825438	0.019776193	0.202269549	0.001458629	-0.01361584	0.832074642
-0.290432406	3.79E-06	-0.736292038	4.18E-43	0.371479315	1.96E-09
-0.237165433	0.000179077	-0.652512216	4.17E-31	0.281789288	7.49E-06
-0.016556872	0.796523927	-0.22882358	0.000304582	0.357842196	8.19E-09

-0.200532223	0.001605631	-0.565661442	4.02E-22	0.308543624	8.42E-07
-0.241170566	0.000137829	-0.716343745	7.42E-40	0.242149307	0.0001292
-0.153330691	0.01630786	-0.39492092	1.43E-10	0.036656178	0.567987683
-0.381734535	6.40E-10	-0.734765984	7.58E-43	0.346078243	2.67E-08
0.390180763	2.47E-10	0.627778268	2.95E-28	-0.22306518	0.000434652
-0.330642994	1.17E-07	-0.648437394	1.28E-30	0.201488698	0.001523104
0.110294031	0.084923654	-0.377135481	1.06E-09	0.243358945	0.000119235
-0.281883332	7.44E-06	-0.661593094	3.21E-32	0.145740254	0.022505532
-0.355141651	1.08E-08	-0.776701122	1.08E-50	0.351734465	1.52E-08
0.158638292	0.012913467	-0.398992703	8.90E-11	0.254878873	5.44E-05
-0.312307359	6.08E-07	-0.645609368	2.77E-30	0.167113062	0.008772075
-0.052311479	0.414972116	-0.535387966	1.42E-19	0.298814614	1.91E-06
-0.468560263	8.95E-15	-0.787455686	5.53E-53	0.317978577	3.69E-07
0.266612634	2.35E-05	0.437927205	6.68E-13	-0.029762159	0.642954563
-0.102503059	0.109501047	-0.41051898	2.23E-11	0.189696301	0.002871707
-0.151754179	0.0174558	-0.511715882	9.42E-18	0.294270049	2.78E-06
0.316945856	4.04E-07	0.548310202	1.25E-20	-0.095904363	0.134415042
-0.265299339	2.59E-05	-0.654129459	2.66E-31	0.37197907	1.86E-09
-0.139230223	0.029348347	-0.406057044	3.84E-11	0.086373842	0.177796673
-0.133727704	0.036450164	-0.270816832	1.72E-05	0.326639319	1.69E-07
-0.359238542	7.10E-09	-0.719710104	2.20E-40	0.23815492	0.00016793
-0.166514806	0.009019987	-0.363308419	4.66E-09	0.155886337	0.014586784
-0.05107507	0.426100351	-0.487908549	4.66E-16	0.35927903	7.07E-09
-0.403482283	5.22E-11	-0.764879493	2.58E-48	0.241335101	0.000136342
-0.020498386	0.749542109	-0.049038679	0.444801003	0.018069092	0.778401617
-0.062097293	0.333074277	-0.323961058	2.16E-07	0.39749537	1.06E-10
-0.212599288	0.000810878	-0.680891239	1.01E-34	0.252602742	6.37E-05
-0.488539415	4.22E-16	-0.718356043	3.59E-40	0.225116429	0.000383331
-0.106150819	0.097375444	-0.469629081	7.64E-15	0.169671617	0.007778772
-0.366642538	3.28E-09	-0.62823318	2.63E-28	0.196976137	0.001949611
-0.187364076	0.003241799	-0.441286769	4.25E-13	0.03262111	0.611365577
-0.156692665	0.014077855	-0.375797191	1.23E-09	-0.058929793	0.358368437
-0.386696143	3.67E-10	-0.705995316	2.81E-38	0.263419077	2.97E-05
-0.194693506	0.002204539	-0.524108141	1.09E-18	0.219980766	0.000523927
-0.231798346	0.000252578	-0.518882061	2.74E-18	0.062952332	0.326447612
-0.28134369	7.75E-06	-0.668740844	4.00E-33	0.353386051	1.29E-08
-0.050426979	0.432001905	-0.252808669	6.28E-05	0.136578591	0.032607452
-0.140904625	0.027437205	-0.351367913	1.58E-08	0.073288723	0.253112964
-0.272657137	1.50E-05	-0.623703793	8.21E-28	0.190313944	0.002780338
-0.04292156	0.50368613	-0.253518797	5.98E-05	-0.078054972	0.223465905

-0.246683096	9.54E-05	-0.616763844	4.54E-27	0.187316166	0.003249835
-0.200270895	0.00162888	-0.685698817	2.25E-35	0.260820556	3.57E-05
-0.128162835	0.045061625	-0.571390175	1.24E-22	0.098414294	0.124469423
-0.23282067	0.000236706	-0.512196521	8.68E-18	0.103321641	0.10668165
